# The effect of 3-nitrooxypropanol, a potent methane inhibitor, on ruminal microbial gene expression profiles in dairy cows

**DOI:** 10.1186/s40168-022-01341-9

**Published:** 2022-09-13

**Authors:** Dipti W. Pitta, Nagaraju Indugu, Audino Melgar, Alexander Hristov, Krishna Challa, Bonnie Vecchiarelli, Meagan Hennessy, Kapil Narayan, Stephane Duval, Maik Kindermann, Nicola Walker

**Affiliations:** 1 Department of Clinical Studies, School of Veterinary Medicine, University of Pennsylvania, New Bolton Center, Kennett Square, PA 19348 USA; 2 Department of Animal Science, The Pennsylvania State University, State College, PA 16801 USA; 3 Research Centre for Animal Nutrition and Health, DSM Nutritional Products, CH-4303 Kaiseraugst, Switzerland

**Keywords:** Enteric methane, Hydrogenases, Methane mitigation, Ruminal methanogenesis, Total and metabolically active microbes

## Abstract

**Background:**

Enteric methane emissions from dairy cows are an environmental problem as well as a gross feed energy loss to the animal. Methane is generated in the rumen by methanogenic archaea from hydrogen (H_2_) + carbon dioxide and from H_2_ + methanol or methylamines. The methanogenic substrates are provided by non-methanogens during feed fermentation. Methane mitigation approaches have yielded variable results, partially due to an incomplete understanding of the contribution of hydrogenotrophic and methylotrophic archaea to methanogenesis. Research indicates that 3-nitrooxypropanol (3-NOP) reduces enteric methane formation in dairy cows by inhibiting methyl-coenzyme M reductase (MCR), the enzyme responsible for methane formation. The purpose of this study was to utilize metagenomic and metatranscriptomic approaches to investigate the effect of 3-NOP on the rumen microbiome and to determine the fate of H_2_ that accumulates less than expected under inhibited methanogenesis.

**Results:**

The inhibitor 3-NOP was more inhibitory on *Methanobrevibacter* species than methanol-utilizing *Methanosphaera* and tended to reduce the gene expression of MCR. Under inhibited methanogenesis by 3-NOP, fluctuations in H_2_ concentrations were accompanied by changes in the expression of [FeFe] hydrogenases in H_2_-producing bacteria to regulate the amount of H_2_ production. No previously reported alternative H_2_ sinks increased under inhibited methanogenesis except for a significant increase in gene expression of enzymes involved in the butyrate pathway.

**Conclusion:**

By taking a metatranscriptomic approach, this study provides novel insights on the contribution of methylotrophic methanogens to total methanogenesis and regulation of H_2_ metabolism under normal and inhibited methanogenesis by 3-NOP in the rumen.

Video Abstract

**Supplementary Information:**

The online version contains supplementary material available at 10.1186/s40168-022-01341-9.

## Background

Methane (CH4) accounts for 20% of total global greenhouse gas (GHG) emissions and is the second largest contributor to global warming [1]. About 14.5% of all anthropogenic GHG emissions originate from the livestock sector [2] and enteric CH_4_ from livestock production is the largest anthropogenic source of global CH_4_, having contributed approximately 97 Tg CH_4_/year, which is approximately 29.5% of total global CH_4_ emissions from 2000 to 2009 [3]. In addition to the GHG effect, CH_4_ emission results in a gross feed energy loss of approximately 2 to 12% to the ruminant host [4]. Globally, research efforts and funding have been invested to develop novel CH_4_ mitigation strategies including altering the dietary regimen, feed supplements, and the use of both organic and inorganic molecules such as halomethanes and ionophores [2]. However, these different mitigation strategies have had varying degrees of success when implemented, with some strategies accompanied by adverse effects on animal health and production and also on the environment [5], indicating their limited practical use on farms. Furthermore, some of these mitigation strategies, including some CH_4_ inhibitors, that were found to be active in in vitro studies were reported to be ineffective in vivo [6]. Although a few CH_4_ mitigation practices have been found to be effective in inhibiting methanogenesis in vivo [7–9], their impact on archaea and other microbes present in the foregut of ruminants is unknown.

Billions of microbes (bacteria, protozoa, fungi, and archaea) inhabit the rumen and work synergistically to digest what is indigestible feed for the host. Methane is a natural byproduct formed by methanogens (archaea) by utilizing hydrogen (H_2_), carbon dioxide (CO_2_), methanol, and methylamines that are released during carbohydrate and lipid breakdown by other microbes [10, 11]. Therefore, any mitigation strategy developed to reduce methanogenesis can perturb the symbiotic relationship between archaea and other microbial domains, which can negatively impact the rumen fermentation pattern and ultimately reduce feed intake and animal productivity. Strategies that result in depression of feed intakes are not practical for the US dairy and beef production system. Therefore, a greater understanding of the impact of CH_4_ mitigation strategies on the symbiotic relationship among microbial domains and their selective inhibition of microbial genes/enzymes that control CH_4_ production is critically needed before the inhibitors are recommended for on-farm use. However, such information is not available as our knowledge on the functional role of archaea and interactions between bacteria and archaea in the rumen is limited.

Recently, it was demonstrated that supplementing high-yielding dairy cows with 3-nitrooxypropanol (3-NOP; DSM Nutritional Products, CH-4303, Kaiseraugst, Switzerland), a potent CH_4_ inhibitor, led to a 27% and 57% reduction in enteric CH_4_ emissions in beef cattle [12, 13] and 23–37% in dairy cattle [14, 15]. Similarly, supplementation of 3-NOP to dairy cows over a 15-week experimental period reduced enteric CH_4_ emissions by 26 to 30% with no observable impact on feed intake and productivity [7, 16–18]. Further, these authors observed that H_2_ emissions increased under inhibited methanogenesis for the first 8 weeks and then steadily declined in 3-NOP supplemented cows over the remainder of the 15-week period [7]. In this and other studies, the increased H_2_ emissions could only partially be accounted for by the decreased CH_4_ emissions [12, 18–20]. Therefore, the fate of H_2_ under normal and inhibited methanogenesis remains to be determined, as well as whether there are unidentified alternative sinks that can capture H_2_ when it is spared from methanogens in the rumen.

The inhibitor 3-NOP was designed to inhibit methyl-coenzyme M reductase (MCR), a highly conserved enzyme family that is essential for methanogenesis and is found in all methanogenic archaea. Notably, a study by Duin et al. [21] revealed that 3-NOP inhibited the growth of methanogenic archaea but had varying effects on individual methanogenic lineages in vitro. However, the effect of 3-NOP on the rumen microbiome remains to be investigated. We hypothesized that 3-NOP would significantly reduce the methanogenic community composed of hydrogenotrophic methanogens (4H_2_ + CO_2_ → CH_4_) and methylotrophic methanogens (1H_2_ + methanol or methylamines → CH_4_). We also hypothesized that reduction of methanogenesis would induce changes in ruminal H_2_-forming microbial communities via altering H_2_ concentrations in the rumen of dairy cows. To test this hypothesis, we employed a combination of metagenomic (metaG) and metatranscriptomic (metaT) approaches to determine changes in microbial diversity and microbial gene expression in the rumen of dairy cows with and without 3-NOP supplementation to the feed.

## Results

### Changes in total and metabolically active methanogenic communities in response to 3-NOP supplementation

**Fig. 1:**
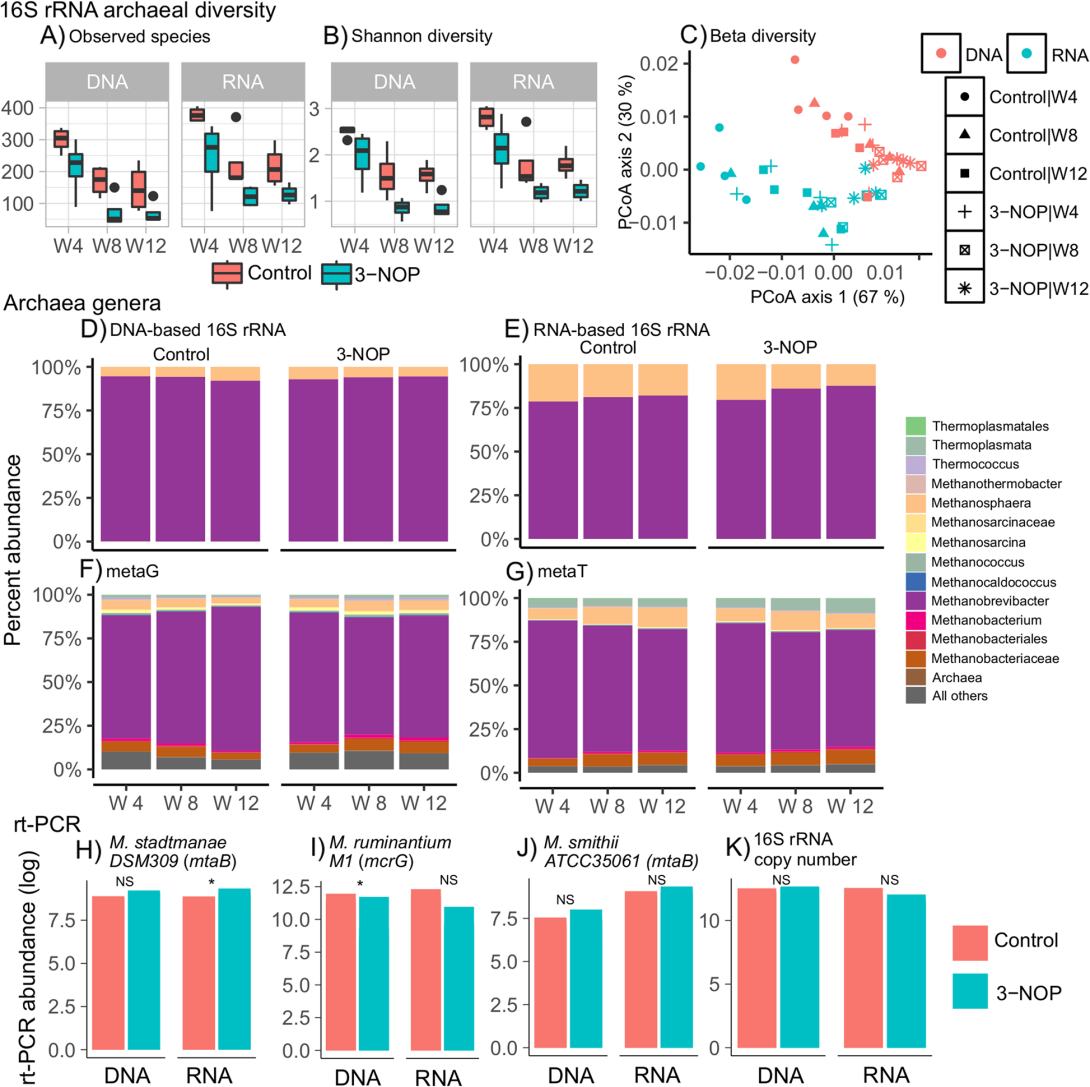
Rumen archaeal diversity and composition in control and 3-nitrooxypropanol (3-NOP) treated cows at weeks 4, 8, and 12. 16S rRNA archaeal diversity: **A** species richness, **B** Shannon diversity, and **C** comparison of overall community between samples by weighted UniFrac distances in DNA-based and RNA-based 16S rRNA analysis (beta diversity). Archaeal genera: comparison of archaeal composition at genus level for **D** DNA-based 16S rRNA, **E** RNA-based 16S rRNA, **F** metagenomics (metaG), and **G** metatranscriptomics (metaT). rt-PCR: quantification of selected methanogens at week 8 by real time PCR (rt-PCR) for **H***Methanosphaera stadtmanae DSM309* (*mtaB*), **I***Methanobrevibacter ruminantium M1* (*mcrG*), **J***Methanobrevibacter smithii ATCC35061* (*mtaB*), and **K** 16S rRNA copy number. PCoA, principal coordinates analysis. NS, no statistical significance in generalized linear mixed model (glmer); *P < 0.05; **P < 0.01; ***P < 0.001. The black circles appearing on the boxplots are the outlier samples

Rumen samples from 8 cows (4 control and 4 3-NOP supplemented cows) collected at weeks 4, 8, and 12 of a 15-week experiment were separated into solid and liquid fractions and analyzed for total (DNA) and metabolically active (RNA) methanogenic archaeal and bacterial diversity (solid communities; Fig. 1; liquid communities; SI Additional file 1: Figure S1). For beta diversity, the methanogenic community profiles differed by treatment (DNA solid: *P* = 0.001; RNA solid: *P* = 0.002; RNA liquid: *P* = 0.004) and week (DNA solid: *P* = 0.001; RNA solid: *P* = 0.001; DNA liquid: *P* = 0.052; RNA liquid: *P* = 0.014), but no interaction was observed between treatment and week in either DNA-based or RNA-based archaeal communities across either fraction (Fig. 1A; SI Additional file 1: Table 1). The solid fraction of rumen samples was further used for metagenomic and metatranscriptomic analysis to understand mechanisms of methanogenesis and to what extent microbial gene expression was altered when dairy cows were supplemented with 3-NOP.

**Table 1:** PERMANOVA analysis for 16S rRNA archaeal amplicon sequencing data. Treatment: control and 3-nitrooxypropanol (3-NOP); week: W4, W8, and W12. (.) *P* < 0.1; (*) *P* < 0.05; (**) *P* < 0.01; (***) *P* < 0.001; *NS*, not significant

**DNA solid**				**RNA solid**			
	*R*^2^	*P* value			*R*^2^	*P* value	
Treatment	0.219	0.001	***	Treatment	0.231	0.002	**
Week	0.254	0.001	***	Week	0.247	0.001	***
Treatment: Week	0.030	0.30	NS	Treatment: Week	0.021	0.36	NS
**DNA liquid**				**RNA liquid**			
Treatment	0.144	0.16	NS	Treatment	0.157	0.004	**
Week	0.187	0.05	. NS	Week	0.207	0.01	*
Treatment: Week	0.010	0.68	NS	Treatment: Week	0.010	0.66	NS
**Liquid vs solid in DNA**				**Liquid vs solid in RNA**			
Phase	0.152	0.004	**	Phase	0.237	0.001	***
**DNA vs RNA in solid**				**DNA vs RNA in liquid**			
Nucleic acid	0.202	0.001	***	Nucleic acid	0.182	0.001	***

Based on sequencing information (Supplementary information text), methanogenic archaea constituted about 6% and 8% of metagenomes and metatranscriptomes, respectively (Supplementary information text, SI Additional file 1: Table S1, Table S2). Across all samples, within methanogenic archaea from metagenomic data, we found that the genus *Methanobrevibacter* alone contributed to 66–82% of total ruminal archaeal abundance followed by unclassified genera of *Methanobacteriaceae* and *Methanosphaera* at 3–6 and 3–5%, respectively (Fig. 1F; Table 2; SI Additional file 1: Table S3). In cows that received 3-NOP supplementation, the relative abundance of *Methanobrevibacter* was lower while that of unclassified *Methanobacteriaceae* and *Methanosphaera* was higher (*P* < 0.05) at weeks 4 and 8 when compared with control cows (SI Additional file 1: Table 2; Table S3). From metatranscriptomic data (Table 2; SI Additional file 1: Table S4), the relative abundance of *Methanosphaera* and genera from Thermoplasmata was nearly doubled compared with metagenomics. The relative abundance of *Methanobrevibacter* was reduced (*P* < 0.001) with 3-NOP supplementation at weeks 4, 8, and 12 whereas that of *Methanosphaera* was higher (*P* < 0.001) at week 4 and week 8 but reduced at week 12. Based on rt-PCR results (Fig. 1H–K), it was evident that 3-NOP significantly reduced *Methanobrevibacter ruminantium* (*P* = 0.03) but had no effect on *Methanobrevibacter smithii* or 16S rRNA gene copy number in either DNA or RNA-based analysis. *Methanosphaera stadtmanae* was numerically increased with 3-NOP supplementation compared with control samples.

**Table 2:** The most abundant (> 1%) archaeal and bacterial genera identified in metagenomics and metatranscriptomics data in cows treated with 3-nitrooxypropanol (3-NOP) compared to control cows at weeks 4, 8, and 12 weeks

Genus	Control				3-NOP				Significance				
	W4	W8	W12	SEM	W4	W8	W12	SEM	Trt	W4 vs W8	W4 vs W12	Trt:W4 vs W8	Trt: Trt:W4 vs W12
**Archaea-metagenomics**													
*Methanobrevibacter*	82.36	75.27	70.38	2.28	73.89	66.96	69.74	2.39	0.02	< 0.001	< 0.001	0.55	< 0.001
*Methanobacteriaceae*	3.76	5.84	5.52	0.33	4.35	6.61	6.39	0.35	0.02	< 0.001	< 0.001	0.74	0.34
*Methanosphaera*	3.17	4.81	5.26	0.48	4.41	5.63	5.31	0.46	0.13	< 0.001	< 0.001	0.006	< 0.001
*Methanobacterium*	0.87	1.39	1.38	0.11	1.21	1.84	1.73	0.13	0.03	< 0.001	< 0.001	0.82	0.27
*Methanosarcina*	0.9	1.11	1.58	0.16	1.54	1.65	1.44	0.19	0.02	< 0.001	< 0.001	0.65	< 0.001
*Thermoplasmata*	0.79	1.04	1.3	0.17	1.01	1.59	1.56	0.16	0.01	< 0.001	< 0.001	0.82	< 0.001
**Archaea-metatranscriptomics**													
*Methanobrevibacter*	78.6	72.19	69.46	1.66	74.01	67.18	66.82	1.84	0.12	< 0.001	< 0.001	< 0.001	< 0.001
*Methanosphaera*	6.14	9.65	11.07	0.88	7.32	10.68	7.92	0.81	0.76	< 0.001	< 0.001	0.002	< 0.001
*Methanobacteriaceae*	4.24	6.98	6.86	0.46	6.47	7.24	8.07	0.45	< 0.001	< 0.001	< 0.001	< 0.001	< 0.001
*Thermoplasmata*	4.83	4.14	4.51	0.39	5.13	6.82	8.03	0.8	0.17	< 0.001	< 0.001	< 0.001	< 0.001
**Bacteria- metagenomics**													
*Bacteroidetes Prevotella*	16.19	16.14	18.56	0.826	17.54	16.52	14.66	0.81	0.24	< 0.001	< 0.001	< 0.001	< 0.001
*Bacteria Unclassified*	4.29	4.45	4.32	0.094	4.17	4.41	4.57	0.092	0.40	< 0.001	0.004	< 0.001	< 0.001
*Fibrobacteres Fibrobacter*	3.16	3.07	3.27	0.346	3.32	2.41	3.64	0.309	0.97	< 0.001	0.36	< 0.001	< 0.001
*Firmicutes Butyrivibrio*	3.6	3.33	2.72	0.332	2.72	2.85	3.18	0.209	0.006	< 0.001	< 0.001	< 0.001	< 0.001
*Bacteroidetes Bacteroides*	2.55	2.52	2.75	0.092	3	2.75	2.54	0.094	0.002	0.66	< 0.001	< 0.001	< 0.001
*Firmicutes Lachnospiraceae*	2.06	2.13	1.94	0.068	1.97	2.02	2.07	0.03	0.007	< 0.001	< 0.001	0.42	< 0.001
*Firmicutes Clostridium*	1.86	1.98	1.87	0.041	1.74	2.03	1.98	0.061	0.02	< 0.001	0.38	< 0.001	< 0.001
*Actinobacteria Bifidobacterium*	2.24	2.13	1.45	0.471	2.1	1.93	1.4	0.323	0.82	< 0.001	< 0.001	0.001	< 0.001
*Firmicutes Lachnoclostridium*	1.51	1.64	1.45	0.052	1.49	1.56	1.59	0.026	0.10	< 0.001	< 0.001	0.002	< 0.001
*Firmicutes Clostridia*	1.38	1.47	1.33	0.045	1.27	1.42	1.5	0.053	0.19	< 0.001	< 0.001	< 0.001	< 0.001
*Firmicutes Ruminococcus*	1.38	1.35	1.29	0.071	1.05	1.31	1.27	0.07	< 0.001	< 0.001	< 0.001	< 0.001	< 0.001
*Actinobacteria Olsenella*	1.1	1.16	1.4	0.137	1.25	1.15	1.16	0.114	0.38	< 0.001	< 0.001	< 0.001	< 0.001
*Firmicutes Clostridiales*	1.14	1.16	1.07	0.036	1.1	1.13	1.2	0.044	0.41	0.09	< 0.001	< 0.001	< 0.001
*Bacteroidetes Prevotellaceae*	0.89	0.96	1.11	0.069	1.11	1.05	0.88	0.059	0.01	< 0.001	< 0.001	< 0.001	< 0.001
**Bacteria-metatranscriptomics**													
*Bacteroidetes Prevotella*	17.65	16.96	18.74	0.749	16.1	16.42	14.9	0.559	0.26	< 0.001	< 0.001	< 0.001	< 0.001
*Firmicutes Lachnospiraceae*	3.81	4.16	3.9	0.144	4.39	4.05	4.62	0.15	0.20	< 0.001	< 0.001	< 0.001	< 0.001
*Bacteria*	3.74	3.68	3.61	0.07	3.63	3.58	3.77	0.056	0.71	< 0.001	0.56	0.001	< 0.001
*Fibrobacteres Fibrobacter*	3.77	3.65	3.92	0.425	3.15	2.8	3.22	0.269	0.21	< 0.001	< 0.001	< 0.001	< 0.001
*Firmicutes Ruminococcus*	3.47	3.71	3.05	0.218	2.59	2.83	2.86	0.145	0.16	< 0.001	< 0.001	< 0.001	< 0.001
*Firmicutes Butyrivibrio*	3.17	2.98	2.77	0.218	3	2.95	2.9	0.195	0.52	0.002	< 0.001	< 0.001	< 0.001
*Firmicutes Lachnoclostridium*	1.97	2.13	1.99	0.068	2.28	2.05	2.53	0.1	0.005	< 0.001	< 0.001	< 0.001	< 0.001
*Bacteroidetes Bacteroides*	2.01	1.87	1.96	0.074	2.04	1.92	1.74	0.069	0.98	< 0.001	< 0.001	< 0.001	< 0.001
*Firmicutes Clostridium*	1.24	1.47	1.71	0.199	2.5	1.56	1.96	0.259	< 0.001	< 0.001	< 0.001	< 0.001	< 0.001
*Firmicutes Eubacterium*	1.51	1.72	1.6	0.073	2.06	1.68	1.71	0.111	0.10	< 0.001	< 0.001	< 0.001	< 0.001
*Firmicutes Clostridia*	1.54	1.54	1.41	0.065	1.61	1.49	1.79	0.075	0.51	< 0.001	0.65	< 0.001	< 0.001
*Firmicutes Clostridiales*	1.34	1.36	1.28	0.05	1.4	1.36	1.6	0.06	0.42	< 0.001	< 0.001	< 0.001	< 0.001
*Actinobacteria Olsenella*	1.12	1.15	1.49	0.116	0.94	1.27	1.53	0.129	0.34	< 0.001	< 0.001	< 0.001	< 0.001
*Firmicutes Faecalibacterium*	1.24	1.24	1.16	0.036	1.16	1.21	1.38	0.04	0.92	< 0.001	< 0.001	< 0.001	< 0.001
*Actinobacteria Bifidobacterium*	1.34	1.57	0.77	0.407	1.72	0.78	1.11	0.17	0.45	< 0.001	< 0.001	< 0.001	< 0.001
*Proteobacteria Proteobacteria*	1.3	1.2	1.19	0.045	1.1	1.21	1.1	0.056	0.003	< 0.001	< 0.001	< 0.001	< 0.001
*Bacteroidetes Prevotellaceae*	1.12	1.24	1.4	0.11	1.12	1.17	1.04	0.06	0.65	< 0.001	< 0.001	< 0.001	< 0.001
*Firmicutes Megasphaera*	0.27	0.3	1.08	0.29	0.23	3.83	0.3	1.302	0.92	< 0.001	< 0.001	< 0.001	< 0.001

### Impact of 3-NOP on methanogenesis pathways

**Fig. 2:**
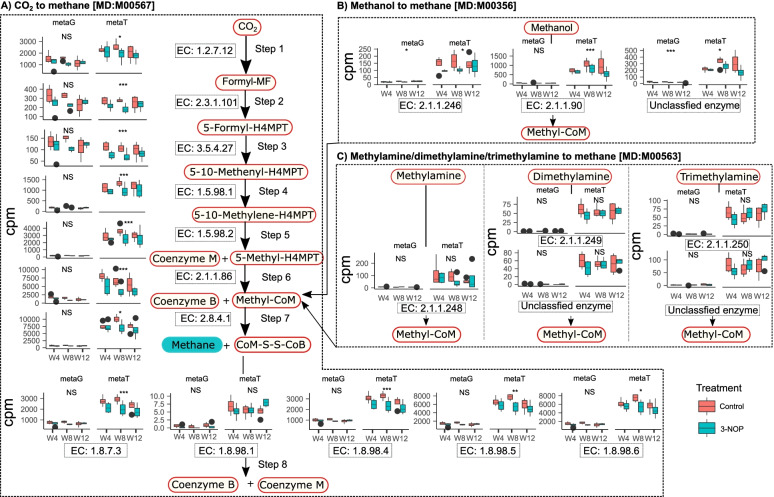
Comparisons of genes (metagenomics; metaG) and transcripts (metatranscriptomics; metaG) abundance in copies per million (cpm) for enzymes involved in methanogenesis between control and 3-nitrooxypropanol (3-NOP)-treated cows at weeks 4, 8, and 12. **A** Carbon dioxide (CO_2_)/hydrogen (H_2_) methanogenesis pathway (KEGG pathway entry MD:M00567), **B** methanol methanogenesis pathway (KEGG pathway entry MD:M00356), and **C** methylamine methanogenesis pathway (KEGG pathway entry MD:M00563). NS, no statistical significance in generalized linear mixed model (glmer); *P < 0.05; **P < 0.01; ***P < 0.001. The black circles appearing on the boxplots are the outlier samples

Using metagenomic and metatranscriptomic data, we quantified the genes and transcripts of enzymes involved in the 3 predominant ruminal methanogenesis pathways (CO_2_-, methanol-, and methylamine-reducing pathways) in cows with and without 3-NOP supplementation at weeks 4, 8, and 12 of the experimental period (Fig. 2). In addition, taxonomy of the annotated genes and transcripts was also tracked to help us understand the role of individual methanogenic lineages in methanogenesis.

### CO_2_-reducing methanogenesis pathway

The CO_2_-reducing pathway is presented as the central pathway represented by steps 1–8 (Fig. 2A). Steps 1–5 are unique to this pathway, and steps 6–8 are common to all methanogenic pathways. Genes coding for the corresponding enzymes involved in steps 1–5 were identified using metagenomics, and gene expression was compared by identifying transcripts using metatranscriptomics (Fig. 2A). In this study, we were not able to identify genes associated with EC: 1.17.1.9, an enzyme required to convert formate to CO_2_ and therefore not shown in Fig. 2A. Only a small number of transcripts were identified for EC: 1.17.1.9, suggesting that in this particular experiment, formate was not a major substrate for methanogenesis. The copy number for genes encoding for enzyme EC: 1.2.7.12 (step 1 in Fig. 2A), required for reduction of CO_2_ to formylmethanofuran, was highest compared with all other enzymes involved in steps 1–5. The transcript copy number for EC: 1.2.7.12 was higher compared with transcripts of enzymes involved in steps 2, 3, and 4 but lower than those in step 5. The number of gene copies for enzyme EC: 1.2.7.12 tended to be lower (*P* = 0.115), but the corresponding transcripts were lower (*P* = 0.05) in 3-NOP supplemented cows compared with control cows. The transcript copy numbers for EC: 2.3.1.101 (*P* < 0.001; step 2), 3.5.4.27 (*P* < 0.001; step 3), 1.5.98.1 (*P* < 0.001; step 4), and 1.5.98.2 (*P* < 0.001; step 5) were substantially reduced in 3-NOP supplemented cows compared with control cows.

### Methanogens involved in the CO_2_-reducing pathway and impact of 3-NOP

The most abundant archaea that contributed to the CO_2_-reducing pathway were identified as *Methanobrevibacter* species in metagenomic data (SI Additional file 1: Table S5). Six species of *Methanobrevibacter* (*M. ruminantium M1*, *M. olleyae YLM1*, *M. millerae SM9*, *M. sp. YE315*, *M. sp AbM4*, and *M. smithii ATCC 35061*) were identified. These 6 archaea species contributed to more than 93% of the genes as well as transcripts coding for enzymes involved in steps 1–5 of the CO_2_-reducing pathway. The most abundant was *M. ruminantium M1* followed by the other 5 *Methanobrevibacter* species with small variations in their contributions towards gene and transcript abundance.

### Impact of 3-NOP on methanol- and methylamine-utilizing pathways

In the methanol-utilizing pathway (Fig. 2B), the genes and transcripts for the enzyme methanol-corrinoid protein co-methyltransferase (*mtaB*; EC: 2.1.1.90) were found in greater numbers compared with the other 2 enzymes involved in this pathway. Gene copies were numerically reduced with 3-NOP supplementation at weeks 4 and 8 but not at week 12. However, transcripts for this enzyme were reduced (*P* = 0.001) at weeks 4, 8, and 12 in cows supplemented with 3-NOP with the greatest reduction at week 12. These data indicate that although changes in gene copies were inconsistent, their expression was reduced at all sampling times in 3-NOP supplemented dairy cows.

The archaea populations that contributed genes/transcripts to the methanol-utilizing pathway were identified as *Methanosphaera BMS*, *Methanosphaera stadtmanae*, *Methanogenic archaeon ISO4-H5*, and *Methanobrevibacter smithii* (SI Additional file 1: Table S6). Metagenomic results showed that these 4 archaea species comprised about 97–99% of identified methanol-utilizing archaea with 46–50% of gene copies from *M. stadtmanae*, 39–44% from *M. BMS*, 5–9% from *M. smithii*, and 2–4% from *M. archaeon.* In metatranscriptomics, *M. BMS* had the greatest metabolic activity with nearly 56% of contribution followed by *M. stadtmanae* at 38% and *M. archaeon* at 5%, indicating their significant role in utilizing methanol. Although *M. smithii* had considerable gene copies for methanol utilization, there was no expression found for these genes indicating that *M. smithii* is not capable of utilizing methanol.

In the methylamine-utilizing pathway (Fig. 2C), most of the genes coding for enzymes involved in transfer of methylamines were found in greater numbers relative to dimethyl or trimethylamines, although the total gene/transcript copies were much lower than those involved in other pathways. Across all animals, copies of the gene coding for the enzyme methylamine-corrinoid protein co-methyltransferase (EC: 2.1.1.248) were negligible (<10 copies per million [cpm]) whereas the corresponding transcript contribution was 10 times greater than its gene abundance. No differences were noted between treatment groups in either their genes or transcripts. The archaea populations that contributed to the methylamine pathway were *Methanogenic archaeon ISO4* and *Thermoplasmatales archaeon BRNA1* with some contribution from *Candidatus Methanomethylophilus alvus Mx1201* (SI Additional file 1: Table S7).

### Impact of 3-NOP on methyl-coenzyme M reductase, the connecting point for all methanogenesis pathways

The enzyme MCR (EC: 2.8.4.1) is responsible for CH_4_ formation by incorporating methyl Co-M and Co-B to form a heterodisulphide and releasing CH_4_ in the penultimate step [22]. In the current study, the copy number of genes and transcripts for EC: 2.8.4.1 were among the most abundant genes/transcripts involved in methanogenesis pathways. Although variable responses were noted for gene copies, transcripts tended to be reduced (*P* = 0.062) in 3-NOP supplemented dairy cows indicating that the reduction in CH_4_ emissions by 3-NOP as described [18] is accompanied by a reduction in expression of genes coding for MCR (Fig. 2A).

The enzyme MCR has 3 subunits (alpha, beta, and gamma) encoded by K00399, K00401, and K00402, respectively [23]. Using metagenomics and metatranscriptomics, across all samples, we found that gene and transcript copy numbers for K00399 and K00402 were similar to each other whereas K00401 was numerically lower compared with K00399 and K00402 (Table 3). With 3-NOP supplementation, gene copies for MCR tended to be lower with a 23% and 26% reduction observed at weeks 4 and 8 and a 15% increase at week 12. The transcripts of the MCR enzyme also tended to be lower (*P* = 0.062) with a 4%, 30%, and 14% reduction in 3-NOP supplemented cows at weeks 4, 8, and 12, respectively (Table 3) compared with control cows. Notably, the reduction in transcript copies was greater for the beta chain (K00401; *P* < 0.001) in response to 3-NOP, followed by the alpha and then gamma subunits, indicating that the beta subunit may be more vulnerable to inhibition by 3-NOP. The effect of 3-NOP was greatest at week 8, which may be attributed to an increase in dry matter intakes during that period [18] which may have increased the allowance of 3-NOP in the rumen compared with weeks 4 and 12 [24]. In addition, we identified the three genes (*mcr*𝛼, *mcrB*, *mcr*𝛾) from the methanogen MAGs across all samples. Although the percent reduction varied between raw reads and methanogen MAGs between treatments at each sampling week, overall differences in MCR enzyme followed the same trend for raw reads as well as MAGs (Table 3). Other genes and transcripts involved in step 8 had very low copy number and are discussed in the Supplementary information text and Table S8 (SI Additional file 1).

**Table 3:** Changes in genes and transcripts (copies per million, CPM) encoding for methyl-coenzyme M reductase (MCR) enzyme (EC: 2.8.4.1) in the rumen of dairy cows supplemented with 3-nitrooxypropanol (3-NOP) compared to control cows at weeks 4, 8, and 12. Based on raw reads and based on metagenome assembled genomes (MAGs). K00399, alpha subunit of MCR; K00401, beta subunit of MCR; K00402, gamma subunit of MCR

MCR genes identification based on raw reads										
	Metagenomics									
	W4			W8			W12			
	Control	3-NOP	% reduction	Control	3-NOP	% reduction	Control	3-NOP	% reduction	Treatment ***P*** value
**K00399**	265	213	20	306	230	25	227	253	−11	0.18
**K00401**	245	178	27	239	163	32	169	192	−14	0.18
**K00402**	253	197	22	293	223	24	210	251	−20	0.22
**2.8.4.1**	763	588	23	838	616	26	606	697	−15	0.12
**Metatranscriptomics**										
**K00399**	2756	2578	6	3656	2548	30	2667	2333	13	0.15
**K00401**	2135	1781	17	2529	1606	36	1885	1415	25	0.001
**K00402**	2679	2908	−9	3946	2905	26	2829	2571	9	0.64
**EC:2.8.4.1**	7570	7268	4	10131	7059	30	7381	6320	14	0.06
**MCR gene identification based on MAGs**										
**Metagenomics**										
**K00399**	32	33	−3	49	47	5	36	51	−42	0.47
**K00401**	52	43	17	55	37	33	38	42	−11	0.10
**K00402**	142	92	35	88	41	54	69	54	22	0.06
**EC:2.8.4.1**	75	56	26	64	42	35	48	49	−3	0.07
**Metatranscriptomics**										
**K00399**	411	507	−24	777	607	22	596	591	1	0.92
**K00401**	537	565	−5	877	570	35	681	545	20	0.08
**K00402**	1282	988	23	1276	816	36	891	605	32	0.02
**EC:2.8.4.1**	743	687	8	977	664	32	723	580	20	0.01

$$\mathrm{Percent}\ \mathrm{reduction}=\frac{\left(\mathrm{Control}\right)-\left(3-\mathrm{NOP}\right)}{\left(\mathrm{Control}\right)}\ \mathrm{X}\ 100$$

### Impact of 3-NOP on bacterial populations

**Fig. 3:**
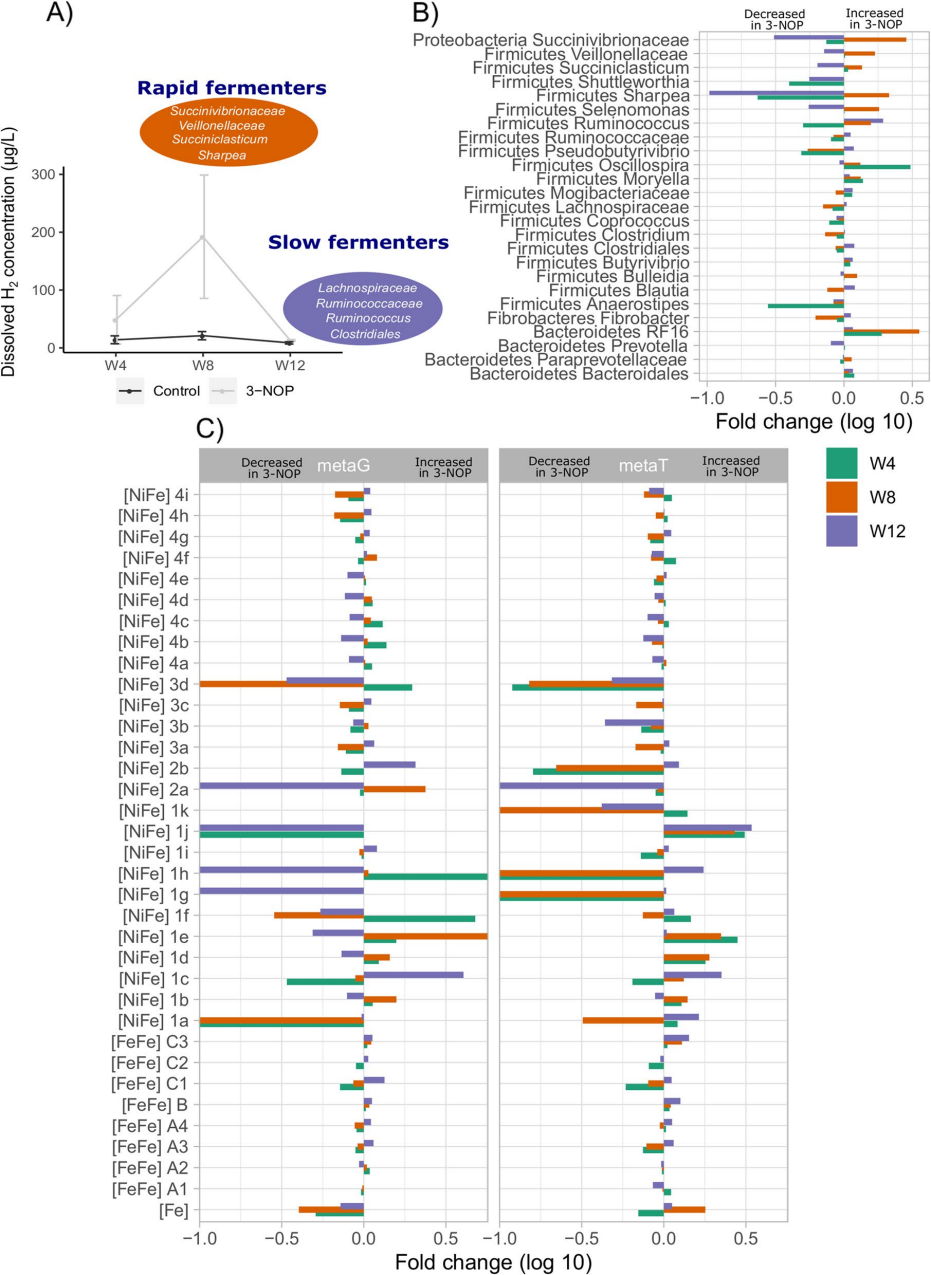
Effect of 3-nitrooxypropanol (3-NOP) on rumen bacteria via changes in dissolved hydrogen (H_2_) concentrations. **A** Effect of 3-NOP on dissolved H_2_ in lactating dairy cows; the bacterial genera appearing in the oval shapes are rapid (orange) and slow (blue) fermenters that were increased in 3-NOP treated cows at week 8 and week 12, respectively (see panel **B**). **B** Fold change (log 10) between control and 3-NOP-treated cows at weeks 4, 8, and 12 in the relative abundance of selected bacterial genera in DNA-based 16S rRNA analysis. The selection of bacterial genera was based on significant differences (glmer test) between treatment groups (*P* < 0.05). **C** Fold change (log 10) between control and 3-NOP treated cows at weeks 4, 8, and 12 in hydrogenase content in metagenomes (metaG) and metatranscriptomes (metaT). Hydrogenase content is shown based on hydrogenase subgroup. These are divided into fermentative hydrogenases (H_2_-producing; group A1, A2, B [FeFe]-hydrogenases), bifurcating hydrogenases (bidirectional; group A3, A4 [FeFe]-hydrogenases), respiratory hydrogenases (H_2_-uptake; group 1a, 1b, 1c, 1d, 1e, 1f, 1g, 1h, 1i, 1j [NiFe]-hydrogenases), respiratory hydrogenases (H_2_-evolving; group 4b, 4d [NiFe]-hydrogenases), alternative and sensory hydrogenases (H_2_-uptake; 2a, 2b [NiFe]-hydrogenases), cofactor-coupled bidirectional hydrogenases (3b, 3d, [NiFe]-hydrogenases), methanogenic hydrogenases (H_2_-uptake; group 1k, 3a, 3c, 4h, 4i [NiFe]-hydrogenases, [Fe]-hydrogenases), energy-converting hydrogenases (bidirectional; group 4a, 4c, 4e, 4f, 4g [NiFe]-hydrogenases), and sensory hydrogenases (group C [FeFe]-hydrogenases). Positive and negative log 10-fold change is the increased and decreased relative abundance, respectively, in 3-NOP compared with controls cows.

In DNA-based 16S rRNA sequencing and metagenomics, Firmicutes and Bacteroidetes were the most dominant bacterial phyla (Table 2; SI Additional file 1: Table S9; Table S11). However, in RNA-based 16S rRNA sequencing and metatranscriptomics (Table 2; SI Additional file 1: Table S10; Table S12), the contribution of Firmicutes was much higher and that of Bacteroidetes was much lower than their corresponding gene copies. Further, the relative abundance of Fibrobacteres and Proteobacteria was much higher in RNA compared with DNA in 16S rRNA sequencing. Firmicutes was dominated by *Lachnospiraceae, Ruminococcaceae*, *Clostridiales,* and *Butyrivibrio*, Bacteroidetes was dominated by *Prevotella*, *Bacteroidales*, and *S24-7*, and Proteobacteria was dominated by *Succinivibrionaceae*. Bacterial lineages that showed significant differences (generalized linear mixed model [glmer] test; *P* < 0.05) between control and 3-NOP groups were selected to indicate changes in the relative abundance between weeks (Fig. 3A, B). In cows that received 3-NOP, *Lachnospiraceae*, *Ruminococcaceae*, and *Clostridiales* were reduced at weeks 4 and 8 but increased by week 12, whereas *Succinivibrionaceae*, *Succiniclasticum*, *Veillonellaceae*, and *Sharpea* showed the opposite trend compared with control cows.

### Hydrogenases regulating H_2_ production under normal and inhibited methanogenesis

Under inhibited methanogenesis, concentrations of both gaseous and dissolved H_2_ increased from week 4 through week 8 and then declined from week 9 through week 12 as described in Melgar et al. ([18] Fig. 3A; SI Additional file 1: Table S13). The dynamics in H_2_ concentrations in response to 3-NOP supplementation were also associated with changes in hydrogenases, which are metalloenzymes that interchangeably convert H_2_ to 2[H] + 2e-. Hydrogenases are broadly classified as [FeFe], which aid in sensing H_2_ concentrations and production of H_2_; [NiFe], which facilitate H_2_ uptake; and [Fe], of which the function is currently unknown. The [FeFe] are further differentiated into A1-A4, B, and C1-C3 with the first two groups regulating H_2_ production and the latter sensing H_2_ concentrations. Both metaG and metaT data revealed that [FeFe]A1, [FeFe]A3, [FeFe]B, [FeFe]C2, and [FeFe]C3 constituted the majority of the [FeFe] hydrogenases (Fig. 3C).

Interestingly, the gene copies encoding for hydrogenases A1, A3, B, C1, and C3 were fairly consistent between weeks 4, 8, and 12 in the control group. However, variations were noted in hydrogenases between sampling weeks within 3-NOP supplemented cows, with A3, B, and C3 progressively increasing from week 4 through week 12 (SI Additional file 1: Table S14, Table S15). When compared with control, the gene copies for B (*P* = 0.007) and C3 (*P* < 0.001) were higher in 3-NOP supplemented cows at all sampling weeks. Both A1 and A3 had a greater number of transcripts, as cpm, compared with gene copies, whereas transcripts of genes encoding for B, C2, and C3 were only slightly greater than their corresponding gene copies. The ratio of A1:A3 was consistent at weeks 4 and 8 whereas A3 was numerically reduced and A1 numerically increased by week 12 in the control group. In the 3-NOP supplemented group, transcripts of genes coding for A1 were numerically reduced from week 4 through week 12 whereas the opposite pattern was observed for genes coding for A3. Transcripts of genes coding for A3 showed a trend (*P* = 0.11) to be lower at weeks 4 and 8 but increased by week 12 in 3-NOP compared with control samples.

### Alternative H_2_ sinks under inhibited methanogenesis

**Fig. 4:**
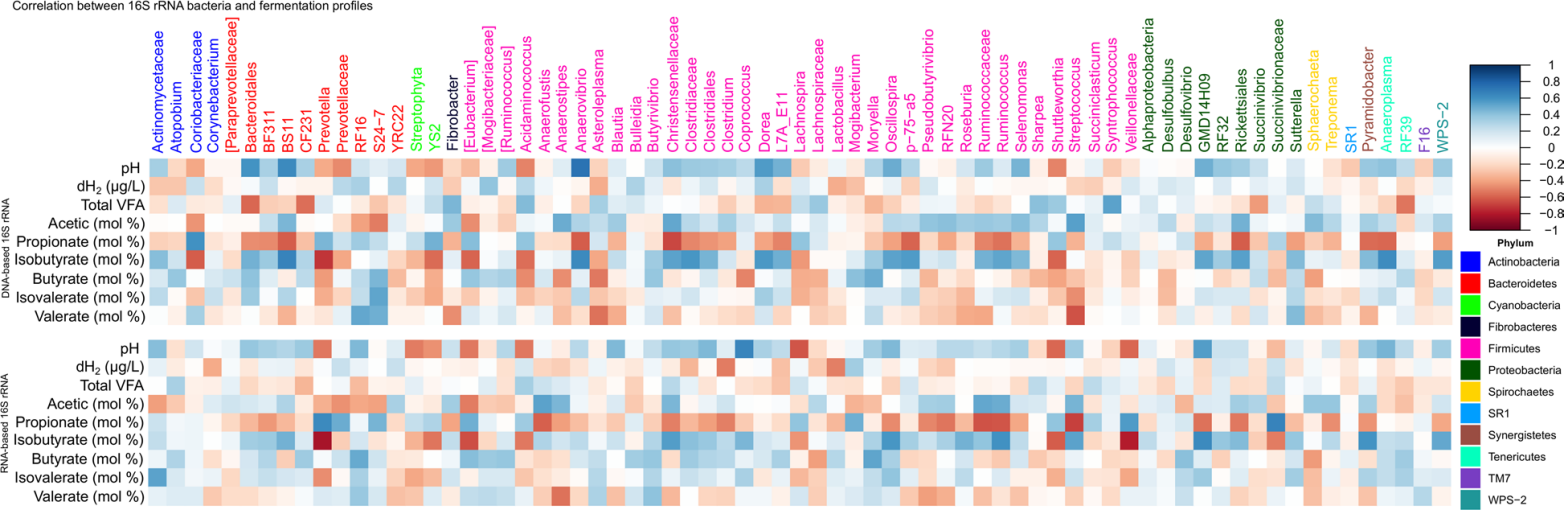
Associations between rumen bacteria and fermentation profiles. Correlation between bacteria (DNA-based [top] and RNA-based 16S rRNA sequencing analysis [bottom]) and fermentation profiles. Different colors of the bacterial genera show the corresponding phylum. dH_2_, dissolved hydrogen; VFA, volatile fatty acids; mol%, molar proportion

We attempted to investigate whether H_2_ spared under inhibited methanogenesis was directed to alternate H_2_ sinks (SI Additional file 1: Table S16; Table S17) that may directly or indirectly compete with methanogens. We identified that methanogenesis is the largest H_2_ sink followed by reduction of CO_2_ to acetate, reduction of fumarate to succinate, reduction of nitrate/nitrite to ammonia, and reduction of sulfate to H_2_S. Lastly, there is a very small contribution from sulfite reductase, fumarate reductase, and ammonia-forming nitrite reductase. Although acetyl-CoA synthetase, a marker gene for acetogenesis, was not significantly increased in either gene or transcripts copies, we found a significant increase (*P* = 0.008) in transcript copies of formyl-tetrahydrofolate synthetase, another marker gene for acetogens, in 3-NOP supplemented cows compared to control at all sampling weeks (SI Additional file 1: Table S16, Table S17). Methane inhibition did not result in a sudden increase of either genes or transcripts of other alternative H_2_ sinks that were investigated in this study (SI Additional file 1: Table S16, Table S17). Because there were differences in fermentation variables including volatile fatty acids (VFA; Table S18), we sought to investigate differences in fermentation pathways leading to VFA production (Fig. 4).

First, we performed correlation analysis between molar proportions of individual VFA and bacteria populations identified from DNA- and RNA-based 16S rRNA sequencing across all samples (Fig. 4). Associations between bacteria and individual VFA were more evident with RNA-based analysis than those identified using DNA-based methods. Bacteria that showed differences (*P* < 0.05) between control and 3-NOP treatments across all sampling weeks were selected to perform correlation analysis with fermentation parameters. We found that most of the lineages from Firmicutes were positively associated with acetate proportions and a few genera, such as *Prevotella* and *Succinivibrionaceae*, were positively associated with propionate.

**Fig. 5:**
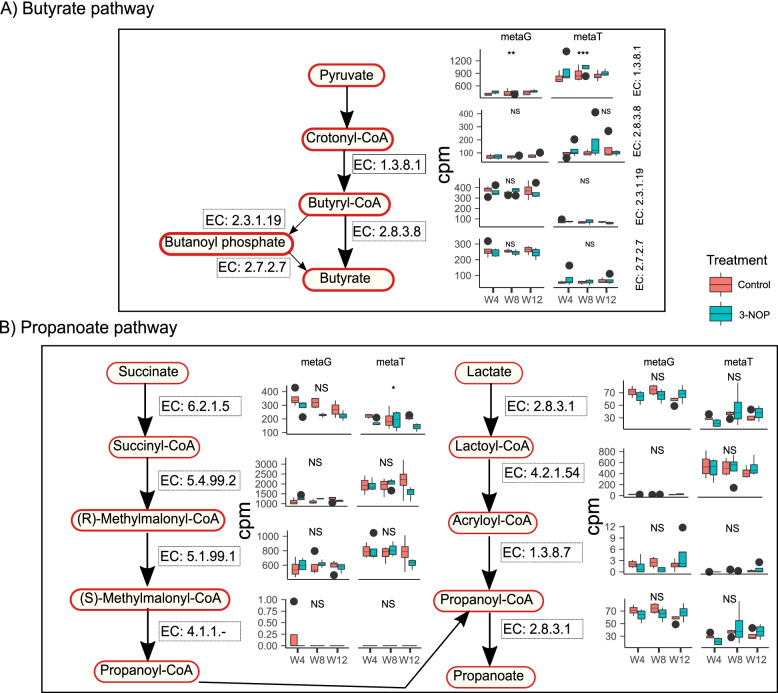
Comparisons of metagenomic (metaG) and metatranscriptomic (metaG) abundance for enzymes involved in the butyrate pathway (**A**) and propanoate pathway (**B**) between control and 3-nitrooxypropanol (3-NOP) treated cows at weeks 4, 8, and 12. cpm, copies per million; NS, no statistical significance in generalized linear mixed model (glmer); *P < 0.05; **P < 0.01; ***P < 0.001. The black circles appearing on the boxplots are the outlier samples

Next, we identified genes and transcripts coding for enzymes involved in different pathways leading to butyrate and propionate formation (Fig. 5A, B). There were differences in gene and transcript copies between control and 3-NOP treatment groups for enzymes involved in the butyrate pathway. Notably, genes and transcripts for the enzyme EC: 1.3.8.1, which catalyzes conversion of crotonyl-CoA to butyryl-CoA, were increased (genes: *P* = 0.001; transcripts: *P* < 0.001) in 3-NOP samples compared with control at all weeks of sampling. These data agree with increased (*P* < 0.05) molar proportions of butyrate in 3-NOP samples compared with control. Bacteria shown to be associated with the butyrate pathway are shown in Table S19 and Table S20 (SI Additional file 1).

The pathways leading to propionate were identified as M00741 (propanoyl-CoA via succinyl-CoA), M00013 (malonate semialdehyde pathway), the propanoate pathway, and the acrylate pathway via lactate (Fig. 5B). No differences were detected in genes involved in propionate pathways between control and 3-NOP group-treated cows. The M00741 pathway appeared to be the predominant pathway. Although there were small changes in transcripts, there was no particular pattern in response to 3-NOP treatment. Similarly, no differences were noted in the molar proportion of ruminal propionate. Bacteria shown to be associated with the propionate pathway are shown in Table S21 and Table S22 (SI Additional file 1). The bacterium that had the predominant role in the propionate pathway was identified as *Prevotella ruminicola* followed by several other bacteria.

## Discussion

Enteric CH_4_ formation is an intractable problem and is a consequence of complex microbial interactions in the forestomach of ruminant animals. Using 3-NOP, a potent CH_4_ inhibitor, as a feed supplement to dairy cow diets has been previously shown to reduce CH_4_ formation by 26 to 30% [7, 18] and by 23–37% [14, 15]. This study enabled us to gain a deeper understanding of the complex interdependencies between methanogens and bacteria for H_2_ under normal and inhibited methanogenesis in the rumen of dairy cows supplemented with 3-NOP. Using a combination of omic approaches, this study provides new information on temporal dynamics in individual methanogenic lineages and their contribute to total methanogenesis. Further, this study has elucidated both temporal changes in bacteria populations in response to fluctuating H_2_ concentrations in the rumen and some of the possible mechanisms by which the spared H_2_ under inhibited methanogenesis by 3-NOP was directed to alternate sinks.

Methanogenesis in the rumen can occur via hydrogenotrophic, methylotrophic, and aceticlastic pathways [25, 26], although the contribution of these pathways to total CH_4_ formation has not been clearly described. In the current study, using both metagenomic and metatranscriptomic approaches, we have identified that the CO_2_-reducing (hydrogenotrophic) pathway was predominant followed by the methanol-utilizing pathway and the methylamine-utilizing pathway regardless of treatment. However, metatranscriptomics predicts that the methylotrophic methanogenic (methanol- and methylamine-reducing) pathways may have greater contributions to total methanogenesis compared with their corresponding gene content, revealing that methylotrophic methanogens may have a greater contribution to CH_4_ formation in the rumen than what was originally anticipated. For example, we found that the total number of gene copies for the methanol-utilizing enzyme methanol--corrinoid protein co-methyltransferase (EC: 2.1.1.90) in *Methanosphaera* was < 100 cpm in metagenomic data, whereas it was > 600 cpm in metatranscriptomics (Fig. 2B), revealing that *Methanosphaera* has greater metabolic activity than what we would anticipate from the total number of gene copies alone. We also found that 2 lineages of *Methanomassiliicoccales*, *Methanogenic archaeon ISO4-H5*, and *Thermoplasmatales archaeon BRNA1*, which contributed to the methylamine-reducing pathway, also had a greater contribution from transcripts than their corresponding genes. These findings agree with those of Söllinger et al. [27] who employed metatranscriptomics to report that methylotrophic methanogens may have a greater role in methanogenesis than was originally thought.

Several studies have reported negative correlations between *Methanobrevibacter* and *Methanosphaera* lineages in the rumen [28, 29]. Because it was assumed that *Methanobrevibacter* is the predominant methanogenic genus and the contribution of *Methanosphaera* lineages is small, the competition among methanogens has been largely ignored until metatranscriptomic approaches were employed [27, 30] to understand methanogenesis. Differences in metabolic capabilities in hydrogenotrophic and methylotrophic methanogens have been discussed in our recent paper [31]. Although both *Methanobrevibacter* and *Methanosphaera* belong to the same order, *Methanobacteriales*, there are contrasting features between the 2 methanogenic genera that have functional relevance to CH_4_ formation and rumen fermentation. First, *Methanobrevibacter* lineages reduce CO_2_ or formate whereas *Methanosphaera* has acquired the ability to extract the methyl group from methanol and therefore adopts a methanogenic pathway that is a hybrid between the hydrogenotrophic and methylotrophic pathways [32]. Second, affinity and thresholds for H_2_ are lower for *Methanosphaera* compared with those of *Methanobrevibacter* [33]. Recently, Feldewert et al. [34] reported that CO_2_-reducing *Methanobrevibacter* species have higher H_2_ thresholds (> 5.0 Pa) compared with methanol-utilizing *Methanosphaera* (1.0 Pa) and methylamine-utilizing *Methanomassiliicoccales* (< 0.1 Pa) suggesting that methylamine- and methanol-utilizing methanogens have an advantage over CO_2_-reducing methanogens. Particularly under conditions when the dissolved H_2_ concentrations in the rumen are low and there is availability of methylamines and methanol substrates, methylotrophic methanogens may outcompete hydrogenotrophic methanogens and may serve as the major pathways for methanogenesis. However, when the concentrations of dissolved H_2_ in the rumen fluid are higher than the thresholds of hydrogenotrophic methanogens, the latter methanogens may dominate due to the abundance of CO_2_ concentrations in the rumen.

In the current study, although the overall contribution of hydrogenotrophic methanogens was much greater than methanol-utilizing and methylamine-utilizing methanogens, the genes encoding for the enzyme EC: 1.2.7.12, which facilitates conversion of CO_2_ to formylmethanofuran, were approximately 1000 cpm while its transcripts were 1500 cpm. In contrast, the genes encoding for the enzyme EC: 2.1.1.90, which facilitates conversion of methanol, were less than 50 cpm and its transcripts were less than 1000 cpm, suggesting a greater contribution from methanol-utilizing methanogens than what would be anticipated from their gene copies. Further, experimental dairy cows were transitioned from a fresh diet to a lactation diet by week 4 after calving and continued on the same lactation diet throughout the study. There were minor changes in the ingredient composition of fresh and lactation diets, but the chemical composition remained similar. There was a gradual increase in the relative abundance of *Methanosphaera* and transcripts of the methanol-reducing pathway from week 4 through week 12, but these increases may be attributed to an increase in DMI as dairy cows advanced in lactation rather than a carryover effect of the fresh diet at week 4. Recently, we reported a greater contribution of *Methanosphaera* compared with other methanogens in the rumen of dairy cows during the first 6 h post-feeding owing to the abundance of methylated substrates due to rapid fermentation of carbohydrates; a gradual decrease in *Methanosphaera* was observed after 6 h post-feeding with an increasing abundance of *Methanobrevibacter* around 10 h post-feeding [35]. Dietary sources including pectin, hemicellulose, choline, and glycine betaine are methyl compounds that are ultimately converted by microbial enzymes to methanol and methylamines in the rumen [27] that are then utilized by methylotrophic methanogens. With an increase in DMI, substrates supplying methyl groups may support an increase in the methanol-reducing pathway; however, more information on the different types of substrates and what conditions favor the abundance of *Methanosphaera* and *Methanomassiliicoccales* representatives and their contribution to total methanogenesis in a temporal manner using RNA-based approaches may help us better understand CH_4_ formation in the rumen.

The CH_4_ inhibitor 3-NOP is an analog of methyl Co-M and therefore inhibits MCR, an enzyme that catalyzes CH_4_ formation [36]. In the current study, abundance of genes encoding for MCR (EC: 2.8.4.1) did not differ between control and supplemented treatment groups. However, their corresponding transcripts were 3 times higher compared with their gene copies across all samples and showed a tendency to reduce in 3-NOP supplemented dairy cows compared with control cows. The effective inhibition of methanogenesis by 3-NOP was clearly observed in reduced gene expression more than gene content which corroborates the findings of Shi et al. [37] that CH_4_ yield in sheep is tightly correlated with gene expression. Further, as indicated in our previous report, the higher dose of 3-NOP ingested by the cows as a result of greater dry matter intakes may have led to greater inhibition of 3-NOP on the methanol pathway at week 8 [35], because a higher dose (>1 μM) of 3-NOP is needed to inhibit *Methanosphaera* whereas only 0.25 μM of 3-NOP is sufficient to inhibit *Methanobrevibacter ruminantium* [21]. Collectively, it can be inferred that 3-NOP inhibits MCR but has differential effects on individual methanogenic linages. It is important to understand the factors governing the distribution of individual methanogenic lineages and at what doses these lineages are inhibited to more effectively reduce CH_4_ formation in the rumen.

Previous reports [7, 18] showed that during 3-NOP supplementation to dairy cows for prolonged periods (15 weeks), while CH_4_ emissions were persistently reduced by 26 to 30%, both gaseous H_2_ measured in breath samples via GreenFeed and dissolved H_2_ concentrations in the rumen increased progressively from week 1 through week 9 and then declined by week 15 (SI Additional file 1: Table S13; Table S18). These changes in H_2_ emissions, although very difficult to measure and change rapidly with time and location in the rumen, may be explained 2 phases as follows: the first phase was characterized by a drop in CH_4_ formation resulting in a spike in dissolved H_2_ concentrations that accumulated during this time period. Based on DNA- and RNA-based 16S rRNA sequencing analysis, we found that certain bacteria including *Prevotella*, *Succinivibrionaceae*, *Veillonellaceae*, *Succiniclasticum*, and *Sharpea*, have been associated with rapid fermentation of hexoses, similar to the findings [38], were increased at week 8 when dissolved H_2_ concentrations were the highest but then were significantly lower at weeks 4 and 12. In contrast, the slow fermenting bacteria such as *Clostridiales*, *Butyrivibrio*, and *Ruminococcus* did not fluctuate as did the rapid fermenting bacteria but were increased by week 12. Because methanogens were inhibited, dissolved H_2_ may be directed towards other hydrogenotrophic bacteria that transiently increased in response to dissolved H_2_ accumulation. However, this increase in dissolved H_2_ concentration in the rumen may have stimulated the expression of H_2_-sensing hydrogenases that then began the second phase of H_2_ dynamics in the rumen. These H_2_-sensing [FeFe] hydrogenases, as described in Zheng et al. [39], then enabled expression of A1 [FeFe] hydrogenases that led to a reduction in H_2_ production by H_2_-producing bacteria. Interestingly, this shift was also accompanied by an increase in ethanol production ([26, 40] and 65 mg/kg of rumen contents at weeks 4, 8, and 12, respectively) suggesting that the amount of H_2_ released is indeed regulated in H_2_-producing bacteria under inhibited methanogenesis as described in Melgar et al. [18]. This process was also accompanied by a significant decrease in the molar proportion of acetate and an increase in molar proportion of butyrate in the rumen.

It has been reported that inhibited methanogenesis and increased H_2_ concentrations may result in an increase in partial pressure of hydrogen (P[H_2_]) [41] which may have happened in the rumen of cows that received 3-NOP supplementation. Greening et al. [42] reported differences in stoichiometries of *Ruminococcus albus 7* in response to high and low H_2_ concentrations in the rumen which have been attributed to the presence of putative sensory group C [FeFe] hydrogenases that can sense H_2_ concentrations. Accordingly, we have identified that group C hydrogenases were increased in 3-NOP supplemented cows compared with control cows at weeks 4, 8, and 12, thus indicating that increasing H_2_ concentrations in the rumen under inhibited methanogenesis induced by 3-NOP were sensed by group C [FeFe] hydrogenases, whereas these remained fairly stable in control cows. Although we did not see a consistent increase in expression of A1, we found that B group [FeFe] hydrogenases were consistently increased in 3-NOP supplemented cows to regulate the amount of H_2_ produced. In pure cultures of *R. albus*, H_2_ production is regulated by either A1 [FeFe] hydrogenases, which are ferredoxin-only hydrogenases, or the A3 group which are electron-bifurcating hydrogenases. Under low P[H_2_], such as when grown in the presence of methanogens, *R. albus* favors the energy-efficient pathway via production of acetate and H_2_ which is regulated by the electron-bifurcating A3 group of [FeFe] hydrogenases. However, under high P[H_2_], i.e., in the absence of methanogens, H_2_ production is regulated by the ferredoxin-only hydrogenase (group A1 [FeFe]-hydrogenase), a bifunctional alcohol and aldehyde dehydrogenase, and regulatory elements including a putative sensory hydrogenase (group C [FeFe]-hydrogenase) [42]. In the current study, H_2_ concentrations were higher at weeks 4 and 8 which may have resulted in a relatively lower expression of bifurcating enzymes [FeFe]A3 in 3-NOP supplemented dairy cows. By week 12, there had been adjustments within fiber-digesting bacteria resulting in a decrease in H_2_ concentrations in the rumen which was accompanied by an increase in electron-bifurcating enzymes. Based on these H_2_ concentrations in the rumen, the ratio of A1:A3 hydrogenase expression was regulated. This may be directly associated with H_2_ concentrations within the rumen with increasing H_2_ concentrations inversely related to bifurcating enzymes whereas these hydrogenases increased with a reduced H_2_ concentrations.

In the rumen, methanogens serve as the major H_2_ sink and this interdependency for H_2_ between methanogens and other fermenting microbes drives fermentation of feeds [43, 44]. Janssen [41] conceptualized a model in which changes in diet (altering forage to grain ratio), lowering pH in the rumen, and inhibiting methanogens may lead to an increase in P[H_2_], thus creating a negative feedback mechanism on H_2_-producing bacteria to reduce H_2_ production. This negative feedback mechanism results in a shift in fermentation pathways in H_2_-producing bacteria from higher H_2_ and acetate production to the formation of more reduced products such as succinate or ethanol as described in Greening et al. [42]. Melgar et al. [18] observed that molar concentration as well as proportion of acetate was reduced in the rumen contents collected from 3-NOP supplemented cows compared with control cows at weeks 4, 8, and 12. However, the H_2_ that would be spared by an approximately 26% reduction in CH_4_ has not been completely accounted for in that study. Although these authors reported a significant increase in pH, formic acid, ethanol, butyrate, gaseous H_2_ emission, and dissolved H_2_ in ruminal contents of cows supplemented with 3-NOP compared with control cows, these increases were not able to account for H_2_ spared under inhibited methanogenesis by 3-NOP. It is interesting to note that the genes or transcripts that code for some of the alternative sinks [42] such as nitrate and nitrite reductase, CO-dehydrogenase/acetyl CoA synthase, fumarate reductase, and sulfite reductase showed only marginal increases, and their overall contribution to the total gene or transcript abundance was insignificant. However, transcripts of formyl-tetrahydrofolate synthetase, a marker enzyme of acetogenesis or the Wood-Ljungdahl pathway, were increased in 3-NOP samples indicating that acetogens may have increased under inhibited methanogenesis. It has been reported that acetogens may serve as one of the alternative H_2_ sinks under reduced methanogenesis in sheep with low CH_4_-yield phenotype [42] and they are the main H_2_ sink in the intestinal tract of marsupials and termites. In a monoculture of *Acetobacterium woodii*, an acetogen, 1 mole of fructose is fermented to 3 moles of acetate, but this bacterium was shown to shift its fermentation pathway to produce 2 moles of acetate, 2 CO_2_, and 4 H_2_ when co-cultured with *Methanobacterium strain AZ*, which kept the H_2_ concentration in the media low [45]. Other than hydrogenotrophic methanogens, all known acetogens can grow on sugars and reduce CO_2_ with H_2_ to acetate only when sugars are not available. It is thus very likely that the acetogens in the rumen normally ferment sugars to 2 moles acetate, 2 CO_2_, and 4 H_2_ when the H_2_ concentration is very low and that they switch to forming 3 moles acetate when the H_2_ concentration increases after 3-NOP inhibition. Rather than reducing CO_2_ with 4 H_2_ to acetate, 4 H_2_ are spared by not being produced.

The steady-state acetate concentration in the rumen was found to be lower after 3-NOP supplementation, which does not exclude sugar-fermenting acetogens being involved as indirect sinks. In the rumen, ethanol is formed from sugars by bacteria such as *Ruminococcus albus* when the H_2_ concentration is high. The ethanol reacts with acetate to butyrate and caproate and with propionate to valarate in a fermentation catalyzed by *Clostridium kluyveri* [46]. Indeed, Melgar et al. [18] found that the steady-state ethanol concentration was significantly higher in 3-NOP supplemented rumen samples compared to control samples (26.68 mg/kg versus 16.51 mg/kg). Further, both butyrate and caproate were significantly higher in 3-NOP rumen samples compared to control samples. Butyrate and caproate formation from ethanol and acetate in *C. kluyveri* involves butyryl-CoA dehydrogenase, which catalyzes the reduction of ferredoxin and crotonyl-CoA with 2 NADH to butyryl-CoA and reduced ferredoxin [46]. In the current study, we noticed a significant increase in the genes and transcripts (overall across all sampling weeks) for butyryl-CoA dehydrogenase (EC: 1.3.8.1), by 10% and 15%, respectively. Furthermore, we also noted that *C. kluyveri’s* contribution for this enzyme was increased in 3-NOP samples compared to control samples.

There are 2 major pathways for butyrate synthesis in the rumen [27]: one mechanism mediated via the butyrate kinase pathway (BP1), which is mostly predominant in *Clostridia*, and the other mechanism mediated via the butyryl-CoA: acetate-CoA-transferase pathway (BP2) which is mostly predominant in *Negativicutes (Selenomondales*, *Veillonellaceae*, and *Acidaminococcaceae*) but also in *C. kluyveri.* It has been shown that BP2 and *C. kluyveri* are dependent on acetate for butyrate formation [47] which indicates a synergy between acetate-producing and butyrate-producing bacteria [48]. In the current study, we found that 3-NOP numerically increased the gene expression of the enzyme acetate-CoA transferase (EC: 2.8.3.8) by approximately 25%, suggesting that the BP2 pathway of butyrate synthesis may be an alternative pathway under inhibited methanogenesis by 3-NOP. However, it remains to be determined how much of spared H_2_ is diverted to butyrate and other reduced products such as ethanol and formic acid. Before we determine the fate of spared H_2_, it is also essential to determine how much H_2_ is spared. The amount of H_2_ spared under inhibited methanogenesis is dependent on methanogen diversity and to what extent individual methanogenic lineages are inhibited. Methanogens that reduce CO_2_ require 4 moles of H_2_ whereas methylotrophic methanogens require only 1 mole of H_2_; thus, the amount of H_2_ spared when the latter methanogens are inhibited is much lower than when the former methanogens are inhibited. Further studies on methanogen diversity and to what extent different CH_4_ inhibitors may inhibit individual methanogenic lineages may help to better quantify the amount of H_2_ spared and then to determine the diversion of H_2_ to alternate sinks to understand energy conservation in the rumen. Such information may help design strategies to formulate diets to reduce enteric CH_4_ formation without perturbing rumen microbiota and to safely divert H_2_ to more reduced fermentation products that are then available for the host metabolism.

## Conclusions

The main findings of this study indicate that methylotrophic methanogens may have a greater contribution to a total methanogenesis than what was originally thought and that metatranscriptomic approaches provide deeper insights on methanogenesis in the rumen. The inhibitor 3-NOP may have a differential effect on individual methanogenic lineages, which may be driven by several factors including dietary composition, dry matter intake, host genetics, and ruminal conditions including pH, VFA molar proportions, and partial pressure of H_2_ in the rumen. Further, increases in concentrations of spared H_2_ under inhibited methanogenesis may lead to a shift in fermentation pathways in H_2_-producing and H_2_-utilizing bacteria such as acetogens. More information is needed to determine how much H_2_ is spared under inhibited methanogenesis by 3-NOP, as the amount of H_2_ spared varied in a temporal manner. Fluctuations in H_2_ were accompanied by changes in hydrogenases, possibly indicating regulation of H_2_ concentrations by hydrogenases in H_2_-producing bacteria. While there appears to be no alternative sinks that can compete with methanogens for H_2_ under inhibited methanogenesis by 3-NOP, butyrate synthesis seems to be the compensatory H_2_ sink. The increase in butyrate concentrations may only be the consequence of a shift in acetogen fermentation pathways that lead to higher acetate production; however, certain bacteria such as *C. Kluyveri* may increase with 3-NOP supplementation and can ferment acetate and ethanol to form butyrate.

## Materials and methods

### Animals and experimental design

The current study was an accompaniment to the animal study described in Melgar et al. [18]. The study and all procedures involving animals were approved by The Pennsylvania State University Institutional Animal Care and Use Committee. The experiment lasted for 15 weeks. As part of the larger experiment (see more details in supplementary information text), 8 ruminally cannulated Holstein cows were enrolled in the experiment as they entered into lactation and were randomly assigned to either control (*n* = 4) or 3-NOP supplementation at 60 mg/kg of feed dry matter (*n* = 4). The 3-NOP supplement was incorporated into the total mixed ration as described in Melgar et al. [18]. It is normal to provide additional Net Energy Lactation (NE_L_) to meet higher energy requirements in the form of a fresh diet during the transition period. The cows were on a fresh diet for the first 3 weeks after calving and then were transitioned to a lactation diet by week 4 after calving. As the goal of the study was to investigate the effects of 3-NOP in early lactation, cows were enrolled as they entered lactation and remained in the study for 15 weeks. Ingredient and chemical composition of the fresh and lactation diets were described in Melgar et al. [18]. Feed analysis, measurement of enteric CH_4_ and dissolved H_2_ concentrations in ruminal fluid, and VFA analysis were described in Melgar et al. [18] and also included in supplementary information text. Methane (g/d), CH_4_ yield (g/kg DMI) and H_2_ measurements from animals in the study at experimental weeks 2, 6, 9, and 15 were found to be significantly different between the two treatment groups (*P* <0.01; Melgar et al. [18]) and are presented in Table S13. The 8 cannulated dairy cows were sampled for rumen contents at 2 h after feeding in experimental weeks 4, 8, and 12. Rumen samples were collected from 4 different (the ventral sac, the atrium or reticulum, and two samples from the feed mat) locations in the rumen, combined to represent a homogenous sample, filtered through 4 layers of cheesecloth to separate solid and liquid fractions, snap-frozen at cow side using liquid nitrogen, and then stored at −80°C until analysis. Both solid and liquid samples were processed for bacterial and archaeal diversity analysis, whereas only solid samples were used for metagenomic, metatranscriptomic, and rt-PCR analysis. Because the microbiome associated with the liquid fractions was found to vary with several factors including dilution with water intake, time of the day and accumulation of fermentation variables, and that the microbiome associated with the solid fraction remained stable throughout the day for individual cows [29], we selected only the solid samples for metagenomic and metatranscriptomic analysis.

### DNA and RNA extraction, PCR amplification, and sequencing

The genomic DNA from both the solid and liquid ruminal samples was extracted using the repeated bead beating and column (RBB + C) method followed by extraction with the QIAmp Fast DNA Stool Mini Kit (Qiagen Sciences; Germantown, MD, USA) as described in Yu and Morrison [49]. The RNA extraction from rumen samples was performed using the Trizol method. The steps involve bead beating in Trizol followed by chloroform and isopropanol extractions and finally by ethanol precipitation. RNA was reverse transcribed to cDNA using the SuperScript VILO cDNA Synthesis Kit (Invitrogen; Carlsbad, CA, USA) according to the manufacturer’s protocol. For each extracted genomic DNA and cDNA sample, both the V1–V2 regions of the bacterial 16S rRNA gene and the V6–V8 regions of the archaeal 16S rRNA gene were PCR-amplified in triplicate. The bacterial-specific primers used were F27 (5′-AGAGTTTGATCCTGGCTCAG-3′) and R338 (5′-TGCTGCCTCCCGTAGGAGT-3′); the archaeal-specific primers used were i958aF (5'-AATTGGAKTCAACGCCKGR-3') and i1378aR (5'-TGTGTGCAAGGAGCAGGGAC-3'). Both sets of primers were barcoded with a unique 12-base error-correcting Golay code for multiplexing as described in Song et al. [50]. Polymerase chain reaction was performed in triplicate using the Accuprime Taq DNA Polymerase System (Invitrogen). The thermal cycling conditions for PCR amplification of the bacterial 16S rRNA gene involved an initial denaturing step at 95°C for 5 min followed by 20 cycles (denaturing at 95°C for 30 s, annealing at 56°C for 30 s, extension at 72°C for 90 s) and a final extension step at 72°C for 8 min. The thermal cycling conditions for PCR amplification of the archaeal 16S rRNA gene involved an initial denaturing step at 94°C for 2 min followed by 30 cycles (denaturing at 94°C for 30 s, annealing at 56°C for 1 min 30 s, extension at 72°C for 30 s) and a final extension step at 72°C for 8 min. The triplicate amplicon products from each sample were pooled and then quantified using a Spectramax M2e microplate reader (Molecular Devices; San Jose, CA). The quantified amplicons were combined by adding each sample to a pool in equimolar concentration, and pools were bead purified using Agencourt AMPure XP Beads (Beckman-Coulter; Indianapolis, IN, USA). Sequencing was performed at the PennCHOP Microbiome Core, University of Pennsylvania, using the Illumina MiSeq platform. The rt-PCR assay on rumen samples was performed as described in Pitta et al. [35].

### Metagenomic and metatranscriptomic analysis

For metagenomics, DNA was prepared for whole-genome shotgun sequencing using the Nextera DNA Library Prep Kit (Illumina; San Diego, CA, USA). The library (tight insert size of 250 bp for high-throughput sequencing from both ends by 2 × 150 bp) was sequenced on an Illumina HiSeq 2500 at the Center for Host-Microbe Interactions at the University of Pennsylvania School of Veterinary Medicine. For metatranscriptomics, ribosomal RNA was depleted from total RNA using the Ribo-Zero Plus rRNA Removal Kit (Illumina). Double-stranded cDNA was synthesized from the mRNA-enriched RNA using the TruSeq Stranded mRNA Kit (Illumina), and a library was constructed. The library was sequenced on an Illumina NextSeq 500 at the PennCHOP Microbiome Core, University of Pennsylvania.

### Bioinformatic analysis

The DNA- and RNA-based amplicon 16S rRNA sequences for archaeal and bacterial diversity were analyzed according to the method previously described [35]. The metagenomic and metatranscriptomic sequences were demultiplexed, and the adapter was trimmed at the sequencing facility. These raw sequences were subjected to quality trimming using Trimmomatic (0.36) [51] according to the following parameters: starting from either end of the sequence, bases were trimmed off if their Phred quality score was < 3 or if they appeared as *N*; bases were trimmed off if their average Phred quality score was < 15 when the sequence was analyzed on a 4-base sliding window; and sequences were removed if they were shorter than 36 bases in length. Reads aligning to the host genome (ARS-UCD1.2/bosTau9) were identified and removed using Bowtie2 (v2.2.7) [52] with parameters set by the flag --very sensitive local --un-conc. For metatranscriptomic sequences, an additional quality filtering step was applied in which the rRNA and tRNA sequences were removed using SortmeRNA (v2.1) [53]. Taxonomic labels were assigned to quality-controlled reads by mapping sequences to a low-complexity masked database of bacterial, archaeal, viral, fungal, and protozoal sequences from NCBI complete genomes. The relative abundance of the bacterial and archaeal taxonomy was estimated using Kraken2, version 2.1.1 [54]. The reads were mapped to the KEGG [55] protein database to estimate abundance of microbial gene orthologs using DIAMOND [56], and the functional profiles were performed by HUMAnN2 [57]. The abundance of orthologs was then annotated to Enzyme Commission (EC) numbers already present in the KEGG database. The genes/transcripts that encoded for enzymes at each step of the methanogenesis pathway were identified based on a literature search [27, 30, 37, 40] and the Kyoto Encyclopedia of Genes and Genomes (KEGG) database, and the different methanogenesis pathways were constructed and described by our group in Pitta et al. ([23] Figure S2). Using metagenomic and metatranscriptomic data, we quantified the genes and transcripts of enzymes involved in the 3 predominant ruminal methanogenesis pathways (CO_2_-, methanol-, and methylamine-reducing pathways) along with the butyrate and propanoate pathways in cows with and without 3-NOP supplementation at weeks 4, 8, and 12 of the experimental period (Fig. 2). In addition, taxonomy of the annotated genes and transcripts was also tracked to help us understand the role of individual methanogenic lineages in methanogenesis. Further, hydrogenases were confirmed and classified by aligning the quality filtered reads to a hydrogenase database [42] using the DIAMOND [56] search tool.

We have also derived MCR gene profiles for metagenomics and metatranscriptomic data using metagenome assembled genomes (MAGs) approach. Only 61 MAGs were assembled (Table S25); of these, 54 are bacteria and 7 are methanogens. Among the bacteria, MAG were identified only to class and order level except for *Methanobrevibacter*, *Methanosphaera*, *Prevotella*, *Butyrivibrio*, *Succiniclasticum ruminis*, *Lachnospiraceae bacterium NE2001*, *Sarcina*, *Butyrivibrio*, *Bifidobacterium*, and *Pseudobutyrivibrio*, which showed taxonomy only to the genus or species level. Among the 7 methanogen MAG, 6 were identified as *Methanobrevibacter* genus (species not identified) and only one MAG was identified as *Methanosphaera*. To these MAGs, metagenomics and metatranscriptomic reads were aligned and results were compared for selected enzymes such as MCR which is encoded by *mcra*, *mcrb*, and *mcry.* The methodology used MAG construction, and other bioinformatics details are described in supplementary note (SI Additional file 1).

### Statistical analysis

To facilitate comparisons between samples with different sequencing depths, the gene orthologs were normalized to cpm. Absolute abundance of taxonomy values was compositionally normalized (relative abundance) such that each sample summed to 1. All of the statistical analysis was performed in R [58]. To test for differences in 16S-based amplicons and metagenomic and metatranscriptomic taxonomy analysis, a generalized linear mixed model was constructed with the lme4 package for R [59]. The model used treatment and week as fixed effects with a binomial family object and cow included as a random effect term. For the KEGG gene orthologs, we used treatment as fixed effect with Poisson family object and included a random effect term of week: treatment. The *P* values for multiple tests were corrected using the Benjamini-Hochberg approach. To determine whether the individual methanogens derived from rt-PCR were significantly different between treatment groups, we conducted the Wilcoxon test. The Spearman correlation coefficients were calculated to evaluate correlations between relative abundance of bacterial genera (DNA- and RNA-based 16S rRNA) and fermentation profiles. We considered the relationships with the criteria of absolute correlation coefficients greater than 0.5 and *P* values less than 0.05 as significant.

## Supplementary Information

**Additional file 1: Supplementary Information Text.** **Figure S1**: Alpha diversity based on 16S rRNA amplicon archaeal sequencing data in cows supplemented with 3-nitrooxypropanol (3-NOP) at weeks 4, 8, and 12. (A) observed species in DNA liquid; (B) Shannon diversity in DNA liquid; (C) observed species in RNA liquid; (D) Shannon diversity in RNA liquid. NS = not significant. **Figure S2**: Schematic diagram of possible methanogenesis pathways. **Table S1**: Sequencing information for metagenomics. Trt: treatment group; W: week; 3-NOP: 3-nitrooxypropanol. **Table S2**: Sequencing information for metatranscriptomics. Trt: treatment group; W: week 3-NOP: 3-nitrooxypropanol. **Table S3**: Relative abundance (%) of archaeal taxonomy in metagenomics. SEM: Standard error of mean; Trt: treatment group; W: week; 3-NOP: 3-nitrooxypropanol. **Table S4**: Relative abundance (%) of archaeal taxonomy in metatranscriptomics, SEM: Standard error of mean; Trt: treatment group; W: week; 3-NOP: 3-nitrooxypropanol. **Table S5**: The 6 most abundant archaea (cpm; copies per million) contributing to steps 1-5 in the carbon dioxide (CO_2_)-hydrogen (H_2_) methanogenic pathway in the rumen of dairy cows supplemented with 3-nitrooxypropanol (3-NOP) compared to control cows at weeks 4, 8, and 12. **Table S6**: The most abundant archaea (cpm; copies per million) utilizing methanol as a substrate for methanogenesis in the rumen of dairy cows supplemented with 3-NOP compared to control at weeks 4, 8, and 12. **Table S7**: The most abundant archaea (cpm; copies per million) utilizing methylamines as a substrate for methanogenesis in the rumen of dairy cows supplemented with 3-NOP compared to control at weeks 4, 8, and 12. **Table S8**: Transcripts (cpm; copies per million) coding for EC: 1.8.7.3 (HdrA, HdrB and HdrC) in cows supplemented with 3-nitrooxypropanol (3-NOP) compared to control cows at weeks 4, 8, and 12. ND = not detected. **Table S9**: Effect of 3-nitrooxypropanol (3-NOP) on bacterial taxonomical composition (relative abundance %) in DNA-based 16S rRNA sequencing. SEM: Standard error of mean; Trt: treatment group; W: week; 3-NOP: 3-nitrooxypropanol. **Table S10**: Effect of 3-nitrooxypropanol (3-NOP) on bacterial taxonomical composition (relative abundance %) in RNA-based 16S rRNA sequencing. SEM: Standard error of mean; Trt: treatment group; W: week. **Table S11**: Effect of 3-nitrooxypropanol (3-NOP) on bacterial taxonomical composition (relative abundance %) in metagenomics. SEM: Standard error of mean; Trt: treatment group; W: week. **Table S12**: Effect of 3-nitrooxypropanol (3-NOP) on bacterial taxonomical composition (relative abundance %) in metatranscriptomics. SEM: Standard error of mean; Trt: treatment group; W: week. **Table S13:** Average methane (CH_4_) and hydrogen (H_2_) gas data in control and 3-nitrooxypropanol (3-NOP) treated cows at weeks 2, 6, 9, and 15. **Table S14**: Effect of 3-nitrooxpropanol (3-NOP) on hydrogenase subgroups (cpm; copies per million) including [FeFe], [Fe], and [NiFe] hydrogenases in metagenomics. **Table S15**: Effect of 3-nitrooxpropanol (3-NOP) on hydrogenase subgroups (cpm; copies per million) including [FeFe], [Fe], and [NiFe] hydrogenases in metatranscriptomics. **Table S16**: Effect of 3-NOP on hydrogenases (cpm; copies per million) classified into H_2_ production and H_2_ consumption in metagenomics. **Table S17**: Effect of 3-NOP on hydrogenases (cpm; copies per million) classified into H_2_ production and H_2_ consumption in metatranscriptomics. **Table S18**: Effect of 3-nitrooxypropanol (3-NOP) on rumen fermentation variables in early-lactation dairy cows at weeks 4, 8, and 12. **Table S19:** Taxonomy (cpm; copies per million) associated with the butyrate pathway in metagenomics in cows supplemented with 3-nitrooxypropanol (3-NOP) compared to control cows at weeks 4, 8, and 12. **Table S20:** Taxonomy (cpm; copies per million) associated with the butyrate pathway in metatranscriptomics in cows supplemented with 3-nitrooxypropanol (3-NOP) compared to control cows at weeks 4, 8, and 12. **Table S21:** Taxonomy (cpm; copies per million) associated with the propionate pathway in metagenomics in cows supplemented with 3-nitrooxypropanol (3-NOP) compared to control cows at weeks 4, 8, and 12. **Table S22:** Taxonomy (cpm; copies per million) associated with the propionate pathway in metatranscriptomics in cows supplemented with 3-nitrooxypropanol (3-NOP) compared to control cows at weeks 4, 8, and 12**. Table S23**: List of metagenomes assembled genomes (MAGs) identified in early-lactation dairy cows. **Table S24:** Transcripts (cpm; copies per million) coding for EC: 1.8.98.1 in cows supplemented with 3-nitrooxypropanol (3-NOP) compared to control cows at weeks 4, 8, and 12. ND = not detected. **Table S25:** Transcripts (cpm; copies per million) coding for EC: 1.8.98.5 in cows supplemented with 3-nitrooxypropanol (3-NOP) compared to control cows at weeks 4, 8, and 12. ND = not detected. **Table S26:** Transcripts (cpm; copies per million) coding for EC: 1.8.98.6 in cows supplemented with 3-nitrooxypropanol (3-NOP) compared to control cows at weeks 4, 8, and 12. ND = not detected.

## Data Availability

The raw sequences from DNA- and RNA-based 16S rRNA archaeal and bacterial sequencing, metagenomics, and metatranscriptomics have been deposited in the NCBI Sequence Read Archive (SRA) database under Bio Project accession number PRJNA666417.
