# Supplementary material for: The effect of 3-nitrooxypropanol, a potent methane inhibitor, on ruminal microbial gene expression profiles in dairy cows
Source: Microbiome. 2022 Sep 13;10:146. doi: 10.1186/s40168-022-01341-9 (PMC9469553; doi:10.1186/s40168-022-01341-9)
Supplement: Supplementary file 2 — Additional file 1: Supplementary Information Text. Figure S1: Alpha diversity based on 16S rRNA amplicon archaeal sequencing data in cows supplemented with 3-nitrooxypropanol (3-NOP) at weeks 4, 8, and 12. (A) observed species in DNA liquid; (B) Shannon diversity in DNA liquid; (C) observed species in RNA liquid; (D) Shannon diversity in RNA liquid. NS = not significant. Figure S2: Schematic diagram of possible methanogenesis pathways. Table S1: Sequencing information for metagenomics. Trt: treatment group; W: week; 3-NOP: 3-nitrooxypropanol. Table S2: Sequencing information for metatranscriptomics. Trt: treatment group; W: week 3-NOP: 3-nitrooxypropanol. Table S3: Relative abundance (%) of archaeal taxonomy in metagenomics. SEM: Standard error of mean; Trt: treatment group; W: week; 3-NOP: 3-nitrooxypropanol. Table S4: Relative abundance (%) of archaeal taxonomy in metatranscriptomics, SEM: Standard error of mean; Trt: treatment group; W: week; 3-NOP: 3-nitrooxypropanol. Table S5: The 6 most abundant archaea (cpm; copies per million) contributing to steps 1-5 in the carbon dioxide (CO2)-hydrogen (H2) methanogenic pathway in the rumen of dairy cows supplemented with 3-nitrooxypropanol (3-NOP) compared to control cows at weeks 4, 8, and 12. Table S6: The most abundant archaea (cpm; copies per million) utilizing methanol as a substrate for methanogenesis in the rumen of dairy cows supplemented with 3-NOP compared to control at weeks 4, 8, and 12. Table S7: The most abundant archaea (cpm; copies per million) utilizing methylamines as a substrate for methanogenesis in the rumen of dairy cows supplemented with 3-NOP compared to control at weeks 4, 8, and 12. Table S8: Transcripts (cpm; copies per million) coding for EC: 1.8.7.3 (HdrA, HdrB and HdrC) in cows supplemented with 3-nitrooxypropanol (3-NOP) compared to control cows at weeks 4, 8, and 12. ND = not detected. Table S9: Effect of 3-nitrooxypropanol (3-NOP) on bacterial taxonomical composition (relative abundance % [file 40168_2022_1341_MOESM1_ESM.docx]

**Supplementary Information for**

**The effect of 3-nitrooxypropanol, a potent methane inhibitor, on ruminal microbial gene expression profiles in dairy cows**

Dipti W. Pitta**^*^**, Nagaraju Indugu, Audino Melgar, Alexander Hristov, Krishna Challa, Bonnie Vecchiarelli, Meagan Hennessy, Kapil Narayan, Stephane Duval, Maik Kindermann, Nicola Walker

Corresponding author: Dipti W. Pitta

Email: dpitta@vet.upenn.edu

This PDF includes:

Supplementary Information Text

Figures S1 to S2

Tables S1 to S25

Supplementary references

**Supplementary Information Text**

**Experimental Design**

Details of the experiment was described in Melgar et al. [1]. The study was a 15-wk randomized complete block design and was conducted in 2 phases at The Pennsylvania State University’s Dairy Teaching and Research Center (University Park) tiestall barn. Phase 1 was conducted from January to June, and phase 2 from mid-June to December 2016. The experiment had to be conducted in 2 phases due to limited tiestall space for the number of cows enrolled in the study. A total of 56 multi- and primiparous cows (n = 44 and 12, respectively), including 8 rumen cannulated (10.2 cm internal diameter cannulas; Bar Diamond Inc., Parma, ID), were used in the experiment. Phase 1 involved 30 cows, and phase 2 involved 26 cows. The 8 cannulated cows were distributed between the two phases with 4 cannulated cows in each phase.

**Measurement of CH4 and H2 in experimental animals**

Enteric CH4 and H2 were measured using the GreenFeed system (C-Lock Inc., Rapid City, SD). Measurements were conducted following the procedures recommended by Hristov et al. [2.3]. Briefly, during each measurement period, spot gas emission data were collected over 3 d as follows: starting at 0900, 1500, and 2100 h (d 1), 0300, 1200, and 1700 h (d 2), and 0000 and 0500 h (d 3) to obtain a representative sample of a 24-h feeding cycle. Individual breath gas samples were collected for 5 min followed by a 2-min background sample collection. A cannula extension (fistula attachment; C-Lock Inc.) was used when measuring gas from the cannulated cows to capture and direct through GreenFeed rumen gas potentially leaking through the cannula, as described in Lopes et al. [4]. The cannula extension consisted on an impermeable fabric covering the back and sides of the cow with a tubing attached to it in close proximity of the rumen cannula and extending from the cannula to the GreenFeed unit. The tubing was attached to a port that directed the collected gas toward GreenFeed sensors. Constant negative pressure was maintained by using a vacuum pump to withdraw gas from between the fabric sheet and the animal into the tubing. The gas was then routed from the tubing and released into the GreenFeed air collection pipe where total airflow and concentrations of CH4 and H2 were continuously measured during collection time.

A pelletized cattle feed (Stoker Grower 14, Purina Animal Nutrition LLC, Shoreview, MN) was used as bait feed and was offered at each sampling event for a total of 4,000 g/cow over 3 d, and the amount was included in the DMI value during the gas sampling week. The GreenFeed system is equipped with a head position sensor and gas emission data are rejected when the cow’s head position criteria are not met. The GreenFeed unit was calibrated following the manufacturer’s recommendations.

**Metagenomics assembled genomes (MAGs) construction**

The quality filtered reads from each sample were assembled using idba_ud (v 1.1.3, [5]). A co-assembly was also performed for control and 3-NOP treatment groups at weeks 4,8 and 12 using MEGAHIT (v1.2.9; [6]). Metagenomic binning was applied to both single-sample assemblies and the co-assemblies using three binning tools concoct (1.1.0 ; [7]), MaxBin (v 2.2.7; [8] ), and MetaBAT (v 2.12.1,[9]). The bins obtained from these binning tools were further refined and binned using DAS tool (v1.1.4; [10]). Both single samples bins and co-assembled bins were aggregated and then dereplicated using dRep (v.1.1.2;[11]). Further, the dereplicated bins were assessed for completeness (≥80%) and contamination (≤10%) using CheckM (v.1.0.5;[12]) which produced a total of 61 MAGs. Next, Prodigal (v2.6.3;[13]) was used to identify the protein-coding sequences (CDS) from the MAGs with meta option. All complete genes were clustered at protein level using CD-HIT (v4.8.1) at 95% sequence similarity. The amino acid sequences of proteins in the catalog were functionally annotated by aligning proteins sequences to the KEGG database [14] by DIAMOND (v0.9.21.122; [15]) with e-values ≤1e−5. The individual metagenomics and metatrancipomics reads were aligned to protein gene catalogue using BWA-mem [16] and the number of reads mapped to each gene was calculated using featureCounts (v2.0.1; [17]). These counts data further normalized to copies per million (cpm) and the genes encoding for MCR enzyme were retrieved and presented in Table 2. The taxonomical classification for the MAGs was determined using Bin Annotation Tool (BAT;[18]). The list of MAGs identified in this study are listed in Table S23.

**Sequencing information**

For metagenomics (Table S1), the Illumina HiSeq generated a total of 547,657,259 sequences for 24 samples. After quality filtering, approximately 28% of reads were eliminated resulting in a total of 393,741,632 quality filtered reads with a range of 14,596,178 – 18,587,255 (min – max) sequences per sample. Taxonomy assignment with Kraken2 revealed that approximately 92% and 6% of sequences were assigned to bacteria and archaea, respectively. Eukaryotes and viruses constituted a very small proportion. In metatranscriptomics data (Table S2), a total of 1,265,077,834 raw sequences were obtained for 24 samples. After quality filtering, approximately 37% of sequences were eliminated resulting in a total of 789,672,961 quality filtered reads with a range of 4,934,812 – 103,203,144 (min – max) sequences per sample. Taxonomy assignment with Kraken2 revealed that approximately 90% and 8% of sequences were assigned to bacteria and archaea, respectively. Eukaryotes and viruses constituted a very small proportion.

**Impact of 3-NOP on heterodisulfide reductase, an enzyme that mediates energy conservation in methanogens**

The heterodisulphide reductase complex (Hdr) catalyzes the formation of heterodisulphide and releases Co-M and Co-B in the final step of the methanogenesis pathway. It is not clear how the enzyme complex works in different methanogens. As per the KEGG database, we found 5 enzymes involved in this reaction: i) EC: 1.8.7.3 (Co-B, Co-M:ferredoxin oxidoreductase); ii) EC: 1.8.98.1 (Co-B, Co-M:methanophenazine oxidoreductase); iii) EC: 1.8.98.4 (Co-B, Co-M, ferredoxin:coenzyme F420 oxidoreductase); iv) EC: 1.8.98.5 (Co-B, Co-M, ferredoxin:H_2_ oxidoreductase); and v) EC: 1.8.98.6 (Co-B, Co-M ferredoxin:formate oxidoreductase).

1. **EC: 1.8.7.3:** The heterodisulphide reductase (Hdr) enzyme consists of 5 units: Hdr A, B, C, D, and E. The enzyme EC: 1.8.7.3 includes 3 units (Hdr A-C) that are present in all methanogens whereas the enzyme EC: 1.9.98.1 includes HdrD and E units that are present in methanogens with cytochromes only. The enzyme EC: 1.8.7.3 (Table S8) is present in methanogens without cytochromes and is composed of six subunits (A1, B1, C1, A2, B2, and C2) encoded by 6 genes. Interestingly, we did not detect A2, B2, or C2 in isolation.
2. **EC: 1.8.98.1:** The enzyme Co-B, Co-M:methanophenazine oxidoreductase (EC: 1.8.98.1) is present in methanogens with cytochromes such as methylotrophic methanogens. The transcripts coding for HdrD only were detected at very low levels and HdrE was not detected (Table S24). The major archaea associated with HdrD was *Methanogenic archaeon ISO4* species. Reduction in HdrD transcripts in response to 3-NOP was substantial at week 4 but the effect reduced by weeks 8 and 12.
3. **EC: 1.9.98.4:** The genes for this enzyme are encoded by Hdr A2, B2 and C2 subunits. None of these subunits were detected in our study.
4. **EC: 1.9.98.5:** We have identified 3 genes coding for the large subunit, small subunit, and one iron-sulfur subunit of Co-B, Co-M ferredoxin:hydrogenase oxidoreductase (Table S25). All hydrogenotrophic methanogens and species of *Methanosphaera* appear to contribute to this enzyme but none of the methylotrophic methanogens had genes coding for this enzyme.
5. **EC: 1.8.98.6:** This enzyme involves HdrA2B2C2 complexing with formate dehydrogenase. This enzyme has 2 subunits (alpha and beta) encoded by 2 genes. Only *Methanobrevibacter* species have the ability to utilize formate as a substrate for methane formation. However, genes coding for EC: 1.17.1.9 (formate dehydrogenase) were not identified in this study; instead formate dehydrogenase was complexed with HdrA2B2C2 represented by EC: 1.8.98.6 (Table S26). Transcripts involved in step 8 were reduced in 3-NOP supplemented cows at weeks 4, 8, and 12.

**Taxonomy associated with the butyrate pathway**

In Steps 1, 2, and 3A of the butyrate pathway (Table S19 and S20), the majority of transcripts (more than half) were contributed by *Eubacterium hallii, Butyrivibrio proteoclasticus, Butyrivibrio hungatei, Coprococcus catus, and Butyrivibrio fibrisolvens*. The step that involved conversion of crotonyl-CoA to butanoyl-CoA had lower transcript numbers compared to its genes; these were contributed by several bacteria. The conversion of butanoyl-CoA to butyric acid was facilitated by *Eubacterium* and *Clostridium* species but the overall transcript number for this step was much lower compared to its corresponding genes. A similar trend (higher gene copies and lower transcript copies) was observed for conversion of butanoyl-CoA to butanoylphosphate and then to butyric acid. *Prevotella ruminicola, B. proteoclasticus,* and *B. hungatei* had the greatest number of transcript copies for this gene. Overall, it appears that *B. proteoclasticus* and *B. hungatei* contributed to approximately 40% of butyrate formation.

**Supplementary references**

1. Melgar A, Harper MT, Oh J, Giallongo F, Young ME, Ott TL, Duval S, Hristov AN. Effects of 3-nitrooxypropanol on rumen fermentation, lactational performance, and resumption of ovarian cyclicity in dairy cows. J Dairy Sci. 2020 Jan 1;103(1):410-32. doi:10.3168/jds.2019-17085
2. Hristov, A. N., J. Oh, F. Giallongo, T. Frederick, M. T. Harper, H. L. Weeks, A. F. Branco, P. J. Moate, M. H. Deighton, S. R. O. Williams, M. Kindermann, and S. Duval. An inhibitor persistently decreased enteric methane emission from dairy cows with no negative effect on milk production. Proc. Natl. Acad. Sci. USA, 2015, 112:10663–10668. <https://doi.org/10.1073/pnas.1504124112>.
3. Hristov, A. N., J. Oh, F. Giallongo, T. Frederick, H. Weeks, P. R. Zimmerman, M. T. Harper, R. A. Hristova, R. S. Zimmerman, and A. F. Branco. The use of an automated system (GreenFeed) to monitor enteric methane and carbon dioxide emissions from ruminant animals. J. Vis. Exp. 2015. 103:e52904. <https://doi.org/10.3791/52904>.
4. Lopes, J. C., L. F. de Matos, M. T. Harper, F. Giallongo, J. Oh, D. Gruen, S. Ono, M. Kindermann, S. Duval, and A. N. Hristov. Effect of 3-nitrooxypropanol on methane and hydrogen emissions, methane isotopic signature, and ruminal fermentation in dairy cows. J. Dairy Sci. 2016. 99:5335–5344. <https://doi.org/10.3168/jds.2015-10832>.
5. Peng Y, Leung HC, Yiu SM, Chin FY. IDBA-UD: a de novo assembler for single-cell and metagenomic sequencing data with highly uneven depth. Bioinformatics. 2012 Jun 1;28(11):1420-8.
6. Li D, Liu CM, Luo R, Sadakane K, Lam TW. MEGAHIT: an ultra-fast single-node solution for large and complex metagenomics assembly via succinct de Bruijn graph. Bioinformatics. 2015 May 15;31(10):1674-6.
7. Alneberg J, Bjarnason BS, De Bruijn I, Schirmer M, Quick J, Ijaz UZ, Lahti L, Loman NJ, Andersson AF, Quince C. Binning metagenomic contigs by coverage and composition. Nature methods. 2014 Nov;11(11):1144-6.
8. Wu YW, Simmons BA, Singer SW. MaxBin 2.0: an automated binning algorithm to recover genomes from multiple metagenomic datasets. Bioinformatics. 2016 Feb 15;32(4):605-7.
9. Kang DD, Li F, Kirton E, Thomas A, Egan R, An H, Wang Z. MetaBAT 2: an adaptive binning algorithm for robust and efficient genome reconstruction from metagenome assemblies. PeerJ. 2019 Jul 26;7:e7359.
10. Sieber CM, Probst AJ, Sharrar A, Thomas BC, Hess M, Tringe SG, Banfield JF. Recovery of genomes from metagenomes via a dereplication, aggregation and scoring strategy. Nature microbiology. 2018 Jul;3(7):836-43.
11. Olm MR, Brown CT, Brooks B, Banfield JF. dRep: a tool for fast and accurate genomic comparisons that enables improved genome recovery from metagenomes through de-replication. The ISME journal. 2017 Dec;11(12):2864-8.
12. Parks DH, Imelfort M, Skennerton CT, Hugenholtz P, Tyson GW. CheckM: assessing the quality of microbial genomes recovered from isolates, single cells, and metagenomes. Genome research. 2015 Jul 1;25(7):1043-55.
13. Hyatt D, Chen GL, LoCascio PF, Land ML, Larimer FW, Hauser LJ. Prodigal: prokaryotic gene recognition and translation initiation site identification. BMC bioinformatics. 2010 Dec;11(1):1-1.
14. Ogata H, Goto S, Sato K, Fujibuchi W, Bono H, Kanehisa M. KEGG: Kyoto Encyclopediaof Genes and Genomes. Nucl Acids Res. 2000 Jan 1;28(1):27-30. <https://doi.org/10.1093/nar/28.1.27>
15. Buchfink B, Xie C, Huson DH. Fast and sensitive protein alignment using DIAMOND. Nat Methods. 2015 Jan;12(1):59-60. doi: 10.1038/nmeth.3176
16. Li H. Aligning sequence reads, clone sequences and assembly contigs with BWA-MEM. arXiv preprint arXiv:1303.3997. 2013 Mar 16.
17. Liao Y, Smyth GK, Shi W. featureCounts: an efficient general purpose program for assigning sequence reads to genomic features. Bioinformatics. 2014 Apr 1;30(7):923-30.
18. von Meijenfeldt FA, Arkhipova K, Cambuy DD, Coutinho FH, Dutilh BE. Robust taxonomic classification of uncharted microbial sequences and bins with CAT and BAT. Genome biology. 2019 Dec;20(1):1-4.


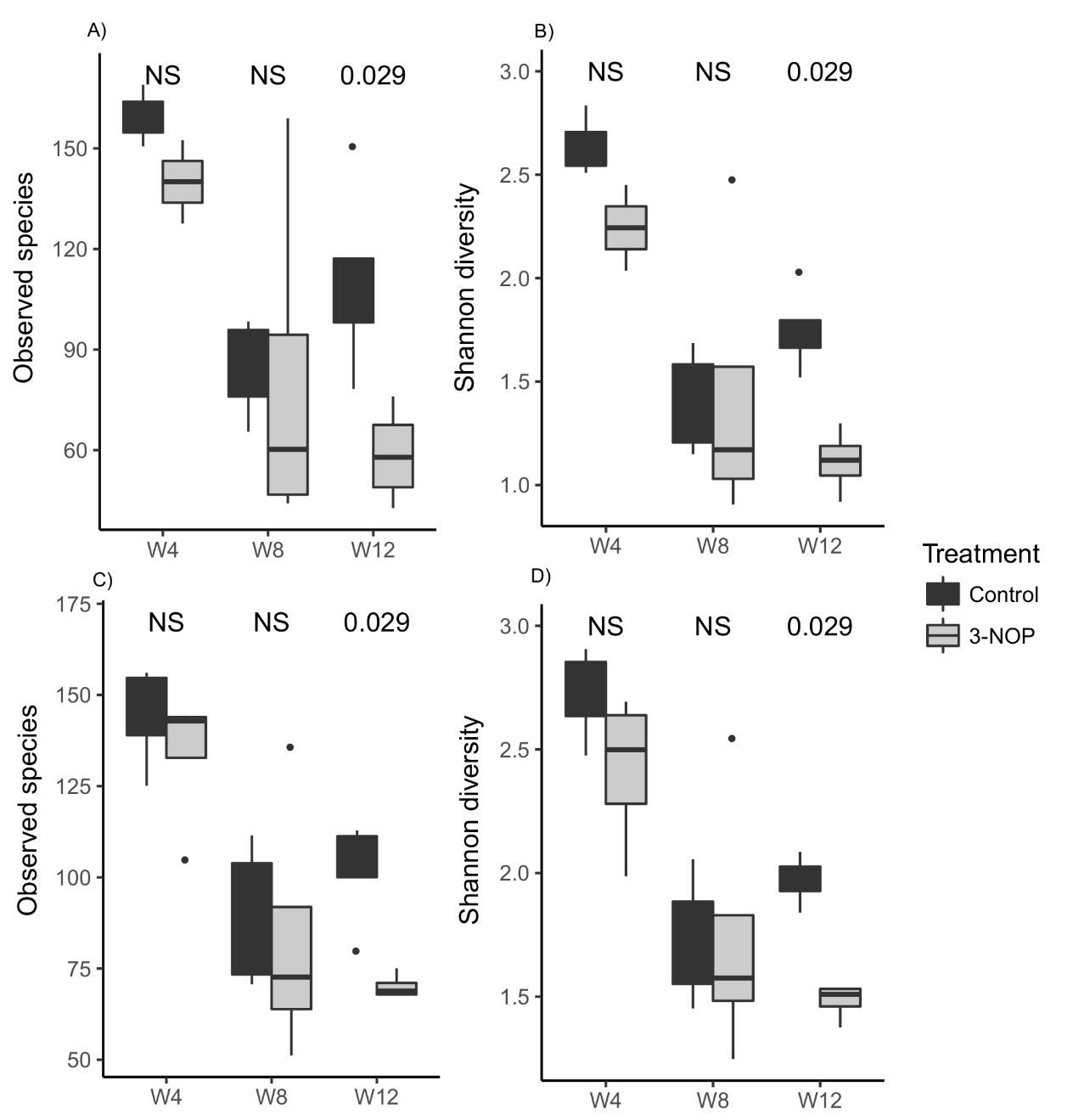


**Figure S1**: Alpha diversity based on 16S rRNA amplicon archaeal sequencing data in cows supplemented with 3-nitrooxypropanol (3-NOP) at weeks 4, 8, and 12. (A) observed species in DNA liquid; (B) Shannon diversity in DNA liquid; (C) observed species in RNA liquid; (D) Shannon diversity in RNA liquid. NS = not significant.


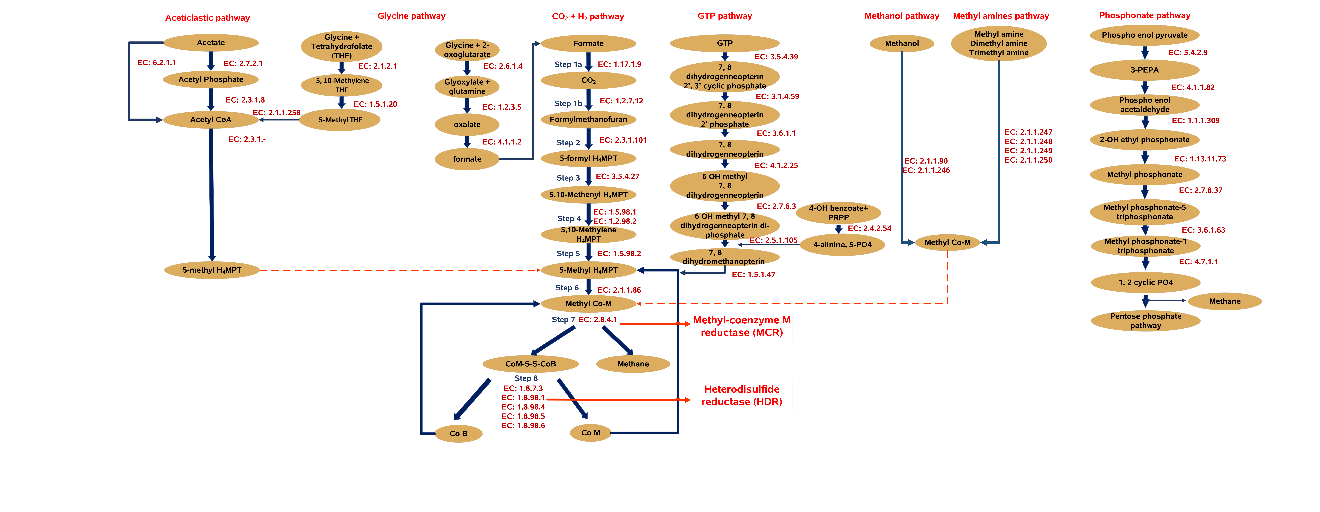


**Figure S2**: Schematic diagram of possible methanogenesis pathways.

**Table S1**: Sequencing information for metagenomics. Trt: treatment group; W: week; 3-NOP: 3-nitrooxypropanol.

| **Sample ID** | **Trt** | **Cow ID** | **Week** | **Number of**  **raw reads (paired)** | **Number of reads**  **after quality filtering (paired)** | **Bacteria**  **(%)** | **Archaea**  **(%)** | **Eukaryota**  **(%)** | **Viruses**  **(%)** | **Unassigned**  **(%)** |
| --- | --- | --- | --- | --- | --- | --- | --- | --- | --- | --- |
| PCMP_s.6S | 3-NOP | 2147 | W12 | 23430368 | 16736992 | 92.37 | 6.75 | 0.48 | 0.09 | 0.31 |
| PCMP_s.14S | 3-NOP | 2147 | W4 | 20680891 | 14775401 | 91.31 | 7.87 | 0.43 | 0.09 | 0.29 |
| PCMP_s.23S | 3-NOP | 2147 | W8 | 23448895 | 17028256 | 93.83 | 5.15 | 0.54 | 0.16 | 0.31 |
| PCMP_s.13S | 3-NOP | 2156 | W12 | 21757903 | 15020873 | 94.01 | 5.08 | 0.52 | 0.09 | 0.30 |
| PCMP_s.7S | 3-NOP | 2156 | W4 | 22888546 | 17035222 | 91.39 | 7.75 | 0.48 | 0.09 | 0.29 |
| PCMP_s.20S | 3-NOP | 2156 | W8 | 21864594 | 15305346 | 93.49 | 5.71 | 0.41 | 0.09 | 0.30 |
| PCMP_s.24S | 3-NOP | 2277 | W12 | 21908368 | 15894691 | 93.81 | 5.31 | 0.48 | 0.09 | 0.30 |
| PCMP_s.11S | 3-NOP | 2277 | W4 | 20014787 | 14596178 | 96.92 | 2.20 | 0.50 | 0.09 | 0.28 |
| PCMP_s.8S | 3-NOP | 2277 | W8 | 20740596 | 15004335 | 95.47 | 3.74 | 0.43 | 0.08 | 0.27 |
| PCMP_s.3S | 3-NOP | 2410 | W12 | 23370054 | 16623735 | 93.84 | 5.33 | 0.43 | 0.09 | 0.31 |
| PCMP_s.17S | 3-NOP | 2410 | W4 | 23260781 | 16993441 | 91.48 | 7.69 | 0.44 | 0.09 | 0.30 |
| PCMP_s.5S | 3-NOP | 2410 | W8 | 24006999 | 16523183 | 94.83 | 4.30 | 0.49 | 0.09 | 0.29 |
| PCMP_s.16S | Control | 2270 | W12 | 22617730 | 15987053 | 93.24 | 5.90 | 0.45 | 0.09 | 0.31 |
| PCMP_s.9S | Control | 2270 | W4 | 23233241 | 16753145 | 92.06 | 7.10 | 0.46 | 0.09 | 0.29 |
| PCMP_s.4S | Control | 2270 | W8 | 23356583 | 16940741 | 91.46 | 7.74 | 0.42 | 0.08 | 0.30 |
| PCMP_s.10S | Control | 2273 | W12 | 22882496 | 16778852 | 93.95 | 5.14 | 0.52 | 0.09 | 0.29 |
| PCMP_s.1S | Control | 2273 | W4 | 26261541 | 18587255 | 86.46 | 12.67 | 0.44 | 0.10 | 0.32 |
| PCMP_s.18S | Control | 2273 | W8 | 24566153 | 18228977 | 91.07 | 8.11 | 0.44 | 0.08 | 0.30 |
| PCMP_s.12S | Control | 2296 | W12 | 22473775 | 16457994 | 96.69 | 2.55 | 0.42 | 0.08 | 0.26 |
| PCMP_s.22S | Control | 2296 | W4 | 23615455 | 16868439 | 90.47 | 8.61 | 0.51 | 0.10 | 0.31 |
| PCMP_s.21S | Control | 2296 | W8 | 24005163 | 16267533 | 93.52 | 5.57 | 0.52 | 0.09 | 0.31 |
| PCMP_s.19S | Control | 2399 | W12 | 21397359 | 15991726 | 90.81 | 8.41 | 0.41 | 0.08 | 0.29 |
| PCMP_s.15S | Control | 2399 | W4 | 21032492 | 15522323 | 92.05 | 7.14 | 0.45 | 0.08 | 0.27 |
| PCMP_s.2S | Control | 2399 | W8 | 24842489 | 17819941 | 90.29 | 8.88 | 0.44 | 0.09 | 0.31 |

**Table S2:** Sequencing information for metatranscriptomics. Trt: treatment group; W: week 3-NOP: 3-nitrooxypropanol.

| Sample ID | Trt | Cow ID | Week | Number of  raw reads (paired) | Number of reads  after quality filtering (paired) | Bacteria  (%) | Archaea  (%) | Eukaryota  (%) | Viruses  (%) | Un-assigned  (%) |
| --- | --- | --- | --- | --- | --- | --- | --- | --- | --- | --- |
| CHMI_2775 | Control | 2270 | W4 | 42155350 | 32066991 | 88.20 | 9.65 | 0.46 | 1.48 | 0.21 |
| CHMI_2773 | Control | 2270 | W8 | 35653898 | 26062199 | 90.31 | 8.27 | 0.53 | 0.69 | 0.21 |
| CHMI_2780 | Control | 2270 | W12 | 62802356 | 45077200 | 86.89 | 9.94 | 0.82 | 2.07 | 0.28 |
| CHMI_2817 | Control | 2273 | W4 | 126024754 | 103203144 | 90.43 | 8.80 | 0.48 | 0.07 | 0.22 |
| CHMI_2782 | Control | 2273 | W8 | 31906910 | 7686587 | 88.43 | 10.53 | 0.46 | 0.34 | 0.23 |
| CHMI_2776_3002 | Control | 2273 | W12 | 52844351 | 39131948 | 90.98 | 8.33 | 0.39 | 0.07 | 0.23 |
| CHMI_0516_S01 | Control | 2296 | W4 | 39970560 | 36147653 | 88.46 | 8.54 | 0.61 | 2.17 | 0.22 |
| CHMI_2829_3004 | Control | 2296 | W8 | 64424881 | 51887507 | 88.83 | 10.39 | 0.42 | 0.15 | 0.22 |
| CHMI_2820 | Control | 2296 | W12 | 35136899 | 24827993 | 94.62 | 4.34 | 0.24 | 0.62 | 0.18 |
| CHMI_2779 | Control | 2399 | W4 | 62999150 | 47316460 | 90.71 | 8.61 | 0.40 | 0.06 | 0.22 |
| CHMI_2818_3000 | Control | 2399 | W8 | 76228190 | 42225825 | 88.42 | 10.61 | 0.30 | 0.46 | 0.22 |
| CHMI_2828_3003 | Control | 2399 | W12 | 64294113 | 49368115 | 87.70 | 11.37 | 0.38 | 0.33 | 0.22 |
| CHMI_2821 | 3-NOP | 2147 | W4 | 68765243 | 18595427 | 89.62 | 9.55 | 0.54 | 0.08 | 0.21 |
| CHMI_2822 | 3-NOP | 2147 | W8 | 30580539 | 4934812 | 89.62 | 9.15 | 0.43 | 0.57 | 0.23 |
| CHMI_2826_3001 | 3-NOP | 2147 | W12 | 64283659 | 46312949 | 89.21 | 9.77 | 0.51 | 0.19 | 0.32 |
| CHMI_2827 | 3-NOP | 2156 | W4 | 68057757 | 57876036 | 92.50 | 6.67 | 0.33 | 0.28 | 0.22 |
| CHMI_2783 | 3-NOP | 2156 | W8 | 46229863 | 17911754 | 90.28 | 8.20 | 0.57 | 0.68 | 0.27 |
| CHMI_2777 | 3-NOP | 2156 | W12 | 52727423 | 12977193 | 87.04 | 7.31 | 0.34 | 5.10 | 0.20 |
| CHMI_0516_S03 | 3-NOP | 2277 | W4 | 33739355 | 29999959 | 93.88 | 5.13 | 0.37 | 0.43 | 0.19 |
| CHMI_2774 | 3-NOP | 2277 | W8 | 36447185 | 13221617 | 94.88 | 4.63 | 0.17 | 0.13 | 0.19 |
| CHMI_2823 | 3-NOP | 2277 | W12 | 50932675 | 8293321 | 90.76 | 7.64 | 0.39 | 1.00 | 0.22 |
| CHMI_2781 | 3-NOP | 2410 | W4 | 33375178 | 7536015 | 88.61 | 10.18 | 0.51 | 0.46 | 0.23 |
| CHMI_2819 | 3-NOP | 2410 | W8 | 41398040 | 33664711 | 88.68 | 8.56 | 0.49 | 2.06 | 0.21 |
| CHMI_2772 | 3-NOP | 2410 | W12 | 44099505 | 33347545 | 94.15 | 5.23 | 0.35 | 0.06 | 0.21 |

**Table S3**: Relative abundance (%) of archaeal taxonomy in metagenomics. SEM: Standard error of mean; Trt: treatment group; W: week; 3-NOP: 3-nitrooxypropanol.

|  | **Control** | | | | **3-NOP** | | | | **Significance** | | | | |
| --- | --- | --- | --- | --- | --- | --- | --- | --- | --- | --- | --- | --- | --- |
| Genus | **W4** | **W8** | **W12** | **SEM** | **W4** | **W8** | **W12** | **SEM** | **Trt** | **W4**  **vs**  **W8** | **W4  vs  W12** | **Trt:**  **W4 vs W8** | **Trt:**  **W4 vs W12** |
| Methanobrevibacter | 82.36 | 75.27 | 70.38 | 2.28 | 73.89 | 66.96 | 69.74 | 2.39 | 0.02 | < 0.001 | < 0.001 | 0.55 | < 0.001 |
| Methanobacteriaceae | 3.76 | 5.84 | 5.52 | 0.33 | 4.35 | 6.61 | 6.39 | 0.35 | 0.02 | < 0.001 | < 0.001 | 0.74 | 0.34 |
| Methanosphaera | 3.17 | 4.81 | 5.26 | 0.48 | 4.41 | 5.63 | 5.31 | 0.46 | 0.13 | < 0.001 | < 0.001 | 0.006 | < 0.001 |
| Methanobacterium | 0.87 | 1.39 | 1.38 | 0.11 | 1.21 | 1.84 | 1.73 | 0.13 | 0.03 | < 0.001 | < 0.001 | 0.82 | 0.27 |
| Methanosarcina | 0.9 | 1.11 | 1.58 | 0.16 | 1.54 | 1.65 | 1.44 | 0.19 | 0.02 | < 0.001 | < 0.001 | 0.65 | < 0.001 |
| Thermoplasmata | 0.79 | 1.04 | 1.3 | 0.17 | 1.01 | 1.59 | 1.56 | 0.16 | 0.01 | < 0.001 | < 0.001 | 0.82 | < 0.001 |
| Thermococcus | 0.62 | 0.74 | 1.13 | 0.11 | 1.09 | 1.18 | 1.02 | 0.14 | 0.01 | < 0.001 | < 0.001 | 0.76 | < 0.001 |
| Methanococcus | 0.62 | 0.81 | 0.97 | 0.07 | 0.89 | 1.2 | 1.02 | 0.08 | 0.02 | < 0.001 | < 0.001 | 0.11 | < 0.001 |
| Methanobacteriales | 0.31 | 0.57 | 0.53 | 0.05 | 0.41 | 0.74 | 0.7 | 0.05 | 0.03 | < 0.001 | < 0.001 | 0.78 | 0.96 |
| Methanothermobacter | 0.18 | 0.41 | 0.48 | 0.06 | 0.33 | 0.62 | 0.56 | 0.06 | 0.02 | < 0.001 | < 0.001 | 0.76 | < 0.001 |
| Methanocaldococcus | 0.31 | 0.38 | 0.45 | 0.03 | 0.43 | 0.47 | 0.45 | 0.04 | 0.02 | < 0.001 | < 0.001 | 0.82 | 0.001 |
| Sulfolobales | 0 | 0.01 | 0 | 0 | 0.01 | 0.01 | 0.01 | 0 | 0.25 | 0.59 | 0.83 | 0.52 | 0.84 |
| Pyrodictium | 0.01 | 0.01 | 0.01 | 0 | 0.02 | 0.01 | 0.01 | 0 | 0.11 | 0.98 | 0.47 | 0.94 | 0.40 |
| Hyperthermus | 0.01 | 0.01 | 0.01 | 0 | 0.01 | 0.02 | 0.01 | 0 | 0.22 | 0.60 | 0.83 | 0.55 | 0.60 |
| Thermoplasmatales | 0.2 | 0.23 | 0.31 | 0.02 | 0.26 | 0.28 | 0.28 | 0.03 | 0.24 | < 0.001 | < 0.001 | 0.82 | 0.007 |
| Halobacteria | 0.01 | 0.01 | 0.02 | 0 | 0.02 | 0.02 | 0.03 | 0 | 0.22 | 0.71 | 0.02 | 0.83 | 0.60 |
| Methanosarcinaceae | 0.22 | 0.31 | 0.44 | 0.05 | 0.46 | 0.48 | 0.42 | 0.06 | < 0.001 | < 0.001 | < 0.001 | 0.04 | < 0.001 |
| Methanococcales | 0.02 | 0.02 | 0.03 | 0 | 0.03 | 0.02 | 0.02 | 0 | 0.31 | 0.98 | 0.16 | 0.82 | 0.06 |
| Thermogladius | 0.01 | 0.01 | 0.02 | 0 | 0.02 | 0.02 | 0.02 | 0 | 0.25 | 0.70 | 0.08 | 0.55 | 0.80 |
| Candidatus  Methanomethylophilus | 0.17 | 0.2 | 0.28 | 0.03 | 0.25 | 0.27 | 0.26 | 0.03 | 0.10 | < 0.001 | < 0.001 | 0.89 | 0.008 |
| Halobacteriales | 0.01 | 0.01 | 0.01 | 0 | 0.01 | 0.01 | 0.02 | 0 | 0.13 | 0.15 | 0.85 | 0.76 | 0.53 |
| Natronobacterium | 0.04 | 0.04 | 0.08 | 0.01 | 0.07 | 0.06 | 0.05 | 0.01 | 0.11 | 0.98 | < 0.001 | 0.94 | < 0.001 |
| Pyrococcus | 0.14 | 0.2 | 0.28 | 0.03 | 0.25 | 0.32 | 0.27 | 0.03 | 0.01 | < 0.001 | < 0.001 | 0.83 | < 0.001 |
| Thermococcaceae | 0.14 | 0.18 | 0.26 | 0.03 | 0.21 | 0.23 | 0.21 | 0.03 | 0.10 | < 0.001 | < 0.001 | 0.95 | < 0.001 |
| Candidatus  Nitrosomarinus | 0.03 | 0.03 | 0.05 | 0 | 0.04 | 0.05 | 0.04 | 0.01 | 0.40 | 0.93 | 0.02 | 0.50 | 0.17 |
| Methanococci | 0.01 | 0.01 | 0 | 0 | 0 | 0 | 0 | 0 | 0.04 | 0.33 | 0.03 | 0.82 | 1.00 |
| Pyrobaculum | 0.01 | 0.01 | 0.02 | 0 | 0.02 | 0.03 | 0.02 | 0 | 0.13 | 0.79 | 0.30 | 0.36 | 0.56 |
| Natrialbaceae | 0.03 | 0.03 | 0.05 | 0.01 | 0.05 | 0.05 | 0.05 | 0.01 | 0.06 | 0.88 | 0.005 | 0.83 | 0.19 |
| Methanothrix | 0.15 | 0.16 | 0.27 | 0.03 | 0.25 | 0.26 | 0.25 | 0.03 | < 0.001 | 0.01 | < 0.001 | 0.82 | < 0.001 |
| Methanococcoides | 0.16 | 0.22 | 0.29 | 0.03 | 0.26 | 0.36 | 0.29 | 0.04 | 0.03 | < 0.001 | < 0.001 | 0.55 | < 0.001 |
| Euryarchaeota | 0.25 | 0.27 | 0.36 | 0.02 | 0.3 | 0.36 | 0.3 | 0.02 | 0.20 | 0.04 | < 0.001 | 0.09 | 0.001 |
| Archaea | 0.14 | 0.22 | 0.25 | 0.02 | 0.22 | 0.31 | 0.28 | 0.02 | < 0.001 | < 0.001 | < 0.001 | 0.72 | 0.001 |
| Thermofilaceae | 0.01 | 0.01 | 0.01 | 0 | 0.01 | 0.01 | 0.01 | 0 | 0.34 | 0.38 | 0.16 | 0.82 | 0.42 |
| Halobacterium | 0.15 | 0.17 | 0.28 | 0.03 | 0.27 | 0.26 | 0.21 | 0.04 | 0.01 | < 0.001 | < 0.001 | 0.76 | < 0.001 |
| Methanohalophilus | 0.07 | 0.07 | 0.11 | 0.01 | 0.13 | 0.13 | 0.11 | 0.02 | 0.07 | 0.98 | < 0.001 | 0.42 | 0.04 |
| Methanoregula | 0.13 | 0.18 | 0.24 | 0.02 | 0.21 | 0.28 | 0.23 | 0.02 | < 0.001 | < 0.001 | < 0.001 | 0.83 | < 0.001 |
| Candidatus  Mancarchaeum | 0.01 | 0 | 0.01 | 0 | 0.01 | 0.01 | 0.01 | 0 | 0.69 | 0.20 | 0.44 | 0.05 | 0.68 |
| Palaeococcus | 0.02 | 0.02 | 0.04 | 0.01 | 0.04 | 0.03 | 0.02 | 0 | 0.05 | 0.98 | 0.03 | 0.82 | 0.005 |
| Pyrolobus | 0.01 | 0.01 | 0.02 | 0 | 0.02 | 0.01 | 0.01 | 0 | 0.57 | 0.36 | 0.16 | 0.76 | 0.13 |
| Methanotorris | 0.07 | 0.06 | 0.07 | 0 | 0.09 | 0.06 | 0.06 | 0.01 | 0.01 | 0.40 | 0.78 | 0.42 | 0.02 |
| Picrophilus | 0.01 | 0.01 | 0.03 | 0 | 0.03 | 0.03 | 0.02 | 0 | 0.03 | 0.98 | 0.003 | 0.88 | 0.02 |
| Haloferax | 0.13 | 0.17 | 0.25 | 0.02 | 0.22 | 0.24 | 0.24 | 0.03 | 0.01 | < 0.001 | < 0.001 | 0.72 | < 0.001 |
| Natrinema | 0.09 | 0.09 | 0.14 | 0.01 | 0.12 | 0.13 | 0.12 | 0.01 | 0.10 | 0.71 | < 0.001 | 0.42 | 0.02 |
| Methanothermus | 0.13 | 0.16 | 0.16 | 0.01 | 0.17 | 0.16 | 0.17 | 0.02 | 0.15 | 0.002 | 0.005 | 0.41 | 0.40 |
| Methanomicrobiaceae | 0.07 | 0.08 | 0.11 | 0.01 | 0.1 | 0.11 | 0.11 | 0.01 | 0.18 | 0.04 | < 0.001 | 0.82 | 0.27 |
| Ferroplasmaceae | 0.01 | 0.02 | 0.02 | 0 | 0.02 | 0.03 | 0.03 | 0 | 0.06 | 0.09 | 0.03 | 0.98 | 0.62 |
| Methanosalsum | 0.04 | 0.07 | 0.07 | 0.01 | 0.07 | 0.1 | 0.1 | 0.01 | 0.02 | < 0.001 | < 0.001 | 0.83 | 0.58 |
| Caldivirga | 0.01 | 0.01 | 0.01 | 0 | 0.02 | 0.01 | 0.01 | 0 | 0.16 | 0.83 | 0.006 | 0.71 | 0.10 |
| Haloarculaceae | 0.09 | 0.1 | 0.15 | 0.01 | 0.15 | 0.15 | 0.13 | 0.02 | 0.03 | 0.06 | < 0.001 | 1.00 | 0.001 |
| Candidatus  Korarchaeum | 0.01 | 0.01 | 0.02 | 0 | 0.02 | 0.02 | 0.01 | 0 | 0.14 | 0.43 | 0.18 | 0.94 | 0.10 |
| Halorubrum | 0.08 | 0.11 | 0.16 | 0.02 | 0.16 | 0.19 | 0.14 | 0.02 | 0.01 | < 0.001 | < 0.001 | 0.97 | < 0.001 |
| Haloterrigena | 0.11 | 0.14 | 0.22 | 0.03 | 0.18 | 0.2 | 0.16 | 0.02 | 0.05 | < 0.001 | < 0.001 | 0.74 | < 0.001 |
| Thermofilum | 0.03 | 0.02 | 0.05 | 0.01 | 0.06 | 0.04 | 0.04 | 0.01 | 0.01 | 0.81 | < 0.001 | 0.76 | < 0.001 |
| Methanococcaceae | 0.01 | 0.03 | 0.03 | 0 | 0.02 | 0.05 | 0.05 | 0.01 | 0.08 | < 0.001 | < 0.001 | 0.82 | 0.72 |
| Saccharolobus | 0.01 | 0.02 | 0.03 | 0 | 0.02 | 0.03 | 0.03 | 0 | 0.12 | 0.001 | < 0.001 | 0.98 | 0.58 |
| Desulfurococcales | 0 | 0 | 0 | 0 | 0 | 0 | 0 | 0 | 0.09 | 0.70 | 0.14 | 0.82 | 0.06 |
| Halopiger | 0.06 | 0.06 | 0.09 | 0.01 | 0.1 | 0.1 | 0.08 | 0.01 | 0.03 | 0.54 | < 0.001 | 0.74 | < 0.001 |
| Halalkalicoccus | 0.06 | 0.05 | 0.09 | 0.01 | 0.08 | 0.08 | 0.07 | 0.01 | 0.14 | 0.64 | < 0.001 | 0.55 | 0.004 |
| Haloarcula | 0.09 | 0.11 | 0.17 | 0.02 | 0.17 | 0.17 | 0.16 | 0.03 | 0.03 | 0.001 | < 0.001 | 0.82 | < 0.001 |
| Haloplanus | 0.02 | 0.02 | 0.04 | 0.01 | 0.03 | 0.04 | 0.04 | 0 | 0.20 | 0.21 | 0.002 | 0.36 | 0.81 |
| Methanothermococcus | 0.09 | 0.11 | 0.13 | 0.01 | 0.12 | 0.17 | 0.15 | 0.01 | 0.16 | 0.007 | < 0.001 | 0.27 | 0.54 |
| Natronomonas | 0.08 | 0.08 | 0.13 | 0.01 | 0.15 | 0.16 | 0.14 | 0.02 | 0.01 | 0.53 | < 0.001 | 0.40 | 0.005 |
| Halorhabdus | 0.08 | 0.1 | 0.17 | 0.02 | 0.17 | 0.19 | 0.14 | 0.02 | < 0.001 | 0.001 | < 0.001 | 0.82 | < 0.001 |
| Archaeoglobus | 0.08 | 0.1 | 0.16 | 0.02 | 0.14 | 0.16 | 0.14 | 0.02 | 0.06 | 0.004 | < 0.001 | 0.82 | 0.001 |
| Thermosphaera | 0.01 | 0.01 | 0.02 | 0 | 0.02 | 0.01 | 0.01 | 0 | 0.02 | 0.77 | < 0.001 | 0.82 | 0.001 |
| Candidatus  Nitrosopelagicus | 0.01 | 0.02 | 0.03 | 0 | 0.02 | 0.03 | 0.02 | 0 | 0.34 | 0.64 | < 0.001 | 0.41 | 0.17 |
| Methanocella | 0.12 | 0.14 | 0.2 | 0.02 | 0.18 | 0.2 | 0.19 | 0.02 | 0.08 | 0.01 | < 0.001 | 0.43 | 0.10 |
| Vulcanisaeta | 0.02 | 0.02 | 0.03 | 0 | 0.04 | 0.04 | 0.03 | 0 | < 0.001 | 0.57 | 0.001 | 0.91 | 0.01 |
| Thermoplasma | 0.03 | 0.04 | 0.05 | 0.01 | 0.04 | 0.04 | 0.04 | 0.01 | 0.25 | 0.06 | 0.03 | 0.83 | 0.27 |
| Ferroglobus | 0.03 | 0.04 | 0.05 | 0 | 0.03 | 0.05 | 0.04 | 0.01 | 0.55 | 0.002 | < 0.001 | 0.55 | 0.34 |
| Salinigranum | 0.07 | 0.1 | 0.13 | 0.01 | 0.11 | 0.14 | 0.11 | 0.01 | 0.03 | < 0.001 | < 0.001 | 0.97 | < 0.001 |
| Metallosphaera | 0.04 | 0.04 | 0.07 | 0.01 | 0.07 | 0.06 | 0.05 | 0.01 | 0.14 | 0.36 | < 0.001 | 0.83 | < 0.001 |
| Methanomassiliicoccus | 0.07 | 0.09 | 0.13 | 0.01 | 0.12 | 0.13 | 0.12 | 0.02 | 0.13 | 0.003 | < 0.001 | 0.83 | 0.002 |
| Desulfurococcaceae | 0 | 0 | 0 | 0 | 0 | 0 | 0 | 0 | 0.08 | 0.13 | 0.23 | 0.72 | 0.48 |
| Candidatus Nitrosotenuis | 0.03 | 0.03 | 0.04 | 0 | 0.05 | 0.04 | 0.04 | 0.01 | 0.05 | 0.59 | 0.001 | 0.72 | 0.03 |
| Methanosphaerula | 0.07 | 0.1 | 0.13 | 0.01 | 0.13 | 0.13 | 0.12 | 0.02 | 0.03 | < 0.001 | < 0.001 | 0.88 | 0.001 |
| Methanohalobium | 0.06 | 0.07 | 0.1 | 0.01 | 0.09 | 0.1 | 0.09 | 0.01 | 0.04 | 0.02 | < 0.001 | 0.97 | 0.05 |
| Haloferacales | 0.04 | 0.05 | 0.06 | 0.01 | 0.06 | 0.07 | 0.07 | 0.01 | 0.01 | 0.08 | 0.001 | 0.72 | 0.42 |
| Methanoculleus | 0.11 | 0.14 | 0.2 | 0.02 | 0.23 | 0.23 | 0.22 | 0.03 | < 0.001 | < 0.001 | < 0.001 | 0.40 | < 0.001 |
| Candidatus  Methanoplasma | 0.06 | 0.07 | 0.1 | 0.01 | 0.08 | 0.08 | 0.08 | 0.01 | 0.14 | 0.001 | < 0.001 | 0.82 | 0.10 |
| Sulfurisphaera | 0.03 | 0.03 | 0.05 | 0.01 | 0.04 | 0.05 | 0.04 | 0.01 | 0.18 | 0.53 | < 0.001 | 0.55 | 0.03 |
| Geoglobus | 0.05 | 0.07 | 0.08 | 0.01 | 0.08 | 0.09 | 0.08 | 0.01 | 0.16 | 0.008 | < 0.001 | 0.83 | 0.08 |
| Methanolobus | 0.08 | 0.09 | 0.13 | 0.01 | 0.11 | 0.14 | 0.14 | 0.02 | 0.26 | 0.04 | < 0.001 | 0.04 | 0.49 |
| Haloferacaceae | 0.07 | 0.09 | 0.14 | 0.01 | 0.12 | 0.14 | 0.11 | 0.01 | < 0.001 | 0.01 | < 0.001 | 0.82 | < 0.001 |
| Haloquadratum | 0.05 | 0.07 | 0.1 | 0.01 | 0.09 | 0.11 | 0.09 | 0.01 | 0.03 | < 0.001 | < 0.001 | 0.97 | 0.001 |
| Halorientalis | 0.04 | 0.05 | 0.07 | 0.01 | 0.08 | 0.08 | 0.06 | 0.01 | 0.01 | 0.14 | < 0.001 | 0.96 | < 0.001 |
| Caldisphaera | 0.04 | 0.04 | 0.06 | 0.01 | 0.05 | 0.08 | 0.08 | 0.01 | 0.10 | 0.27 | < 0.001 | 0.08 | 0.68 |
| Halanaeroarchaeum | 0.04 | 0.04 | 0.07 | 0.01 | 0.07 | 0.08 | 0.06 | 0.01 | 0.01 | 0.27 | < 0.001 | 0.82 | 0.001 |
| Ferroplasma | 0.01 | 0.02 | 0.03 | 0 | 0.02 | 0.03 | 0.03 | 0 | 0.15 | 0.02 | < 0.001 | 0.82 | 0.32 |
| Methanomethylovorans | 0.04 | 0.05 | 0.09 | 0.01 | 0.07 | 0.07 | 0.07 | 0.01 | 0.06 | 0.02 | < 0.001 | 0.98 | 0.001 |
| Cuniculiplasma | 0.02 | 0.04 | 0.05 | 0.01 | 0.05 | 0.08 | 0.06 | 0.01 | 0.01 | < 0.001 | < 0.001 | 0.97 | 0.16 |
| Natronolimnobius | 0.06 | 0.07 | 0.1 | 0.01 | 0.11 | 0.12 | 0.08 | 0.01 | 0.01 | 0.06 | < 0.001 | 0.82 | < 0.001 |
| Halobiforma | 0.06 | 0.08 | 0.1 | 0.01 | 0.1 | 0.12 | 0.09 | 0.01 | 0.02 | 0.003 | < 0.001 | 0.82 | 0.006 |
| Methanobacteria | 0.02 | 0.01 | 0.01 | 0 | 0.02 | 0.01 | 0.01 | 0 | 0.15 | 0.63 | 0.53 | 0.01 | 0.002 |
| Acidianus | 0.06 | 0.07 | 0.11 | 0.01 | 0.1 | 0.12 | 0.1 | 0.01 | 0.03 | 0.04 | < 0.001 | 0.74 | 0.001 |
| Methanospirillum | 0.05 | 0.06 | 0.07 | 0.01 | 0.07 | 0.09 | 0.07 | 0.01 | 0.02 | 0.06 | 0.01 | 0.76 | 0.09 |
| Candidatus  Nitrosocaldus | 0.01 | 0.02 | 0.02 | 0 | 0.02 | 0.02 | 0.02 | 0.01 | 0.14 | 0.08 | < 0.001 | 0.99 | 0.02 |
| Halomicrobium | 0.05 | 0.06 | 0.08 | 0.01 | 0.07 | 0.09 | 0.07 | 0.01 | 0.08 | 0.007 | < 0.001 | 0.72 | 0.10 |
| Halostagnicola | 0.04 | 0.05 | 0.07 | 0.01 | 0.09 | 0.09 | 0.07 | 0.01 | 0.01 | 0.04 | < 0.001 | 0.86 | 0.005 |
| Natronococcus | 0.05 | 0.06 | 0.09 | 0.01 | 0.08 | 0.09 | 0.08 | 0.01 | 0.01 | 0.01 | < 0.001 | 0.82 | 0.003 |
| Natrialbales | 0.03 | 0.04 | 0.07 | 0.01 | 0.07 | 0.06 | 0.06 | 0.01 | 0.04 | 0.01 | < 0.001 | 0.89 | 0.01 |
| Methanocorpusculum | 0.09 | 0.1 | 0.17 | 0.02 | 0.13 | 0.17 | 0.16 | 0.02 | 0.05 | 0.02 | < 0.001 | 0.36 | 0.02 |
| Halohasta | 0.04 | 0.05 | 0.07 | 0.01 | 0.08 | 0.09 | 0.08 | 0.01 | 0.01 | 0.003 | < 0.001 | 0.82 | 0.005 |
| Halobacteriaceae | 0.01 | 0.01 | 0.01 | 0 | 0.01 | 0.01 | 0.01 | 0 | 0.07 | 0.22 | 0.01 | 0.62 | 0.27 |
| Halopenitus | 0.03 | 0.04 | 0.08 | 0.01 | 0.07 | 0.08 | 0.06 | 0.01 | 0.02 | 0.03 | < 0.001 | 0.82 | < 0.001 |
| Acidilobus | 0.03 | 0.03 | 0.05 | 0 | 0.04 | 0.05 | 0.04 | 0.01 | 0.19 | 0.21 | < 0.001 | 0.40 | 0.18 |
| Thermococcales | 0.02 | 0.07 | 0.07 | 0.01 | 0.05 | 0.11 | 0.1 | 0.01 | 0.01 | < 0.001 | < 0.001 | 0.72 | 0.05 |
| Halodesulfurarchaeum | 0.04 | 0.04 | 0.06 | 0.01 | 0.07 | 0.06 | 0.05 | 0.01 | < 0.001 | 0.06 | < 0.001 | 0.82 | < 0.001 |
| Sulfolobus | 0.07 | 0.09 | 0.15 | 0.02 | 0.1 | 0.14 | 0.12 | 0.01 | 0.12 | 0.001 | < 0.001 | 0.40 | 0.002 |
| Aciduliprofundum | 0.04 | 0.05 | 0.08 | 0.01 | 0.06 | 0.1 | 0.08 | 0.01 | 0.08 | 0.001 | < 0.001 | 0.41 | 0.21 |
| Salinarchaeum | 0.05 | 0.06 | 0.11 | 0.02 | 0.11 | 0.1 | 0.08 | 0.02 | 0.01 | 0.002 | < 0.001 | 0.41 | < 0.001 |
| Natrialba | 0.09 | 0.11 | 0.18 | 0.02 | 0.22 | 0.16 | 0.13 | 0.04 | 0.01 | < 0.001 | < 0.001 | 0.01 | < 0.001 |
| Nitrososphaera | 0.03 | 0.05 | 0.08 | 0.01 | 0.07 | 0.08 | 0.07 | 0.01 | 0.01 | 0.002 | < 0.001 | 0.55 | < 0.001 |
| Methanosarcinales | 0 | 0.02 | 0.02 | 0 | 0.01 | 0.03 | 0.03 | 0 | < 0.001 | < 0.001 | < 0.001 | 0.55 | 0.17 |
| Staphylothermus | 0.03 | 0.06 | 0.08 | 0.01 | 0.08 | 0.09 | 0.09 | 0.01 | 0.01 | < 0.001 | < 0.001 | 0.36 | 0.001 |
| Methanomicrobia | 0.01 | 0.02 | 0.02 | 0 | 0.02 | 0.02 | 0.02 | 0 | 0.03 | 0.001 | 0.007 | 0.55 | 0.06 |
| Nitrosopumilus | 0.06 | 0.1 | 0.14 | 0.02 | 0.16 | 0.13 | 0.1 | 0.03 | 0.01 | < 0.001 | < 0.001 | 0.001 | < 0.001 |
| Methanocaldococcaceae | 0.03 | 0.05 | 0.05 | 0 | 0.05 | 0.06 | 0.05 | 0 | 0.03 | < 0.001 | < 0.001 | 0.36 | 0.03 |
| Thermoproteus | 0.02 | 0.03 | 0.07 | 0.01 | 0.06 | 0.05 | 0.04 | 0.01 | 0.01 | 0.003 | < 0.001 | 0.40 | < 0.001 |
| Methanolacinia | 0.04 | 0.06 | 0.1 | 0.01 | 0.11 | 0.09 | 0.07 | 0.02 | 0.01 | 0.001 | < 0.001 | 0.06 | < 0.001 |
| Desulfurococcus | 0.04 | 0.05 | 0.08 | 0.01 | 0.08 | 0.07 | 0.08 | 0.01 | 0.01 | 0.001 | < 0.001 | 0.08 | 0.001 |
| Halogeometricum | 0.03 | 0.05 | 0.08 | 0.01 | 0.07 | 0.06 | 0.05 | 0.01 | < 0.001 | < 0.001 | < 0.001 | 0.03 | < 0.001 |
| Ignicoccus | 0.02 | 0.02 | 0.05 | 0.01 | 0.06 | 0.03 | 0.02 | 0.01 | 0.02 | 0.06 | < 0.001 | 0.03 | < 0.001 |

**Table S4**: Relative abundance (%) of archaeal taxonomy in metatranscriptomics, SEM: Standard error of mean; Trt: treatment group; W: week; 3-NOP: 3-nitrooxypropanol.

|  | **Control** | | | | **3-NOP** | | | | | **Significance** | | | | | |
| --- | --- | --- | --- | --- | --- | --- | --- | --- | --- | --- | --- | --- | --- | --- | --- |
|  |  | | | |  | | | | |  | | | | | |
| Genus | **W4** | **W8** | **W12** | **SEM** | | **W4** | **W8** | **W12** | **SEM** | **Trt** | **W4  vs  W8** | **W4  vs  W12** | **Trt:** | **Trt:** |  |
|  |  |  |  |  |  |  |  |  |  |  |  |  | **W4 vs W8** | **W4 vs W12** |  |
| Methanobrevibacter | 78.6 | 72.19 | 69.46 | 1.66 | | 74.01 | 67.18 | 66.82 | 1.84 | 0.12 | < 0.001 | < 0.001 | < 0.001 | < 0.001 |  |
| Methanosphaera | 6.14 | 9.65 | 11.07 | 0.88 | | 7.32 | 10.68 | 7.92 | 0.81 | 0.76 | < 0.001 | < 0.001 | 0.002 | < 0.001 |  |
| Methanobacteriaceae | 4.24 | 6.98 | 6.86 | 0.46 | | 6.47 | 7.24 | 8.07 | 0.45 | < 0.001 | < 0.001 | < 0.001 | < 0.001 | < 0.001 |  |
| Thermoplasmata | 4.83 | 4.14 | 4.51 | 0.39 | | 5.13 | 6.82 | 8.03 | 0.8 | 0.17 | < 0.001 | < 0.001 | < 0.001 | < 0.001 |  |
| Methanobacterium | 0.32 | 0.93 | 0.93 | 0.11 | | 0.82 | 1.15 | 1.22 | 0.11 | < 0.001 | < 0.001 | < 0.001 | < 0.001 | < 0.001 |  |
| Halodesulfurarchaeum | 0.01 | 0.01 | 0.02 | 0 | | 0.01 | 0.02 | 0.02 | 0 | 0.71 | 0.71 | 0.92 | 0.91 | 0.85 |  |
| Acidilobus | 0.01 | 0.01 | 0.01 | 0 | | 0.01 | 0.01 | 0.01 | 0 | 0.94 | 0.16 | 1.00 | 0.82 | 1.00 |  |
| Thermococcus | 0.41 | 0.39 | 0.49 | 0.04 | | 0.45 | 0.46 | 0.53 | 0.04 | 0.17 | < 0.001 | 0.88 | < 0.001 | 0.004 |  |
| Sulfolobus | 0.03 | 0.03 | 0.03 | 0 | | 0.04 | 0.03 | 0.03 | 0 | 0.75 | 0.57 | 0.66 | 0.90 | 0.79 |  |
| Ferroplasma | 0 | 0 | 0 | 0 | | 0 | 0 | 0 | 0 | 0.33 | 0.94 | 0.50 | 0.88 | 0.98 |  |
| Haloquadratum | 0.02 | 0.02 | 0.03 | 0 | | 0.02 | 0.03 | 0.04 | 0 | 0.94 | 0.74 | 0.92 | 0.61 | < 0.001 |  |
| Euryarchaeota | 0.32 | 0.47 | 0.48 | 0.04 | | 0.39 | 0.45 | 0.6 | 0.05 | 0.56 | < 0.001 | < 0.001 | < 0.001 | < 0.001 |  |
| Hyperthermus | 0 | 0 | 0 | 0 | | 0 | 0 | 0 | 0 | 0.35 | 0.51 | 0.86 | 0.88 | 0.73 |  |
| Thermoplasma | 0.01 | 0.01 | 0.02 | 0 | | 0.01 | 0.02 | 0.02 | 0 | 0.75 | 0.53 | 0.37 | 0.91 | 0.54 |  |
| Acidilobaceae | 0 | 0 | 0 | 0 | | 0 | 0 | 0 | 0 | 0.41 | 0.94 | 0.60 | 0.97 | 0.27 |  |
| Aciduliprofundum | 0.02 | 0.02 | 0.02 | 0 | | 0.02 | 0.02 | 0.03 | 0 | 0.45 | 0.69 | 0.88 | 0.96 | 0.03 |  |
| Haloferacaceae | 0.05 | 0.05 | 0.06 | 0 | | 0.04 | 0.06 | 0.06 | 0.01 | 0.89 | 0.98 | 0.009 | 0.91 | 0.001 |  |
| Methanotorris | 0.03 | 0.02 | 0.02 | 0 | | 0.03 | 0.02 | 0.02 | 0 | 0.89 | 0.02 | 0.71 | 0.91 | 0.41 |  |
| Natronococcus | 0.02 | 0.02 | 0.02 | 0 | | 0.02 | 0.02 | 0.03 | 0 | 0.27 | 0.90 | 0.60 | 0.63 | 0.56 |  |
| Sulfurisphaera | 0.01 | 0.01 | 0.01 | 0 | | 0.01 | 0.02 | 0.01 | 0 | 0.90 | 0.75 | 0.97 | 0.13 | 0.17 |  |
| Halopenitus | 0.02 | 0.02 | 0.02 | 0 | | 0.02 | 0.02 | 0.03 | 0 | 0.07 | 0.97 | 0.58 | 0.26 | 0.95 |  |
| Candidatus  Mancarchaeum | 0 | 0 | 0 | 0 | | 0 | 0 | 0 | 0 | 0.81 | 0.11 | 0.72 | 0.53 | 0.77 |  |
| Nitrosopumilales | 0 | 0 | 0 | 0 | | 0 | 0 | 0 | 0 | 0.11 | 0.68 | 0.88 | 0.91 | 0.33 |  |
| Methanosarcinaceae | 0.09 | 0.12 | 0.15 | 0.01 | | 0.13 | 0.18 | 0.17 | 0.01 | 0.08 | 0.51 | < 0.001 | 0.82 | 0.56 |  |
| Methanospirillum | 0.02 | 0.02 | 0.03 | 0 | | 0.03 | 0.03 | 0.04 | 0 | 0.06 | 0.81 | 0.84 | 0.41 | 0.50 |  |
| Thermofilaceae | 0 | 0 | 0.01 | 0 | | 0.01 | 0 | 0.01 | 0 | 0.42 | 0.92 | 0.37 | 0.91 | 0.14 |  |
| Pyrodictium | 0 | 0 | 0 | 0 | | 0 | 0.01 | 0.01 | 0 | 0.16 | 0.82 | 0.38 | 0.82 | 0.55 |  |
| Methanobacteriales | 0.17 | 0.43 | 0.47 | 0.05 | | 0.4 | 0.5 | 0.58 | 0.05 | < 0.001 | < 0.001 | < 0.001 | < 0.001 | < 0.001 |  |
| Methanosarcina | 0.35 | 0.32 | 0.44 | 0.03 | | 0.42 | 0.45 | 0.41 | 0.02 | 0.16 | < 0.001 | < 0.001 | < 0.001 | 0.05 |  |
| Methanomicrobia | 0.02 | 0.02 | 0.02 | 0 | | 0.02 | 0.02 | 0.02 | 0 | 0.23 | 0.75 | 0.67 | 0.19 | 0.68 |  |
| Methanococcoides | 0.08 | 0.08 | 0.09 | 0.01 | | 0.11 | 0.11 | 0.12 | 0.01 | < 0.001 | 0.57 | 0.88 | < 0.001 | 0.55 |  |
| Candidatus  Nitrosocaldus | 0.01 | 0.01 | 0.01 | 0 | | 0.01 | 0.01 | 0.01 | 0 | 0.90 | 0.04 | 0.62 | 0.16 | 0.75 |  |
| Halorientalis | 0.03 | 0.02 | 0.03 | 0 | | 0.02 | 0.02 | 0.03 | 0 | 0.41 | 0.09 | 0.19 | 0.84 | 0.83 |  |
| Ferroplasmaceae | 0.01 | 0.01 | 0.01 | 0 | | 0.01 | 0.01 | 0.01 | 0 | 0.89 | 0.14 | 0.02 | 0.43 | 0.97 |  |
| Methanococcales | 0.01 | 0.01 | 0.01 | 0 | | 0.01 | 0.01 | 0 | 0 | 0.86 | 0.31 | 0.56 | 0.65 | 0.08 |  |
| Pyrolobus | 0 | 0 | 0 | 0 | | 0 | 0.01 | 0.01 | 0 | 0.76 | 0.13 | 1.00 | 0.27 | 0.31 |  |
| Natrialbales | 0.03 | 0.02 | 0.04 | 0.01 | | 0.03 | 0.03 | 0.04 | 0 | 0.89 | < 0.001 | 0.92 | 0.25 | 0.19 |  |
| Thermoplasmatales | 0.56 | 0.36 | 0.41 | 0.05 | | 0.24 | 0.2 | 0.43 | 0.04 | < 0.001 | < 0.001 | < 0.001 | 0.08 | < 0.001 |  |
| Methanothermus | 0.05 | 0.05 | 0.06 | 0 | | 0.06 | 0.06 | 0.04 | 0.01 | 0.99 | 0.98 | 0.001 | 0.04 | < 0.001 |  |
| Thermogladius | 0.01 | 0.01 | 0.01 | 0 | | 0.01 | 0.01 | 0.01 | 0 | 0.49 | 0.001 | 0.36 | 0.91 | 0.50 |  |
| Halorubraceae | 0 | 0 | 0 | 0 | | 0 | 0 | 0 | 0 | 0.39 | 0.29 | 0.21 | 0.52 | 0.89 |  |
| Ignicoccus | 0.01 | 0.01 | 0.01 | 0 | | 0.01 | 0.01 | 0.01 | 0 | 0.91 | 0.03 | 0.60 | 0.04 | 0.59 |  |
| Palaeococcus | 0.01 | 0.01 | 0.01 | 0 | | 0.02 | 0.01 | 0.01 | 0 | 0.16 | 0.82 | 0.77 | 0.40 | 0.002 |  |
| Methanosarcinales | 0.01 | 0.01 | 0.01 | 0 | | 0.01 | 0.01 | 0.01 | 0 | 0.70 | 0.22 | 0.50 | 0.004 | 0.73 |  |
| Natronobacterium | 0.02 | 0.02 | 0.02 | 0 | | 0.02 | 0.02 | 0.02 | 0 | 0.96 | 0.02 | 0.84 | 0.06 | 0.17 |  |
| Salinigranum | 0.03 | 0.03 | 0.03 | 0 | | 0.03 | 0.04 | 0.04 | 0.01 | 0.46 | 0.29 | 0.33 | 0.82 | 0.03 |  |
| Thermofilum | 0.03 | 0.03 | 0.04 | 0 | | 0.03 | 0.04 | 0.05 | 0 | 0.08 | 0.27 | 0.21 | 0.72 | 0.56 |  |
| Methanococcus | 0.24 | 0.31 | 0.36 | 0.02 | | 0.38 | 0.37 | 0.37 | 0.02 | < 0.001 | < 0.001 | < 0.001 | < 0.001 | < 0.001 |  |
| Methanosalsum | 0.02 | 0.02 | 0.02 | 0 | | 0.03 | 0.03 | 0.03 | 0 | 0.05 | 0.007 | 0.23 | 0.91 | 0.70 |  |
| Thermococcaceae | 0.14 | 0.13 | 0.14 | 0.01 | | 0.13 | 0.14 | 0.18 | 0.01 | 0.75 | 0.03 | 0.001 | 0.36 | < 0.001 |  |
| Methanosphaerula | 0.03 | 0.03 | 0.04 | 0 | | 0.04 | 0.04 | 0.05 | 0 | 0.24 | 0.99 | 0.001 | 0.18 | 0.37 |  |
| Methanoregula | 0.08 | 0.07 | 0.09 | 0 | | 0.09 | 0.1 | 0.1 | 0.01 | 0.11 | 0.01 | 0.02 | 0.89 | 0.44 |  |
| Thermosphaera | 0 | 0 | 0 | 0 | | 0 | 0 | 0 | 0 | 0.89 | 0.009 | 0.04 | 0.91 | 0.14 |  |
| Candidatus  Korarchaeum | 0 | 0 | 0 | 0 | | 0 | 0.01 | 0 | 0 | 0.37 | 0.56 | 0.37 | 0.51 | 0.15 |  |
| Halomicrobium | 0.02 | 0.02 | 0.02 | 0 | | 0.02 | 0.02 | 0.02 | 0 | 0.70 | 0.25 | 0.01 | 0.75 | 0.14 |  |
| Methanocella | 0.06 | 0.05 | 0.07 | 0.01 | | 0.05 | 0.07 | 0.07 | 0.01 | 0.44 | 0.24 | 0.04 | 0.52 | 0.35 |  |
| Methanolacinia | 0.02 | 0.02 | 0.02 | 0 | | 0.02 | 0.02 | 0.02 | 0 | 0.41 | 0.12 | 0.09 | 0.90 | 0.31 |  |
| Methanomicrobiales | 0 | 0 | 0 | 0 | | 0 | 0 | 0 | 0 | 0.39 | 0.55 | 0.71 | 0.13 | 0.13 |  |
| Thermoproteus | 0.01 | 0.01 | 0.01 | 0 | | 0.01 | 0.01 | 0.01 | 0 | 0.53 | 0.66 | 0.10 | 0.39 | 0.18 |  |
| Natrialba | 0.07 | 0.09 | 0.11 | 0.02 | | 0.07 | 0.11 | 0.12 | 0.01 | 0.96 | 0.13 | 0.21 | 0.01 | < 0.001 |  |
| Caldivirga | 0 | 0 | 0 | 0 | | 0 | 0 | 0 | 0 | 0.56 | 0.28 | 0.06 | 0.63 | 0.36 |  |
| Methanothrix | 0.07 | 0.06 | 0.07 | 0.01 | | 0.06 | 0.08 | 0.1 | 0.01 | 0.78 | < 0.001 | 0.31 | 0.33 | < 0.001 |  |
| Halogeometricum | 0.02 | 0.02 | 0.03 | 0.01 | | 0.02 | 0.03 | 0.02 | 0 | 0.75 | < 0.001 | 0.001 | 0.002 | 0.97 |  |
| Halobacteriales | 0 | 0.01 | 0.01 | 0 | | 0.01 | 0.01 | 0.01 | 0 | 0.24 | 0.85 | < 0.001 | 0.65 | 0.01 |  |
| Halohasta | 0.02 | 0.02 | 0.03 | 0 | | 0.02 | 0.03 | 0.03 | 0 | 0.88 | 0.005 | 0.42 | 0.23 | 0.05 |  |
| Natrinema | 0.07 | 0.05 | 0.07 | 0.01 | | 0.06 | 0.07 | 0.08 | 0.01 | 0.89 | < 0.001 | 0.30 | 0.13 | 0.001 |  |
| Candidatus  Methanomethylophilus | 0.4 | 0.26 | 0.33 | 0.04 | | 0.17 | 0.19 | 0.3 | 0.03 | < 0.001 | < 0.001 | < 0.001 | < 0.001 | < 0.001 |  |
| Halorubrum | 0.05 | 0.04 | 0.06 | 0.01 | | 0.04 | 0.06 | 0.06 | 0.01 | 0.89 | < 0.001 | 0.36 | 0.08 | 0.05 |  |
| Halorhabdus | 0.05 | 0.04 | 0.05 | 0.01 | | 0.05 | 0.04 | 0.06 | 0.01 | 0.76 | < 0.001 | 0.01 | 0.63 | 0.001 |  |
| Natrialbaceae | 0.03 | 0.03 | 0.04 | 0.01 | | 0.04 | 0.05 | 0.06 | 0.01 | 0.04 | 0.56 | 0.002 | 0.66 | 0.18 |  |
| Haloterrigena | 0.09 | 0.08 | 0.12 | 0.01 | | 0.09 | 0.13 | 0.11 | 0.01 | 0.26 | 0.02 | 0.08 | 0.51 | 0.18 |  |
| Methanococci | 0 | 0 | 0 | 0 | | 0 | 0 | 0 | 0 | 0.17 | 0.22 | 0.33 | 0.18 | 0.79 |  |
| Candidatus  Nitrosomarinus | 0.01 | 0.01 | 0.01 | 0 | | 0.01 | 0.01 | 0.01 | 0 | 0.11 | 0.55 | 0.38 | 0.54 | 0.03 |  |
| Haloferacales | 0.02 | 0.02 | 0.02 | 0 | | 0.02 | 0.02 | 0.03 | 0 | 0.19 | 0.30 | 0.13 | 0.21 | 0.68 |  |
| Halostagnicola | 0.02 | 0.01 | 0.02 | 0 | | 0.02 | 0.02 | 0.02 | 0 | 0.07 | 0.14 | 0.01 | 0.79 | 0.46 |  |
| Saccharolobus | 0 | 0.01 | 0 | 0 | | 0.01 | 0.01 | 0.01 | 0 | 0.30 | 0.008 | 0.25 | 0.13 | 0.86 |  |
| Halanaeroarchaeum | 0.02 | 0.02 | 0.02 | 0 | | 0.02 | 0.03 | 0.02 | 0 | 0.78 | < 0.001 | 0.004 | 0.06 | 0.56 |  |
| Haloarcula | 0.07 | 0.05 | 0.06 | 0.01 | | 0.07 | 0.08 | 0.09 | 0.01 | 0.17 | < 0.001 | < 0.001 | 0.91 | 0.001 |  |
| Methanohalobium | 0.02 | 0.03 | 0.03 | 0 | | 0.03 | 0.03 | 0.03 | 0 | 0.25 | 0.17 | 0.001 | 0.21 | 0.68 |  |
| Halalkalicoccus | 0.02 | 0.02 | 0.03 | 0 | | 0.02 | 0.04 | 0.04 | 0.01 | 0.71 | 0.001 | 0.30 | 0.24 | 0.06 |  |
| Candidatus  Nitrosopelagicus | 0.01 | 0.01 | 0.01 | 0 | | 0.01 | 0.02 | 0.01 | 0 | 0.44 | 0.001 | 0.15 | < 0.001 | 0.82 |  |
| Nitrososphaera | 0.02 | 0.02 | 0.02 | 0 | | 0.02 | 0.03 | 0.03 | 0 | 0.79 | 0.27 | 0.04 | 0.18 | 0.06 |  |
| Pyrobaculum | 0.01 | 0.01 | 0.01 | 0 | | 0.01 | 0.01 | 0.01 | 0 | 0.20 | < 0.001 | 0.002 | 0.75 | 0.45 |  |
| Methanomethylovorans | 0.02 | 0.02 | 0.03 | 0 | | 0.02 | 0.03 | 0.03 | 0 | 0.36 | 0.02 | 0.05 | 0.58 | 0.31 |  |
| Methanothermobacter | 0.13 | 0.32 | 0.11 | 0.08 | | 0.12 | 0.12 | 0.15 | 0.01 | 0.42 | < 0.001 | < 0.001 | < 0.001 | < 0.001 |  |
| Salinarchaeum | 0.02 | 0.02 | 0.04 | 0.01 | | 0.01 | 0.03 | 0.03 | 0.01 | 0.67 | 0.27 | 0.26 | 0.04 | 0.05 |  |
| Halobacteriaceae | 0 | 0 | 0 | 0 | | 0 | 0 | 0 | 0 | 0.24 | 0.53 | 0.59 | 0.06 | 0.02 |  |
| Archaeoglobus | 0.05 | 0.04 | 0.05 | 0 | | 0.05 | 0.06 | 0.07 | 0.01 | 0.71 | 0.005 | 0.10 | 0.28 | 0.01 |  |
| Staphylothermus | 0.02 | 0.02 | 0.02 | 0 | | 0.02 | 0.02 | 0.02 | 0 | 0.28 | 0.21 | 0.03 | 0.06 | 0.70 |  |
| Methanocaldococcus | 0.14 | 0.21 | 0.23 | 0.02 | | 0.24 | 0.26 | 0.28 | 0.02 | < 0.001 | < 0.001 | < 0.001 | < 0.001 | < 0.001 |  |
| Picrophilus | 0.01 | 0.01 | 0.01 | 0 | | 0.02 | 0.02 | 0.02 | 0 | 0.06 | 0.74 | 0.27 | 0.03 | 0.20 |  |
| Methanocorpusculum | 0.05 | 0.04 | 0.05 | 0 | | 0.04 | 0.06 | 0.08 | 0.01 | 0.95 | < 0.001 | 0.08 | 0.02 | < 0.001 |  |
| Metallosphaera | 0.04 | 0.04 | 0.06 | 0.01 | | 0.03 | 0.03 | 0.03 | 0 | 0.69 | < 0.001 | < 0.001 | 0.29 | 0.16 |  |
| Methanocaldococcaceae | 0.02 | 0.03 | 0.03 | 0 | | 0.03 | 0.03 | 0.03 | 0 | 0.56 | < 0.001 | < 0.001 | 0.58 | 0.06 |  |
| Vulcanisaeta | 0.01 | 0.01 | 0.01 | 0 | | 0.02 | 0.01 | 0.01 | 0 | < 0.001 | 0.02 | 0.86 | 0.31 | 0.12 |  |
| Halobacteria | 0.01 | 0 | 0 | 0 | | 0.01 | 0.01 | 0.01 | 0 | 0.67 | 0.07 | < 0.001 | 0.59 | < 0.001 |  |
| Halopiger | 0.03 | 0.02 | 0.04 | 0.01 | | 0.03 | 0.03 | 0.03 | 0 | 0.78 | 0.001 | 0.04 | 0.06 | 0.28 |  |
| Halobiforma | 0.03 | 0.02 | 0.02 | 0 | | 0.02 | 0.03 | 0.03 | 0 | 0.75 | 0.008 | 0.21 | 0.06 | 0.15 |  |
| Archaea | 0.11 | 0.16 | 0.27 | 0.06 | | 0.26 | 0.17 | 0.2 | 0.03 | 0.01 | 0.05 | < 0.001 | < 0.001 | < 0.001 |  |
| Haloarculaceae | 0.05 | 0.04 | 0.05 | 0 | | 0.05 | 0.06 | 0.06 | 0.01 | 0.20 | < 0.001 | 0.04 | 0.69 | 0.05 |  |
| Methanohalophilus | 0.04 | 0.04 | 0.04 | 0 | | 0.04 | 0.04 | 0.05 | 0 | 0.76 | 0.002 | 0.001 | 0.28 | < 0.001 |  |
| Methanolobus | 0.03 | 0.05 | 0.05 | 0.01 | | 0.04 | 0.06 | 0.07 | 0.01 | 0.09 | < 0.001 | < 0.001 | 0.40 | 0.46 |  |
| Natronomonas | 0.05 | 0.04 | 0.05 | 0 | | 0.05 | 0.06 | 0.06 | 0.01 | 0.12 | 0.01 | 0.08 | 0.63 | 0.03 |  |
| Geoglobus | 0.03 | 0.02 | 0.03 | 0 | | 0.03 | 0.03 | 0.03 | 0 | 0.94 | < 0.001 | 0.02 | 0.01 | 0.02 |  |
| Natronolimnobius | 0.06 | 0.04 | 0.06 | 0.01 | | 0.05 | 0.08 | 0.07 | 0.01 | 0.71 | < 0.001 | < 0.001 | 0.06 | < 0.001 |  |
| Nitrosopumilus | 0.02 | 0.03 | 0.04 | 0 | | 0.03 | 0.03 | 0.03 | 0 | 0.11 | 0.08 | < 0.001 | 0.67 | 0.05 |  |
| Methanoculleus | 0.08 | 0.07 | 0.08 | 0 | | 0.08 | 0.08 | 0.13 | 0.01 | 0.30 | 0.04 | 0.15 | 0.08 | < 0.001 |  |
| Methanomicrobiaceae | 0.04 | 0.04 | 0.04 | 0 | | 0.04 | 0.05 | 0.05 | 0.01 | 0.47 | 0.009 | 0.31 | 0.005 | < 0.001 |  |
| Caldisphaera | 0.01 | 0.02 | 0.03 | 0.01 | | 0.03 | 0.03 | 0.03 | 0 | 0.08 | 0.57 | < 0.001 | 0.24 | < 0.001 |  |
| Haloferax | 0.1 | 0.11 | 0.14 | 0.01 | | 0.11 | 0.13 | 0.17 | 0.02 | 0.11 | < 0.001 | < 0.001 | < 0.001 | 0.08 |  |
| Methanobacteria | 0.01 | 0.01 | 0.01 | 0 | | 0.01 | 0.01 | 0 | 0 | 0.10 | 0.30 | 0.30 | 0.02 | 0.19 |  |
| Haloplanus | 0.01 | 0.01 | 0.01 | 0 | | 0.01 | 0.01 | 0.01 | 0 | 0.42 | 0.02 | 0.19 | 0.04 | 0.20 |  |
| Sulfolobales | 0 | 0 | 0 | 0 | | 0.01 | 0.01 | 0 | 0 | 0.15 | 0.58 | 0.06 | 0.10 | 0.009 |  |
| Cuniculiplasma | 0.01 | 0.01 | 0.02 | 0 | | 0.02 | 0.02 | 0.03 | 0 | 0.03 | 0.47 | < 0.001 | 0.04 | 0.20 |  |
| Ferroglobus | 0.01 | 0.01 | 0.01 | 0 | | 0.01 | 0.02 | 0.02 | 0 | 0.45 | 0.13 | 0.08 | 0.10 | 0.001 |  |
| Pyrococcus | 0.07 | 0.09 | 0.1 | 0.01 | | 0.09 | 0.11 | 0.12 | 0.01 | 0.02 | < 0.001 | < 0.001 | < 0.001 | 0.16 |  |
| Halobacterium | 0.08 | 0.07 | 0.08 | 0.01 | | 0.09 | 0.1 | 0.11 | 0.01 | 0.01 | 0.05 | 0.008 | 0.02 | 0.12 |  |
| Thermococcales | 0.01 | 0.02 | 0.02 | 0 | | 0.02 | 0.02 | 0.03 | 0 | 0.03 | 0.03 | < 0.001 | 0.02 | 0.52 |  |
| Methanomassiliicoccus | 0.03 | 0.03 | 0.04 | 0 | | 0.02 | 0.03 | 0.03 | 0 | 0.05 | < 0.001 | 0.24 | 0.14 | 0.001 |  |
| Methanothermococcus | 0.03 | 0.03 | 0.04 | 0 | | 0.04 | 0.04 | 0.04 | 0 | < 0.001 | 0.13 | < 0.001 | 0.14 | < 0.001 |  |
| Candidatus  Methanoplasma | 0.08 | 0.06 | 0.07 | 0.01 | | 0.03 | 0.04 | 0.06 | 0.01 | < 0.001 | < 0.001 | < 0.001 | 0.10 | < 0.001 |  |
| Acidianus | 0.02 | 0.02 | 0.03 | 0 | | 0.03 | 0.03 | 0.03 | 0 | 0.06 | 0.01 | 0.001 | 0.15 | 0.001 |  |
| Desulfurococcus | 0.05 | 0.03 | 0.03 | 0.01 | | 0.02 | 0.03 | 0.04 | 0 | 0.10 | < 0.001 | < 0.001 | 0.04 | < 0.001 |  |
| Methanococcaceae | 0 | 0.01 | 0.01 | 0 | | 0.01 | 0.01 | 0.01 | 0 | 0.03 | 0.001 | < 0.001 | 0.04 | 0.14 |  |
| Candidatus  Nitrosotenuis | 0.02 | 0.01 | 0.01 | 0 | | 0.01 | 0.02 | 0.01 | 0 | 0.09 | < 0.001 | < 0.001 | < 0.001 | 0.007 |  |

**Table S5:** The 6 most abundant archaea (cpm; copies per million) contributing to steps 1-5 in the carbon dioxide (CO_2_)-hydrogen (H_2_) methanogenic pathway in the rumen of dairy cows supplemented with 3-nitrooxypropanol (3-NOP) compared to control cows at weeks 4, 8, and 12.

|  | **Metagenomics** | | | | | |
| --- | --- | --- | --- | --- | --- | --- |
|  | **Week 4** | | **Week 8** | | **Week 12** | |
| **Archaea populations in the rumen** | **Control** | **3-NOP** | **Control** | **3-NOP** | **Control** | **3-NOP** |
| *Methanobrevibacter ruminantium M1* | 772.2 | 576.7 | 674.2 | 394.6 | 448.2 | 462.9 |
| *Methanobrevibacter olleyae YLM1* | 581.5 | 441.2 | 498.8 | 292.5 | 338.8 | 341.6 |
| *Methanobrevibacter millerae SM9* | 319.9 | 233.2 | 387.6 | 306.5 | 292.9 | 386.6 |
| *Methanobrevibacter sp. YE315* | 350.8 | 220.5 | 315.4 | 180.2 | 247.8 | 226.6 |
| *Methanobrevibacter sp. AbM4* | 140.4 | 140.8 | 296.4 | 260.4 | 197 | 289.6 |
| *Methanobrevibacter smithii ATCC 35061* | 176.2 | 131.7 | 189.3 | 135.2 | 136.3 | 157.4 |
| Total genes contributed by 6 most abundant archaea | 2341 | 1744.1 | 2361.6 | 1569.4 | 1661 | 1864.6 |
| Total archaeal genes for steps 1-5 | 2480.3 | 1854.9 | 2510.8 | 1680.2 | 1777.8 | 1995.7 |
| % of gene contribution from 6 most abundant archaea | 94.4 | 94 | 94.1 | 93.4 | 93.4 | 93.4 |
|  | **Metatranscriptomics** | | | | | |
| *Methanobrevibacter ruminantium M1* | 1698.9 | 1553.3 | 1979.8 | 1392.8 | 1527.3 | 1359.8 |
| *Methanobrevibacter millerae SM9* | 1438.8 | 1254.1 | 1858 | 1365.8 | 1607.2 | 1449.6 |
| *Methanobrevibacter sp. YE315* | 1227 | 872.2 | 1350.4 | 877.5 | 1130.5 | 880.4 |
| *Methanobrevibacter olleyae YLM1* | 1200.2 | 1004.1 | 1266 | 883.1 | 974.8 | 827 |
| *Methanobrevibacter smithii ATCC 35061* | 570.3 | 468.9 | 654.9 | 476.1 | 537.5 | 463.3 |
| *Methanobrevibacter sp. AbM4* | 192 | 309.3 | 390.3 | 338.9 | 318.8 | 353.3 |
| Total transcripts contributed by 6 most abundant archaea | 6327.2 | 5462 | 7499.4 | 5334.1 | 6096.2 | 5333.4 |
| Total archaeal transcripts for steps 1-5 | 6749 | 5795.4 | 7982.8 | 5646.8 | 6480.1 | 5639.5 |
| % of transcript contribution from 6 most abundant archaea | 93.8 | 94.2 | 93.9 | 94.5 | 94.1 | 94.6 |

**Table S6**: The most abundant archaea (cpm; copies per million) utilizing methanol as a substrate for methanogenesis in the rumen of dairy cows supplemented with 3-NOP compared to control at weeks 4, 8, and 12.

|  | **Metagenomics** | | | | | |
| --- | --- | --- | --- | --- | --- | --- |
|  | **Week 4** | | **Week 8** | | **Week 12** | |
| **Archaea populations in the rumen** | **Control** | **3-NOP** | **Control** | **3-NOP** | **Control** | **3-NOP** |
| *Methanosphaera stadtmanae DSM 3091* | 45.1 | 34.8 | 48.5 | 36.9 | 42.3 | 40.7 |
| *Methanosphaera sp. BMS* | 38.4 | 30.7 | 44.7 | 34 | 33.5 | 38.9 |
| *Methanobrevibacter smithii ATCC 35061* | 8.4 | 1.9 | 8.9 | 4.8 | 5.5 | 4.5 |
| *Methanogenic archaeon ISO4-H5* | 3.6 | 2.1 | 2.2 | 2.1 | 2 | 2.7 |
| Total genes contributed by 4 most abundant archaea | 95.5 | 69.6 | 104.3 | 77.7 | 83.3 | 86.9 |
| Total methanol-utilizing archaeal genes | 97.9 | 69.9 | 105.9 | 78.5 | 84.9 | 87.5 |
| % of gene contribution from 4 most abundant archaea | 97.5 | 99.6 | 98.5 | 99.1 | 98.1 | 99.3 |
|  | **Metatranscriptomics** | | | | | |
| *Methanosphaera sp. BMS* | 605 | 534.9 | 901 | 641 | 846.5 | 453.2 |
| *Methanosphaera stadtmanae DSM 3091* | 404.8 | 363.8 | 606.3 | 439.8 | 569.5 | 326.1 |
| *Methanogenic archaeon ISO4-H5* | 67.7 | 57.1 | 55.1 | 76.7 | 56.7 | 50.2 |
| *Methanobrevibacter smithii ATCC 35061* | 1.1 | 0.3 | 1.4 | 1 | 1 | 0.5 |
| Total transcripts contributed by 4 most abundant archaea | 1078.6 | 956.1 | 1563.9 | 1158.5 | 1473.7 | 829.9 |
| Total methanol-utilizing archaeal transcripts | 1082.1 | 959.2 | 1566.7 | 1162.9 | 1477.1 | 832 |
| % of transcript contribution from 4 most abundant archaea | 99.7 | 99.7 | 99.8 | 99.6 | 99.8 | 99.7 |

**Table S7:** The most abundant archaea (cpm; copies per million) utilizing methylamines as a substrate for methanogenesis in the rumen of dairy cows supplemented with 3-NOP compared to control at weeks 4, 8, and 12.

|  | **Metagenomics** | | | | | | |
| --- | --- | --- | --- | --- | --- | --- | --- |
|  | **Week 4** | | **Week 8** | | | **Week 12** | |
| **Methylamine-utilizing archaea** | **Control** | **3-NOP** | **Control** | **3-NOP** | **Control** | | **3-NOP** |
| *Methanogenic archaeon ISO4-H5* | 2.9 | 0.9 | 4 | 1.9 | 3.5 | | 3.4 |
| *Thermoplasmatales archaeon BRNA1* | 0.1 | 0.1 | 0.4 | 0.2 | 0 | | 0.1 |
| *Candidatus Methanomethylophilus alvus Mx1201* | 0.1 | 0.1 | 0.1 | 0.1 | 0.1 | | 0.2 |
| Total genes contributed by 3 most abundant archaea | 3.1 | 1.1 | 4.4 | 2.2 | 3.5 | | 3.7 |
| Total methylamine-utilizing archaeal genes | 3.2 | 1.2 | 4.4 | 2.3 | 3.6 | | 3.7 |
| % of gene contribution from 3 most abundant archaea | 99 | 95.2 | 100 | 98.6 | 98.6 | | 100 |
|  | **Metatranscriptomics** | | | | | | |
| *Methanogenic archaeon ISO4-H5* | 360.7 | 243.5 | 294 | 272.4 | 266.2 | | 338 |
| *Thermoplasmatales archaeon BRNA1* | 14.8 | 9.7 | 12.2 | 9.8 | 10.1 | | 11.7 |
| *Candidatus Methanomethylophilus alvus Mx1201* | 10.6 | 7.4 | 9.8 | 7.3 | 8.3 | | 8.6 |
| *Candidatus Methanoplasma termitum MpT1* | 1.7 | 1.1 | 2.5 | 1.3 | 0.9 | | 1.3 |
| *Methanosalsum zhilinae DSM 4017* | 0.2 | 0.1 | 0.1 | 0.2 | 0.1 | | 0.7 |
| Total transcripts contributed by 5 most abundant archaea | 387.9 | 261.8 | 318.7 | 291.1 | 285.6 | | 360.2 |
| Total methylamine-utilizing archaeal transcripts | 389.3 | 262.8 | 320.5 | 292.5 | 287.3 | | 361.6 |
| % of transcript contribution from 5 most abundant archaea | 99.6 | 99.6 | 99.4 | 99.5 | 99.4 | | 99.6 |

**Table S8:** Transcripts (cpm; copies per million) coding for EC: 1.8.7.3 (HdrA, HdrB and HdrC) in cows supplemented with 3-nitrooxypropanol (3-NOP) compared to control cows at weeks 4, 8, and 12. ND = not detected.

|  | **Week 4** | | **Week 8** | | **Week 12** | |
| --- | --- | --- | --- | --- | --- | --- |
| Step 8a \|1.8.7.3 | **Control** | **3-NOP** | **Control** | **3-NOP** | **Control** | **3-NOP** |
| **K22480-A1 (heterodisulfide reductase subunit A1) - HdrA1** | **3.88** | **2.77** | **3.61** | **1.89** | **3.33** | **2.55** |
| K22480 \| *Methanobrevibacter sp. YE315* | 1.48 | 0.49 | 1.30 | 0.70 | 1.10 | 0.72 |
| K22480 \| *Methanobrevibacter millerae SM9* | 1.09 | 0.76 | 1.08 | 0.45 | 0.84 | 0.62 |
| K22480 \| *Methanosphaera sp. BMS* | 0.65 | 0.90 | 0.58 | 0.46 | 0.70 | 0.67 |
| K22480 \| *Methanobrevibacter smithii ATCC 35061* | 0.23 | 0.25 | 0.35 | 0.11 | 0.26 | 0.19 |
| K22480 \| *Methanosphaera stadtmanae DSM 3091* | 0.23 | 0.28 | 0.14 | 0.08 | 0.23 | 0.23 |
| K22480 \| *Methanobrevibacter olleyae YLM1* | 0.19 | 0.07 | 0.15 | 0.08 | 0.16 | 0.10 |
| **K22481-B1 (heterodisulfide reductase subunit B1) - HdrB1** | **3.49** | **3.85** | **3.28** | **1.91** | **3.27** | **1.42** |
| K22481 \| *Methanobrevibacter sp. YE315* | 1.62 | 1.90 | 1.65 | 0.49 | 1.67 | 0.48 |
| K22481 \| *Methanosphaera sp. BMS* | 1.27 | 1.22 | 0.94 | 1.01 | 1.03 | 0.63 |
| K22481 \| *Methanobrevibacter millerae SM9* | 0.40 | 0.48 | 0.33 | 0.17 | 0.40 | 0.19 |
| **K22482-C1 (heterodisulfide reductase subunit C1) - HdrC1** | **2.27** | **0.99** | **2.79** | **1.49** | **1.95** | **2.29** |
| K22482 \| *Methanobrevibacter sp. YE315* | 1.04 | 0.27 | 1.86 | 0.43 | 0.91 | 0.96 |
| K22482 \| *Methanosphaera stadtmanae DSM 3091* | 0.31 | 0.36 | 0.43 | 0.79 | 0.49 | 0.47 |
| K22482 \| *Methanosphaera sp. BMS* | 0.50 | 0.22 | 0.19 | 0.08 | 0.28 | 0.31 |
| K22482 \| *Methanobrevibacter millerae SM9* | 0.30 | 0.14 | 0.23 | 0.11 | 0.27 | 0.46 |
| K22482 \| *Methanobrevibacter olleyae YLM1* | 0.10 | 0.00 | 0.09 | 0.07 | 0.00 | 0.01 |
| K003388-A2 (heterodisulfide reductase subunit A2) - HdrA2 | ND | ND | ND | ND | ND | ND |
| K003389-B2 (heterodisulfide reductase subunit B2) - HdrB2 | ND | ND | ND | ND | ND | ND |
| K003390-C2 (heterodisulfide reductase subunit C2) - HdrC2 | ND | ND | ND | ND | ND | ND |

**Table S9**: Effect of 3-nitrooxypropanol (3-NOP) on bacterial taxonomical composition (relative abundance %) in DNA-based 16S rRNA sequencing. SEM: Standard error of mean; Trt: treatment group; W: week; 3-NOP: 3-nitrooxypropanol.

|  | **Control** | | | | | **3-nitrooxypropanol (3-NOP)** | | | | | **Significance** | | | | | |
| --- | --- | --- | --- | --- | --- | --- | --- | --- | --- | --- | --- | --- | --- | --- | --- | --- |
| Genus | **W4** | **W8** | **W12** | **SEM** | **W4** | | **W8** | **W12** | **SEM** | **Trt** | | **W4  vs W8** | **W4  vs  W12** | **Trt:  W4 vs  W8** | **Trt:  W4 vs  W12** |  |
| Actinobacteria Actinomycetaceae | 0 | 0 | 0 | 0.001 | 0 | | 0 | 0 | 0.001 | < 0.001 | | < 0.001 | < 0.001 | < 0.001 | < 0.001 |  |
| Actinobacteria Atopobium | 0.02 | 0.02 | 0.03 | 0.005 | 0.03 | | 0.02 | 0.03 | 0.003 | 0.58 | | 0.81 | 0.01 | 0.26 | 0.31 |  |
| Actinobacteria Coriobacteriaceae | 0.63 | 0.68 | 1.21 | 0.23 | 0.95 | | 0.68 | 0.65 | 0.142 | 0.22 | | 0.72 | < 0.001 | < 0.001 | < 0.001 |  |
| Actinobacteria Corynebacterium | 0.03 | 0.02 | 0.02 | 0.003 | 0.04 | | 0.01 | 0.11 | 0.022 | 0.59 | | 0.23 | 0.26 | < 0.001 | < 0.001 |  |
| Bacteria | 0 | 0 | 0 | 0.001 | 0 | | 0 | 0 | 0 | 0.57 | | 0.81 | 0.55 | 0.87 | 0.14 |  |
| Bacteroidetes [Paraprevotellaceae] | 0.45 | 0.38 | 0.45 | 0.017 | 0.42 | | 0.43 | 0.44 | 0.028 | 0.58 | | < 0.001 | 0.38 | 0.001 | 0.34 |  |
| Bacteroidetes Bacteroidales | 6.37 | 5.22 | 5.76 | 0.398 | 7.56 | | 5.7 | 6.7 | 0.426 | 0.19 | | < 0.001 | < 0.001 | < 0.001 | 0.84 |  |
| Bacteroidetes BF311 | 0.13 | 0.17 | 0.14 | 0.03 | 0.12 | | 0.1 | 0.21 | 0.023 | 0.91 | | < 0.001 | 0.05 | < 0.001 | < 0.001 |  |
| Bacteroidetes BS11 | 0.15 | 0.08 | 0.11 | 0.03 | 0.1 | | 0.07 | 0.07 | 0.023 | 0.92 | | 0.01 | 0.002 | 0.59 | 0.39 |  |
| Bacteroidetes CF231 | 0.52 | 0.56 | 0.59 | 0.044 | 0.58 | | 0.6 | 0.62 | 0.034 | 0.71 | | 0.81 | 0.34 | 0.55 | 0.54 |  |
| Bacteroidetes Prevotella | 26.18 | 26.09 | 26.71 | 1.467 | 26.63 | | 26.18 | 21.43 | 1.416 | 0.99 | | < 0.001 | 0.03 | 0.07 | < 0.001 |  |
| Bacteroidetes Prevotellaceae | 0.16 | 0.18 | 0.16 | 0.014 | 0.17 | | 0.19 | 0.18 | 0.011 | 0.90 | | 0.81 | 0.33 | 0.39 | 0.17 |  |
| Bacteroidetes RF16 | 0.3 | 0.24 | 0.42 | 0.044 | 0.56 | | 0.84 | 0.49 | 0.073 | < 0.001 | | < 0.001 | < 0.001 | < 0.001 | < 0.001 |  |
| Bacteroidetes S24-7 | 3.64 | 4.01 | 4.98 | 0.364 | 6.97 | | 5.07 | 6.26 | 0.418 | < 0.001 | | < 0.001 | < 0.001 | < 0.001 | < 0.001 |  |
| Bacteroidetes YRC22 | 0.55 | 0.55 | 0.61 | 0.035 | 0.61 | | 0.51 | 0.46 | 0.031 | 0.59 | | 0.11 | 0.11 | 0.08 | < 0.001 |  |
| Cyanobacteria Streptophyta | 0.1 | 0.09 | 0.03 | 0.019 | 0.03 | | 0.07 | 0.06 | 0.009 | < 0.001 | | < 0.001 | < 0.001 | < 0.001 | < 0.001 |  |
| Cyanobacteria YS2 | 0.09 | 0.11 | 0.22 | 0.043 | 0.19 | | 0.08 | 0.09 | 0.046 | 0.30 | | 0.34 | < 0.001 | < 0.001 | < 0.001 |  |
| Fibrobacteres Fibrobacter | 2.13 | 1.81 | 1.58 | 0.212 | 1.89 | | 1.12 | 1.77 | 0.214 | 0.69 | | < 0.001 | < 0.001 | < 0.001 | < 0.001 |  |
| Firmicutes [Eubacterium] | 0.05 | 0.04 | 0.04 | 0.01 | 0.07 | | 0.08 | 0.04 | 0.011 | 0.31 | | 0.16 | 0.89 | 0.03 | 0.003 |  |
| Firmicutes [Mogibacteriaceae] | 0.67 | 0.8 | 0.72 | 0.039 | 0.77 | | 0.69 | 0.84 | 0.033 | 0.13 | | 0.002 | 0.17 | < 0.001 | 0.53 |  |
| Firmicutes [Ruminococcus] | 0.02 | 0 | 0.01 | 0.004 | 0.01 | | 0.04 | 0.01 | 0.008 | 0.52 | | 0.001 | < 0.001 | < 0.001 | < 0.001 |  |
| Firmicutes Acidaminococcus | 0.04 | 0.06 | 0.05 | 0.009 | 0.04 | | 0.03 | 0.02 | 0.011 | 0.81 | | 0.007 | 0.02 | < 0.001 | < 0.001 |  |
| Firmicutes Anaerofustis | 0.02 | 0.02 | 0.01 | 0.003 | 0.01 | | 0.02 | 0.02 | 0.002 | 0.02 | | 0.56 | 0.16 | 0.007 | < 0.001 |  |
| Firmicutes Anaerostipes | 0.07 | 0.04 | 0.04 | 0.01 | 0.02 | | 0.04 | 0.03 | 0.004 | 0.001 | | < 0.001 | < 0.001 | < 0.001 | < 0.001 |  |
| Firmicutes Anaerovibrio | 0.04 | 0.02 | 0.02 | 0.005 | 0.04 | | 0.03 | 0.03 | 0.005 | 0.99 | | 0.01 | < 0.001 | 0.08 | 0.08 |  |
| Firmicutes Asteroleplasma | 0.01 | 0.01 | 0.01 | 0.001 | 0.01 | | 0.01 | 0 | 0.001 | 0.99 | | 0.93 | 0.97 | 0.33 | 0.002 |  |
| Firmicutes Blautia | 0.15 | 0.18 | 0.12 | 0.013 | 0.15 | | 0.14 | 0.15 | 0.016 | 0.87 | | 0.56 | < 0.001 | 0.10 | 0.01 |  |
| Firmicutes Bulleidia | 0.21 | 0.26 | 0.2 | 0.013 | 0.22 | | 0.32 | 0.19 | 0.023 | 0.93 | | 0.001 | 0.40 | 0.07 | 0.30 |  |
| Firmicutes Butyrivibrio | 8.31 | 9.38 | 8.24 | 0.853 | 9.24 | | 10.32 | 9.54 | 0.652 | 0.10 | | < 0.001 | < 0.001 | 0.80 | 0.28 |  |
| Firmicutes Christensenellaceae | 0.17 | 0.11 | 0.2 | 0.037 | 0.1 | | 0.1 | 0.13 | 0.013 | 0.39 | | 0.81 | < 0.001 | 0.87 | 0.82 |  |
| Firmicutes Clostridiaceae | 0.05 | 0.1 | 0.07 | 0.017 | 0.05 | | 0.04 | 0.07 | 0.009 | 0.81 | | < 0.001 | < 0.001 | < 0.001 | 0.68 |  |
| Firmicutes Clostridiales | 10.15 | 10.03 | 9.36 | 0.727 | 8.98 | | 8.71 | 11.13 | 0.711 | 0.57 | | 0.006 | < 0.001 | < 0.001 | < 0.001 |  |
| Firmicutes Clostridium | 0.33 | 0.26 | 0.28 | 0.023 | 0.29 | | 0.19 | 0.29 | 0.026 | 0.52 | | < 0.001 | 0.01 | 0.07 | 0.04 |  |
| Firmicutes Coprococcus | 1.4 | 1.14 | 1.07 | 0.084 | 1.1 | | 1.04 | 0.94 | 0.057 | < 0.001 | | < 0.001 | < 0.001 | < 0.001 | 0.002 |  |
| Firmicutes Dorea | 0 | 0 | 0 | 0.001 | 0 | | 0 | 0 | 0 | 0.99 | | 0.93 | 0.73 | 0.92 | 0.53 |  |
| Firmicutes Erysipelotrichaceae | 0.01 | 0.01 | 0 | 0.001 | 0 | | 0 | 0.01 | 0.002 | 0.59 | | 0.91 | 0.38 | 0.54 | 0.04 |  |
| Firmicutes L7A_E11 | 0.09 | 0.04 | 0.05 | 0.009 | 0.09 | | 0.06 | 0.07 | 0.013 | 0.93 | | < 0.001 | < 0.001 | 0.09 | 0.002 |  |
| Firmicutes Lachnospira | 0.03 | 0.04 | 0.04 | 0.004 | 0.03 | | 0.03 | 0.02 | 0.003 | 0.90 | | 0.47 | 0.02 | 0.53 | 0.01 |  |
| Firmicutes Lachnospiraceae | 10.16 | 13.46 | 9.79 | 0.714 | 8.38 | | 9.47 | 10.25 | 0.414 | 0.009 | | < 0.001 | < 0.001 | < 0.001 | < 0.001 |  |
| Firmicutes Lactobacillus | 0.02 | 0.01 | 0.01 | 0.004 | 0.01 | | 0.01 | 0.02 | 0.006 | 0.37 | | 0.65 | 0.001 | 0.80 | < 0.001 |  |
| Firmicutes Mogibacterium | 0 | 0 | 0.01 | 0.001 | 0 | | 0 | 0 | 0.001 | 0.39 | | 0.56 | 0.04 | 0.48 | 0.10 |  |
| Firmicutes Moryella | 0.42 | 0.44 | 0.51 | 0.031 | 0.57 | | 0.58 | 0.56 | 0.034 | 0.007 | | 0.53 | < 0.001 | 0.76 | < 0.001 |  |
| Firmicutes Oscillospira | 0.2 | 0.22 | 0.23 | 0.032 | 0.62 | | 0.29 | 0.22 | 0.153 | 0.28 | | 0.34 | 0.03 | < 0.001 | < 0.001 |  |
| Firmicutes p-75-a5 | 0.09 | 0.08 | 0.09 | 0.009 | 0.08 | | 0.08 | 0.09 | 0.008 | 0.76 | | 0.93 | 0.58 | 0.83 | 0.24 |  |
| Firmicutes Pseudobutyrivibrio | 0.28 | 0.33 | 0.18 | 0.053 | 0.13 | | 0.18 | 0.21 | 0.021 | 0.10 | | 0.20 | < 0.001 | < 0.001 | < 0.001 |  |
| Firmicutes RFN20 | 0.12 | 0.12 | 0.1 | 0.014 | 0.06 | | 0.07 | 0.12 | 0.012 | 0.004 | | 0.23 | 0.04 | 0.05 | < 0.001 |  |
| Firmicutes Roseburia | 0.01 | 0.01 | 0 | 0.001 | 0.01 | | 0.01 | 0 | 0.001 | 0.39 | | 0.54 | 0.02 | 0.39 | 0.52 |  |
| Firmicutes Ruminococcaceae | 5.43 | 4.81 | 4.53 | 0.376 | 4.36 | | 4.04 | 5.07 | 0.353 | 0.31 | | < 0.001 | < 0.001 | 0.33 | < 0.001 |  |
| Firmicutes Ruminococcus | 3.65 | 2.63 | 2.66 | 0.332 | 1.83 | | 4.11 | 5.14 | 0.845 | 0.001 | | < 0.001 | < 0.001 | < 0.001 | < 0.001 |  |
| Firmicutes Selenomonas | 0.03 | 0.02 | 0.03 | 0.005 | 0.03 | | 0.03 | 0.01 | 0.005 | 0.89 | | < 0.001 | 0.11 | < 0.001 | 0.13 |  |
| Firmicutes Sharpea | 0.8 | 0.32 | 0.92 | 0.295 | 0.19 | | 0.68 | 0.1 | 0.14 | 0.39 | | < 0.001 | < 0.001 | < 0.001 | < 0.001 |  |
| Firmicutes Shuttleworthia | 0.29 | 0.2 | 0.22 | 0.033 | 0.12 | | 0.2 | 0.12 | 0.044 | 0.03 | | < 0.001 | 0.002 | < 0.001 | 0.01 |  |
| Firmicutes Streptococcus | 0.01 | 0.01 | 0.01 | 0.002 | 0.01 | | 0.01 | 0.01 | 0.002 | 0.13 | | 1.00 | 0.06 | 0.87 | 0.02 |  |
| Firmicutes Succiniclasticum | 2.12 | 1.52 | 2.41 | 0.175 | 2.27 | | 2.06 | 1.54 | 0.19 | 0.33 | | < 0.001 | < 0.001 | < 0.001 | < 0.001 |  |
| Firmicutes Syntrophococcus | 0 | 0.01 | 0.01 | 0.001 | 0.01 | | 0.01 | 0 | 0.001 | 0.25 | | 0.47 | 0.22 | 0.26 | 0.04 |  |
| Firmicutes Veillonellaceae | 0.15 | 0.12 | 0.18 | 0.023 | 0.15 | | 0.2 | 0.13 | 0.036 | 0.91 | | < 0.001 | < 0.001 | < 0.001 | < 0.001 |  |
| Proteobacteria Alphaproteobacteria | 0.02 | 0.02 | 0.02 | 0.004 | 0.01 | | 0.02 | 0.03 | 0.005 | 0.39 | | 0.52 | 0.47 | 0.37 | 0.001 |  |
| Proteobacteria Desulfobulbus | 0 | 0 | 0 | 0 | 0 | | 0 | 0 | 0.001 | 0.89 | | 0.81 | 0.82 | 0.64 | 0.80 |  |
| Proteobacteria Desulfovibrio | 0 | 0.01 | 0.02 | 0.003 | 0.01 | | 0.01 | 0.01 | 0.002 | 0.04 | | 0.47 | < 0.001 | 0.26 | < 0.001 |  |
| Proteobacteria GMD14H09 | 0.04 | 0.04 | 0.03 | 0.009 | 0.03 | | 0.02 | 0.06 | 0.008 | 0.90 | | 0.38 | 0.41 | 0.02 | < 0.001 |  |
| Proteobacteria RF32 | 0.06 | 0.07 | 0.06 | 0.012 | 0.03 | | 0.08 | 0.05 | 0.011 | 0.31 | | 0.03 | 0.82 | 0.001 | 0.16 |  |
| Proteobacteria Rickettsiales | 0.01 | 0.01 | 0.01 | 0.003 | 0 | | 0 | 0.01 | 0.002 | 0.39 | | 0.77 | 0.22 | 0.94 | 0.01 |  |
| Proteobacteria Succinivibrio | 0 | 0 | 0 | 0 | 0 | | 0 | 0 | 0.001 | 0.76 | | 0.65 | 0.55 | 0.33 | 0.93 |  |
| Proteobacteria Succinivibrionaceae | 0.07 | 0.04 | 0.08 | 0.013 | 0.05 | | 0.1 | 0.02 | 0.015 | 0.28 | | < 0.001 | 0.37 | < 0.001 | < 0.001 |  |
| Proteobacteria Sutterella | 0.01 | 0.02 | 0.02 | 0.003 | 0.02 | | 0.02 | 0.03 | 0.003 | 0.71 | | 0.63 | 0.03 | 0.51 | 0.90 |  |
| Spirochaetes Sphaerochaeta | 0.03 | 0.02 | 0.03 | 0.005 | 0.01 | | 0.01 | 0.02 | 0.002 | 0.007 | | 0.58 | 0.26 | 0.76 | 0.04 |  |
| Spirochaetes Treponema | 1.06 | 0.95 | 0.89 | 0.124 | 0.75 | | 0.63 | 1.05 | 0.088 | 0.28 | | < 0.001 | < 0.001 | 0.96 | < 0.001 |  |
| SR1 | 0.3 | 0.24 | 0.27 | 0.04 | 0.1 | | 0.26 | 0.26 | 0.033 | < 0.001 | | < 0.001 | < 0.001 | < 0.001 | < 0.001 |  |
| Synergistetes Pyramidobacter | 0.02 | 0.01 | 0.02 | 0.003 | 0.02 | | 0.01 | 0.02 | 0.002 | 0.39 | | 0.007 | 0.09 | 0.18 | 0.03 |  |
| Tenericutes Anaeroplasma | 0.08 | 0.06 | 0.08 | 0.01 | 0.06 | | 0.07 | 0.09 | 0.009 | 0.46 | | 0.11 | 0.58 | 0.005 | < 0.001 |  |
| Tenericutes RF39 | 0.12 | 0.13 | 0.18 | 0.02 | 0.16 | | 0.18 | 0.15 | 0.018 | 0.28 | | 0.15 | < 0.001 | 0.87 | < 0.001 |  |
| TM7 F16 | 0.43 | 0.43 | 0.59 | 0.047 | 0.24 | | 0.34 | 0.43 | 0.033 | < 0.001 | | 0.51 | < 0.001 | < 0.001 | < 0.001 |  |
| Unassigned | 10.54 | 10.38 | 11.98 | 0.581 | 11.41 | | 12.23 | 10.75 | 0.593 | 0.61 | | < 0.001 | < 0.001 | < 0.001 | < 0.001 |  |
| WPS-2 | 0.05 | 0.09 | 0.07 | 0.018 | 0.03 | | 0.03 | 0.05 | 0.007 | 0.99 | | < 0.001 | 0.001 | < 0.001 | 0.12 |  |

**Table S10**: Effect of 3-nitrooxypropanol (3-NOP) on bacterial taxonomical composition (relative abundance %) in RNA-based 16S rRNA sequencing. SEM: Standard error of mean; Trt: treatment group; W: week

|  | **Control** | | | | **3-nitrooxypropanol (3-NOP)** | | | | | **Significance** | | | | |
| --- | --- | --- | --- | --- | --- | --- | --- | --- | --- | --- | --- | --- | --- | --- |
| Genus | **W4** | **W8** | **W12** | **SEM** | **W4** | **W8** | **W12** | **SEM** | **Trt** | | **W4  vs  W8** | **W4 vs  W12** | **Trt:  W4  vs W8** | **Trt:  W4  vs W12** |
| Actinobacteria Actinomycetaceae | 0 | 0 | 0 | 0 | 0 | 0 | 0 | 0.001 | 0.43 | | 0.85 | 0.15 | 0.49 | 0.60 |
| Actinobacteria Atopobium | 0.02 | 0.02 | 0.02 | 0.003 | 0.02 | 0.02 | 0.03 | 0.002 | 0.73 | | 0.63 | 0.93 | 0.81 | 0.12 |
| Actinobacteria Coriobacteriaceae | 0.17 | 0.11 | 0.15 | 0.014 | 0.13 | 0.14 | 0.15 | 0.014 | 0.13 | | < 0.001 | 0.05 | < 0.001 | 0.001 |
| Actinobacteria Corynebacterium | 0.01 | 0.01 | 0.01 | 0.002 | 0.01 | 0.01 | 0.05 | 0.013 | 0.49 | | 0.79 | 0.37 | 0.09 | < 0.001 |
| Bacteria | 0 | 0 | 0 | 0 | 0 | 0 | 0 | 0.001 | 0.51 | | 0.86 | 0.93 | 0.29 | 0.27 |
| Bacteroidetes [Paraprevotellaceae] | 0.35 | 0.43 | 0.35 | 0.04 | 0.31 | 0.32 | 0.33 | 0.037 | 0.80 | | < 0.001 | 0.26 | < 0.001 | 0.91 |
| Bacteroidetes Bacteroidales | 2.91 | 2.14 | 2.4 | 0.2 | 3.51 | 2.31 | 2.57 | 0.25 | 0.25 | | < 0.001 | < 0.001 | < 0.001 | < 0.001 |
| Bacteroidetes BF311 | 0.06 | 0.04 | 0.04 | 0.008 | 0.05 | 0.04 | 0.05 | 0.006 | 0.81 | | < 0.001 | < 0.001 | 0.53 | 0.001 |
| Bacteroidetes BS11 | 0.02 | 0.01 | 0.01 | 0.004 | 0 | 0 | 0.01 | 0.001 | 0.29 | | 0.002 | < 0.001 | 0.09 | < 0.001 |
| Bacteroidetes CF231 | 0.22 | 0.18 | 0.24 | 0.017 | 0.3 | 0.24 | 0.27 | 0.028 | 0.14 | | < 0.001 | 0.20 | 0.58 | < 0.001 |
| Bacteroidetes Prevotella | 14.43 | 14.61 | 17.37 | 0.97 | 15.48 | 16.31 | 13.27 | 0.969 | 0.80 | | 0.85 | < 0.001 | < 0.001 | < 0.001 |
| Bacteroidetes Prevotellaceae | 0.15 | 0.13 | 0.14 | 0.012 | 0.18 | 0.17 | 0.14 | 0.011 | 0.27 | | 0.03 | 0.11 | 0.88 | 0.05 |
| Bacteroidetes RF16 | 0.5 | 0.3 | 0.45 | 0.062 | 0.86 | 1 | 0.49 | 0.088 | 0.03 | | < 0.001 | < 0.001 | < 0.001 | < 0.001 |
| Bacteroidetes S24-7 | 0.72 | 0.69 | 0.74 | 0.082 | 0.99 | 0.72 | 0.86 | 0.084 | 0.14 | | 0.04 | 0.51 | < 0.001 | < 0.001 |
| Bacteroidetes YRC22 | 0.28 | 0.26 | 0.26 | 0.024 | 0.27 | 0.2 | 0.24 | 0.02 | 0.83 | | 0.08 | 0.08 | 0.001 | 0.34 |
| Cyanobacteria Streptophyta | 0 | 0.01 | 0 | 0.001 | 0 | 0 | 0 | 0.001 | 0.83 | | 0.14 | 0.74 | 0.25 | 0.62 |
| Cyanobacteria YS2 | 0.09 | 0.13 | 0.23 | 0.023 | 0.17 | 0.14 | 0.11 | 0.022 | 0.09 | | < 0.001 | < 0.001 | < 0.001 | < 0.001 |
| Fibrobacteres Fibrobacter | 4.14 | 3.54 | 5.06 | 0.41 | 4.76 | 2.77 | 4.72 | 0.385 | 0.14 | | < 0.001 | < 0.001 | < 0.001 | < 0.001 |
| Firmicutes [Eubacterium] | 0.02 | 0.02 | 0.03 | 0.005 | 0.05 | 0.04 | 0.03 | 0.007 | 0.16 | | 0.85 | 0.51 | 0.99 | 0.001 |
| Firmicutes [Mogibacteriaceae] | 0.7 | 0.65 | 0.79 | 0.042 | 0.78 | 0.67 | 0.83 | 0.044 | 0.37 | | 0.02 | < 0.001 | 0.01 | 0.06 |
| Firmicutes [Ruminococcus] | 0.01 | 0.01 | 0.01 | 0.003 | 0.01 | 0.02 | 0.01 | 0.004 | 0.83 | | 0.52 | 0.002 | < 0.001 | 0.001 |
| Firmicutes Acidaminococcus | 0.04 | 0.06 | 0.09 | 0.018 | 0.08 | 0.03 | 0.03 | 0.019 | 0.83 | | < 0.001 | < 0.001 | < 0.001 | < 0.001 |
| Firmicutes Anaerofustis | 0.01 | 0.01 | 0.01 | 0.001 | 0.01 | 0 | 0.01 | 0.001 | 0.64 | | 0.73 | 0.90 | 0.60 | 0.74 |
| Firmicutes Anaerostipes | 0.29 | 0.23 | 0.17 | 0.04 | 0.39 | 0.11 | 0.26 | 0.114 | 0.83 | | < 0.001 | < 0.001 | < 0.001 | 0.16 |
| Firmicutes Anaerovibrio | 0.02 | 0.01 | 0.01 | 0.002 | 0.01 | 0.02 | 0.01 | 0.002 | 0.32 | | 0.07 | 0.06 | 0.01 | 0.27 |
| Firmicutes Asteroleplasma | 0 | 0 | 0.01 | 0.001 | 0.01 | 0.01 | 0 | 0.001 | 0.04 | | 0.86 | 0.05 | 0.35 | 0.001 |
| Firmicutes Blautia | 0.31 | 0.22 | 0.18 | 0.023 | 0.26 | 0.2 | 0.22 | 0.028 | 0.51 | | < 0.001 | < 0.001 | 0.09 | < 0.001 |
| Firmicutes Bulleidia | 0.16 | 0.13 | 0.14 | 0.007 | 0.15 | 0.18 | 0.13 | 0.01 | 0.51 | | 0.002 | 0.06 | < 0.001 | 0.81 |
| Firmicutes Butyrivibrio | 10.91 | 10.09 | 10.18 | 0.462 | 11.51 | 11.51 | 12.5 | 0.508 | 0.51 | | < 0.001 | < 0.001 | < 0.001 | < 0.001 |
| Firmicutes Christensenellaceae | 0.14 | 0.09 | 0.14 | 0.026 | 0.07 | 0.06 | 0.07 | 0.01 | 0.14 | | < 0.001 | 0.74 | 0.01 | 0.97 |
| Firmicutes Clostridiaceae | 0.05 | 0.09 | 0.19 | 0.05 | 0.43 | 0.13 | 0.19 | 0.07 | < 0.001 | | < 0.001 | < 0.001 | < 0.001 | < 0.001 |
| Firmicutes Clostridiales | 9.38 | 8.77 | 7.67 | 0.445 | 9.36 | 7.94 | 8.86 | 0.651 | 0.98 | | < 0.001 | < 0.001 | < 0.001 | < 0.001 |
| Firmicutes Clostridium | 0.23 | 0.22 | 0.37 | 0.052 | 0.33 | 0.19 | 0.45 | 0.079 | 0.10 | | 0.96 | < 0.001 | < 0.001 | < 0.001 |
| Firmicutes Coprococcus | 1.9 | 1.35 | 1.28 | 0.115 | 1.51 | 1.99 | 2.62 | 0.358 | 0.17 | | < 0.001 | < 0.001 | < 0.001 | < 0.001 |
| Firmicutes Dorea | 0.01 | 0.01 | 0.01 | 0.001 | 0.01 | 0.01 | 0.01 | 0.001 | 0.91 | | 0.85 | 0.63 | 0.92 | 0.94 |
| Firmicutes Erysipelotrichaceae | 0.01 | 0 | 0 | 0.001 | 0 | 0 | 0 | 0.001 | 0.16 | | 0.002 | 0.001 | 0.11 | 0.001 |
| Firmicutes L7A_E11 | 0.02 | 0.01 | 0.02 | 0.002 | 0.02 | 0.02 | 0.02 | 0.003 | 0.66 | | < 0.001 | 0.20 | 0.26 | 0.98 |
| Firmicutes Lachnospira | 0.03 | 0.06 | 0.05 | 0.006 | 0.03 | 0.04 | 0.03 | 0.005 | 0.83 | | < 0.001 | < 0.001 | < 0.001 | 0.001 |
| Firmicutes Lachnospiraceae | 14.87 | 18.9 | 13.26 | 1.117 | 11.38 | 12.72 | 15.48 | 0.749 | 0.01 | | < 0.001 | < 0.001 | < 0.001 | < 0.001 |
| Firmicutes Lactobacillus | 0.01 | 0 | 0.01 | 0.002 | 0.01 | 0 | 0.01 | 0.001 | 0.64 | | 0.21 | < 0.001 | 0.99 | 0.10 |
| Firmicutes Mogibacterium | 0.01 | 0.01 | 0.01 | 0.001 | 0.01 | 0.01 | 0.01 | 0.001 | 0.91 | | 0.52 | 0.93 | 0.99 | 0.42 |
| Firmicutes Moryella | 0.44 | 0.43 | 0.43 | 0.026 | 0.57 | 0.47 | 0.51 | 0.023 | < 0.001 | | 0.85 | 0.58 | < 0.001 | 0.05 |
| Firmicutes Oscillospira | 0.26 | 0.26 | 0.27 | 0.019 | 0.49 | 0.28 | 0.36 | 0.085 | 0.18 | | 0.86 | 0.29 | < 0.001 | < 0.001 |
| Firmicutes p-75-a5 | 0.04 | 0.04 | 0.03 | 0.003 | 0.03 | 0.03 | 0.03 | 0.005 | 0.74 | | 0.85 | 0.16 | 0.45 | 0.42 |
| Firmicutes Pseudobutyrivibrio | 0.19 | 0.17 | 0.11 | 0.022 | 0.11 | 0.1 | 0.13 | 0.013 | 0.10 | | 0.06 | < 0.001 | 0.99 | < 0.001 |
| Firmicutes RFN20 | 0.07 | 0.05 | 0.06 | 0.005 | 0.06 | 0.04 | 0.06 | 0.006 | 0.61 | | < 0.001 | 0.02 | 0.99 | 0.18 |
| Firmicutes Roseburia | 0.02 | 0.04 | 0.01 | 0.007 | 0.1 | 0.09 | 0.04 | 0.029 | 0.16 | | < 0.001 | < 0.001 | < 0.001 | 0.64 |
| Firmicutes Ruminococcaceae | 5.82 | 5.26 | 4.71 | 0.297 | 4.47 | 4.41 | 4.9 | 0.281 | 0.12 | | < 0.001 | < 0.001 | < 0.001 | < 0.001 |
| Firmicutes Ruminococcus | 10.78 | 9.55 | 8.28 | 0.732 | 6.9 | 10.75 | 11.59 | 1.432 | 0.03 | | < 0.001 | < 0.001 | < 0.001 | < 0.001 |
| Firmicutes Selenomonas | 0.15 | 0.08 | 0.09 | 0.016 | 0.11 | 0.12 | 0.09 | 0.012 | 0.10 | | < 0.001 | < 0.001 | < 0.001 | 0.01 |
| Firmicutes Sharpea | 0.17 | 0.09 | 0.27 | 0.089 | 0.01 | 0.1 | 0 | 0.025 | 0.11 | | < 0.001 | < 0.001 | < 0.001 | < 0.001 |
| Firmicutes Shuttleworthia | 0.3 | 0.3 | 0.33 | 0.032 | 0.2 | 0.27 | 0.2 | 0.044 | 0.25 | | 0.85 | 0.009 | < 0.001 | 0.23 |
| Firmicutes Streptococcus | 0.01 | 0 | 0.01 | 0.001 | 0.01 | 0 | 0.01 | 0.001 | 0.64 | | 0.29 | 0.48 | 0.49 | 0.68 |
| Firmicutes Succiniclasticum | 0.29 | 0.27 | 0.38 | 0.029 | 0.31 | 0.29 | 0.21 | 0.028 | 0.64 | | 0.20 | < 0.001 | 0.77 | < 0.001 |
| Firmicutes Syntrophococcus | 0.01 | 0.01 | 0 | 0.001 | 0 | 0 | 0.01 | 0.001 | 0.69 | | 0.97 | 0.07 | 0.99 | 0.03 |
| Firmicutes Veillonellaceae | 0.19 | 0.21 | 0.34 | 0.059 | 0.25 | 0.33 | 0.22 | 0.078 | 0.83 | | 0.01 | < 0.001 | 0.03 | < 0.001 |
| Proteobacteria Alphaproteobacteria | 0.02 | 0.02 | 0.02 | 0.003 | 0.02 | 0.02 | 0.04 | 0.006 | 0.64 | | 0.79 | 0.41 | 0.25 | < 0.001 |
| Proteobacteria Desulfobulbus | 0.01 | 0.01 | 0.01 | 0.001 | 0 | 0.01 | 0.01 | 0.001 | 0.24 | | 0.68 | 0.74 | 0.05 | 0.21 |
| Proteobacteria Desulfovibrio | 0.04 | 0.03 | 0.06 | 0.005 | 0.07 | 0.04 | 0.04 | 0.009 | < 0.001 | | 0.05 | < 0.001 | 0.008 | < 0.001 |
| Proteobacteria GMD14H09 | 0.12 | 0.18 | 0.13 | 0.035 | 0.18 | 0.1 | 0.18 | 0.03 | 0.77 | | < 0.001 | 0.02 | < 0.001 | 0.10 |
| Proteobacteria RF32 | 0.05 | 0.06 | 0.04 | 0.006 | 0.04 | 0.06 | 0.03 | 0.007 | 0.61 | | 0.003 | 0.79 | 0.15 | 0.07 |
| Proteobacteria Rickettsiales | 0.01 | 0.01 | 0.01 | 0.003 | 0.01 | 0.01 | 0.01 | 0.002 | 0.83 | | 0.08 | < 0.001 | 0.99 | 0.02 |
| Proteobacteria Succinivibrio | 0.01 | 0.01 | 0.02 | 0.002 | 0.01 | 0.01 | 0.01 | 0.001 | 0.91 | | 0.004 | 0.38 | 0.05 | 0.11 |
| Proteobacteria Succinivibrionaceae | 4.74 | 5.8 | 8.1 | 1.007 | 8.02 | 7.5 | 1.97 | 1.695 | 0.44 | | < 0.001 | < 0.001 | < 0.001 | < 0.001 |
| Proteobacteria Sutterella | 0 | 0.01 | 0.01 | 0.001 | 0.01 | 0 | 0.01 | 0.001 | 0.51 | | 0.19 | 0.39 | 0.12 | 0.74 |
| Spirochaetes Sphaerochaeta | 0.02 | 0.02 | 0.02 | 0.002 | 0.01 | 0.01 | 0.02 | 0.002 | 0.002 | | 0.56 | 0.30 | 0.49 | 0.001 |
| Spirochaetes Treponema | 0.68 | 0.71 | 0.82 | 0.069 | 0.59 | 0.47 | 0.82 | 0.06 | 0.60 | | 0.10 | < 0.001 | < 0.001 | 0.001 |
| SR1 | 0.07 | 0.07 | 0.09 | 0.013 | 0.05 | 0.08 | 0.08 | 0.01 | 0.32 | | 0.16 | < 0.001 | 0.004 | 0.11 |
| Synergistetes Pyramidobacter | 0.05 | 0.03 | 0.04 | 0.004 | 0.03 | 0.03 | 0.03 | 0.004 | 0.21 | | 0.01 | 0.10 | 0.16 | 0.21 |
| Tenericutes Anaeroplasma | 0.08 | 0.05 | 0.07 | 0.01 | 0.06 | 0.08 | 0.09 | 0.008 | 0.64 | | < 0.001 | 0.05 | < 0.001 | < 0.001 |
| Tenericutes RF39 | 0.04 | 0.04 | 0.04 | 0.003 | 0.05 | 0.04 | 0.04 | 0.007 | 0.10 | | 0.89 | 0.11 | 0.26 | < 0.001 |
| TM7 F16 | 0.04 | 0.05 | 0.09 | 0.007 | 0.06 | 0.05 | 0.05 | 0.006 | 0.40 | | 0.08 | < 0.001 | 0.05 | < 0.001 |
| Unassigned | 11.84 | 12.11 | 12.43 | 0.21 | 13.22 | 12.61 | 12.77 | 0.304 | 0.05 | | < 0.001 | < 0.001 | < 0.001 | < 0.001 |
| WPS-2 | 0.13 | 0.27 | 0.19 | 0.049 | 0.14 | 0.11 | 0.17 | 0.029 | 0.82 | | < 0.001 | < 0.001 | < 0.001 | < 0.001 |

**Table S11**: Effect of 3-nitrooxypropanol (3-NOP) on bacterial taxonomical composition (relative abundance %) in metagenomics. SEM: Standard error of mean; Trt: treatment group; W: week.

|  | **Control** | | | | **3-nitrooxypropanol (3-NOP)** | | | | | **Significance** | | | | |
| --- | --- | --- | --- | --- | --- | --- | --- | --- | --- | --- | --- | --- | --- | --- |
|  | **W4** | **W8** | **W12** | **SEM** | **W4** | **W8** | **W12** | **SEM** | Trt | | **W4  vs  W8** | **W4 vs  W12** | **Trt:  W4  vs W8** | **Trt:  W4  vs W8** |
| Actinobacteria Actinobacteria | 0.83 | 0.84 | 0.79 | 0.023 | 0.8 | 0.8 | 0.87 | 0.022 | 0.32 | | 0.23 | < 0.001 | 0.92 | < 0.001 |
| Actinobacteria Actinomyces | 0.16 | 0.16 | 0.15 | 0.005 | 0.15 | 0.16 | 0.16 | 0.004 | 0.21 | | 0.05 | 0.15 | 0.70 | < 0.001 |
| Actinobacteria Adlercreutzia | 0.13 | 0.13 | 0.12 | 0.006 | 0.13 | 0.13 | 0.13 | 0.003 | 0.17 | | 0.41 | < 0.001 | 0.51 | < 0.001 |
| Actinobacteria Amycolatopsis | 0.09 | 0.09 | 0.09 | 0.002 | 0.09 | 0.08 | 0.09 | 0.003 | 0.44 | | 0.03 | 0.001 | 0.32 | 0.001 |
| Actinobacteria Arthrobacter | 0.15 | 0.15 | 0.15 | 0.005 | 0.15 | 0.15 | 0.15 | 0.003 | 0.38 | | 0.50 | 0.008 | 0.31 | < 0.001 |
| Actinobacteria Atopobiaceae | 0.09 | 0.1 | 0.13 | 0.014 | 0.1 | 0.1 | 0.1 | 0.009 | 0.57 | | < 0.001 | < 0.001 | < 0.001 | < 0.001 |
| Actinobacteria Bifidobacteriaceae | 0.25 | 0.23 | 0.19 | 0.032 | 0.18 | 0.2 | 0.18 | 0.017 | 0.15 | | < 0.001 | < 0.001 | < 0.001 | < 0.001 |
| Actinobacteria Bifidobacterium | 2.24 | 2.13 | 1.45 | 0.471 | 2.1 | 1.93 | 1.4 | 0.323 | 0.82 | | < 0.001 | < 0.001 | 0.001 | < 0.001 |
| Actinobacteria Collinsella | 0.18 | 0.18 | 0.18 | 0.009 | 0.17 | 0.17 | 0.18 | 0.006 | 0.37 | | 0.67 | 0.41 | 0.97 | 0.1 |
| Actinobacteria Coriobacteriaceae | 0.1 | 0.1 | 0.09 | 0.005 | 0.09 | 0.09 | 0.09 | 0.004 | 0.04 | | 0.20 | < 0.001 | 0.07 | < 0.001 |
| Actinobacteria Corynebacteriaceae | 0.09 | 0.09 | 0.09 | 0.002 | 0.1 | 0.08 | 0.12 | 0.009 | 0.71 | | 0.35 | 0.01 | 0.001 | < 0.001 |
| Actinobacteria Corynebacterium | 0.56 | 0.6 | 0.56 | 0.012 | 0.63 | 0.55 | 0.81 | 0.057 | 0.24 | | < 0.001 | 0.31 | < 0.001 | < 0.001 |
| Actinobacteria Denitrobacterium | 0.17 | 0.18 | 0.19 | 0.013 | 0.15 | 0.16 | 0.16 | 0.006 | 0.22 | | < 0.001 | < 0.001 | 0.05 | 0.001 |
| Actinobacteria Eggerthella | 0.2 | 0.2 | 0.19 | 0.008 | 0.18 | 0.18 | 0.19 | 0.006 | 0.007 | | 0.86 | < 0.001 | 0.54 | < 0.001 |
| Actinobacteria Eggerthellales | 0.11 | 0.11 | 0.1 | 0.004 | 0.1 | 0.11 | 0.11 | 0.004 | < 0.001 | | 0.13 | < 0.001 | 0.004 | < 0.001 |
| Actinobacteria Gordonia | 0.07 | 0.08 | 0.07 | 0.002 | 0.08 | 0.07 | 0.07 | 0.004 | 0.63 | | 0.67 | 0.001 | < 0.001 | 0.6 |
| Actinobacteria Gordonibacter | 0.25 | 0.25 | 0.23 | 0.011 | 0.23 | 0.23 | 0.24 | 0.007 | 0.003 | | 0.15 | < 0.001 | 0.43 | < 0.001 |
| Actinobacteria Microbacterium | 0.13 | 0.12 | 0.11 | 0.005 | 0.12 | 0.11 | 0.13 | 0.004 | 0.26 | | 0.21 | < 0.001 | 0.84 | < 0.001 |
| Actinobacteria Mycobacterium | 0.14 | 0.13 | 0.13 | 0.003 | 0.14 | 0.13 | 0.14 | 0.003 | 0.85 | | 0.58 | 0.001 | 0.95 | < 0.001 |
| Actinobacteria Mycolicibacterium | 0.11 | 0.11 | 0.11 | 0.003 | 0.11 | 0.11 | 0.11 | 0.004 | 0.85 | | 0.05 | < 0.001 | 0.55 | 0.002 |
| Actinobacteria Nocardia | 0.09 | 0.09 | 0.09 | 0.003 | 0.1 | 0.09 | 0.1 | 0.003 | 0.92 | | 0.27 | 0.001 | 0.80 | 0.001 |
| Actinobacteria Olsenella | 1.1 | 1.16 | 1.4 | 0.137 | 1.25 | 1.15 | 1.16 | 0.114 | 0.38 | | < 0.001 | < 0.001 | < 0.001 | < 0.001 |
| Actinobacteria Rhodococcus | 0.14 | 0.14 | 0.13 | 0.004 | 0.14 | 0.13 | 0.14 | 0.005 | 0.96 | | 0.21 | < 0.001 | 0.06 | < 0.001 |
| Actinobacteria Slackia | 0.3 | 0.29 | 0.29 | 0.012 | 0.28 | 0.28 | 0.3 | 0.009 | 0.07 | | 0.16 | 0.009 | 0.07 | < 0.001 |
| Actinobacteria Streptomyces | 0.84 | 0.83 | 0.79 | 0.023 | 0.82 | 0.79 | 0.85 | 0.022 | 0.38 | | 0.001 | < 0.001 | 0.92 | < 0.001 |
| Actinobacteria Streptomycetaceae | 0.3 | 0.3 | 0.28 | 0.01 | 0.29 | 0.29 | 0.31 | 0.009 | 0.42 | | 0.17 | < 0.001 | 0.82 | < 0.001 |
| Bacteria | 4.29 | 4.45 | 4.32 | 0.094 | 4.17 | 4.41 | 4.57 | 0.092 | 0.40 | | < 0.001 | 0.004 | < 0.001 | < 0.001 |
| Bacteroidetes Alistipes | 0.27 | 0.26 | 0.28 | 0.006 | 0.31 | 0.28 | 0.27 | 0.007 | < 0.001 | | 0.20 | 0.001 | < 0.001 | < 0.001 |
| Bacteroidetes Bacteroidaceae | 0.44 | 0.43 | 0.48 | 0.016 | 0.51 | 0.47 | 0.45 | 0.014 | < 0.001 | | 0.67 | < 0.001 | < 0.001 | < 0.001 |
| Bacteroidetes Bacteroidales | 0.11 | 0.1 | 0.12 | 0.003 | 0.13 | 0.11 | 0.12 | 0.003 | 0.003 | | 0.06 | 0.002 | 0.06 | < 0.001 |
| Bacteroidetes Bacteroides | 2.55 | 2.52 | 2.75 | 0.092 | 3 | 2.75 | 2.54 | 0.094 | 0.002 | | 0.66 | < 0.001 | < 0.001 | < 0.001 |
| Bacteroidetes Bacteroidia | 0.76 | 0.79 | 0.89 | 0.038 | 0.91 | 0.87 | 0.81 | 0.025 | < 0.001 | | < 0.001 | < 0.001 | < 0.001 | < 0.001 |
| Bacteroidetes Barnesiella | 0.4 | 0.4 | 0.44 | 0.014 | 0.49 | 0.44 | 0.43 | 0.014 | < 0.001 | | 0.09 | < 0.001 | < 0.001 | < 0.001 |
| Bacteroidetes Capnocytophaga | 0.18 | 0.17 | 0.18 | 0.005 | 0.2 | 0.18 | 0.17 | 0.006 | < 0.001 | | 0.15 | 0.008 | < 0.001 | < 0.001 |
| Bacteroidetes Chryseobacterium | 0.39 | 0.38 | 0.41 | 0.013 | 0.44 | 0.39 | 0.39 | 0.011 | < 0.001 | | 0.98 | < 0.001 | < 0.001 | < 0.001 |
| Bacteroidetes Flavobacteriaceae | 0.15 | 0.14 | 0.16 | 0.003 | 0.16 | 0.16 | 0.15 | 0.003 | 0.08 | | < 0.001 | 0.43 | 0.64 | < 0.001 |
| Bacteroidetes Flavobacteriales | 0.09 | 0.09 | 0.09 | 0.002 | 0.09 | 0.09 | 0.09 | 0.001 | 0.007 | | 0.50 | 0.001 | 0.58 | 0.001 |
| Bacteroidetes Flavobacterium | 0.24 | 0.23 | 0.25 | 0.004 | 0.26 | 0.25 | 0.24 | 0.005 | 0.12 | | 0.001 | 0.06 | 0.31 | < 0.001 |
| Bacteroidetes Hymenobacter | 0.41 | 0.4 | 0.42 | 0.009 | 0.46 | 0.42 | 0.42 | 0.008 | < 0.001 | | 0.05 | 0.02 | < 0.001 | < 0.001 |
| Bacteroidetes Hymenobacteraceae | 0.1 | 0.1 | 0.11 | 0.002 | 0.11 | 0.11 | 0.1 | 0.002 | < 0.001 | | 0.66 | < 0.001 | 0.04 | < 0.001 |
| Bacteroidetes Muribaculum | 0.32 | 0.33 | 0.36 | 0.012 | 0.42 | 0.37 | 0.36 | 0.013 | < 0.001 | | 0.001 | < 0.001 | < 0.001 | < 0.001 |
| Bacteroidetes Odoribacter | 0.08 | 0.08 | 0.09 | 0.002 | 0.1 | 0.09 | 0.08 | 0.003 | 0.004 | | 0.76 | 0.004 | 0.01 | < 0.001 |
| Bacteroidetes Pedobacter | 0.09 | 0.09 | 0.09 | 0.002 | 0.1 | 0.09 | 0.09 | 0.003 | 0.26 | | 0.08 | 0.27 | 1.00 | 0.001 |
| Bacteroidetes Petrimonas | 0.15 | 0.15 | 0.17 | 0.004 | 0.18 | 0.17 | 0.16 | 0.004 | < 0.001 | | 0.20 | < 0.001 | < 0.001 | < 0.001 |
| Bacteroidetes Pontibacter | 0.13 | 0.12 | 0.13 | 0.002 | 0.14 | 0.13 | 0.13 | 0.003 | < 0.001 | | 0.02 | 0.06 | 0.18 | < 0.001 |
| Bacteroidetes Porphyromonas | 0.28 | 0.27 | 0.31 | 0.009 | 0.34 | 0.3 | 0.3 | 0.008 | < 0.001 | | 0.98 | < 0.001 | < 0.001 | < 0.001 |
| Bacteroidetes Prevotella | 16.2 | 16.14 | 18.56 | 0.826 | 17.54 | 16.52 | 14.66 | 0.81 | 0.24 | | < 0.001 | < 0.001 | < 0.001 | < 0.001 |
| Bacteroidetes Prevotellaceae | 0.89 | 0.96 | 1.11 | 0.069 | 1.11 | 1.05 | 0.88 | 0.059 | 0.01 | | < 0.001 | < 0.001 | < 0.001 | < 0.001 |
| Bacteroidetes Proteiniphilum | 0.09 | 0.09 | 0.1 | 0.003 | 0.11 | 0.1 | 0.09 | 0.003 | < 0.001 | | 0.82 | 0.01 | < 0.001 | < 0.001 |
| Bacteroidetes Rufibacter | 0.11 | 0.1 | 0.11 | 0.003 | 0.12 | 0.11 | 0.11 | 0.003 | < 0.001 | | 0.35 | 0.004 | 0.05 | < 0.001 |
| Bacteroidetes Spirosoma | 0.13 | 0.13 | 0.13 | 0.003 | 0.14 | 0.13 | 0.13 | 0.002 | < 0.001 | | 0.94 | 0.005 | 0.02 | < 0.001 |
| Bacteroidetes Tannerella | 0.36 | 0.35 | 0.38 | 0.011 | 0.42 | 0.39 | 0.37 | 0.009 | < 0.001 | | 0.98 | < 0.001 | < 0.001 | < 0.001 |
| Bacteroidetes Tannerellaceae | 0.14 | 0.14 | 0.16 | 0.006 | 0.17 | 0.16 | 0.15 | 0.005 | < 0.001 | | 0.50 | < 0.001 | < 0.001 | < 0.001 |
| Chloroflexi Dehalococcoides | 0.06 | 0.07 | 0.08 | 0.006 | 0.08 | 0.08 | 0.07 | 0.006 | 0.06 | | < 0.001 | < 0.001 | < 0.001 | < 0.001 |
| Cyanobacteria Synechococcus | 0.12 | 0.11 | 0.12 | 0.002 | 0.12 | 0.12 | 0.12 | 0.002 | 0.25 | | 0.31 | 0.67 | 0.78 | 0.4 |
| Deinococcus-Thermus Deinococcus | 0.21 | 0.2 | 0.2 | 0.005 | 0.2 | 0.2 | 0.21 | 0.005 | 0.26 | | 0.21 | < 0.001 | 0.20 | < 0.001 |
| Fibrobacteres Fibrobacter | 3.16 | 3.07 | 3.27 | 0.346 | 3.32 | 2.41 | 3.64 | 0.309 | 0.97 | | < 0.001 | 0.36 | < 0.001 | < 0.001 |
| Firmicutes Acidaminococcus | 0.29 | 0.33 | 0.37 | 0.023 | 0.35 | 0.41 | 0.32 | 0.037 | 0.25 | | < 0.001 | < 0.001 | 0.39 | < 0.001 |
| Firmicutes Anaerostipes | 0.21 | 0.23 | 0.21 | 0.007 | 0.21 | 0.22 | 0.23 | 0.007 | 0.46 | | < 0.001 | 0.86 | 0.78 | < 0.001 |
| Firmicutes Bacillaceae | 0.19 | 0.19 | 0.2 | 0.005 | 0.19 | 0.2 | 0.21 | 0.006 | 0.85 | | 0.67 | 0.85 | 0.58 | 0.0 |
| Firmicutes Bacilli | 0.19 | 0.2 | 0.19 | 0.004 | 0.17 | 0.21 | 0.21 | 0.007 | 0.02 | | 0.15 | 0.83 | < 0.001 | < 0.001 |
| Firmicutes Bacillus | 0.69 | 0.68 | 0.65 | 0.011 | 0.63 | 0.68 | 0.7 | 0.014 | < 0.001 | | < 0.001 | < 0.001 | < 0.001 | < 0.001 |
| Firmicutes Blautia | 0.68 | 0.71 | 0.64 | 0.021 | 0.7 | 0.68 | 0.69 | 0.014 | 0.97 | | 0.002 | < 0.001 | 0.001 | < 0.001 |
| Firmicutes Butyrivibrio | 3.6 | 3.33 | 2.72 | 0.332 | 2.72 | 2.85 | 3.18 | 0.209 | 0.006 | | < 0.001 | < 0.001 | < 0.001 | < 0.001 |
| Firmicutes Christensenella | 0.15 | 0.15 | 0.14 | 0.006 | 0.14 | 0.15 | 0.16 | 0.005 | 0.02 | | 0.67 | < 0.001 | 0.005 | < 0.001 |
| Firmicutes Clostridia | 1.38 | 1.47 | 1.33 | 0.045 | 1.27 | 1.42 | 1.5 | 0.053 | 0.19 | | < 0.001 | < 0.001 | < 0.001 | < 0.001 |
| Firmicutes Clostridiaceae | 0.29 | 0.3 | 0.29 | 0.013 | 0.26 | 0.31 | 0.32 | 0.017 | 0.02 | | 0.10 | 0.33 | < 0.001 | < 0.001 |
| Firmicutes Clostridiales | 1.14 | 1.16 | 1.07 | 0.036 | 1.1 | 1.13 | 1.2 | 0.044 | 0.41 | | 0.09 | < 0.001 | < 0.001 | < 0.001 |
| Firmicutes Clostridiales Family XIII. Incertae Sedis | 0.13 | 0.14 | 0.12 | 0.005 | 0.12 | 0.12 | 0.14 | 0.004 | 0.20 | | < 0.001 | 0.20 | 0.30 | < 0.001 |
| Firmicutes Clostridioides | 0.13 | 0.14 | 0.13 | 0.004 | 0.15 | 0.15 | 0.16 | 0.01 | 0.53 | | 0.19 | 0.32 | 0.68 | 0.004 |
| Firmicutes Clostridium | 1.86 | 1.98 | 1.87 | 0.041 | 1.74 | 2.03 | 1.98 | 0.061 | 0.02 | | < 0.001 | 0.38 | < 0.001 | < 0.001 |
| Firmicutes Desulfitobacterium | 0.11 | 0.1 | 0.1 | 0.002 | 0.1 | 0.1 | 0.1 | 0.001 | 0.33 | | 0.38 | 0.001 | 0.64 | 0.1 |
| Firmicutes Desulfotomaculum | 0.09 | 0.09 | 0.09 | 0.002 | 0.09 | 0.09 | 0.09 | 0.002 | 0.02 | | 0.008 | 0.03 | < 0.001 | < 0.001 |
| Firmicutes Dialister | 0.1 | 0.21 | 0.15 | 0.028 | 0.1 | 0.2 | 0.14 | 0.033 | 0.97 | | < 0.001 | < 0.001 | 0.21 | 0.005 |
| Firmicutes Enterococcus | 0.16 | 0.15 | 0.15 | 0.005 | 0.14 | 0.15 | 0.16 | 0.004 | 0.03 | | < 0.001 | < 0.001 | < 0.001 | < 0.001 |
| Firmicutes Erysipelotrichaceae | 0.1 | 0.1 | 0.11 | 0.003 | 0.1 | 0.11 | 0.1 | 0.003 | 0.25 | | 0.06 | < 0.001 | 0.83 | < 0.001 |
| Firmicutes Ethanoligenens | 0.18 | 0.16 | 0.15 | 0.008 | 0.15 | 0.17 | 0.19 | 0.01 | 0.01 | | < 0.001 | < 0.001 | < 0.001 | < 0.001 |
| Firmicutes Eubacterium | 0.91 | 0.96 | 0.94 | 0.03 | 0.97 | 0.93 | 0.89 | 0.015 | 0.06 | | < 0.001 | < 0.001 | < 0.001 | < 0.001 |
| Firmicutes Faecalibacterium | 0.96 | 0.98 | 0.9 | 0.036 | 0.91 | 0.95 | 1.03 | 0.037 | 0.24 | | 0.67 | < 0.001 | < 0.001 | < 0.001 |
| Firmicutes Faecalibaculum | 0.11 | 0.12 | 0.12 | 0.004 | 0.12 | 0.12 | 0.12 | 0.003 | 0.007 | | 0.09 | 0.01 | 0.31 | 0.003 |
| Firmicutes Firmicutes | 0.25 | 0.26 | 0.26 | 0.005 | 0.23 | 0.27 | 0.27 | 0.008 | 0.12 | | 0.21 | 0.02 | < 0.001 | < 0.001 |
| Firmicutes Flavonifractor | 0.5 | 0.48 | 0.45 | 0.025 | 0.5 | 0.48 | 0.53 | 0.035 | 0.80 | | < 0.001 | < 0.001 | 0.02 | < 0.001 |
| Firmicutes Herbinix | 0.12 | 0.13 | 0.12 | 0.003 | 0.12 | 0.12 | 0.13 | 0.002 | 0.97 | | 0.007 | 0.73 | 0.09 | 0.0 |
| Firmicutes Hungateiclostridiaceae | 0.32 | 0.29 | 0.27 | 0.018 | 0.26 | 0.29 | 0.33 | 0.019 | 0.03 | | < 0.001 | < 0.001 | < 0.001 | < 0.001 |
| Firmicutes Hungateiclostridium | 0.14 | 0.14 | 0.13 | 0.004 | 0.12 | 0.14 | 0.15 | 0.006 | 0.004 | | 0.23 | < 0.001 | < 0.001 | < 0.001 |
| Firmicutes Intestinimonas | 0.31 | 0.3 | 0.28 | 0.016 | 0.31 | 0.3 | 0.33 | 0.021 | 0.85 | | 0.005 | < 0.001 | 0.43 | < 0.001 |
| Firmicutes Lachnoanaerobaculum | 0.18 | 0.19 | 0.18 | 0.006 | 0.17 | 0.18 | 0.18 | 0.003 | 0.10 | | < 0.001 | 0.14 | 0.87 | 0.001 |
| Firmicutes Lachnoclostridium | 1.51 | 1.64 | 1.45 | 0.052 | 1.49 | 1.56 | 1.59 | 0.026 | 0.10 | | < 0.001 | < 0.001 | 0.002 | < 0.001 |
| Firmicutes Lachnospiraceae | 2.06 | 2.13 | 1.94 | 0.068 | 1.97 | 2.02 | 2.07 | 0.03 | 0.007 | | < 0.001 | < 0.001 | 0.42 | < 0.001 |
| Firmicutes Lactobacillaceae | 0.12 | 0.12 | 0.11 | 0.006 | 0.1 | 0.12 | 0.13 | 0.007 | 0.13 | | 0.21 | 0.18 | < 0.001 | < 0.001 |
| Firmicutes Lactobacillus | 0.72 | 0.67 | 0.69 | 0.046 | 0.64 | 0.67 | 0.79 | 0.047 | 0.41 | | < 0.001 | < 0.001 | < 0.001 | < 0.001 |
| Firmicutes Lactococcus | 0.07 | 0.09 | 0.06 | 0.009 | 0.06 | 0.07 | 0.06 | 0.002 | 0.94 | | < 0.001 | 0.26 | < 0.001 | 0.2 |
| Firmicutes Megasphaera | 0.28 | 0.3 | 0.5 | 0.072 | 0.47 | 0.86 | 0.29 | 0.199 | 0.44 | | < 0.001 | < 0.001 | < 0.001 | < 0.001 |
| Firmicutes Mogibacterium | 0.15 | 0.17 | 0.14 | 0.009 | 0.14 | 0.15 | 0.16 | 0.007 | 0.35 | | < 0.001 | < 0.001 | 0.03 | < 0.001 |
| Firmicutes Mordavella | 0.46 | 0.5 | 0.44 | 0.016 | 0.44 | 0.47 | 0.48 | 0.011 | 0.02 | | < 0.001 | < 0.001 | 0.50 | < 0.001 |
| Firmicutes Oscillibacter | 0.87 | 0.84 | 0.78 | 0.044 | 0.84 | 0.84 | 0.92 | 0.054 | 0.60 | | < 0.001 | < 0.001 | < 0.001 | < 0.001 |
| Firmicutes Paenibacillaceae | 0.2 | 0.2 | 0.19 | 0.005 | 0.19 | 0.2 | 0.21 | 0.005 | < 0.001 | | 0.65 | 0.001 | 0.003 | < 0.001 |
| Firmicutes Paenibacillus | 0.95 | 0.92 | 0.88 | 0.025 | 0.89 | 0.91 | 0.95 | 0.013 | < 0.001 | | < 0.001 | < 0.001 | < 0.001 | < 0.001 |
| Firmicutes Pediococcus | 0.06 | 0.07 | 0.05 | 0.005 | 0.04 | 0.08 | 0.05 | 0.009 | 0.007 | | < 0.001 | < 0.001 | < 0.001 | < 0.001 |
| Firmicutes Planococcus | 0.09 | 0.09 | 0.09 | 0.002 | 0.09 | 0.09 | 0.09 | 0.001 | 0.33 | | 0.97 | 0.88 | 0.13 | 0.4 |
| Firmicutes Pseudoclostridium | 0.07 | 0.07 | 0.06 | 0.002 | 0.06 | 0.07 | 0.08 | 0.004 | 0.001 | | 0.72 | 0.01 | 0.001 | < 0.001 |
| Firmicutes Roseburia | 0.52 | 0.55 | 0.5 | 0.015 | 0.51 | 0.52 | 0.52 | 0.009 | 0.32 | | < 0.001 | < 0.001 | 0.05 | < 0.001 |
| Firmicutes Ruminococcaceae | 0.34 | 0.31 | 0.3 | 0.015 | 0.27 | 0.35 | 0.37 | 0.024 | 0.003 | | < 0.001 | < 0.001 | < 0.001 | < 0.001 |
| Firmicutes Ruminococcus | 1.38 | 1.35 | 1.29 | 0.071 | 1.05 | 1.31 | 1.27 | 0.07 | < 0.001 | | < 0.001 | < 0.001 | < 0.001 | < 0.001 |
| Firmicutes Selenomonas | 0.52 | 0.5 | 0.51 | 0.015 | 0.5 | 0.54 | 0.5 | 0.01 | 0.33 | | < 0.001 | 0.002 | < 0.001 | 0.2 |
| Firmicutes Staphylococcus | 0.25 | 0.26 | 0.25 | 0.009 | 0.23 | 0.27 | 0.26 | 0.01 | 0.10 | | 0.04 | 0.23 | < 0.001 | < 0.001 |
| Firmicutes Streptococcaceae | 0.14 | 0.14 | 0.13 | 0.004 | 0.12 | 0.15 | 0.14 | 0.005 | 0.002 | | 0.70 | < 0.001 | < 0.001 | < 0.001 |
| Firmicutes Streptococcus | 0.45 | 0.45 | 0.43 | 0.005 | 0.42 | 0.46 | 0.45 | 0.01 | 0.002 | | 0.21 | < 0.001 | < 0.001 | < 0.001 |
| Firmicutes Weissella | 0.1 | 0.12 | 0.06 | 0.013 | 0.09 | 0.08 | 0.11 | 0.022 | 0.33 | | < 0.001 | < 0.001 | < 0.001 | < 0.001 |
| Fusobacteria Fusobacterium | 0.14 | 0.15 | 0.14 | 0.006 | 0.13 | 0.16 | 0.16 | 0.007 | 0.15 | | 0.03 | 0.93 | < 0.001 | < 0.001 |
| Proteobacteria Achromobacter | 0.1 | 0.1 | 0.1 | 0.003 | 0.1 | 0.1 | 0.1 | 0.003 | 0.33 | | 0.05 | 0.003 | 0.08 | 0.006 |
| Proteobacteria Acinetobacter | 0.14 | 0.14 | 0.14 | 0.003 | 0.14 | 0.15 | 0.17 | 0.006 | 0.29 | | 0.56 | 0.15 | < 0.001 | < 0.001 |
| Proteobacteria Aeromonadaceae | 0.1 | 0.1 | 0.1 | 0.004 | 0.1 | 0.11 | 0.09 | 0.003 | 0.38 | | 0.98 | 0.36 | < 0.001 | 0.4 |
| Proteobacteria Aeromonas | 0.16 | 0.17 | 0.15 | 0.004 | 0.16 | 0.17 | 0.15 | 0.004 | 0.97 | | 0.02 | 0.26 | 0.97 | 0.3 |
| Proteobacteria Alphaproteobacteria | 0.16 | 0.16 | 0.16 | 0.004 | 0.16 | 0.15 | 0.16 | 0.003 | 0.35 | | 0.83 | 0.06 | 0.63 | < 0.001 |
| Proteobacteria Azospirillum | 0.1 | 0.1 | 0.1 | 0.003 | 0.1 | 0.1 | 0.11 | 0.002 | 0.44 | | 0.72 | < 0.001 | 0.41 | < 0.001 |
| Proteobacteria Betaproteobacteria | 0.16 | 0.16 | 0.16 | 0.003 | 0.16 | 0.16 | 0.16 | 0.003 | 0.67 | | 0.85 | 0.47 | 0.62 | 0.2 |
| Proteobacteria Bordetella | 0.15 | 0.15 | 0.14 | 0.005 | 0.15 | 0.15 | 0.16 | 0.003 | 0.40 | | 0.21 | < 0.001 | 0.70 | < 0.001 |
| Proteobacteria Bradyrhizobium | 0.15 | 0.15 | 0.14 | 0.004 | 0.14 | 0.14 | 0.15 | 0.004 | 0.05 | | 0.10 | < 0.001 | 0.60 | < 0.001 |
| Proteobacteria Burkholderia | 0.31 | 0.3 | 0.3 | 0.006 | 0.3 | 0.32 | 0.31 | 0.009 | 0.78 | | 0.26 | 0.005 | < 0.001 | 0.006 |
| Proteobacteria Burkholderiaceae | 0.14 | 0.14 | 0.13 | 0.004 | 0.14 | 0.14 | 0.14 | 0.003 | 0.03 | | 0.006 | < 0.001 | 0.01 | < 0.001 |
| Proteobacteria Burkholderiales | 0.18 | 0.18 | 0.17 | 0.006 | 0.18 | 0.18 | 0.19 | 0.004 | 0.29 | | 0.34 | 0.001 | 0.31 | < 0.001 |
| Proteobacteria Campylobacter | 0.12 | 0.11 | 0.12 | 0.003 | 0.11 | 0.12 | 0.12 | 0.004 | 0.05 | | 0.16 | 0.36 | < 0.001 | 0.002 |
| Proteobacteria Cupriavidus | 0.09 | 0.08 | 0.08 | 0.002 | 0.09 | 0.08 | 0.09 | 0.002 | 0.58 | | 0.04 | 0.001 | 0.49 | < 0.001 |
| Proteobacteria Desulfovibrio | 0.33 | 0.32 | 0.32 | 0.008 | 0.33 | 0.33 | 0.34 | 0.007 | 0.90 | | 0.02 | 0.006 | 0.80 | 0.002 |
| Proteobacteria Enterobacter | 0.12 | 0.12 | 0.12 | 0.002 | 0.12 | 0.13 | 0.12 | 0.002 | 0.33 | | 0.58 | 0.003 | 0.08 | 0.2 |
| Proteobacteria Enterobacterales | 0.16 | 0.16 | 0.15 | 0.004 | 0.16 | 0.17 | 0.16 | 0.003 | 0.26 | | 0.72 | < 0.001 | 0.12 | 0.003 |
| Proteobacteria Enterobacteriaceae | 0.16 | 0.16 | 0.15 | 0.003 | 0.16 | 0.15 | 0.15 | 0.002 | 0.97 | | 0.87 | < 0.001 | 0.54 | 0.0 |
| Proteobacteria Gammaproteobacteria | 0.26 | 0.24 | 0.24 | 0.005 | 0.25 | 0.26 | 0.25 | 0.006 | 0.37 | | 0.002 | 0.001 | < 0.001 | 0.007 |
| Proteobacteria Geobacter | 0.25 | 0.25 | 0.24 | 0.006 | 0.24 | 0.25 | 0.25 | 0.004 | 0.16 | | 0.15 | 0.005 | 0.63 | < 0.001 |
| Proteobacteria Halomonas | 0.13 | 0.13 | 0.12 | 0.003 | 0.13 | 0.13 | 0.12 | 0.003 | 0.96 | | 0.78 | 0.04 | 0.83 | 0.8 |
| Proteobacteria Marinobacter | 0.09 | 0.09 | 0.09 | 0.002 | 0.09 | 0.09 | 0.09 | 0.002 | 0.94 | | 0.21 | 0.67 | 0.99 | 0.4 |
| Proteobacteria Massilia | 0.11 | 0.11 | 0.11 | 0.003 | 0.11 | 0.11 | 0.11 | 0.002 | 0.87 | | 0.18 | 0.13 | 0.70 | 0.6 |
| Proteobacteria Methylobacterium | 0.1 | 0.1 | 0.09 | 0.003 | 0.1 | 0.09 | 0.1 | 0.003 | 0.02 | | 0.21 | < 0.001 | 0.93 | < 0.001 |
| Proteobacteria Pantoea | 0.09 | 0.08 | 0.08 | 0.003 | 0.09 | 0.08 | 0.09 | 0.003 | 0.94 | | < 0.001 | < 0.001 | 0.83 | 0.1 |
| Proteobacteria Paraburkholderia | 0.12 | 0.12 | 0.12 | 0.002 | 0.12 | 0.12 | 0.12 | 0.002 | 0.33 | | 0.67 | 0.20 | 0.96 | 0.2 |
| Proteobacteria Paracoccus | 0.08 | 0.08 | 0.08 | 0.003 | 0.08 | 0.08 | 0.08 | 0.002 | 0.47 | | 0.73 | 0.002 | 0.76 | 0.0 |
| Proteobacteria Pelobacter | 0.1 | 0.09 | 0.09 | 0.003 | 0.1 | 0.1 | 0.1 | 0.002 | 0.97 | | 0.82 | 0.26 | 0.63 | 0.007 |
| Proteobacteria Proteobacteria | 0.95 | 0.95 | 0.93 | 0.021 | 0.92 | 0.98 | 0.95 | 0.018 | 0.30 | | 0.43 | < 0.001 | < 0.001 | < 0.001 |
| Proteobacteria Pseudomonadaceae | 0.29 | 0.29 | 0.27 | 0.007 | 0.29 | 0.28 | 0.29 | 0.005 | 0.89 | | 0.89 | < 0.001 | 0.33 | < 0.001 |
| Proteobacteria Pseudomonas | 0.89 | 0.87 | 0.84 | 0.019 | 0.88 | 0.88 | 0.88 | 0.014 | 0.38 | | 0.001 | < 0.001 | 0.09 | < 0.001 |
| Proteobacteria Rhizobiaceae | 0.14 | 0.14 | 0.13 | 0.004 | 0.13 | 0.13 | 0.14 | 0.003 | 0.007 | | 0.88 | 0.002 | 0.87 | < 0.001 |
| Proteobacteria Rhizobium | 0.23 | 0.23 | 0.22 | 0.005 | 0.22 | 0.22 | 0.23 | 0.004 | 0.009 | | 0.22 | < 0.001 | 0.58 | < 0.001 |
| Proteobacteria Serratia | 0.11 | 0.11 | 0.11 | 0.002 | 0.11 | 0.11 | 0.11 | 0.002 | 0.27 | | 0.97 | 0.001 | 1.00 | 0.001 |
| Proteobacteria Shewanella | 0.13 | 0.13 | 0.12 | 0.002 | 0.13 | 0.14 | 0.13 | 0.002 | 0.58 | | 0.03 | 0.001 | 0.001 | < 0.001 |
| Proteobacteria Sinorhizobium | 0.08 | 0.08 | 0.08 | 0.002 | 0.08 | 0.08 | 0.09 | 0.002 | 0.02 | | 0.68 | 0.03 | 0.79 | < 0.001 |
| Proteobacteria Sphingobium | 0.11 | 0.11 | 0.1 | 0.003 | 0.11 | 0.1 | 0.11 | 0.003 | 0.97 | | 0.07 | 0.009 | 0.92 | 0.0 |
| Proteobacteria Sphingomonas | 0.16 | 0.16 | 0.15 | 0.004 | 0.15 | 0.16 | 0.16 | 0.004 | 0.13 | | 0.98 | 0.18 | 0.02 | 0.003 |
| Proteobacteria Stenotrophomonas | 0.1 | 0.1 | 0.09 | 0.003 | 0.1 | 0.09 | 0.09 | 0.002 | 0.89 | | 0.03 | < 0.001 | 0.85 | 0.7 |
| Proteobacteria Vibrio | 0.23 | 0.22 | 0.22 | 0.004 | 0.22 | 0.23 | 0.22 | 0.003 | 0.12 | | < 0.001 | < 0.001 | < 0.001 | 0.1 |
| Proteobacteria Xanthomonas | 0.1 | 0.1 | 0.09 | 0.002 | 0.1 | 0.1 | 0.1 | 0.002 | 0.60 | | 0.001 | < 0.001 | 0.33 | 0.008 |
| Spirochaetes Sphaerochaeta | 0.09 | 0.09 | 0.08 | 0.002 | 0.09 | 0.09 | 0.09 | 0.001 | 0.006 | | 0.04 | < 0.001 | 0.04 | < 0.001 |
| Spirochaetes Treponema | 0.41 | 0.43 | 0.4 | 0.023 | 0.37 | 0.39 | 0.48 | 0.017 | 0.13 | | 0.28 | < 0.001 | < 0.001 | < 0.001 |
| Synergistetes Cloacibacillus | 0.09 | 0.09 | 0.08 | 0.004 | 0.08 | 0.08 | 0.09 | 0.004 | 0.12 | | 0.72 | < 0.001 | 0.06 | < 0.001 |
| Tenericutes Mycoplasma | 0.15 | 0.16 | 0.16 | 0.007 | 0.13 | 0.16 | 0.17 | 0.01 | 0.001 | | 0.05 | 0.02 | < 0.001 | < 0.001 |
| All others | 22.9 | 22.7 | 22.3 | 0.34 | 22.43 | 22.89 | 23.48 | 0.276 | 0.22 | | < 0.001 | < 0.001 | < 0.001 | < 0.001 |

**Table S12**: Effect of 3-nitrooxypropanol (3-NOP) on bacterial taxonomical composition (relative abundance %) in metatranscriptomics. SEM: Standard error of mean; Trt: treatment group; W: week.

|  | **Control** | | | | **3-nitrooxypropanol (3-NOP)** | | | | **Significance** | | | | |  |
| --- | --- | --- | --- | --- | --- | --- | --- | --- | --- | --- | --- | --- | --- | --- |
|  | **W4** | **W8** | **W12** | **SEM** | **W4** | **W8** | **W12** | **SEM** | **Trt** | **W4  vs  W8** | **W4 vs  W12** | **Trt:  W4  vs W8** | **Trt:  W4  vs W8** |  |
|  |  |  |  |  |  |  |  |  |  |  |  |  |  |  |
| Bacteria | 3.74 | 3.68 | 3.61 | 0.07 | 3.63 | 3.58 | 3.77 | 0.056 | 0.71 | < 0.001 | 0.6 | 0.001 | < 0.001 |  |
| Actinobacteria Actinobacteria | 0.75 | 0.74 | 0.71 | 0.021 | 0.68 | 0.71 | 0.85 | 0.027 | 0.72 | < 0.001 | < 0.001 | 0.14 | < 0.001 |  |
| Actinobacteria Actinomyces | 0.12 | 0.12 | 0.11 | 0.004 | 0.11 | 0.12 | 0.13 | 0.004 | 0.92 | < 0.001 | 0.3 | 0.07 | < 0.001 |  |
| Actinobacteria Adlercreutzia | 0.08 | 0.08 | 0.08 | 0.003 | 0.07 | 0.07 | 0.09 | 0.004 | 0.88 | < 0.001 | 1.0 | 0.02 | < 0.001 |  |
| Actinobacteria Arthrobacter | 0.12 | 0.12 | 0.12 | 0.003 | 0.12 | 0.13 | 0.13 | 0.003 | 0.71 | < 0.001 | 0.7 | < 0.001 | < 0.001 |  |
| Actinobacteria Atopobiaceae | 0.11 | 0.11 | 0.14 | 0.011 | 0.09 | 0.12 | 0.15 | 0.015 | 0.21 | < 0.001 | < 0.001 | < 0.001 | < 0.001 |  |
| Actinobacteria Atopobium | 0.11 | 0.12 | 0.11 | 0.006 | 0.1 | 0.11 | 0.1 | 0.005 | 0.32 | < 0.001 | 0.7 | 0.73 | < 0.001 |  |
| Actinobacteria Bifidobacteriaceae | 0.19 | 0.22 | 0.14 | 0.037 | 0.16 | 0.14 | 0.2 | 0.024 | 0.21 | < 0.001 | < 0.001 | < 0.001 | < 0.001 |  |
| Actinobacteria Bifidobacterium | 1.34 | 1.57 | 0.77 | 0.407 | 1.72 | 0.78 | 1.11 | 0.17 | 0.45 | < 0.001 | < 0.001 | < 0.001 | < 0.001 |  |
| Actinobacteria  Collinsella | 0.21 | 0.21 | 0.21 | 0.007 | 0.18 | 0.2 | 0.22 | 0.011 | 0.16 | < 0.001 | 0.8 | < 0.001 | < 0.001 |  |
| Actinobacteria Corynebacteriaceae | 0.15 | 0.14 | 0.14 | 0.005 | 0.15 | 0.14 | 0.18 | 0.007 | 0.93 | 0.04 | < 0.001 | 0.02 | < 0.001 |  |
| Actinobacteria Corynebacterium | 0.59 | 0.59 | 0.57 | 0.011 | 0.55 | 0.56 | 0.7 | 0.037 | 0.75 | < 0.001 | 0.8 | < 0.001 | < 0.001 |  |
| Actinobacteria Cryptobacterium | 0.02 | 0.02 | 0.04 | 0.006 | 0.01 | 0.02 | 0.02 | 0.002 | 0.21 | < 0.001 | < 0.001 | < 0.001 | 0.39 |  |
| Actinobacteria Denitrobacterium | 0.07 | 0.08 | 0.08 | 0.002 | 0.06 | 0.07 | 0.09 | 0.005 | 0.59 | < 0.001 | < 0.001 | < 0.001 | < 0.001 |  |
| Actinobacteria Eggerthella | 0.11 | 0.11 | 0.11 | 0.004 | 0.09 | 0.11 | 0.13 | 0.007 | 0.65 | 0.003 | 0.8 | 0.68 | < 0.001 |  |
| Actinobacteria Gordonibacter | 0.14 | 0.14 | 0.14 | 0.005 | 0.12 | 0.14 | 0.16 | 0.008 | 0.23 | < 0.001 | 0.9 | < 0.001 | < 0.001 |  |
| Actinobacteria Mycobacterium | 0.09 | 0.09 | 0.09 | 0.002 | 0.09 | 0.09 | 0.13 | 0.013 | 0.14 | < 0.001 | 0.0 | < 0.001 | < 0.001 |  |
| Actinobacteria  Olsenella | 1.12 | 1.15 | 1.49 | 0.116 | 0.94 | 1.27 | 1.53 | 0.129 | 0.34 | < 0.001 | < 0.001 | < 0.001 | < 0.001 |  |
| Actinobacteria Rhodococcus | 0.1 | 0.1 | 0.09 | 0.002 | 0.1 | 0.1 | 0.1 | 0.002 | 0.47 | < 0.001 | 0.5 | < 0.001 | < 0.001 |  |
| Actinobacteria  Slackia | 0.16 | 0.17 | 0.18 | 0.008 | 0.14 | 0.16 | 0.19 | 0.009 | 0.37 | 0.13 | < 0.001 | 0.55 | < 0.001 |  |
| Actinobacteria Streptomyces | 0.44 | 0.44 | 0.43 | 0.012 | 0.42 | 0.43 | 0.45 | 0.009 | 0.98 | < 0.001 | 0.9 | < 0.001 | < 0.001 |  |
| Actinobacteria Streptomycetaceae | 0.18 | 0.18 | 0.17 | 0.006 | 0.17 | 0.17 | 0.19 | 0.004 | 0.92 | < 0.001 | < 0.001 | < 0.001 | < 0.001 |  |
| Bacteroidetes  Alistipes | 0.27 | 0.25 | 0.26 | 0.011 | 0.27 | 0.25 | 0.25 | 0.005 | 0.98 | < 0.001 | < 0.001 | 0.01 | 0.04 |  |
| Bacteroidetes Bacteroidaceae | 0.45 | 0.42 | 0.44 | 0.015 | 0.46 | 0.44 | 0.4 | 0.018 | 0.78 | < 0.001 | < 0.001 | 0.003 | < 0.001 |  |
| Bacteroidetes Bacteroidales | 0.1 | 0.09 | 0.1 | 0.004 | 0.1 | 0.09 | 0.09 | 0.004 | 0.98 | < 0.001 | < 0.001 | 0.30 | 0.51 |  |
| Bacteroidetes Bacteroides | 2.01 | 1.87 | 1.96 | 0.074 | 2.04 | 1.92 | 1.74 | 0.069 | 0.98 | < 0.001 | < 0.001 | < 0.001 | < 0.001 |  |
| Bacteroidetes Bacteroidia | 0.59 | 0.56 | 0.6 | 0.03 | 0.6 | 0.57 | 0.53 | 0.02 | 0.82 | < 0.001 | < 0.001 | 0.79 | < 0.001 |  |
| Bacteroidetes Barnesiella | 0.32 | 0.29 | 0.31 | 0.017 | 0.34 | 0.32 | 0.29 | 0.014 | 0.59 | < 0.001 | < 0.001 | < 0.001 | < 0.001 |  |
| Bacteroidetes Capnocytophaga | 0.13 | 0.12 | 0.12 | 0.005 | 0.15 | 0.13 | 0.12 | 0.008 | 0.71 | < 0.001 | < 0.001 | 0.88 | < 0.001 |  |
| Bacteroidetes Chryseobacterium | 0.11 | 0.11 | 0.11 | 0.003 | 0.14 | 0.12 | 0.1 | 0.008 | 0.65 | < 0.001 | < 0.001 | 0.92 | < 0.001 |  |
| Bacteroidetes Flavobacteriaceae | 0.13 | 0.13 | 0.13 | 0.003 | 0.14 | 0.13 | 0.12 | 0.006 | 0.88 | 0.80 | 0.1 | 0.41 | < 0.001 |  |
| Bacteroidetes Flavobacterium | 0.17 | 0.17 | 0.17 | 0.003 | 0.18 | 0.17 | 0.15 | 0.006 | 0.43 | 0.05 | < 0.001 | < 0.001 | 0.005 |  |
| Bacteroidetes Hymenobacter | 0.31 | 0.28 | 0.29 | 0.011 | 0.3 | 0.29 | 0.27 | 0.009 | 0.92 | < 0.001 | < 0.001 | 0.94 | < 0.001 |  |
| Bacteroidetes Hymenobacteraceae | 0.09 | 0.08 | 0.08 | 0.003 | 0.09 | 0.09 | 0.08 | 0.003 | 0.54 | < 0.001 | < 0.001 | < 0.001 | 0.94 |  |
| Bacteroidetes Mucinivorans | 0.1 | 0.1 | 0.11 | 0.007 | 0.09 | 0.1 | 0.09 | 0.003 | 0.29 | < 0.001 | 0.2 | < 0.001 | 0.001 |  |
| Bacteroidetes Muribaculum | 0.48 | 0.46 | 0.52 | 0.029 | 0.5 | 0.46 | 0.45 | 0.018 | 0.92 | < 0.001 | 0.4 | < 0.001 | < 0.001 |  |
| Bacteroidetes Petrimonas | 0.11 | 0.1 | 0.1 | 0.005 | 0.11 | 0.11 | 0.1 | 0.005 | 0.92 | < 0.001 | < 0.001 | 0.17 | 0.003 |  |
| Bacteroidetes Pontibacter | 0.1 | 0.09 | 0.09 | 0.002 | 0.1 | 0.1 | 0.09 | 0.003 | 0.87 | 0.001 | < 0.001 | 0.005 | 0.79 |  |
| Bacteroidetes Porphyromonas | 0.36 | 0.33 | 0.34 | 0.016 | 0.36 | 0.35 | 0.32 | 0.012 | 0.92 | < 0.001 | < 0.001 | 0.02 | < 0.001 |  |
| Bacteroidetes  Prevotella | 17.65 | 16.96 | 18.74 | 0.749 | 16.1 | 16.42 | 14.9 | 0.559 | 0.26 | < 0.001 | < 0.001 | < 0.001 | < 0.001 |  |
| Bacteroidetes Prevotellaceae | 1.12 | 1.24 | 1.4 | 0.11 | 1.12 | 1.17 | 1.04 | 0.06 | 0.65 | < 0.001 | < 0.001 | < 0.001 | < 0.001 |  |
| Bacteroidetes Proteiniphilum | 0.08 | 0.07 | 0.08 | 0.004 | 0.08 | 0.08 | 0.07 | 0.003 | 0.98 | < 0.001 | < 0.001 | 0.89 | < 0.001 |  |
| Bacteroidetes  Spirosoma | 0.12 | 0.12 | 0.12 | 0.003 | 0.12 | 0.12 | 0.11 | 0.003 | 0.39 | < 0.001 | < 0.001 | 0.008 | 0.76 |  |
| Bacteroidetes  Tannerella | 0.39 | 0.36 | 0.41 | 0.022 | 0.41 | 0.37 | 0.34 | 0.009 | 0.84 | < 0.001 | < 0.001 | < 0.001 | < 0.001 |  |
| Bacteroidetes Tannerellaceae | 0.15 | 0.14 | 0.14 | 0.005 | 0.16 | 0.15 | 0.14 | 0.005 | 0.43 | 0.002 | < 0.001 | < 0.001 | < 0.001 |  |
| Balneolaeota Candidatus Cyclonatronum | 0.02 | 0.02 | 0.02 | 0.001 | 0.02 | 0.02 | 0.06 | 0.014 | 0.59 | 0.02 | 0.002 | < 0.001 | < 0.001 |  |
| Chlorobi Prosthecochloris | 0.1 | 0.09 | 0.09 | 0.004 | 0.09 | 0.08 | 0.14 | 0.011 | 0.29 | 0.10 | 0.0 | < 0.001 | < 0.001 |  |
| Cyanobacteria Synechococcus | 0.1 | 0.1 | 0.1 | 0.002 | 0.11 | 0.1 | 0.1 | 0.004 | 0.78 | 0.02 | 0.001 | 0.35 | < 0.001 |  |
| Deinococcus-Thermus Deinococcus | 0.15 | 0.14 | 0.14 | 0.006 | 0.13 | 0.14 | 0.15 | 0.004 | 0.90 | < 0.001 | 0.4 | 0.17 | < 0.001 |  |
| Fibrobacteres Fibrobacter | 3.77 | 3.65 | 3.92 | 0.425 | 3.15 | 2.8 | 3.22 | 0.269 | 0.21 | < 0.001 | < 0.001 | < 0.001 | < 0.001 |  |
| Firmicutes Acidaminococcus | 0.23 | 0.27 | 0.27 | 0.016 | 0.23 | 0.27 | 0.26 | 0.018 | 0.98 | 0.01 | < 0.001 | 0.005 | 0.11 |  |
| Firmicutes  Anaerostipes | 0.27 | 0.31 | 0.3 | 0.019 | 0.43 | 0.31 | 0.36 | 0.04 | 0.01 | < 0.001 | < 0.001 | < 0.001 | < 0.001 |  |
| Firmicutes  Anaerotignum | 0.1 | 0.08 | 0.08 | 0.005 | 0.11 | 0.08 | 0.1 | 0.006 | 0.59 | < 0.001 | < 0.001 | < 0.001 | < 0.001 |  |
| Firmicutes  Bacillaceae | 0.18 | 0.17 | 0.17 | 0.009 | 0.19 | 0.17 | 0.19 | 0.008 | 0.34 | 0.006 | < 0.001 | < 0.001 | 0.03 |  |
| Firmicutes Bacilli | 0.19 | 0.21 | 0.21 | 0.01 | 0.2 | 0.2 | 0.2 | 0.008 | 0.37 | < 0.001 | < 0.001 | < 0.001 | < 0.001 |  |
| Firmicutes Bacillus | 0.46 | 0.47 | 0.47 | 0.024 | 0.55 | 0.46 | 0.49 | 0.025 | 0.26 | < 0.001 | < 0.001 | < 0.001 | < 0.001 |  |
| Firmicutes Blautia | 0.85 | 0.94 | 0.89 | 0.036 | 1.22 | 0.95 | 1.05 | 0.061 | 0.003 | < 0.001 | < 0.001 | < 0.001 | < 0.001 |  |
| Firmicutes  Butyrivibrio | 3.17 | 2.98 | 2.77 | 0.218 | 3 | 2.95 | 2.9 | 0.195 | 0.52 | 0.002 | < 0.001 | < 0.001 | < 0.001 |  |
| Firmicutes Carnobacterium | 0.04 | 0.04 | 0.04 | 0.007 | 0.07 | 0.04 | 0.04 | 0.008 | 0.03 | < 0.001 | < 0.001 | < 0.001 | < 0.001 |  |
| Firmicutes Cellulosilyticum | 0.11 | 0.11 | 0.12 | 0.009 | 0.16 | 0.11 | 0.11 | 0.012 | 0.26 | 0.31 | < 0.001 | < 0.001 | < 0.001 |  |
| Firmicutes Christensenella | 0.14 | 0.14 | 0.13 | 0.006 | 0.12 | 0.13 | 0.16 | 0.007 | 0.92 | < 0.001 | 0.4 | < 0.001 | < 0.001 |  |
| Firmicutes Clostridia | 1.54 | 1.54 | 1.41 | 0.065 | 1.61 | 1.49 | 1.79 | 0.075 | 0.51 | < 0.001 | 0.6 | < 0.001 | < 0.001 |  |
| Firmicutes Clostridiaceae | 0.24 | 0.29 | 0.35 | 0.048 | 0.54 | 0.32 | 0.37 | 0.059 | < 0.001 | < 0.001 | < 0.001 | < 0.001 | < 0.001 |  |
| Firmicutes Clostridiales | 1.34 | 1.36 | 1.28 | 0.05 | 1.4 | 1.36 | 1.6 | 0.06 | 0.42 | < 0.001 | < 0.001 | < 0.001 | < 0.001 |  |
| Firmicutes Clostridiales Family XIII. Incertae Sedis | 0.12 | 0.13 | 0.12 | 0.006 | 0.12 | 0.12 | 0.14 | 0.005 | 0.92 | < 0.001 | < 0.001 | 0.79 | < 0.001 |  |
| Firmicutes Clostridium | 1.24 | 1.47 | 1.71 | 0.199 | 2.5 | 1.56 | 1.96 | 0.259 | < 0.001 | < 0.001 | < 0.001 | < 0.001 | < 0.001 |  |
| Firmicutes Desulfitobacterium | 0.07 | 0.08 | 0.07 | 0.002 | 0.07 | 0.08 | 0.09 | 0.005 | 0.56 | < 0.001 | < 0.001 | < 0.001 | 0.005 |  |
| Firmicutes Desulfosporosinus | 0.06 | 0.07 | 0.06 | 0.002 | 0.06 | 0.07 | 0.06 | 0.007 | 0.91 | < 0.001 | < 0.001 | 0.89 | 0.06 |  |
| Firmicutes Desulfotomaculum | 0.06 | 0.07 | 0.06 | 0.003 | 0.07 | 0.06 | 0.08 | 0.006 | 0.21 | < 0.001 | 0.3 | < 0.001 | < 0.001 |  |
| Firmicutes Dialister | 0.16 | 0.34 | 0.21 | 0.055 | 0.16 | 0.31 | 0.22 | 0.06 | 0.92 | < 0.001 | < 0.001 | < 0.001 | 0.002 |  |
| Firmicutes  Enterococcus | 0.1 | 0.09 | 0.1 | 0.008 | 0.12 | 0.09 | 0.09 | 0.007 | 0.21 | 0.15 | < 0.001 | < 0.001 | < 0.001 |  |
| Firmicutes Erysipelotrichaceae | 0.09 | 0.09 | 0.1 | 0.006 | 0.11 | 0.09 | 0.1 | 0.006 | 0.08 | 0.02 | < 0.001 | < 0.001 | < 0.001 |  |
| Firmicutes Ethanoligenens | 0.14 | 0.13 | 0.12 | 0.008 | 0.12 | 0.16 | 0.16 | 0.018 | 0.93 | < 0.001 | 0.003 | 0.008 | < 0.001 |  |
| Firmicutes  Eubacterium | 1.51 | 1.72 | 1.6 | 0.073 | 2.06 | 1.68 | 1.71 | 0.111 | 0.10 | < 0.001 | < 0.001 | < 0.001 | < 0.001 |  |
| Firmicutes Faecalibacterium | 1.24 | 1.24 | 1.16 | 0.036 | 1.16 | 1.21 | 1.38 | 0.04 | 0.92 | < 0.001 | < 0.001 | < 0.001 | < 0.001 |  |
| Firmicutes Faecalibaculum | 0.17 | 0.17 | 0.17 | 0.006 | 0.19 | 0.19 | 0.2 | 0.007 | 0.07 | < 0.001 | 0.1 | 0.001 | 0.008 |  |
| Firmicutes Firmicutes | 0.3 | 0.29 | 0.3 | 0.017 | 0.3 | 0.28 | 0.29 | 0.013 | 0.78 | 0.61 | < 0.001 | < 0.001 | 0.005 |  |
| Firmicutes  Flavonifractor | 0.41 | 0.4 | 0.37 | 0.023 | 0.39 | 0.39 | 0.46 | 0.019 | 0.72 | < 0.001 | 0.5 | < 0.001 | < 0.001 |  |
| Firmicutes Herbinix | 0.18 | 0.2 | 0.22 | 0.01 | 0.31 | 0.24 | 0.23 | 0.028 | 0.003 | < 0.001 | < 0.001 | < 0.001 | < 0.001 |  |
| Firmicutes Hungateiclostridiaceae | 0.42 | 0.35 | 0.33 | 0.03 | 0.34 | 0.35 | 0.52 | 0.05 | 0.78 | 0.26 | < 0.001 | < 0.001 | < 0.001 |  |
| Firmicutes Hungateiclostridium | 0.17 | 0.15 | 0.13 | 0.009 | 0.19 | 0.14 | 0.15 | 0.013 | 0.78 | < 0.001 | < 0.001 | < 0.001 | 0.03 |  |
| Firmicutes Intestinimonas | 0.45 | 0.44 | 0.42 | 0.024 | 0.45 | 0.45 | 0.55 | 0.028 | 0.65 | < 0.001 | < 0.001 | < 0.001 | < 0.001 |  |
| Firmicutes Lachnoanaerobaculum | 0.36 | 0.38 | 0.34 | 0.018 | 0.45 | 0.35 | 0.39 | 0.027 | 0.21 | < 0.001 | 0.0 | < 0.001 | < 0.001 |  |
| Firmicutes Lachnoclostridium | 1.97 | 2.13 | 1.99 | 0.068 | 2.28 | 2.05 | 2.53 | 0.1 | 0.005 | < 0.001 | < 0.001 | < 0.001 | < 0.001 |  |
| Firmicutes Lachnospiraceae | 3.81 | 4.16 | 3.9 | 0.144 | 4.39 | 4.05 | 4.62 | 0.15 | 0.20 | < 0.001 | < 0.001 | < 0.001 | < 0.001 |  |
| Firmicutes Lactobacillaceae | 0.1 | 0.11 | 0.12 | 0.011 | 0.12 | 0.1 | 0.15 | 0.01 | 0.02 | < 0.001 | < 0.001 | < 0.001 | < 0.001 |  |
| Firmicutes  Lactobacillus | 0.46 | 0.44 | 0.47 | 0.029 | 0.52 | 0.43 | 0.48 | 0.02 | 0.10 | 0.01 | < 0.001 | < 0.001 | < 0.001 |  |
| Firmicutes  Megamonas | 0.13 | 0.14 | 0.11 | 0.009 | 0.12 | 0.11 | 0.1 | 0.009 | 0.78 | < 0.001 | 0.0 | < 0.001 | < 0.001 |  |
| Firmicutes  Megasphaera | 0.27 | 0.3 | 1.08 | 0.29 | 0.23 | 3.83 | 0.3 | 1.302 | 0.92 | < 0.001 | < 0.001 | < 0.001 | < 0.001 |  |
| Firmicutes Mogibacterium | 0.15 | 0.18 | 0.15 | 0.009 | 0.15 | 0.17 | 0.19 | 0.01 | 0.51 | < 0.001 | < 0.001 | < 0.001 | < 0.001 |  |
| Firmicutes  Mordavella | 0.77 | 0.82 | 0.73 | 0.029 | 0.75 | 0.73 | 0.97 | 0.042 | 0.34 | < 0.001 | 0.6 | < 0.001 | < 0.001 |  |
| Firmicutes Negativicoccus | 0.03 | 0.03 | 0.03 | 0.001 | 0.03 | 0.03 | 0.04 | 0.002 | 0.98 | 0.002 | 0.0 | < 0.001 | < 0.001 |  |
| Firmicutes  Oscillibacter | 0.76 | 0.72 | 0.66 | 0.041 | 0.75 | 0.71 | 0.84 | 0.036 | 0.53 | < 0.001 | < 0.001 | < 0.001 | < 0.001 |  |
| Firmicutes Paenibacillaceae | 0.18 | 0.19 | 0.18 | 0.004 | 0.19 | 0.18 | 0.2 | 0.006 | 0.90 | < 0.001 | 0.1 | 0.001 | < 0.001 |  |
| Firmicutes  Paenibacillus | 0.73 | 0.73 | 0.69 | 0.018 | 0.72 | 0.7 | 0.8 | 0.021 | 0.75 | < 0.001 | < 0.001 | < 0.001 | < 0.001 |  |
| Firmicutes  Pediococcus | 0.19 | 0.25 | 0.19 | 0.015 | 0.17 | 0.2 | 0.17 | 0.014 | 0.60 | < 0.001 | < 0.001 | < 0.001 | 0.003 |  |
| Firmicutes Pseudoclostridium | 0.1 | 0.11 | 0.1 | 0.005 | 0.11 | 0.11 | 0.11 | 0.004 | 0.21 | 0.21 | < 0.001 | 0.001 | < 0.001 |  |
| Firmicutes Roseburia | 0.8 | 0.92 | 0.83 | 0.031 | 0.91 | 0.83 | 0.94 | 0.025 | 0.21 | < 0.001 | < 0.001 | < 0.001 | 0.02 |  |
| Firmicutes Ruminococcaceae | 0.57 | 0.54 | 0.49 | 0.031 | 0.5 | 0.47 | 0.65 | 0.039 | 0.93 | < 0.001 | < 0.001 | < 0.001 | < 0.001 |  |
| Firmicutes Ruminococcus | 3.47 | 3.71 | 3.05 | 0.218 | 2.59 | 2.83 | 2.86 | 0.145 | 0.16 | < 0.001 | < 0.001 | < 0.001 | < 0.001 |  |
| Firmicutes Selenomonadaceae | 0.09 | 0.1 | 0.1 | 0.004 | 0.08 | 0.09 | 0.11 | 0.008 | 0.92 | 0.001 | < 0.001 | 0.84 | < 0.001 |  |
| Firmicutes Selenomonas | 0.87 | 0.78 | 0.91 | 0.038 | 0.91 | 1.01 | 0.92 | 0.058 | 0.59 | < 0.001 | < 0.001 | < 0.001 | < 0.001 |  |
| Firmicutes Staphylococcus | 0.12 | 0.13 | 0.15 | 0.011 | 0.18 | 0.13 | 0.12 | 0.013 | 0.12 | < 0.001 | < 0.001 | < 0.001 | < 0.001 |  |
| Firmicutes Streptococcaceae | 0.14 | 0.15 | 0.14 | 0.01 | 0.16 | 0.15 | 0.14 | 0.009 | 0.26 | < 0.001 | < 0.001 | < 0.001 | < 0.001 |  |
| Firmicutes Streptococcus | 0.33 | 0.34 | 0.34 | 0.016 | 0.41 | 0.34 | 0.36 | 0.02 | 0.01 | < 0.001 | < 0.001 | < 0.001 | < 0.001 |  |
| Firmicutes Thermoanaerobacterales Family III. Incertae Sedis | 0.06 | 0.05 | 0.05 | 0.006 | 0.07 | 0.05 | 0.05 | 0.009 | 0.48 | < 0.001 | < 0.001 | < 0.001 | 0.005 |  |
| Firmicutes Veillonella | 0.06 | 0.06 | 0.05 | 0.002 | 0.06 | 0.05 | 0.08 | 0.011 | 0.18 | 0.31 | < 0.001 | < 0.001 | 0.81 |  |
| Firmicutes Weissella | 0.03 | 0.06 | 0.04 | 0.008 | 0.04 | 0.04 | 0.03 | 0.002 | 0.56 | < 0.001 | < 0.001 | < 0.001 | < 0.001 |  |
| Fusobacteria Fusobacterium | 0.08 | 0.09 | 0.1 | 0.007 | 0.14 | 0.1 | 0.09 | 0.014 | 0.20 | < 0.001 | < 0.001 | < 0.001 | < 0.001 |  |
| Proteobacteria Acidithiobacillus | 0.08 | 0.08 | 0.07 | 0.01 | 0.05 | 0.05 | 0.04 | 0.007 | 0.42 | < 0.001 | 0.002 | < 0.001 | < 0.001 |  |
| Proteobacteria Acinetobacter | 0.1 | 0.1 | 0.1 | 0.004 | 0.13 | 0.09 | 0.11 | 0.007 | 0.16 | 0.007 | 0.003 | < 0.001 | < 0.001 |  |
| Proteobacteria Aeromonadaceae | 0.18 | 0.18 | 0.17 | 0.015 | 0.15 | 0.18 | 0.14 | 0.02 | 0.15 | 0.05 | < 0.001 | < 0.001 | < 0.001 |  |
| Proteobacteria Aeromonas | 0.27 | 0.27 | 0.25 | 0.019 | 0.22 | 0.26 | 0.22 | 0.023 | 0.21 | < 0.001 | < 0.001 | < 0.001 | < 0.001 |  |
| Proteobacteria Alphaproteobacteria | 0.16 | 0.16 | 0.15 | 0.004 | 0.14 | 0.15 | 0.16 | 0.004 | 0.21 | < 0.001 | 0.4 | 0.31 | < 0.001 |  |
| Proteobacteria Altererythrobacter | 0.07 | 0.07 | 0.06 | 0.005 | 0.08 | 0.08 | 0.07 | 0.009 | 0.08 | < 0.001 | 0.0 | < 0.001 | < 0.001 |  |
| Proteobacteria Betaproteobacteria | 0.15 | 0.15 | 0.14 | 0.005 | 0.13 | 0.15 | 0.14 | 0.005 | 0.10 | 0.001 | < 0.001 | < 0.001 | < 0.001 |  |
| Proteobacteria Bordetella | 0.11 | 0.1 | 0.1 | 0.003 | 0.1 | 0.1 | 0.11 | 0.002 | 0.75 | 0.12 | < 0.001 | 0.11 | < 0.001 |  |
| Proteobacteria Bradyrhizobium | 0.1 | 0.1 | 0.1 | 0.002 | 0.09 | 0.09 | 0.1 | 0.003 | 0.08 | 0.007 | 0.001 | 0.02 | < 0.001 |  |
| Proteobacteria  Buchnera | 0.12 | 0.08 | 0.11 | 0.016 | 0.06 | 0.09 | 0.04 | 0.017 | 0.21 | < 0.001 | < 0.001 | < 0.001 | 0.76 |  |
| Proteobacteria Burkholderia | 0.19 | 0.19 | 0.18 | 0.003 | 0.17 | 0.19 | 0.2 | 0.005 | 0.37 | < 0.001 | 0.2 | < 0.001 | < 0.001 |  |
| Proteobacteria Burkholderiaceae | 0.1 | 0.1 | 0.09 | 0.002 | 0.09 | 0.1 | 0.1 | 0.002 | 0.21 | 0.97 | 0.001 | < 0.001 | < 0.001 |  |
| Proteobacteria Burkholderiales | 0.16 | 0.16 | 0.16 | 0.004 | 0.15 | 0.16 | 0.16 | 0.004 | 0.10 | 0.11 | < 0.001 | < 0.001 | < 0.001 |  |
| Proteobacteria Campylobacter | 0.1 | 0.11 | 0.1 | 0.004 | 0.13 | 0.1 | 0.1 | 0.007 | 0.02 | < 0.001 | < 0.001 | < 0.001 | < 0.001 |  |
| Proteobacteria Candidatus Riesia | 0.11 | 0.1 | 0.12 | 0.015 | 0.06 | 0.11 | 0.04 | 0.017 | 0.01 | < 0.001 | 0.7 | < 0.001 | < 0.001 |  |
| Proteobacteria Desulfovibrio | 0.4 | 0.38 | 0.38 | 0.015 | 0.35 | 0.4 | 0.42 | 0.012 | 0.34 | 0.34 | < 0.001 | < 0.001 | < 0.001 |  |
| Proteobacteria Enterobacter | 0.1 | 0.09 | 0.09 | 0.003 | 0.1 | 0.09 | 0.09 | 0.003 | 0.53 | 0.001 | < 0.001 | 0.97 | < 0.001 |  |
| Proteobacteria Enterobacterales | 0.16 | 0.15 | 0.14 | 0.005 | 0.15 | 0.16 | 0.15 | 0.006 | 0.41 | < 0.001 | < 0.001 | < 0.001 | < 0.001 |  |
| Proteobacteria Enterobacteriaceae | 0.13 | 0.13 | 0.12 | 0.003 | 0.14 | 0.13 | 0.13 | 0.004 | 0.67 | 0.46 | < 0.001 | 0.39 | < 0.001 |  |
| Proteobacteria Erwinia | 0.03 | 0.03 | 0.03 | 0.001 | 0.06 | 0.03 | 0.03 | 0.007 | 0.01 | < 0.001 | < 0.001 | < 0.001 | < 0.001 |  |
| Proteobacteria Filomicrobium | 0.04 | 0.04 | 0.04 | 0.005 | 0.06 | 0.07 | 0.04 | 0.017 | 0.37 | < 0.001 | < 0.001 | < 0.001 | < 0.001 |  |
| Proteobacteria Francisella | 0.05 | 0.05 | 0.05 | 0.004 | 0.08 | 0.05 | 0.06 | 0.007 | 0.01 | < 0.001 | 0.0 | < 0.001 | < 0.001 |  |
| Proteobacteria Gammaproteobacteria | 0.3 | 0.26 | 0.25 | 0.01 | 0.29 | 0.3 | 0.29 | 0.011 | 0.37 | < 0.001 | < 0.001 | < 0.001 | < 0.001 |  |
| Proteobacteria Geobacter | 0.23 | 0.23 | 0.21 | 0.006 | 0.21 | 0.22 | 0.24 | 0.005 | 0.32 | < 0.001 | < 0.001 | 0.005 | < 0.001 |  |
| Proteobacteria Haematospirillum | 0.11 | 0.18 | 0.13 | 0.016 | 0.08 | 0.1 | 0.11 | 0.015 | 0.37 | < 0.001 | < 0.001 | < 0.001 | < 0.001 |  |
| Proteobacteria Halomonas | 0.12 | 0.12 | 0.11 | 0.005 | 0.11 | 0.12 | 0.11 | 0.005 | 0.21 | 0.20 | < 0.001 | < 0.001 | < 0.001 |  |
| Proteobacteria Histophilus | 0.1 | 0.11 | 0.09 | 0.017 | 0.07 | 0.1 | 0.04 | 0.018 | 0.41 | < 0.001 | < 0.001 | < 0.001 | 0.16 |  |
| Proteobacteria Marinobacter | 0.09 | 0.09 | 0.08 | 0.003 | 0.08 | 0.09 | 0.09 | 0.003 | 0.001 | 0.005 | < 0.001 | < 0.001 | < 0.001 |  |
| Proteobacteria Moraxella | 0.22 | 0.16 | 0.2 | 0.023 | 0.1 | 0.17 | 0.08 | 0.025 | 0.001 | < 0.001 | < 0.001 | < 0.001 | < 0.001 |  |
| Proteobacteria Neisseriales | 0.34 | 0.29 | 0.34 | 0.05 | 0.16 | 0.27 | 0.11 | 0.046 | 0.10 | < 0.001 | < 0.001 | < 0.001 | < 0.001 |  |
| Proteobacteria Pandoraea | 0.11 | 0.11 | 0.12 | 0.009 | 0.08 | 0.1 | 0.09 | 0.008 | 0.02 | 0.06 | 0.0 | < 0.001 | < 0.001 |  |
| Proteobacteria Pantoea | 0.08 | 0.07 | 0.07 | 0.003 | 0.09 | 0.08 | 0.1 | 0.007 | 0.02 | < 0.001 | < 0.001 | 0.008 | < 0.001 |  |
| Proteobacteria Pelobacter | 0.1 | 0.1 | 0.09 | 0.003 | 0.1 | 0.1 | 0.12 | 0.005 | 0.37 | < 0.001 | < 0.001 | < 0.001 | < 0.001 |  |
| Proteobacteria Proteobacteria | 1.3 | 1.2 | 1.19 | 0.045 | 1.1 | 1.21 | 1.1 | 0.056 | 0.003 | < 0.001 | < 0.001 | < 0.001 | < 0.001 |  |
| Proteobacteria Pseudodesulfovibrio | 0.09 | 0.09 | 0.08 | 0.003 | 0.08 | 0.09 | 0.09 | 0.002 | 0.08 | 0.002 | < 0.001 | < 0.001 | < 0.001 |  |
| Proteobacteria Pseudomonadaceae | 0.29 | 0.29 | 0.27 | 0.01 | 0.25 | 0.28 | 0.28 | 0.01 | 0.08 | 0.81 | < 0.001 | < 0.001 | < 0.001 |  |
| Proteobacteria Pseudomonas | 0.75 | 0.73 | 0.7 | 0.015 | 0.72 | 0.72 | 0.73 | 0.02 | 0.18 | 0.54 | < 0.001 | < 0.001 | < 0.001 |  |
| Proteobacteria Rhizobiaceae | 0.14 | 0.13 | 0.12 | 0.004 | 0.12 | 0.12 | 0.13 | 0.004 | 0.09 | 0.008 | < 0.001 | 0.17 | < 0.001 |  |
| Proteobacteria Rhizobium | 0.18 | 0.18 | 0.17 | 0.003 | 0.16 | 0.17 | 0.18 | 0.004 | 0.002 | 0.002 | < 0.001 | < 0.001 | < 0.001 |  |
| Proteobacteria Rhodobacteraceae | 0.07 | 0.08 | 0.07 | 0.003 | 0.07 | 0.07 | 0.08 | 0.004 | 0.98 | < 0.001 | < 0.001 | 0.05 | < 0.001 |  |
| Proteobacteria Shewanella | 0.12 | 0.1 | 0.1 | 0.004 | 0.12 | 0.12 | 0.11 | 0.006 | 0.21 | < 0.001 | < 0.001 | < 0.001 | < 0.001 |  |
| Proteobacteria Sphingobium | 0.1 | 0.1 | 0.09 | 0.003 | 0.09 | 0.09 | 0.1 | 0.002 | 0.52 | < 0.001 | 0.9 | 0.008 | < 0.001 |  |
| Proteobacteria Sphingomonas | 0.11 | 0.11 | 0.1 | 0.002 | 0.1 | 0.11 | 0.11 | 0.002 | 0.03 | < 0.001 | 0.003 | 0.008 | < 0.001 |  |
| Proteobacteria Vibrio | 0.21 | 0.2 | 0.19 | 0.006 | 0.22 | 0.21 | 0.2 | 0.008 | 0.65 | < 0.001 | < 0.001 | < 0.001 | < 0.001 |  |
| Proteobacteria Yersiniaceae | 0.1 | 0.11 | 0.1 | 0.006 | 0.1 | 0.11 | 0.09 | 0.008 | 0.20 | 0.001 | < 0.001 | < 0.001 | < 0.001 |  |
| Spirochaetes Sphaerochaeta | 0.1 | 0.1 | 0.1 | 0.003 | 0.11 | 0.1 | 0.11 | 0.004 | 0.009 | 0.007 | < 0.001 | < 0.001 | 0.24 |  |
| Spirochaetes Treponema | 0.65 | 0.7 | 0.59 | 0.045 | 0.55 | 0.65 | 0.7 | 0.038 | 0.39 | < 0.001 | 0.003 | < 0.001 | < 0.001 |  |
| Tenericutes Mycoplasma | 0.08 | 0.08 | 0.09 | 0.008 | 0.1 | 0.08 | 0.08 | 0.006 | 0.26 | < 0.001 | < 0.001 | < 0.001 | < 0.001 |  |
| All others | 19.28 | 18.81 | 18.39 | 0.358 | 19.28 | 18.92 | 19.54 | 0.371 | 0.98 | < 0.001 | < 0.001 | < 0.001 | < 0.001 |  |

**Table S13:** Average methane (CH_4_) and hydrogen (H_2_) gas data in control and 3-nitrooxypropanol (3-NOP) treated cows at weeks 2, 6, 9, and 15.

| **Week** | **Treatment** | **CH_4_ (gram/day)** | **CH_4_ yield (gram/kg DMI)** | **H_2_ (gram/day)** |  |
| --- | --- | --- | --- | --- | --- |
| W2 | Control | 331.50 | 15.45 | 0.004 |  |
| W6 | Control | 364.26 | 14.91 | 0.001 |  |
| W9 | Control | 372.00 | 15.03 | 0.002 |  |
| W15 | Control | 385.29 | 18.15 | 0.001 |  |
| W2 | 3-NOP | 239.00 | 11.79 | 0.499 |  |
| W6 | 3-NOP | 257.58 | 10.86 | 0.516 |  |
| W9 | 3-NOP | 271.09 | 10.75 | 0.578 |  |
| W15 | 3-NOP | 306.04 | 12.34 | 0.320 |  |

Across all weeks, the three measures described above were significantly (*P* < 0.001) different between the two groups Melgar et al., [1].

**Table S14**: Effect of 3-nitrooxpropanol (3-NOP) on hydrogenase subgroups (cpm; copies per million) including [FeFe], [Fe], and [NiFe] hydrogenases in metagenomics.

|  | **Control** | | | **3-nitrooxypropanol (3-NOP)** | | |  |
| --- | --- | --- | --- | --- | --- | --- | --- |
|  | **W4** | **W8** | **W12** | **W4** | **W8** | **W12** | **Significance** |
| [Fe] | 5.31 | 3.13 | 2.29 | 2.70 | 1.26 | 1.65 | 0.065 |
| [FeFe] Group A1 | 534.76 | 530.54 | 528.54 | 514.10 | 518.74 | 528.94 | 0.258 |
| [FeFe] Group A2 | 42.62 | 44.02 | 48.49 | 46.31 | 45.93 | 45.35 | 0.716 |
| [FeFe] Group A3 | 403.13 | 415.73 | 375.74 | 358.55 | 380.80 | 430.21 | 0.654 |
| [FeFe] Group A4 | 5.33 | 6.37 | 5.54 | 4.82 | 5.61 | 6.12 | 0.864 |
| [FeFe] Group B | 232.79 | 243.37 | 230.44 | 239.46 | 263.24 | 258.33 | 0.007 |
| [FeFe] Group C1 | 43.96 | 37.45 | 33.80 | 31.51 | 32.35 | 45.21 | 0.633 |
| [FeFe] Group C2 | 303.95 | 287.86 | 278.62 | 272.39 | 287.88 | 296.48 | 0.590 |
| [FeFe] Group C3 | 240.00 | 235.81 | 233.18 | 251.97 | 261.92 | 263.09 | 0.000 |
| [NiFe] Group 1a | 0.01 | 0.01 | 0.02 | 0.00 | 0.00 | 0.01 | NA |
| [NiFe] Group 1b | 0.33 | 0.25 | 0.44 | 0.38 | 0.40 | 0.35 | 0.341 |
| [NiFe] Group 1c | 0.02 | 0.04 | 0.02 | 0.01 | 0.04 | 0.06 | NA |
| [NiFe] Group 1d | 1.09 | 0.92 | 1.36 | 1.35 | 1.33 | 1.00 | 0.853 |
| [NiFe] Group 1e | 0.02 | 0.00 | 0.02 | 0.02 | 0.02 | 0.01 | NA |
| [NiFe] Group 1f | 0.03 | 0.11 | 0.14 | 0.13 | 0.03 | 0.08 | NA |
| [NiFe] Group 1g | 0.00 | 0.00 | 0.01 | 0.00 | 0.00 | 0.00 | NA |
| [NiFe] Group 1h | 0.00 | 0.01 | 0.02 | 0.01 | 0.02 | 0.00 | NA |
| [NiFe] Group 1i | 3.02 | 3.16 | 2.49 | 2.93 | 2.97 | 2.99 | 0.808 |
| [NiFe] Group 1j | 0.03 | 0.00 | 0.03 | 0.00 | 0.00 | 0.00 | NA |
| [NiFe] Group 2a | 0.02 | 0.01 | 0.01 | 0.01 | 0.02 | 0.00 | NA |
| [NiFe] Group 2b | 0.02 | 0.00 | 0.01 | 0.01 | 0.00 | 0.02 | NA |
| [NiFe] Group 3a | 25.50 | 30.16 | 20.38 | 19.82 | 20.96 | 23.60 | 0.113 |
| [NiFe] Group 3b | 1.07 | 1.13 | 1.16 | 0.89 | 1.20 | 1.00 | 0.847 |
| [NiFe] Group 3c | 30.04 | 37.32 | 25.53 | 24.37 | 26.67 | 28.38 | 0.139 |
| [NiFe] Group 3d | 0.01 | 0.04 | 0.02 | 0.01 | 0.00 | 0.01 | NA |
| [NiFe] Group 4a | 3.99 | 4.00 | 4.46 | 4.48 | 4.08 | 3.62 | 1.000 |
| [NiFe] Group 4b | 1.58 | 1.79 | 2.21 | 2.17 | 1.89 | 1.61 | 0.768 |
| [NiFe] Group 4c | 2.87 | 3.10 | 3.86 | 3.75 | 3.42 | 3.17 | 0.823 |
| [NiFe] Group 4d | 4.82 | 5.07 | 6.10 | 5.46 | 5.68 | 4.68 | 0.859 |
| [NiFe] Group 4e | 39.97 | 40.02 | 46.60 | 41.20 | 40.72 | 37.06 | 0.323 |
| [NiFe] Group 4f | 0.92 | 0.59 | 0.58 | 0.84 | 0.71 | 0.61 | 0.514 |
| [NiFe] Group 4g | 23.51 | 22.46 | 21.55 | 20.85 | 21.35 | 23.41 | 0.697 |
| [NiFe] Group 4h | 18.75 | 19.57 | 13.48 | 13.45 | 12.92 | 15.00 | 0.032 |
| [NiFe] Group 4i | 28.38 | 29.40 | 20.52 | 22.85 | 19.63 | 22.44 | 0.055 |

**Table S15**: Effect of 3-nitrooxpropanol (3-NOP) on hydrogenase subgroups (cpm; copies per million) including [FeFe], [Fe], and [NiFe] hydrogenases in metatranscriptomics.

|  | **Control** | | | **(3-NOP)** | | |  |
| --- | --- | --- | --- | --- | --- | --- | --- |
|  | **W4** | **W8** | **W12** | **W4** | **W8** | **W12** | **Significance** |
| [Fe] | 0.99 | 0.97 | 1.6 | 0.69 | 1.74 | 1.8 | 0.49 |
| [FeFe] Group A1 | 1693.66 | 1732.16 | 1927.43 | 1875.54 | 1700.15 | 1653.77 | 0.61 |
| [FeFe] Group A2 | 46.4 | 51.01 | 55.35 | 45.16 | 49.88 | 53.32 | 0.67 |
| [FeFe] Group A3 | 1416.19 | 1475.28 | 1236.03 | 1057.11 | 1155.55 | 1418.94 | 0.11 |
| [FeFe] Group A4 | 9.88 | 11.95 | 12.27 | 10.18 | 11.3 | 13.79 | 0.77 |
| [FeFe] Group B | 329.97 | 337.64 | 329.65 | 357.43 | 372.43 | 416.09 | < 0.001 |
| [FeFe] Group C1 | 48.84 | 44.56 | 36.68 | 28.62 | 35.82 | 40.93 | 0.04 |
| [FeFe] Group C2 | 316.32 | 297.5 | 266.69 | 256.22 | 298.38 | 254.06 | 0.15 |
| [FeFe] Group C3 | 277.01 | 240.53 | 230.71 | 291.54 | 310.48 | 328.38 | < 0.001 |
| [NiFe] Group 1a | 0.01 | 0.03 | 0.03 | 0.02 | 0.01 | 0.05 | NA |
| [NiFe] Group 1b | 3.27 | 4.21 | 5.57 | 4.19 | 5.88 | 4.93 | 0.39 |
| [NiFe] Group 1c | 0.1 | 0.05 | 0.04 | 0.06 | 0.06 | 0.1 | NA |
| [NiFe] Group 1d | 10.47 | 10.93 | 17.72 | 18.82 | 20.72 | 17.76 | 0.006 |
| [NiFe] Group 1e | 0.2 | 0.27 | 0.48 | 0.57 | 0.61 | 0.5 | 0.12 |
| [NiFe] Group 1f | 0.11 | 0.15 | 0.16 | 0.16 | 0.11 | 0.18 | NA |
| [NiFe] Group 1g | 0.02 | 0.04 | 0.03 | 0 | 0 | 0.04 | NA |
| [NiFe] Group 1h | 0.06 | 0.06 | 0.06 | 0 | 0 | 0.11 | NA |
| [NiFe] Group 1i | 7.09 | 8.94 | 7.51 | 5.14 | 8.15 | 8.05 | 0.55 |
| [NiFe] Group 1j | 0.03 | 0.02 | 0.05 | 0.1 | 0.05 | 0.17 | NA |
| [NiFe] Group 1k | 0.02 | 0.01 | 0.02 | 0.03 | 0 | 0.01 | NA |
| [NiFe] Group 2a | 0 | 0.01 | 0.01 | 0 | 0.01 | 0 | NA |
| [NiFe] Group 2b | 0.03 | 0.02 | 0.02 | 0 | 0 | 0.02 | NA |
| [NiFe] Group 3a | 252.69 | 346.34 | 273.86 | 241.98 | 232.82 | 295.92 | 0.22 |
| [NiFe] Group 3b | 0.5 | 0.42 | 0.45 | 0.36 | 0.35 | 0.2 | 0.42 |
| [NiFe] Group 3c | 351.15 | 457.93 | 347.51 | 345.09 | 310.81 | 340.83 | 0.07 |
| [NiFe] Group 3d | 0.02 | 0.02 | 0.02 | 0 | 0 | 0.01 | NA |
| [NiFe] Group 4a | 5.33 | 5.23 | 5.64 | 5.16 | 5.42 | 4.8 | 0.93 |
| [NiFe] Group 4b | 2.21 | 2.72 | 2.95 | 2.16 | 2.3 | 2.21 | 0.61 |
| [NiFe] Group 4c | 4.67 | 4.68 | 5.33 | 5.02 | 4.31 | 4.24 | 0.78 |
| [NiFe] Group 4d | 4.94 | 5.46 | 5.18 | 5.07 | 5.05 | 4.56 | 0.72 |
| [NiFe] Group 4e | 47.43 | 50.14 | 50.7 | 41.31 | 45.24 | 52.76 | 0.29 |
| [NiFe] Group 4f | 0.71 | 0.87 | 0.89 | 0.84 | 0.73 | 0.75 | 0.66 |
| [NiFe] Group 4g | 45.74 | 42.86 | 38.11 | 37.79 | 34.23 | 42.3 | 0.14 |
| [NiFe] Group 4h | 8.33 | 9.44 | 9.05 | 8.78 | 8.44 | 9.13 | 1.00 |
| [NiFe] Group 4i | 29.81 | 42.74 | 38.4 | 33.38 | 32.36 | 31.26 | 0.12 |

**Table S16**: Effect of 3-NOP on hydrogenases (cpm; copies per million) classified into H_2_ production and H_2_ consumption in metagenomics.

|  | **Control** | | | **3-nitrooxypropanol** | | |  |
| --- | --- | --- | --- | --- | --- | --- | --- |
|  |  |  |  | **(3-NOP)** | | |  |
|  | **W4** | **W8** | **W12** | **W4** | **W8** | **W12** | **Significance** |
| Bifurcating hydrogenases | 408.46 | 422.1 | 381.28 | 363.36 | 386.41 | 436.33 | 0.65 |
| Energy-converting hydrogenases | 71.25 | 70.17 | 77.06 | 71.13 | 70.28 | 67.86 | 0.04 |
| Fermentative hydrogenases | 810.17 | 817.94 | 807.47 | 799.87 | 827.92 | 832.62 | 0.93 |
| Methanogenic hydrogenases | 107.98 | 119.59 | 82.2 | 83.19 | 81.44 | 91.08 | 0.77 |
| Respiratory hydrogenases | 4.49 | 4.49 | 4.44 | 4.78 | 4.77 | 4.47 | 0.89 |
| Sensory hydrogenases | 587.91 | 561.11 | 545.61 | 555.87 | 582.14 | 604.77 | 0.77 |
| Formate tetrahydrofolate ligase | 875 | 903.75 | 843.5 | 815 | 941.33 | 947.75 | 0.51 |
| Acetyl-CoA synthase | 22.4 | 19.93 | 25.74 | 19.68 | 23.23 | 20.72 | 0.68 |
| Adenylylsulfate reductase | 94.85 | 49.28 | 71.35 | 54.49 | 71.34 | 60.21 | 0.46 |
| Alternative sulfite reductase | 95.33 | 108.62 | 103.58 | 88.95 | 98.75 | 110.51 | 0.48 |
| Ammonia-forming nitrite reductase | 78.11 | 47.38 | 80.84 | 52.89 | 86.36 | 69.08 | 0.66 |
| Cytochrome bd oxidase | 267.97 | 242.09 | 279.28 | 247.74 | 286.6 | 256.11 | 0.41 |
| Dissimilatory nitrate reductase | 3.83 | 1.5 | 5.63 | 1.63 | 4.13 | 3.16 | 0.36 |
| Dissimilatory sulfite reductase | 2.26 | 1.76 | 2.25 | 1.44 | 3.21 | 2.34 | 0.91 |
| DMSO/TMAO reductase | 7.94 | 10.1 | 7.42 | 7.64 | 7.38 | 9.53 | 0.99 |
| Fumarate reductase | 45.2 | 46.62 | 46 | 52.22 | 48.16 | 55.51 | 0.29 |
| Hydrogenase-associated diaphorases | 547.27 | 675.05 | 556.75 | 578.84 | 550.32 | 633.03 | 0.06 |
| Methyl-CoM reductase | 236.62 | 298.86 | 234.95 | 260.16 | 196.05 | 267.25 | 0.67 |
| Nitrogenases | 688.55 | 715.86 | 721.29 | 713.4 | 685.43 | 716.03 | 0.53 |
| Periplasmic nitrate reductase | 4.84 | 7.01 | 4.85 | 6.41 | 5.2 | 6.28 | 0.57 |

**Table S17**: Effect of 3-NOP on hydrogenases (cpm; copies per million) classified into H_2_ production and H_2_ consumption in metatranscriptomics.

|  | **Control** | | | **3-nitrooxypropanol** | | |  |
| --- | --- | --- | --- | --- | --- | --- | --- |
|  |  |  |  | **(3-NOP)** | | |  |
|  | **W4** | **W8** | **W12** | **W4** | **W8** | **W12** | **Significance** |
| Bifurcating hydrogenases | 1426.07 | 1487.23 | 1248.3 | 1067.29 | 1166.85 | 1432.74 | 0.12 |
| Energy-converting hydrogenases | 103.89 | 103.79 | 100.67 | 90.12 | 89.93 | 104.85 | 0.13 |
| Fermentative hydrogenases | 2070.03 | 2120.8 | 2312.43 | 2278.13 | 2122.46 | 2123.18 | 0.25 |
| Methanogenic hydrogenases | 642.97 | 857.43 | 670.42 | 629.93 | 586.16 | 678.94 | 0.06 |
| Respiratory hydrogenases | 21.04 | 24.28 | 31 | 28.37 | 34.92 | 31.02 | 0.68 |
| Sensory hydrogenases | 642.18 | 582.59 | 534.08 | 576.38 | 644.68 | 623.37 | < 0.001 |
| Formate tetrahydrofolate ligase | 357.25 | 389.5 | 393.75 | 397.75 | 441 | 414.33 | 0.008 |
| Acetyl-CoA synthase | 27.42 | 36.39 | 17.8 | 24.41 | 33.02 | 18.69 | 0.58 |
| Adenylylsulfate reductase | 54.31 | 64.75 | 51.9 | 46.93 | 52.23 | 53 | 0.08 |
| Alternative sulfite reductase | 30.26 | 32.5 | 21.63 | 19.85 | 26.67 | 22.36 | 0.91 |
| Ammonia-forming nitrite reductase | 16.74 | 80.71 | 18.02 | 24.76 | 16.51 | 13.09 | 0.18 |
| Cytochrome bd oxidase | 51.23 | 87.68 | 53.64 | 54.63 | 44.8 | 58.11 | 0.79 |
| Dissimilatory nitrate reductase | 0.56 | 0.93 | 0.61 | 0.59 | 0.52 | 0.3 | 0.06 |
| Dissimilatory sulfite reductase | 20.3 | 25.58 | 13.56 | 14.91 | 23.34 | 15.07 | 0.59 |
| DMSO/TMAO reductase | 6.16 | 5.64 | 7.26 | 6.89 | 5.28 | 5.41 | 0.25 |
| Fumarate reductase | 43.69 | 54.5 | 49.45 | 51.25 | 58.03 | 52.16 | 0.34 |
| Hydrogenase-associated diaphorases | 816.54 | 603.97 | 738.89 | 639.93 | 603.96 | 799.65 | 0.11 |
| Methyl-CoM reductase | 2950.52 | 2161.84 | 2930.19 | 2817.51 | 2441.68 | 3236.49 | 0.72 |
| Nitrogenases | 108.05 | 109.24 | 128.2 | 137.6 | 133.87 | 132.54 | 0.59 |
| Periplasmic nitrate reductase | 1.23 | 1.6 | 1.35 | 1.07 | 1.35 | 1.43 | 0.12 |

Table S18: Effect of 3-nitrooxypropanol (3-NOP) on rumen fermentation variables in early-lactation dairy cows at weeks 4, 8, and 12.

|  | **Week 4** | | **Week 8** | | **Week 12** | |  |
| --- | --- | --- | --- | --- | --- | --- | --- |
|  | **Control** | **3-NOP** | **Control** | **3-NOP** | **Control** | **3-NOP** | **Treatment**  **p value** |
| pH | 5.9 | 6 | 5.74 | 5.96 | 5.86 | 5.9 | 0.04 |
| Ammonia, mM | 2.68 | 1.91 | 2.41 | 1.64 | 2.24 | 2.2 | 0.18 |
| Total VFA, mM | 145.44 | 136.5 | 147.28 | 125.21 | 139.25 | 130.26 | 0.05 |
| VFA, mol% |  |  |  |  |  |  |  |
| Acetate | 60.34 | 54.66 | 58.3 | 53.76 | 58.45 | 54.16 | 0.04 |
| Propionate | 25.16 | 26.72 | 26.92 | 27.88 | 25.41 | 26.34 | 0.51 |
| Butyrate | 11.55 | 14.55 | 11.71 | 13.85 | 13.03 | 15.53 | 0.008 |
| Isobutyrate | 0.52 | 0.59 | 0.55 | 0.73 | 0.54 | 0.58 | 0.42 |
| Valerate | 1.48 | 2.06 | 1.67 | 2.62 | 1.74 | 2.17 | 0.03 |
| Isovalerate | 0.95 | 1.43 | 0.86 | 1.16 | 0.84 | 1.22 | 0.12 |
| Acetate:Propionate | 2.40 | 2.05 | 2.17 | 1.93 | 2.30 | 2.06 | 0.22 |
| Dissolved hydrogen, µg/L | 14.06 | 48.58 | 21.13 | 192.2 | 8.7 | 12.87 | 0.14 |
| Ethanol, mg/kg | 16.51 | 26.68 | 18.58 | 57.25 | 18.77 | 65.85 | 0.01 |
| Formic acid, µM | 41.85 | 64.71 | 40.58 | 94.98 | 28.67 | 96.58 | < 0.001 |
| Total protozoa, × 10^4^ /mL | 307292 | 377214 | 256771 | 306901 | 283594 | 290755.21 | 0.64 |

**Table S19:** Taxonomy (cpm; copies per million) associated with the butyrate pathway in metagenomics in cows supplemented with 3-nitrooxypropanol (3-NOP) compared to control cows at weeks 4, 8, and 12.

|  | **Week 4** | | **Week 8** | | **Week 12** | | |  |
| --- | --- | --- | --- | --- | --- | --- | --- | --- |
|  | Control | 3-NOP | Control | 3-NOP | | Control | 3-NOP | |
| **EC:1.3.8.1** |  |  |  |  | |  |  | |
| K00248\|Butyrivibrio proteoclasticus | 76.72 | 64.00 | 78.53 | 74.35 | | 73.36 | 79.15 | |
| K00248\|Butyrate-producing bacterium SM4/1 | 65.64 | 70.37 | 66.64 | 73.89 | | 63.17 | 74.18 | |
| K00248\|Roseburia hominis | 33.10 | 42.43 | 34.86 | 42.82 | | 36.41 | 40.34 | |
| K00248\|Porphyromonas gingivalis W83 | 27.42 | 42.67 | 33.65 | 39.61 | | 34.50 | 43.98 | |
| K00248\|Eubacterium rectale ATCC 33656 | 30.40 | 38.09 | 33.47 | 33.27 | | 35.96 | 36.89 | |
| K00248\|Odoribacter splanchnicus | 11.41 | 18.09 | 12.96 | 14.10 | | 15.40 | 17.20 | |
| K00248\|Clostridium beijerinckii NCIMB 8052 | 8.93 | 10.62 | 9.83 | 13.59 | | 7.97 | 10.90 | |
| K00248\|Eubacterium sulci | 10.33 | 5.72 | 12.03 | 10.04 | | 8.82 | 11.10 | |
| K00248\|Oscillibacter valericigenes | 8.40 | 8.51 | 10.30 | 10.07 | | 9.45 | 10.45 | |
| K00248\|Brachyspira pilosicoli 95/1000 | 7.38 | 9.46 | 8.22 | 9.28 | | 9.92 | 9.71 | |
| K00248\|Porphyromonas asaccharolytica | 7.12 | 10.64 | 6.43 | 8.45 | | 8.19 | 9.80 | |
| K00248\|Alkaliphilus oremlandii | 7.14 | 9.37 | 8.58 | 8.83 | | 8.16 | 8.27 | |
| K00248\|Brachyspira murdochii | 7.15 | 8.09 | 8.07 | 8.57 | | 8.68 | 9.52 | |
| K00248\|Brachyspira intermedia | 6.94 | 8.52 | 7.29 | 8.30 | | 8.84 | 8.88 | |
| K00248\|Clostridioides difficile 630 | 6.47 | 7.24 | 7.38 | 6.48 | | 7.16 | 7.21 | |
| K00248\|Ilyobacter polytropus | 6.05 | 7.79 | 6.07 | 6.84 | | 6.67 | 6.79 | |
| K00248\|Fusobacterium nucleatum subsp. nucleatum ATCC 25586 | 4.81 | 5.61 | 6.83 | 8.22 | | 7.28 | 7.12 | |
| K00248\|Brachyspira hyodysenteriae WA1 | 5.59 | 7.05 | 6.17 | 6.76 | | 6.85 | 7.15 | |
| K00248\|Clostridium tetani E88 | 5.14 | 6.26 | 5.99 | 8.13 | | 6.73 | 7.16 | |
| K00248\|Acetoanaerobium sticklandii | 6.02 | 6.99 | 6.17 | 6.57 | | 7.15 | 5.48 | |
| K00248\|Bacteroidales bacterium CF | 4.44 | 5.36 | 3.73 | 5.10 | | 4.74 | 4.66 | |
| K00248\|Eubacterium limosum | 3.88 | 4.50 | 5.11 | 4.46 | | 4.64 | 4.70 | |
| K00248\|Clostridium saccharolyticum WM1 | 4.71 | 4.10 | 5.02 | 4.29 | | 3.64 | 4.74 | |
| K00248\|Clostridium sp. SY8519 | 3.87 | 2.80 | 5.70 | 3.96 | | 4.37 | 4.39 | |
| K00248\|Clostridium novyi | 2.97 | 4.50 | 3.76 | 2.02 | | 3.26 | 2.66 | |
| K00248\|Erysipelotrichaceae bacterium I46 | 2.12 | 3.11 | 2.34 | 2.92 | | 2.85 | 2.95 | |
| K00248\|Faecalibaculum rodentium | 2.73 | 1.98 | 1.73 | 3.15 | | 2.91 | 2.12 | |
| K00248\|Clostridium saccharobutylicum | 2.56 | 2.11 | 3.02 | 2.81 | | 1.71 | 1.94 | |
| K00248\|Geosporobacter ferrireducens | 2.22 | 1.83 | 2.42 | 2.44 | | 2.39 | 2.37 | |
| K00248\|Clostridium argentinense | 2.04 | 2.44 | 2.03 | 2.19 | | 2.12 | 2.62 | |
| K00248\|Clostridium tyrobutyricum | 2.22 | 2.09 | 2.48 | 1.92 | | 1.46 | 2.66 | |
| K00248\|Alkaliphilus metalliredigens | 1.80 | 2.02 | 2.05 | 2.63 | | 1.88 | 2.10 | |
| K00248\|Clostridium perfringens 13 | 1.44 | 2.49 | 2.28 | 1.90 | | 1.96 | 2.33 | |
| K00248\|Fusobacterium periodonticum | 1.65 | 2.20 | 1.86 | 1.67 | | 1.96 | 2.27 | |
| K00248\|Clostridium estertheticum | 1.57 | 1.93 | 1.51 | 1.71 | | 1.94 | 2.07 | |
| K00248\|Fusobacterium hwasookii | 1.26 | 2.07 | 1.76 | 1.61 | | 1.83 | 2.14 | |
| K00248\|Clostridium kluyveri DSM 555 | 1.32 | 2.16 | 1.06 | 1.14 | | 1.42 | 2.44 | |
| K00248\|Filifactor alocis | 1.61 | 1.79 | 1.21 | 1.30 | | 1.57 | 1.02 | |
| K00248\|Anaerostipes hadrus | 1.53 | 0.87 | 1.63 | 1.53 | | 1.23 | 0.87 | |
| K00248\|Clostridium carboxidivorans | 1.17 | 1.24 | 1.49 | 1.30 | | 0.93 | 0.93 | |
| K00248\|All other taxa | 8.06 | 10.02 | 9.21 | 11.55 | | 11.31 | 9.28 | |
| **EC:2.3.1.19** |  |  |  |  | |  |  | |
| K00634\|Prevotella ruminicola | 306.31 | 322.73 | 295.73 | 322.99 | | 325.39 | 293.20 | |
| K00634\|Butyrivibrio proteoclasticus | 64.84 | 36.20 | 52.83 | 45.36 | | 44.00 | 53.90 | |
| K00634\|Flavonifractor plautii | 2.45 | 1.53 | 1.56 | 1.72 | | 1.86 | 1.99 | |
| K00634\|All other taxa | 0.74 | 0.43 | 0.38 | 0.95 | | 0.41 | 0.42 | |
| **EC:2.8.3.8** |  |  |  |  | |  |  | |
| K01035\|Odoribacter splanchnicus | 14.87 | 14.74 | 14.98 | 20.34 | | 12.76 | 22.81 | |
| K01034\|Eubacterium sulci | 10.28 | 9.22 | 11.07 | 11.63 | | 8.93 | 12.98 | |
| K01035\|Eubacterium sulci | 9.44 | 8.68 | 8.89 | 10.37 | | 8.81 | 13.29 | |
| K01035\|Porphyromonas crevioricanis | 6.60 | 5.43 | 5.15 | 7.23 | | 6.97 | 7.23 | |
| K01035\|Vibrio mediterranei | 2.97 | 4.05 | 1.75 | 4.13 | | 5.92 | 4.63 | |
| K01035\|Clostridium sp. SY8519 | 3.30 | 2.72 | 5.75 | 3.13 | | 3.62 | 3.04 | |
| K01035\|Marinifilaceae bacterium SPP2 | 2.96 | 3.69 | 1.48 | 3.81 | | 5.10 | 4.32 | |
| K01035\|Intestinimonas butyriciproducens | 2.65 | 1.88 | 1.97 | 2.90 | | 1.43 | 3.55 | |
| K01035\|Flavonifractor plautii | 2.90 | 1.16 | 2.12 | 2.01 | | 1.40 | 1.89 | |
| K01035\|Bacteroidales bacterium CF | 1.43 | 0.43 | 1.21 | 2.62 | | 1.75 | 2.34 | |
| K01035\|Alkaliphilus metalliredigens | 1.60 | 1.47 | 1.14 | 1.47 | | 2.09 | 1.58 | |
| K01035\|Clostridium argentinense | 1.76 | 0.88 | 1.34 | 1.60 | | 1.44 | 1.95 | |
| K01035\|All other taxa | 9.42 | 16.18 | 10.46 | 17.03 | | 14.70 | 13.05 | |
| **EC:2.7.2.7** |  |  |  |  | |  |  | |
| K00929\|Prevotella ruminicola | 131.22 | 141.28 | 138.48 | 146.63 | | 156.36 | 138.64 | |
| K00929\|Butyrivibrio hungatei | 30.80 | 16.39 | 24.81 | 17.34 | | 18.99 | 22.56 | |
| K00929\|Butyrivibrio proteoclasticus | 29.06 | 16.63 | 23.30 | 17.56 | | 18.55 | 21.60 | |
| K00929\|Coprococcus sp. ART55/1 | 9.05 | 10.63 | 9.47 | 10.36 | | 9.77 | 9.80 | |
| K00929\|Mogibacterium diversum | 11.08 | 8.30 | 13.07 | 7.52 | | 8.28 | 10.26 | |
| K00929\|Prevotella denticola | 4.80 | 5.76 | 5.45 | 4.86 | | 6.16 | 4.26 | |
| K00929\|Clostridium sp. SY8519 | 5.02 | 3.77 | 5.43 | 4.99 | | 4.36 | 4.22 | |
| K00929\|Eggerthella sp. YY7918 | 4.19 | 4.84 | 4.06 | 6.86 | | 3.57 | 4.04 | |
| K00929\|Collinsella aerofaciens | 5.08 | 4.16 | 5.63 | 2.44 | | 4.55 | 3.67 | |
| K00929\|Prevotella jejuni | 3.51 | 4.25 | 3.39 | 3.50 | | 4.38 | 2.92 | |
| K00929\|Prevotella fusca | 4.26 | 4.41 | 3.31 | 3.30 | | 3.62 | 2.96 | |
| K00929\|Clostridium cellulovorans | 3.33 | 3.64 | 2.44 | 2.68 | | 2.22 | 2.45 | |
| K00929\|Olsenella uli | 1.94 | 2.35 | 1.33 | 2.65 | | 2.81 | 2.79 | |
| K00929\|Clostridium bornimense | 2.58 | 2.62 | 1.18 | 1.14 | | 2.11 | 2.16 | |
| K00929\|Prevotella melaninogenica | 1.91 | 1.84 | 1.80 | 1.82 | | 1.84 | 1.12 | |
| K00929\|All other taxa | 10.25 | 12.93 | 12.16 | 10.68 | | 14.29 | 8.54 | |

**Table S20:** Taxonomy (cpm; copies per million) associated with the butyrate pathway in metatranscriptomics in cows supplemented with 3-nitrooxypropanol (3-NOP) compared to control cows at weeks 4, 8, and 12.

|  | **W4** | | | **W8** | | | **W12** | | |
| --- | --- | --- | --- | --- | --- | --- | --- | --- | --- |
|  | Control | 3-NOP | 3-NOP | | Control | Control | | 3-NOP |  |
| **EC:1.3.8.1** |  |  |  | |  |  | |  |  |
| K00248\|Roseburia hominis | 133.15 | 172.69 | 144.56 | | 170.06 | 134.78 | | 142.49 |  |
| K00248\|Butyrivibrio proteoclasticus | 98.00 | 122.06 | 114.59 | | 131.70 | 104.56 | | 117.92 |  |
| K00248\|Butyrate-producing bacterium SM4/1 | 82.86 | 128.96 | 95.67 | | 120.95 | 87.58 | | 103.84 |  |
| K00248\|Clostridium beijerinckii NCIMB 8052 | 90.70 | 93.49 | 90.02 | | 103.61 | 92.76 | | 87.05 |  |
| K00248\|Clostridium saccharolyticum WM1 | 64.54 | 60.95 | 55.96 | | 60.89 | 56.12 | | 50.05 |  |
| K00248\|Eubacterium rectale ATCC 33656 | 37.07 | 53.89 | 48.04 | | 54.96 | 45.62 | | 50.59 |  |
| K00248\|Clostridium novyi | 43.19 | 46.69 | 44.70 | | 50.35 | 40.64 | | 45.02 |  |
| K00248\|Clostridium sp. SY8519 | 25.84 | 19.27 | 24.75 | | 26.85 | 22.59 | | 21.82 |  |
| K00248\|Eubacterium sulci | 17.86 | 15.81 | 21.31 | | 21.26 | 17.50 | | 25.36 |  |
| K00248\|Oscillibacter valericigenes | 17.58 | 17.95 | 17.85 | | 23.64 | 19.14 | | 21.58 |  |
| K00248\|Porphyromonas gingivalis W83 | 14.32 | 17.23 | 16.26 | | 16.21 | 18.22 | | 13.91 |  |
| K00248\|Erysipelotrichaceae bacterium I46 | 10.91 | 9.63 | 12.43 | | 11.56 | 11.11 | | 11.76 |  |
| K00248\|Brachyspira pilosicoli 95/1000 | 9.67 | 10.88 | 11.43 | | 12.39 | 10.11 | | 10.05 |  |
| K00248\|Alkaliphilus oremlandii | 8.91 | 11.96 | 10.52 | | 12.04 | 9.69 | | 10.77 |  |
| K00248\|Clostridioides difficile 630 | 8.53 | 10.61 | 9.52 | | 11.51 | 9.36 | | 9.32 |  |
| K00248\|Brachyspira intermedia | 8.03 | 9.72 | 9.67 | | 11.05 | 8.61 | | 9.71 |  |
| K00248\|Fusobacterium nucleatum subsp. nucleatum ATCC 25586 | 5.57 | 8.22 | 7.97 | | 11.70 | 10.28 | | 11.24 |  |
| K00248\|Acetoanaerobium sticklandii | 6.67 | 8.70 | 8.22 | | 10.72 | 7.66 | | 8.61 |  |
| K00248\|Eubacterium limosum | 7.52 | 6.88 | 8.79 | | 9.33 | 8.31 | | 9.09 |  |
| K00248\|Brachyspira murdochii | 7.49 | 7.83 | 8.87 | | 9.09 | 8.05 | | 7.82 |  |
| K00248\|Odoribacter splanchnicus | 7.80 | 7.42 | 8.34 | | 8.89 | 8.08 | | 7.55 |  |
| K00248\|Filifactor alocis | 6.84 | 8.09 | 7.71 | | 9.43 | 6.39 | | 7.91 |  |
| K00248\|Brachyspira hyodysenteriae WA1 | 6.80 | 7.18 | 7.97 | | 8.58 | 7.34 | | 7.33 |  |
| K00248\|Anaerococcus prevotii | 6.16 | 7.23 | 7.30 | | 8.20 | 5.88 | | 7.10 |  |
| K00248\|Clostridium tetani E88 | 4.42 | 7.40 | 6.54 | | 7.35 | 8.58 | | 6.36 |  |
| K00248\|Ilyobacter polytropus | 5.51 | 7.10 | 6.78 | | 7.17 | 5.98 | | 5.98 |  |
| K00248\|Clostridium butyricum | 2.70 | 8.48 | 4.59 | | 6.29 | 6.36 | | 8.82 |  |
| K00248\|Clostridium saccharoperbutylacetonicum | 2.61 | 7.76 | 5.04 | | 5.76 | 6.26 | | 8.81 |  |
| K00248\|Clostridium saccharobutylicum | 2.30 | 7.00 | 3.57 | | 4.89 | 5.17 | | 7.51 |  |
| K00248\|Clostridium tyrobutyricum | 3.95 | 4.41 | 4.50 | | 5.94 | 5.03 | | 6.11 |  |
| K00248\|Alkaliphilus metalliredigens | 2.96 | 4.72 | 4.23 | | 5.55 | 3.40 | | 4.27 |  |
| K00248\|Porphyromonas asaccharolytica | 3.13 | 4.38 | 4.53 | | 3.85 | 4.10 | | 3.72 |  |
| K00248\|Faecalibaculum rodentium | 2.42 | 4.83 | 2.86 | | 3.51 | 3.07 | | 3.91 |  |
| K00248\|Clostridium perfringens 13 | 2.11 | 4.08 | 2.83 | | 3.15 | 3.20 | | 4.76 |  |
| K00248\|Clostridium carboxidivorans | 2.63 | 2.86 | 3.29 | | 3.87 | 2.82 | | 3.98 |  |
| K00248\|Clostridium estertheticum | 2.38 | 3.22 | 2.75 | | 3.73 | 2.96 | | 3.66 |  |
| K00248\|Clostridium argentinense | 1.80 | 3.88 | 2.54 | | 2.72 | 2.90 | | 4.78 |  |
| K00248\|Fusobacterium periodonticum | 1.93 | 3.15 | 2.64 | | 3.09 | 2.61 | | 3.49 |  |
| K00248\|Bacteroidales bacterium CF | 2.39 | 2.67 | 3.20 | | 2.81 | 2.78 | | 2.75 |  |
| K00248\|Clostridium scatologenes | 2.24 | 2.38 | 2.93 | | 3.03 | 2.53 | | 3.40 |  |
| K00248\|Fusobacterium hwasookii | 1.81 | 2.84 | 2.48 | | 2.85 | 2.50 | | 3.32 |  |
| K00248\|Clostridium kluyveri DSM 555 | 1.97 | 2.96 | 2.46 | | 3.88 | 1.98 | | 2.26 |  |
| K00248\|Geosporobacter ferrireducens | 2.37 | 2.05 | 2.23 | | 3.12 | 2.01 | | 1.89 |  |
| K00248\|Fervidobacterium pennivorans | 1.07 | 2.80 | 1.55 | | 4.79 | 1.50 | | 1.83 |  |
| K00248\|Clostridium baratii | 1.11 | 1.94 | 1.72 | | 1.65 | 1.79 | | 3.02 |  |
| K00248\|Thermoanaerobacterium thermosaccharolyticum DSM 571 | 1.10 | 2.05 | 1.87 | | 2.02 | 1.92 | | 1.60 |  |
| K00248\|Clostridium sporogenes | 1.40 | 1.40 | 1.28 | | 2.17 | 1.14 | | 0.96 |  |
| K00248\|Natranaerobius thermophilus | 0.93 | 1.34 | 1.53 | | 1.67 | 1.28 | | 1.53 |  |
| K00248\|Clostridium cellulovorans | 1.25 | 1.31 | 1.71 | | 1.01 | 1.56 | | 1.26 |  |
| K00248\|Carboxydothermus hydrogenoformans | 0.69 | 0.85 | 1.21 | | 1.27 | 1.07 | | 1.03 |  |
| K00248\|All other taxa | 5.45 | 5.22 | 5.72 | | 8.15 | 5.99 | | 7.10 |  |
| **EC:2.8.3.8** |  |  |  | |  |  | |  |  |
| K01035\|Eubacterium sulci | 18.50 | 22.25 | 21.20 | | 28.66 | 22.10 | | 26.94 |  |
| K01035\|Odoribacter splanchnicus | 23.12 | 23.57 | 21.81 | | 19.65 | 20.64 | | 21.34 |  |
| K01034\|Eubacterium sulci | 20.82 | 17.33 | 23.48 | | 24.19 | 18.48 | | 22.23 |  |
| K01034\|Megasphaera elsdenii | 0.00 | 11.64 | 0.00 | | 31.18 | 18.22 | | 0.00 |  |
| K01035\|Megasphaera elsdenii | 0.27 | 11.15 | 0.11 | | 27.93 | 13.26 | | 0.37 |  |
| K01035\|Clostridium sp. SY8519 | 5.46 | 4.82 | 6.03 | | 8.17 | 5.12 | | 7.17 |  |
| K01035\|Flavonifractor plautii | 3.65 | 4.10 | 3.06 | | 5.29 | 4.41 | | 2.94 |  |
| K01035\|Anaerostipes hadrus | 0.82 | 3.26 | 0.78 | | 6.44 | 4.00 | | 2.03 |  |
| K01035\|Megasphaera sp. AJH120 | 0.08 | 2.62 | 0.22 | | 7.86 | 3.65 | | 0.04 |  |
| K01035\|Alkaliphilus metalliredigens | 1.94 | 2.73 | 2.08 | | 2.78 | 2.99 | | 1.75 |  |
| K01035\|Clostridium argentinense | 2.49 | 2.68 | 2.09 | | 3.27 | 1.69 | | 1.71 |  |
| K01035\|Intestinimonas butyriciproducens | 1.60 | 2.05 | 1.35 | | 2.35 | 1.68 | | 1.91 |  |
| K01034\|Clostridium sp. SY8519 | 1.60 | 0.88 | 2.45 | | 1.10 | 1.75 | | 1.61 |  |
| K01035\|Vibrio mediterranei | 1.23 | 1.04 | 1.76 | | 0.99 | 1.43 | | 0.76 |  |
| K01035\|Bacteroidales bacterium CF | 0.97 | 1.06 | 1.74 | | 1.03 | 1.31 | | 1.08 |  |
| K01035\|All other taxa | 9.24 | 9.90 | 10.89 | | 12.44 | 11.91 | | 8.46 |  |
| **EC:2.3.1.19** |  |  |  | |  |  | |  |  |
| K00634\|Prevotella ruminicola | 51.41 | 51.09 | 47.56 | | 53.23 | 52.00 | | 35.49 |  |
| K00634\|Butyrivibrio proteoclasticus | 19.02 | 16.36 | 17.40 | | 18.28 | 13.66 | | 18.21 |  |
| K00634\|All other taxa | 0.75 | 5.77 | 2.18 | | 2.99 | 3.77 | | 5.83 |  |
| **EC:2.7.2.7** |  |  |  | |  |  | |  |  |
| K00929\|Prevotella ruminicola | 18.72 | 18.55 | 18.30 | | 20.68 | 21.89 | | 13.23 |  |
| K00929\|Coprococcus sp. ART55/1 | 6.79 | 21.84 | 7.47 | | 12.09 | 8.61 | | 15.76 |  |
| K00929\|Butyrivibrio proteoclasticus | 9.09 | 6.82 | 7.02 | | 5.88 | 7.36 | | 6.62 |  |
| K00929\|Butyrivibrio hungatei | 9.17 | 7.14 | 6.87 | | 5.36 | 7.25 | | 6.39 |  |
| K00929\|Clostridium butyricum | 0.24 | 5.32 | 1.67 | | 1.79 | 4.19 | | 5.49 |  |
| K00929\|Clostridium sp. SY8519 | 2.45 | 1.17 | 1.84 | | 1.44 | 2.60 | | 3.27 |  |
| K00929\|Mogibacterium diversum | 1.84 | 1.44 | 2.21 | | 1.48 | 1.79 | | 2.49 |  |
| K00929\|Clostridium cellulovorans | 0.92 | 3.74 | 1.01 | | 1.60 | 1.15 | | 1.84 |  |
| K00929\|Clostridium saccharobutylicum | 0.25 | 2.10 | 0.70 | | 1.04 | 1.57 | | 2.47 |  |
| K00929\|Clostridium saccharoperbutylacetonicum | 0.12 | 1.46 | 0.69 | | 0.85 | 1.46 | | 1.91 |  |
| K00929\|All other taxa | 5.87 | 11.34 | 7.25 | | 7.17 | 8.43 | | 8.85 |  |

**Table S21:** Taxonomy (cpm; copies per million) associated with the propionate pathway in metagenomics in cows supplemented with 3-nitrooxypropanol (3-NOP) compared to control cows at weeks 4, 8, and 12.

|  | **W4** | | **W8** | | **W12** | |
| --- | --- | --- | --- | --- | --- | --- |
|  | **Control** | **3-NOP** | **Control** | **3-NOP** | **Control** | **3-NOP** |
| **EC: 2.8.3.1** |  |  |  |  |  |  |
| K01026\|Blautia obeum | 24.45 | 18.78 | 25.93 | 19.57 | 18.31 | 23.96 |
| K01026\|Ruminococcus torques | 20.84 | 19.57 | 23.62 | 17.44 | 16.95 | 21.86 |
| K01026\|Coprococcus sp. ART55/1 | 15.50 | 13.53 | 14.43 | 12.63 | 12.97 | 10.51 |
| K01026\|Clostridium drakei | 1.40 | 1.57 | 1.52 | 1.69 | 1.41 | 1.85 |
| K01026\|Clostridium butyricum | 0.94 | 1.21 | 1.17 | 1.08 | 1.12 | 0.95 |
| K01026\|Clostridium perfringens 13 | 1.54 | 0.60 | 0.81 | 1.44 | 0.67 | 1.07 |
| All other taxa | 6.53 | 7.77 | 6.85 | 11.25 | 6.44 | 7.47 |
| **EC: 6.2.1.5** |  |  |  |  |  |  |
| K01902\|Methanobrevibacter ruminantium M1 | 47.79 | 40.16 | 51.13 | 36.72 | 35.37 | 41.12 |
| K01902\|Methanobrevibacter olleyae YLM1 | 42.50 | 33.78 | 42.39 | 31.10 | 31.43 | 33.87 |
| K01903\|Methanobrevibacter ruminantium M1 | 55.91 | 43.04 | 41.38 | 19.27 | 26.50 | 22.04 |
| K01903\|Methanobrevibacter olleyae YLM1 | 44.54 | 34.87 | 29.21 | 12.15 | 20.16 | 16.47 |
| K01902\|Methanobrevibacter sp. YE315 | 30.88 | 15.29 | 26.20 | 12.60 | 18.73 | 19.82 |
| K01903\|Methanobrevibacter sp. YE315 | 17.13 | 9.67 | 16.45 | 3.98 | 15.12 | 7.36 |
| K01902\|Methanobrevibacter millerae SM9 | 8.78 | 8.10 | 7.20 | 3.86 | 6.54 | 7.23 |
| K01903\|Methanobrevibacter millerae SM9 | 8.72 | 4.55 | 6.56 | 3.86 | 7.77 | 7.26 |
| K01902\|Methanobrevibacter smithii ATCC 35061 | 6.34 | 4.88 | 3.75 | 1.41 | 2.46 | 1.87 |
| K01902\|Selenomonas ruminantium | 4.59 | 2.31 | 2.73 | 3.33 | 4.56 | 3.08 |
| K01903\|Selenomonas ruminantium | 4.46 | 3.05 | 2.06 | 4.33 | 3.20 | 3.34 |
| K01902\|Prevotella denticola | 0.62 | 5.31 | 3.29 | 2.54 | 6.79 | 1.18 |
| K01902\|Methanosphaera sp. BMS | 4.16 | 2.62 | 2.38 | 2.36 | 2.86 | 3.38 |
| K01903\|Methanosphaera sp. BMS | 3.38 | 2.69 | 3.90 | 2.19 | 2.82 | 2.56 |
| K01902\|Prevotella fusca | 0.52 | 4.57 | 2.17 | 1.52 | 5.15 | 0.79 |
| K01902\|Prevotella melaninogenica | 0.51 | 3.17 | 1.74 | 1.26 | 3.49 | 0.78 |
| K01903\|Bifidobacterium pseudolongum | 2.08 | 3.08 | 1.74 | 1.59 | 0.67 | 1.16 |
| K01902\|Methanosphaera stadtmanae DSM 3091 | 1.17 | 1.82 | 1.73 | 2.52 | 2.07 | 1.00 |
| K01902\|Prevotella jejuni | 0.51 | 2.52 | 1.49 | 1.31 | 3.10 | 0.70 |
| K01903\|Methanobrevibacter sp. AbM4 | 2.39 | 1.78 | 1.81 | 1.32 | 1.20 | 0.64 |
| K01903\|Methanosphaera stadtmanae DSM 3091 | 1.15 | 1.33 | 1.46 | 1.64 | 1.41 | 1.71 |
| K01903\|Selenomonas sputigena | 1.88 | 1.21 | 0.78 | 1.72 | 1.78 | 1.15 |
| K01902\|Bifidobacterium pseudolongum | 1.55 | 2.37 | 1.11 | 1.72 | 0.40 | 1.20 |
| K01902\|Methanobrevibacter sp. AbM4 | 0.09 | 0.33 | 1.04 | 2.46 | 2.41 | 1.39 |
| K01903\|Selenomonas sp. oral taxon 920 | 1.26 | 0.88 | 0.78 | 1.45 | 1.64 | 1.14 |
| K01903\|Selenomonas sp. oral taxon 478 | 1.11 | 0.81 | 0.78 | 1.35 | 1.45 | 1.14 |
| K01903\|Selenomonas sp. oral taxon 136 | 1.11 | 0.81 | 0.78 | 1.35 | 1.45 | 1.14 |
| K01903\|Prevotella fusca | 0.03 | 2.38 | 1.00 | 1.29 | 1.71 | 0.15 |
| K01903\|Methanobrevibacter smithii ATCC 35061 | 0.98 | 0.92 | 1.84 | 0.66 | 1.32 | 0.84 |
| K01903\|Prevotella denticola | 0.18 | 1.73 | 1.45 | 1.28 | 1.44 | 0.32 |
| All other taxa | 52.30 | 48.69 | 56.63 | 63.50 | 54.53 | 37.08 |
| **EC: 4.2.1.54** |  |  |  |  |  |  |
| K20626\|Coprococcus catus | 11.79 | 13.79 | 12.46 | 11.00 | 8.86 | 15.45 |
| K20627\|Coprococcus catus | 10.07 | 11.23 | 9.68 | 7.96 | 8.19 | 10.93 |
| K20627\|Megasphaera elsdenii | 0.61 | 2.07 | 0.00 | 2.06 | 1.49 | 0.00 |
| K20626\|Megasphaera elsdenii | 0.02 | 0.54 | 0.00 | 1.18 | 0.40 | 0.00 |
| All other taxa | 0.40 | 1.20 | 0.43 |  | 0.64 | 1.03 |
| **EC: 5.4.99.2** |  |  |  |  |  |  |
| K01847\|Prevotella ruminicola | 362.40 | 400.75 | 361.44 | 401.36 | 398.95 | 359.16 |
| K01847\|Bacteroides dorei HS1_L_1_B_010 | 27.74 | 33.58 | 27.86 | 30.20 | 30.76 | 28.38 |
| K01847\|Bacteroides vulgatus | 27.64 | 31.89 | 27.46 | 30.32 | 30.76 | 27.81 |
| K01847\|Bacteroides salanitronis | 24.47 | 26.71 | 24.12 | 26.53 | 26.52 | 24.44 |
| K01847\|Bacteroides ovatus | 20.06 | 23.36 | 20.44 | 23.52 | 23.22 | 21.46 |
| K01847\|Parabacteroides distasonis | 20.03 | 23.81 | 19.94 | 22.97 | 22.53 | 21.23 |
| K01847\|Parabacteroides sp. CT06 | 19.88 | 23.62 | 19.77 | 22.82 | 22.34 | 21.02 |
| K01847\|Bacteroides xylanisolvens | 19.45 | 22.40 | 19.52 | 22.55 | 22.07 | 20.40 |
| K01847\|Bacteroides caccae | 18.59 | 21.41 | 18.10 | 21.55 | 20.82 | 19.75 |
| K01847\|Bacteroides fragilis YCH46 | 17.46 | 20.75 | 17.88 | 20.54 | 19.67 | 18.51 |
| K01847\|Bacteroides caecimuris | 17.60 | 20.36 | 17.36 | 20.14 | 19.66 | 18.71 |
| K01847\|Bacteroides zoogleoformans | 17.33 | 19.85 | 16.88 | 19.50 | 19.15 | 18.44 |
| K01847\|Proteiniphilum saccharofermentans | 16.22 | 19.59 | 16.58 | 19.28 | 18.57 | 16.79 |
| K01847\|Bacteroides heparinolyticus | 15.96 | 19.32 | 16.09 | 19.07 | 18.13 | 17.43 |
| K01847\|Porphyromonas gingivalis W83 | 15.70 | 19.04 | 15.89 | 18.54 | 17.91 | 16.96 |
| K01847\|Tannerella forsythia | 15.79 | 18.88 | 15.98 | 18.48 | 17.90 | 16.74 |
| K01847\|Bacteroides cellulosilyticus | 15.99 | 18.28 | 15.92 | 18.08 | 17.90 | 16.75 |
| K01847\|Bacteroides thetaiotaomicron VPI-5482 | 15.52 | 18.20 | 15.68 | 18.24 | 17.34 | 16.56 |
| K01847\|Bacteroides helcogenes | 15.11 | 17.83 | 15.37 | 18.15 | 17.39 | 16.60 |
| K01847\|Petrimonas mucosa | 15.23 | 18.55 | 15.14 | 17.76 | 16.83 | 16.04 |
| K01847\|Tannerella sp. oral taxon HOT-286 | 15.09 | 18.27 | 14.83 | 17.73 | 17.26 | 16.03 |
| K01847\|Barnesiella viscericola | 14.48 | 17.66 | 14.14 | 16.73 | 16.83 | 16.59 |
| K01847\|Muribaculum intestinale | 14.88 | 17.01 | 14.37 | 16.98 | 16.78 | 15.90 |
| K01847\|Porphyromonas crevioricanis | 13.32 | 16.20 | 13.20 | 15.49 | 14.86 | 13.96 |
| K01847\|Petrimonas sp. IBARAKI | 12.09 | 14.34 | 11.94 | 14.39 | 13.65 | 12.56 |
| K01847\|Candidatus Azobacteroides pseudotrichonymphae (Coptotermes formosanus) | 11.13 | 13.36 | 11.57 | 12.91 | 12.96 | 11.81 |
| K01847\|Porphyromonas asaccharolytica | 10.96 | 13.46 | 10.81 | 13.32 | 12.30 | 12.67 |
| K01847\|Paludibacter propionicigenes | 8.91 | 10.11 | 8.69 | 9.61 | 9.67 | 9.75 |
| K01847\|Fermentimonas caenicola | 8.74 | 9.93 | 8.52 | 9.83 | 9.58 | 9.38 |
| K01847\|Selenomonas ruminantium | 8.47 | 8.64 | 7.99 | 10.37 | 10.38 | 8.70 |
| K01847\|Selenomonas sputigena | 7.79 | 8.01 | 8.23 | 10.01 | 10.76 | 8.06 |
| K01847\|Odoribacter splanchnicus | 7.34 | 8.04 | 7.21 | 8.15 | 8.33 | 8.01 |
| K01849\|Veillonella parvula | 4.97 | 9.88 | 6.46 | 7.76 | 7.93 | 7.09 |
| K01849\|Veillonella atypica | 4.93 | 9.80 | 6.22 | 7.65 | 7.81 | 7.03 |
| K01849\|Veillonella rodentium | 4.93 | 9.80 | 6.22 | 7.65 | 7.81 | 7.03 |
| K01849\|Pelosinus sp. UFO1 | 8.75 | 7.86 | 5.46 | 8.37 | 6.26 | 6.52 |
| K01848\|Pelosinus fermentans | 6.19 | 8.01 | 5.74 | 7.65 | 8.11 | 6.87 |
| K01847\|Selenomonas sp. oral taxon 136 | 6.25 | 7.05 | 6.31 | 8.26 | 8.16 | 6.50 |
| K01847\|Selenomonas sp. oral taxon 478 | 6.22 | 7.03 | 6.33 | 8.27 | 8.17 | 6.46 |
| K01849\|Pelosinus fermentans | 8.71 | 7.77 | 5.10 | 7.75 | 6.12 | 5.94 |
| K01848\|Selenomonas ruminantium | 5.55 | 6.47 | 5.92 | 6.77 | 7.23 | 6.91 |
| K01847\|Selenomonas sp. oral taxon 920 | 5.58 | 6.09 | 5.32 | 7.23 | 7.38 | 5.79 |
| K01849\|Selenomonas sputigena | 3.26 | 6.97 | 4.74 | 5.85 | 6.77 | 5.38 |
| K01847\|Bacteroidales bacterium CF | 4.60 | 5.37 | 4.28 | 4.99 | 5.14 | 4.87 |
| K01848\|Megamonas hypermegale | 4.19 | 4.95 | 4.21 | 4.87 | 5.33 | 4.36 |
| K01848\|Pelosinus sp. UFO1 | 4.58 | 4.96 | 3.58 | 5.08 | 5.14 | 4.47 |
| K01849\|Megamonas hypermegale | 4.21 | 5.84 | 2.57 | 4.51 | 4.54 | 3.91 |
| K01847\|Marinifilaceae bacterium SPP2 | 3.41 | 4.49 | 3.46 | 4.20 | 4.43 | 3.70 |
| K01849\|Selenomonas ruminantium | 4.04 | 4.66 | 2.56 | 4.93 | 3.37 | 4.06 |
| K01847\|Alistipes finegoldii | 4.17 | 3.65 | 3.18 | 3.62 | 4.03 | 4.04 |
| K01847\|Veillonella atypica | 3.20 | 3.61 | 3.24 | 3.64 | 4.28 | 3.23 |
| K01847\|Veillonella rodentium | 3.12 | 3.36 | 3.18 | 3.36 | 4.00 | 3.12 |
| K01847\|Megamonas hypermegale | 2.86 | 3.48 | 3.38 | 3.46 | 3.79 | 3.11 |
| K01847\|Veillonella parvula | 3.08 | 3.43 | 3.02 | 3.36 | 3.95 | 3.11 |
| K01847\|Emticicia oligotrophica | 2.90 | 3.25 | 2.97 | 2.92 | 3.06 | 2.88 |
| K01847\|Alkalitalea saponilacus | 2.39 | 3.13 | 2.69 | 2.82 | 2.88 | 3.34 |
| K01847\|Alistipes shahii | 3.13 | 2.36 | 2.62 | 2.33 | 2.71 | 2.89 |
| K01848\|Veillonella atypica | 2.18 | 2.76 | 2.18 | 2.94 | 3.06 | 2.85 |
| K01848\|Veillonella rodentium | 2.18 | 2.76 | 2.13 | 2.94 | 3.06 | 2.63 |
| K01848\|Veillonella parvula | 2.14 | 2.72 | 2.10 | 2.87 | 2.91 | 2.48 |
| K01847\|Runella slithyformis | 2.58 | 2.66 | 2.38 | 2.38 | 2.48 | 2.38 |
| K01847\|Runella sp. HYN0085 | 2.57 | 2.65 | 2.37 | 2.37 | 2.47 | 2.38 |
| K01848\|Selenomonas sp. oral taxon 478 | 2.32 | 2.39 | 1.77 | 2.70 | 2.62 | 2.29 |
| K01848\|Selenomonas sp. oral taxon 136 | 2.32 | 2.38 | 1.77 | 2.68 | 2.61 | 2.29 |
| K01848\|Selenomonas sp. oral taxon 920 | 2.35 | 2.35 | 1.77 | 2.65 | 2.56 | 2.19 |
| K01847\|Draconibacterium orientale | 2.35 | 2.32 | 2.45 | 2.37 | 2.10 | 2.20 |
| K01847\|Intestinimonas butyriciproducens | 2.19 | 2.16 | 2.23 | 2.27 | 2.48 | 2.07 |
| K01847\|Hymenobacter sp. DG25A | 2.13 | 2.51 | 2.15 | 2.31 | 2.03 | 2.17 |
| K01847\|Mucinivorans hirudinis | 1.83 | 2.28 | 1.75 | 2.09 | 2.11 | 2.41 |
| K01848\|Selenomonas sputigena | 1.54 | 2.02 | 1.77 | 2.38 | 2.36 | 2.11 |
| K01847\|Leptospira biflexa serovar Patoc Patoc 1 (Paris) | 1.63 | 2.05 | 1.66 | 1.96 | 2.00 | 1.95 |
| K01847\|Hymenobacter sp. DG25B | 1.79 | 2.07 | 1.81 | 1.84 | 1.78 | 1.79 |
| K01847\|Flammeovirga sp. MY04 | 1.63 | 1.93 | 1.68 | 1.86 | 1.83 | 1.58 |
| K01849\|Selenomonas sp. oral taxon 920 | 2.32 | 2.06 | 1.15 | 1.92 | 1.93 | 1.02 |
| K01847\|Pelobacter propionicus | 1.48 | 1.85 | 1.40 | 1.53 | 1.73 | 1.60 |
| K01847\|Pelosinus fermentans | 1.57 | 1.57 | 1.72 | 1.41 | 2.04 | 1.26 |
| K01847\|Bacillus simplex | 1.34 | 1.43 | 1.32 | 1.40 | 1.73 | 1.49 |
| K01849\|Ferroglobus placidus DSM 10642 | 1.24 | 1.82 | 1.05 | 1.49 | 1.85 | 1.20 |
| K01847\|Cloacibacillus porcorum | 1.16 | 1.22 | 1.17 | 1.34 | 2.00 | 1.25 |
| K01849\|Selenomonas sp. oral taxon 478 | 1.53 | 1.80 | 0.85 | 1.59 | 1.37 | 1.00 |
| K01849\|Selenomonas sp. oral taxon 136 | 1.53 | 1.73 | 0.85 | 1.59 | 1.37 | 1.00 |
| K01847\|Siansivirga zeaxanthinifaciens | 1.22 | 1.88 | 1.19 | 1.19 | 1.33 | 1.17 |
| K01847\|Lacunisphaera limnophila | 1.14 | 1.28 | 0.81 | 1.15 | 1.79 | 1.58 |
| K01849\|Geoglobus ahangari 234 | 0.91 | 1.47 | 1.21 | 1.18 | 1.50 | 1.14 |
| K01847\|Flavobacterium branchiophilum | 1.02 | 1.23 | 1.17 | 1.12 | 1.22 | 1.54 |
| K01847\|Leptospira interrogans serovar Lai 56601 | 1.16 | 1.07 | 1.25 | 1.24 | 1.11 | 1.21 |
| K01847\|Leptospira borgpetersenii JB197 | 1.10 | 1.26 | 1.10 | 1.21 | 1.13 | 1.16 |
| K01847\|Fretibacterium fastidiosum | 1.11 | 1.17 | 1.08 | 1.18 | 1.34 | 1.00 |
| K01847\|Leptospira mayottensis | 1.09 | 1.20 | 1.17 | 1.08 | 1.00 | 1.06 |
| K01847\|Bacillus kochii | 0.87 | 1.12 | 0.70 | 0.73 | 1.37 | 1.36 |
| K01849\|Flavonifractor plautii | 1.00 | 1.31 | 0.85 | 1.26 | 0.95 | 0.66 |
| K01849\|Archaeoglobus fulgidus DSM 4304 (VC-16) | 0.65 | 1.46 | 0.84 | 1.12 | 1.15 | 0.83 |
| All other taxa | 79.49 | 85.14 | 87.95 | 90.94 | 92.30 | 90.50 |
| **EC: 1.3.8.7** |  |  |  |  |  |  |
| K00249\|Corynebacterium terpenotabidum | 0.08 | 0.00 | 0.00 | 0.00 | 0.03 | 1.05 |
| K00249\|Corynebacterium lactis | 0.18 | 0.01 | 0.26 | 0.00 | 0.29 | 0.30 |
| K00249\|Corynebacterium sp. ATCC 6931 | 0.18 | 0.01 | 0.26 | 0.00 | 0.29 | 0.14 |
| K00249\|Corynebacterium variabile | 0.08 | 0.00 | 0.00 | 0.00 | 0.03 | 0.73 |
| K00249\|Corynebacterium jeikeium | 0.03 | 0.01 | 0.04 | 0.09 | 0.05 | 0.42 |
| All other taxa | 1.52 | 1.46 | 1.92 |  | 1.12 | 1.73 |
| **EC: 5.1.99.1** |  |  |  |  |  |  |
| K05606\|Prevotella ruminicola | 448.99 | 470.25 | 466.82 | 498.34 | 467.05 | 444.25 |
| K05606\|Muribaculum intestinale | 10.57 | 19.02 | 7.44 | 10.73 | 13.44 | 15.96 |
| K05606\|Barnesiella viscericola | 10.30 | 10.05 | 10.43 | 8.26 | 10.75 | 12.62 |
| K05606\|Bacteroides salanitronis | 5.32 | 6.27 | 10.01 | 7.00 | 7.20 | 8.04 |
| K05606\|Bacteroides cellulosilyticus | 5.69 | 7.07 | 6.59 | 5.94 | 6.61 | 8.82 |
| K05606\|Bacteroides fragilis YCH46 | 5.53 | 7.38 | 7.03 | 6.27 | 6.71 | 5.50 |
| K05606\|Bacteroides caecimuris | 5.15 | 6.54 | 6.84 | 6.46 | 6.11 | 4.80 |
| K05606\|Bacteroides caccae | 5.15 | 6.65 | 6.79 | 6.17 | 6.11 | 4.64 |
| K05606\|Bacteroides ovatus | 5.15 | 6.65 | 6.79 | 6.17 | 6.11 | 4.64 |
| K05606\|Bacteroides thetaiotaomicron VPI-5482 | 5.15 | 6.65 | 6.79 | 6.17 | 6.11 | 4.64 |
| K05606\|Bacteroides xylanisolvens | 5.15 | 6.65 | 6.79 | 6.17 | 6.11 | 4.64 |
| K05606\|Bacteroides zoogleoformans | 4.05 | 6.71 | 5.87 | 6.02 | 6.50 | 4.73 |
| K05606\|Porphyromonas crevioricanis | 4.72 | 6.03 | 4.50 | 5.01 | 6.17 | 6.08 |
| K05606\|Bacteroides helcogenes | 3.74 | 5.32 | 4.78 | 6.51 | 4.59 | 5.54 |
| K05606\|Bacteroides dorei HS1_L_1_B_010 | 2.66 | 6.31 | 5.03 | 3.06 | 7.68 | 5.64 |
| K05606\|Coprococcus catus | 2.37 | 4.11 | 6.05 | 3.95 | 2.64 | 5.21 |
| K05606\|Bacteroides heparinolyticus | 2.53 | 2.95 | 2.03 | 3.90 | 2.24 | 3.64 |
| K05606\|Selenomonas ruminantium | 3.37 | 4.29 | 2.81 | 4.03 | 1.96 | 0.14 |
| K05606\|Paludibacter propionicigenes | 2.55 | 2.91 | 2.81 | 1.94 | 1.73 | 2.41 |
| K05606\|Flavonifractor plautii | 2.16 | 1.70 | 1.57 | 3.65 | 2.27 | 0.65 |
| K05606\|Tannerella sp. oral taxon HOT-286 | 3.15 | 1.55 | 2.06 | 0.62 | 1.13 | 2.31 |
| K05606\|Bacteroidales bacterium CF | 1.96 | 0.86 | 0.37 | 2.08 | 0.30 | 2.84 |
| All other taxa | 8.96 | 11.21 | 7.74 | 8.42 | 9.89 | 9.01 |
| **EC :2.8.3.1** |  |  |  |  |  |  |
| K010261\|Blautia obeum | 24.45 | 18.78 | 25.93 | 19.57 | 18.31 | 23.96 |
| K010261\|Ruminococcus torques | 20.84 | 19.57 | 23.62 | 17.44 | 16.95 | 21.86 |
| K010261\|Coprococcus sp. ART55/1 | 15.50 | 13.53 | 14.43 | 12.63 | 12.97 | 10.51 |
| K010261\|Clostridium drakei | 1.40 | 1.57 | 1.52 | 1.69 | 1.41 | 1.85 |
| K010261\|Clostridium butyricum | 0.94 | 1.21 | 1.17 | 1.08 | 1.12 | 0.95 |
| K010261\|Clostridium perfringens 13 | 1.54 | 0.60 | 0.81 | 1.44 | 0.67 | 1.07 |
| All other taxa | 6.53 | 7.77 | 6.85 | 11.25 | 6.44 | 7.47 |
| **EC: 4.1.1.-** |  |  |  |  |  |  |
| K11264\|Citrobacter amalonaticus FDAARGOS_165 | 0.01 | 0.00 | 0.00 | 0.00 | 0.00 | 0.00 |
| K11264\|Citrobacter braakii | 0.01 | 0.00 | 0.00 | 0.00 | 0.00 | 0.00 |
| K11264\|Citrobacter sp. CFNIH10 | 0.01 | 0.00 | 0.00 | 0.00 | 0.00 | 0.00 |
| K11264\|Citrobacter youngae | 0.01 | 0.00 | 0.00 | 0.00 | 0.00 | 0.00 |
| K11264\|Enterobacter lignolyticus SCF1 | 0.01 | 0.00 | 0.00 | 0.00 | 0.00 | 0.00 |
| K11264\|Escherichia coli K-12 MG1655 | 0.01 | 0.00 | 0.00 | 0.00 | 0.00 | 0.00 |
| K11264\|Escherichia marmotae | 0.01 | 0.00 | 0.00 | 0.00 | 0.00 | 0.00 |
| K11264\|Salmonella bongori NCTC 12419 | 0.01 | 0.00 | 0.00 | 0.00 | 0.00 | 0.00 |
| K11264\|Salmonella enterica subsp. arizonae | 0.01 | 0.00 | 0.00 | 0.00 | 0.00 | 0.00 |
| K11264\|Shigella sp. PAMC 28760 | 0.01 | 0.00 | 0.00 | 0.00 | 0.00 | 0.00 |
| K11264\|Citrobacter freundii | 0.01 | 0.00 | 0.00 | 0.00 | 0.00 | 0.00 |
| K11264\|Citrobacter sp. CRE-46 | 0.01 | 0.00 | 0.00 | 0.00 | 0.00 | 0.00 |
| K11264\|Citrobacter sp. FDAARGOS_156 | 0.01 | 0.00 | 0.00 | 0.00 | 0.00 | 0.00 |
| K11264\|Escherichia albertii | 0.01 | 0.00 | 0.00 | 0.00 | 0.00 | 0.00 |
| K11264\|Klebsiella michiganensis KCTC 1686 | 0.01 | 0.00 | 0.00 | 0.00 | 0.00 | 0.00 |
| K11264\|Klebsiella oxytoca KONIH1 | 0.01 | 0.00 | 0.00 | 0.00 | 0.00 | 0.00 |
| K11264\|Enterobacter sp. R4-368 | 0.01 | 0.00 | 0.00 | 0.00 | 0.00 | 0.00 |
| K11264\|Kosakonia sacchari | 0.01 | 0.00 | 0.00 | 0.00 | 0.00 | 0.00 |
| K11264\|Shigella boydii Sb227 (serotype 4) | 0.01 | 0.00 | 0.00 | 0.00 | 0.00 | 0.00 |
| K11264\|Shigella flexneri 301 (serotype 2a) | 0.01 | 0.00 | 0.00 | 0.00 | 0.00 | 0.00 |
| K11264\|Citrobacter werkmanii | 0.01 | 0.00 | 0.00 | 0.00 | 0.00 | 0.00 |
| K11264\|Shigella sonnei Ss046 | 0.01 | 0.00 | 0.00 | 0.00 | 0.00 | 0.00 |
| K11264\|Kosakonia oryzae | 0.01 | 0.00 | 0.00 | 0.00 | 0.00 | 0.00 |
| K11264\|Kosakonia radicincitans | 0.01 | 0.00 | 0.00 | 0.00 | 0.00 | 0.00 |
| K11264\|Yersinia enterocolitica subsp. enterocolitica 8081 (serotype 0:8) | 0.00 | 0.00 | 0.00 | 0.00 | 0.00 | 0.00 |

**Table S22:** Taxonomy (cpm; copies per million) associated with the propionate pathway in metatranscriptomics in cows supplemented with 3-nitrooxypropanol (3-NOP) compared to control cows at weeks 4, 8, and 12

|  | **W4** |  | **W8** |  | **W12** |  |
| --- | --- | --- | --- | --- | --- | --- |
|  | **Control** | **3-NOP** | **Control** | **3-NOP** | **Control** | **3-NOP** |
| **EC:2.8.3.1** |  |  |  |  |  |  |
| K01026\|Coprococcus catus | 5.80 | 3.20 | 5.30 | 4.75 | 3.71 | 4.69 |
| K01026\|Clostridium drakei | 2.45 | 2.21 | 2.59 | 2.96 | 2.43 | 2.81 |
| K01026\|Eubacterium limosum | 1.65 | 1.16 | 3.34 | 2.89 | 2.42 | 3.24 |
| K01026\|Blautia obeum | 2.55 | 1.23 | 3.17 | 2.33 | 2.28 | 3.09 |
| K01026\|Megasphaera elsdenii | 0.03 | 0.87 | 0.13 | 11.54 | 1.25 | 0.09 |
| K01026\|Clostridium butyricum | 1.82 | 1.43 | 2.31 | 2.87 | 2.23 | 2.69 |
| K01026\|Ruminococcus torques | 2.37 | 1.47 | 2.34 | 1.76 | 1.90 | 2.31 |
| K01026\|Anaerotignum propionicum | 1.53 | 1.32 | 1.98 | 1.92 | 1.58 | 2.13 |
| K01026\|Desulfitobacterium metallireducens | 1.00 | 1.18 | 0.97 | 1.40 | 1.12 | 1.58 |
| K01026\|Clostridium perfringens 13 | 1.02 | 0.74 | 1.30 | 1.16 | 1.06 | 1.33 |
| K01026\|Clostridium tetani E88 | 0.99 | 0.84 | 1.28 | 0.99 | 1.02 | 1.31 |
| K01026\|All others | 8.45 | 5.91 | 11.35 | 10.57 | 10.10 | 12.00 |
| **EC:4.2.1.54** |  |  |  |  |  |  |
| K20626\|Coprococcus catus | 336.37 | 308.38 | 309.42 | 316.77 | 264.04 | 324.18 |
| K20626\|Desulfosporosinus meridiei | 8.19 | 8.37 | 9.33 | 7.99 | 6.95 | 8.33 |
| K20626\|Desulfosporosinus orientis | 4.46 | 5.16 | 5.82 | 4.58 | 3.73 | 4.62 |
| K20626\|Clostridium botulinum BKT015925 | 4.24 | 3.68 | 5.20 | 3.71 | 3.27 | 3.61 |
| K20626\|Clostridium novyi | 4.23 | 3.64 | 5.18 | 3.72 | 3.26 | 3.67 |
| K20626\|Megasphaera elsdenii | 1.60 | 0.99 | 1.91 | 1.78 | 1.39 | 1.19 |
| K20626\|beta-lactamase class C ACC-4 | 0.33 | 0.37 | 0.37 | 0.48 | 0.39 | 0.39 |
| K20627\|Coprococcus catus | 177.40 | 148.73 | 166.04 | 155.09 | 128.45 | 158.41 |
| K20627\|Clostridium novyi | 3.33 | 3.49 | 4.77 | 2.91 | 2.55 | 3.83 |
| K20627\|Clostridium botulinum BKT015925 | 1.59 | 1.67 | 1.90 | 2.20 | 1.30 | 1.66 |
| K20627\|Desulfosporosinus orientis | 1.26 | 1.44 | 1.39 | 0.73 | 0.65 | 0.77 |
| K20627\|Desulfosporosinus meridiei | 0.22 | 0.18 | 0.23 | 0.11 | 0.15 | 0.24 |
| K20627\|Megasphaera elsdenii | 0.15 | 0.05 | 0.21 | 0.33 | 0.10 | 0.21 |
| K20627\|beta-lactamase class C ACC-4 | 0.02 | 0.23 | 0.02 | 0.12 | 0.07 | 0.25 |
| **EC: 1.3.8.7** |  |  |  |  |  |  |
| K00249\|Oceanobacillus iheyensis | 0.06 | 0.00 | 0.10 | 0.00 | 0.03 | 0.00 |
| K00249\|Corynebacterium terpenotabidum | 0.01 | 0.00 | 0.00 | 0.00 | 0.02 | 0.14 |
| K00249\|Corynebacterium variabile | 0.01 | 0.00 | 0.00 | 0.00 | 0.02 | 0.13 |
| K00249\|Corynebacterium jeikeium | 0.00 | 0.00 | 0.00 | 0.00 | 0.01 | 0.10 |
| K00249\|Bacillus cereus E33L | 0.01 | 0.00 | 0.00 | 0.00 | 0.00 | 0.08 |
| K00249\|Geobacillus thermodenitrificans | 0.02 | 0.00 | 0.03 | 0.02 | 0.01 | 0.01 |
| K00249\|Corynebacterium kroppenstedtii | 0.01 | 0.00 | 0.01 | 0.00 | 0.01 | 0.05 |
| K00249\|Marinithermus hydrothermalis | 0.00 | 0.00 | 0.00 | 0.00 | 0.00 | 0.06 |
| K00249\|All others | 0.08 | 0.00 | 0.02 | 0.05 | 0.12 | 0.17 |
| **EC:2.8.3.1** |  |  |  |  |  |  |
| K01026\|Coprococcus catus1 | 5.80 | 3.20 | 5.30 | 4.75 | 3.71 | 4.69 |
| K01026\|Clostridium drakei1 | 2.45 | 2.21 | 2.59 | 2.96 | 2.43 | 2.81 |
| K01026\|Eubacterium limosum1 | 1.65 | 1.16 | 3.34 | 2.89 | 2.42 | 3.24 |
| K01026\|Blautia obeum1 | 2.55 | 1.23 | 3.17 | 2.33 | 2.28 | 3.09 |
| K01026\|Megasphaera elsdenii1 | 0.03 | 0.87 | 0.13 | 11.54 | 1.25 | 0.09 |
| K01026\|Clostridium butyricum1 | 1.82 | 1.43 | 2.31 | 2.87 | 2.23 | 2.69 |
| K01026\|Ruminococcus torques1 | 2.37 | 1.47 | 2.34 | 1.76 | 1.90 | 2.31 |
| K01026\|Anaerotignum propionicum1 | 1.53 | 1.32 | 1.98 | 1.92 | 1.58 | 2.13 |
| K01026\|Desulfitobacterium metallireducens1 | 1.00 | 1.18 | 0.97 | 1.40 | 1.12 | 1.58 |
| K01026\|Clostridium perfringens 131 | 1.02 | 0.74 | 1.30 | 1.16 | 1.06 | 1.33 |
| K01026\|Clostridium tetani E881 | 0.99 | 0.84 | 1.28 | 0.99 | 1.02 | 1.31 |
| K01026\|All others | 8.45 | 5.91 | 11.35 | 10.57 | 10.10 | 12.00 |
| **EC:6.2.1.5** |  |  |  |  |  |  |
| K01902\|Methanobrevibacter olleyae YLM1 | 13.30 | 12.89 | 14.03 | 11.50 | 13.96 | 12.57 |
| K01902\|Methanobrevibacter ruminantium M1 | 13.55 | 13.32 | 15.14 | 10.11 | 14.12 | 10.31 |
| K01902\|Selenomonas ruminantium | 7.52 | 8.13 | 5.57 | 11.05 | 12.38 | 9.73 |
| K01902\|Methanobrevibacter sp. YE315 | 12.41 | 5.06 | 10.17 | 4.52 | 13.42 | 5.20 |
| K01902\|Prevotella denticola | 0.53 | 2.72 | 2.60 | 1.19 | 4.11 | 0.49 |
| K01902\|Methanobrevibacter millerae SM9 | 2.23 | 1.67 | 2.00 | 1.66 | 2.08 | 1.71 |
| K01902\|Cycloclasticus sp. P1 | 2.00 | 1.49 | 1.44 | 2.20 | 1.55 | 1.43 |
| K01902\|Cycloclasticus sp. PY97N | 2.00 | 1.49 | 1.44 | 2.20 | 1.55 | 1.43 |
| K01902\|Cycloclasticus zancles | 2.00 | 1.49 | 1.44 | 2.20 | 1.55 | 1.43 |
| K01902\|Microvirga ossetica | 1.81 | 1.33 | 1.29 | 1.92 | 1.38 | 1.16 |
| K01902\|Alkalitalea saponilacus | 1.90 | 1.64 | 1.16 | 1.75 | 0.69 | 1.16 |
| K01902\|Methanosphaera stadtmanae DSM 3091 | 0.96 | 1.40 | 0.94 | 1.39 | 1.77 | 1.59 |
| K01902\|Prevotella fusca | 0.37 | 1.55 | 1.40 | 0.73 | 2.97 | 0.32 |
| K01902\|Colwellia sp. MT41 | 1.58 | 0.80 | 1.26 | 1.35 | 1.36 | 0.83 |
| K01902\|Methanosphaera sp. BMS | 1.23 | 0.96 | 1.35 | 1.44 | 1.31 | 0.86 |
| K01902\|Glaciecola nitratireducens | 1.47 | 0.92 | 1.38 | 1.33 | 1.22 | 0.78 |
| K01902\|Desulfovibrio piger | 0.79 | 1.04 | 1.07 | 1.34 | 1.41 | 1.20 |
| K01902\|Selenomonas sp. oral taxon 478 | 0.45 | 1.00 | 0.80 | 1.64 | 1.48 | 1.11 |
| K01902\|Bifidobacterium pseudolongum | 0.73 | 1.70 | 1.66 | 0.75 | 0.47 | 1.00 |
| K01902\|All others | 76.93 | 49.99 | 70.97 | 73.28 | 58.73 | 48.62 |
| K01903\|Methanobrevibacter ruminantium M1 | 15.78 | 9.89 | 8.30 | 3.89 | 9.95 | 3.58 |
| K01903\|Methanobrevibacter olleyae YLM1 | 11.62 | 7.55 | 6.13 | 2.74 | 6.97 | 1.99 |
| K01903\|Selenomonas ruminantium | 4.31 | 5.71 | 3.66 | 7.03 | 6.83 | 5.55 |
| K01903\|Methanobrevibacter sp. YE315 | 7.49 | 3.29 | 5.27 | 2.44 | 6.23 | 1.88 |
| K01903\|Methanobrevibacter millerae SM9 | 2.98 | 2.04 | 2.70 | 2.95 | 3.22 | 2.28 |
| K01903\|Selenomonas sputigena | 1.39 | 1.45 | 1.36 | 2.20 | 1.63 | 1.45 |
| K01903\|Bifidobacterium pseudolongum | 1.25 | 3.69 | 1.57 | 0.81 | 0.46 | 1.48 |
| K01903\|Selenomonas sp. oral taxon 920 | 0.90 | 0.92 | 0.97 | 1.53 | 1.13 | 0.68 |
| K01903\|All others | 31.74 | 25.75 | 28.47 | 31.32 | 33.39 | 19.41 |
| **EC:5.4.99.2** |  |  |  |  |  |  |
| K01847\|Prevotella ruminicola | 482.86 | 479.99 | 440.21 | 455.95 | 466.05 | 347.70 |
| K01847\|Bacteroides dorei HS1_L_1_B_010 | 30.41 | 30.06 | 27.56 | 28.39 | 29.07 | 24.79 |
| K01847\|Bacteroides vulgatus | 30.44 | 29.98 | 27.16 | 28.34 | 29.09 | 24.87 |
| K01847\|Bacteroides salanitronis | 26.05 | 26.16 | 23.36 | 25.37 | 25.48 | 21.07 |
| K01847\|Parabacteroides distasonis | 22.26 | 22.25 | 19.75 | 20.91 | 20.86 | 17.89 |
| K01847\|Parabacteroides sp. CT06 | 22.03 | 22.05 | 19.55 | 20.70 | 20.64 | 17.69 |
| K01847\|Bacteroides ovatus | 19.77 | 19.43 | 17.98 | 19.11 | 19.23 | 16.08 |
| K01847\|Bacteroides xylanisolvens | 19.48 | 19.48 | 17.57 | 18.58 | 19.01 | 16.15 |
| K01847\|Proteiniphilum saccharofermentans | 19.45 | 19.16 | 17.27 | 18.71 | 18.85 | 14.91 |
| K01847\|Tannerella forsythia | 19.15 | 19.35 | 16.55 | 18.28 | 18.07 | 15.32 |
| K01847\|Bacteroides caccae | 18.27 | 18.10 | 16.51 | 17.19 | 17.75 | 15.11 |
| K01847\|Tannerella sp. oral taxon HOT-286 | 17.50 | 18.34 | 15.42 | 18.00 | 17.11 | 14.87 |
| K01847\|Bacteroides fragilis YCH46 | 17.63 | 17.53 | 15.82 | 16.86 | 16.90 | 14.33 |
| K01847\|Bacteroides caecimuris | 17.22 | 17.24 | 15.59 | 16.33 | 16.87 | 14.28 |
| K01847\|Petrimonas mucosa | 16.88 | 17.31 | 14.63 | 15.93 | 16.48 | 13.73 |
| K01847\|Porphyromonas gingivalis W83 | 16.15 | 16.63 | 14.61 | 17.10 | 15.72 | 13.35 |
| K01847\|Bacteroides zoogleoformans | 16.41 | 16.48 | 15.08 | 15.96 | 15.88 | 13.35 |
| K01847\|Bacteroides cellulosilyticus | 16.24 | 16.41 | 14.95 | 15.60 | 15.70 | 13.40 |
| K01847\|Barnesiella viscericola | 16.00 | 16.03 | 14.84 | 15.57 | 15.40 | 14.02 |
| K01847\|Muribaculum intestinale | 14.94 | 15.87 | 14.57 | 16.05 | 15.23 | 12.83 |
| K01847\|Bacteroides heparinolyticus | 15.42 | 15.45 | 14.26 | 15.50 | 14.99 | 12.77 |
| K01847\|Bacteroides helcogenes | 15.26 | 15.64 | 14.03 | 14.74 | 15.04 | 12.79 |
| K01847\|Porphyromonas crevioricanis | 15.15 | 15.47 | 13.72 | 15.32 | 14.68 | 12.45 |
| K01847\|Bacteroides thetaiotaomicron VPI-5482 | 15.07 | 15.15 | 13.82 | 14.67 | 14.74 | 12.55 |
| K01847\|Candidatus Azobacteroides pseudotrichonymphae (Coptotermes formosanus) | 14.97 | 14.56 | 12.98 | 14.18 | 14.02 | 11.50 |
| K01847\|Porphyromonas asaccharolytica | 12.69 | 12.25 | 11.64 | 12.13 | 13.18 | 11.10 |
| K01847\|Petrimonas sp. IBARAKI | 12.77 | 12.78 | 11.31 | 12.30 | 12.24 | 10.48 |
| K01847\|Paludibacter propionicigenes | 9.90 | 10.50 | 9.22 | 10.58 | 10.06 | 8.49 |
| K01847\|Fermentimonas caenicola | 9.40 | 9.33 | 8.36 | 8.58 | 9.15 | 7.85 |
| K01847\|Odoribacter splanchnicus | 8.87 | 9.11 | 8.44 | 9.11 | 8.63 | 7.15 |
| K01847\|Bacteroidales bacterium CF | 4.53 | 4.18 | 4.12 | 4.37 | 4.21 | 3.53 |
| K01847\|Marinifilaceae bacterium SPP2 | 3.53 | 3.90 | 3.24 | 3.56 | 3.50 | 3.16 |
| K01847\|Alistipes finegoldii | 3.07 | 3.06 | 3.33 | 3.67 | 2.98 | 2.61 |
| K01847\|Alistipes shahii | 2.78 | 2.87 | 3.14 | 3.02 | 2.89 | 2.47 |
| K01847\|Draconibacterium orientale | 2.80 | 2.54 | 2.57 | 3.01 | 2.74 | 2.11 |
| K01847\|Emticicia oligotrophica | 2.75 | 2.75 | 2.46 | 2.53 | 2.93 | 2.31 |
| K01847\|Alkalitalea saponilacus | 2.54 | 2.53 | 2.48 | 2.47 | 2.78 | 2.47 |
| K01847\|Selenomonas ruminantium | 2.90 | 2.71 | 2.24 | 2.58 | 2.71 | 1.95 |
| K01847\|Megamonas hypermegale | 2.35 | 2.31 | 2.29 | 2.33 | 2.37 | 1.75 |
| K01847\|Leptospira biflexa serovar Patoc Patoc 1 (Paris) | 2.02 | 2.14 | 1.93 | 2.13 | 2.12 | 1.87 |
| K01847\|Selenomonas sputigena | 2.17 | 1.99 | 1.73 | 1.98 | 2.02 | 1.58 |
| K01847\|Mucinivorans hirudinis | 1.62 | 2.05 | 1.78 | 1.95 | 1.82 | 1.52 |
| K01847\|Runella sp. HYN0085 | 1.80 | 1.69 | 1.82 | 1.78 | 1.86 | 1.47 |
| K01847\|Runella slithyformis | 1.80 | 1.68 | 1.83 | 1.77 | 1.87 | 1.46 |
| K01847\|Veillonella atypica | 1.73 | 1.74 | 1.69 | 1.83 | 1.64 | 1.30 |
| K01847\|Veillonella parvula | 1.74 | 1.74 | 1.59 | 1.87 | 1.58 | 1.29 |
| K01847\|Veillonella rodentium | 1.69 | 1.69 | 1.63 | 1.78 | 1.62 | 1.29 |
| K01847\|Intestinimonas butyriciproducens | 1.62 | 1.48 | 1.58 | 1.56 | 1.53 | 1.06 |
| K01847\|Selenomonas sp. oral taxon 478 | 1.79 | 1.41 | 1.41 | 1.45 | 1.54 | 1.19 |
| K01847\|Selenomonas sp. oral taxon 136 | 1.78 | 1.41 | 1.41 | 1.45 | 1.54 | 1.19 |
| K01847\|Hymenobacter sp. DG25A | 1.42 | 1.46 | 1.45 | 1.27 | 1.48 | 1.22 |
| K01847\|Selenomonas sp. oral taxon 920 | 1.55 | 1.30 | 1.28 | 1.30 | 1.37 | 1.06 |
| K01847\|Bacillus simplex | 1.27 | 1.17 | 1.30 | 1.35 | 1.31 | 1.20 |
| K01847\|Flavobacterium branchiophilum | 1.13 | 1.27 | 1.26 | 1.27 | 1.36 | 1.02 |
| K01847\|Hymenobacter sp. DG25B | 1.28 | 1.21 | 1.21 | 1.10 | 1.20 | 1.04 |
| K01847\|Fretibacterium fastidiosum | 1.19 | 1.27 | 1.22 | 1.21 | 1.21 | 0.91 |
| K01847\|Flammeovirga sp. MY04 | 1.19 | 1.17 | 0.94 | 1.12 | 1.07 | 0.83 |
| K01847\|Leptospira borgpetersenii JB197 | 1.06 | 1.03 | 1.08 | 1.01 | 1.15 | 0.78 |
| K01847\|All others | 61.60 | 57.60 | 64.55 | 61.07 | 63.26 | 52.07 |
| K01848\|Selenomonas ruminantium | 71.08 | 72.18 | 76.98 | 90.14 | 100.57 | 69.85 |
| K01848\|Pelosinus fermentans | 65.60 | 67.90 | 63.86 | 66.61 | 85.45 | 51.43 |
| K01848\|Megamonas hypermegale | 47.09 | 45.08 | 50.04 | 53.05 | 62.99 | 40.45 |
| K01848\|Pelosinus sp. UFO1 | 35.95 | 36.08 | 35.73 | 38.78 | 46.77 | 28.17 |
| K01848\|Veillonella atypica | 22.19 | 23.83 | 28.22 | 29.29 | 31.62 | 20.98 |
| K01848\|Veillonella rodentium | 22.09 | 23.70 | 28.11 | 29.26 | 31.50 | 20.92 |
| K01848\|Veillonella parvula | 21.92 | 23.54 | 26.20 | 27.79 | 30.80 | 19.90 |
| K01848\|Selenomonas sp. oral taxon 478 | 21.48 | 22.06 | 22.56 | 24.07 | 28.21 | 17.80 |
| K01848\|Selenomonas sp. oral taxon 136 | 21.47 | 22.06 | 22.55 | 24.07 | 28.20 | 17.80 |
| K01848\|Selenomonas sp. oral taxon 920 | 18.46 | 17.81 | 19.51 | 22.10 | 24.24 | 15.33 |
| K01848\|Selenomonas sputigena | 14.16 | 13.41 | 18.66 | 20.52 | 19.56 | 12.85 |
| K01848\|Thermanaerovibrio acidaminovorans | 7.28 | 7.96 | 7.29 | 7.20 | 9.68 | 5.98 |
| K01848\|Paeniclostridium sordellii | 7.29 | 7.87 | 8.41 | 7.82 | 8.33 | 5.59 |
| K01848\|Melioribacter roseus | 6.97 | 7.80 | 7.59 | 6.86 | 9.40 | 5.51 |
| K01848\|Caldanaerobacter subterraneus subsp. tengcongensis | 2.96 | 3.14 | 3.77 | 4.92 | 5.26 | 3.71 |
| K01848\|Geosporobacter ferrireducens | 2.99 | 3.19 | 3.16 | 3.13 | 3.93 | 2.48 |
| K01848\|Deferribacter desulfuricans | 2.80 | 3.21 | 2.75 | 2.50 | 3.60 | 2.22 |
| K01848\|Clostridium drakei | 2.55 | 2.48 | 2.77 | 3.26 | 2.89 | 1.83 |
| K01848\|Clostridium scatologenes | 2.58 | 2.56 | 2.67 | 3.20 | 2.86 | 1.82 |
| K01848\|Ilyobacter polytropus | 2.46 | 2.31 | 2.52 | 2.94 | 3.37 | 2.00 |
| K01848\|Thermosipho melanesiensis | 2.32 | 2.60 | 2.42 | 2.25 | 3.06 | 2.01 |
| K01848\|Alkaliphilus oremlandii | 1.64 | 1.70 | 2.03 | 1.81 | 2.33 | 1.44 |
| K01848\|Acetoanaerobium sticklandii | 1.34 | 1.44 | 2.21 | 2.12 | 1.98 | 1.28 |
| K01848\|Thermoanaerobacter wiegelii | 1.51 | 1.60 | 1.68 | 1.83 | 2.05 | 1.22 |
| K01848\|Petrotoga mobilis | 1.17 | 1.60 | 1.72 | 1.80 | 2.27 | 1.31 |
| K01848\|Thermosulfidibacter takaii | 1.23 | 1.51 | 1.75 | 1.34 | 2.06 | 1.15 |
| K01848\|Flavonifractor plautii | 0.82 | 0.79 | 1.61 | 1.74 | 1.44 | 1.09 |
| K01848\|Kosmotoga olearia | 0.85 | 0.83 | 1.10 | 1.04 | 1.53 | 0.96 |
| K01848\|Thermosediminibacter oceani | 0.88 | 0.98 | 1.04 | 1.37 | 1.06 | 0.77 |
| K01848\|All others | 15.94 | 16.46 | 20.92 | 21.42 | 23.35 | 14.82 |
| K01849\|Pelosinus sp. UFO1 | 67.79 | 68.25 | 63.51 | 72.21 | 90.43 | 51.00 |
| K01849\|Pelosinus fermentans | 58.69 | 59.36 | 51.89 | 64.28 | 78.56 | 45.55 |
| K01849\|Veillonella parvula | 41.81 | 41.37 | 35.91 | 42.84 | 55.72 | 26.73 |
| K01849\|Veillonella atypica | 41.34 | 40.87 | 35.34 | 42.01 | 55.15 | 26.29 |
| K01849\|Veillonella rodentium | 41.25 | 40.78 | 35.21 | 41.95 | 55.02 | 26.20 |
| K01849\|Megamonas hypermegale | 41.75 | 34.78 | 37.87 | 43.05 | 50.19 | 31.54 |
| K01849\|Selenomonas ruminantium | 30.19 | 29.72 | 25.60 | 31.06 | 36.22 | 27.07 |
| K01849\|Selenomonas sputigena | 15.92 | 18.82 | 18.08 | 22.97 | 25.03 | 17.75 |
| K01849\|Selenomonas sp. oral taxon 920 | 7.65 | 6.05 | 7.31 | 9.38 | 10.88 | 4.87 |
| K01849\|Symbiobacterium thermophilum | 6.03 | 6.79 | 5.20 | 8.14 | 8.11 | 6.17 |
| K01849\|Selenomonas sp. oral taxon 478 | 4.85 | 4.26 | 5.31 | 7.67 | 7.71 | 4.67 |
| K01849\|Selenomonas sp. oral taxon 136 | 4.76 | 4.14 | 5.13 | 7.59 | 7.63 | 4.59 |
| K01849\|Archaeoglobus fulgidus DSM 4304 (VC-16) | 4.08 | 4.22 | 3.97 | 5.46 | 5.68 | 3.80 |
| K01849\|Caldanaerobacter subterraneus subsp. tengcongensis | 0.32 | 0.52 | 7.54 | 7.35 | 3.13 | 3.04 |
| K01849\|Ferroglobus placidus DSM 10642 | 3.35 | 3.69 | 3.19 | 3.68 | 4.17 | 2.77 |
| K01849\|Flavonifractor plautii | 3.22 | 2.60 | 3.23 | 3.30 | 4.19 | 2.39 |
| K01849\|Geoglobus ahangari 234 | 2.04 | 1.68 | 2.39 | 2.10 | 2.70 | 1.62 |
| K01849\|Thermococcus sp. 4557 | 1.76 | 1.50 | 1.55 | 1.96 | 2.55 | 1.63 |
| K01849\|Geoglobus acetivorans SBH6 | 1.69 | 1.64 | 1.62 | 1.74 | 2.08 | 1.47 |
| K01849\|Thermosediminibacter oceani | 0.19 | 0.15 | 2.53 | 3.24 | 1.29 | 0.93 |
| K01849\|Thermococcus cleftensis CL1 (Thermococcus sp. CL1) | 1.24 | 0.89 | 1.27 | 1.17 | 1.67 | 1.21 |
| K01849\|Thermococcus piezophilus CDGS | 1.22 | 0.88 | 1.25 | 1.14 | 1.65 | 1.19 |
| K01849\|Thermococcus celer Vu 13 | 1.20 | 0.88 | 1.23 | 1.14 | 1.63 | 1.20 |
| K01849\|Thermococcus barossii SHCK-94 | 1.14 | 0.84 | 1.22 | 1.13 | 1.59 | 1.18 |
| K01849\|Ilyobacter polytropus | 0.84 | 0.48 | 1.77 | 1.60 | 1.33 | 0.91 |
| K01849\|Thermococcus sp. 5-4 | 1.12 | 0.82 | 1.20 | 1.08 | 1.56 | 1.15 |
| K01849\|Thermococcus siculi RG-20 | 1.12 | 0.82 | 1.19 | 1.04 | 1.55 | 1.15 |
| K01849\|Thermoanaerobacter wiegelii | 0.29 | 0.27 | 2.17 | 2.22 | 1.12 | 0.72 |
| K01849\|Thermococcus thioreducens OGL-20P | 1.08 | 0.81 | 1.13 | 1.03 | 1.52 | 1.12 |
| K01849\|Thermococcus radiotolerans EJ2 | 1.04 | 0.79 | 1.09 | 1.01 | 1.47 | 1.09 |
| K01849\|Thermococcus pacificus P-4 | 0.96 | 0.74 | 1.03 | 0.87 | 1.37 | 1.04 |
| K01849\|All others | 10.00 | 8.01 | 31.83 | 31.21 | 22.72 | 14.64 |
| **EC:5.1.99.1** |  |  |  |  |  |  |
| K05606\|Prevotella ruminicola | 634.01 | 610.70 | 581.46 | 611.92 | 576.38 | 463.60 |
| K05606\|Coprococcus catus | 21.28 | 26.95 | 24.82 | 28.37 | 19.82 | 27.01 |
| K05606\|Selenomonas ruminantium | 8.11 | 17.16 | 13.20 | 22.49 | 28.71 | 16.04 |
| K05606\|Bacteroides salanitronis | 12.91 | 15.99 | 13.06 | 14.02 | 14.39 | 9.71 |
| K05606\|Bacteroides cellulosilyticus | 9.76 | 11.07 | 9.91 | 12.32 | 9.67 | 7.29 |
| K05606\|Muribaculum intestinale | 7.63 | 10.90 | 10.24 | 7.61 | 10.44 | 12.41 |
| K05606\|Bacteroides helcogenes | 8.72 | 9.23 | 7.89 | 8.36 | 10.11 | 6.31 |
| K05606\|Bacteroides caecimuris | 8.23 | 9.49 | 8.04 | 9.08 | 8.64 | 6.98 |
| K05606\|Bacteroides zoogleoformans | 7.79 | 7.86 | 9.96 | 7.97 | 8.36 | 8.12 |
| K05606\|Bacteroides fragilis YCH46 | 7.89 | 9.00 | 8.05 | 9.05 | 8.26 | 6.25 |
| K05606\|Bacteroides caccae | 7.63 | 8.95 | 7.86 | 8.55 | 7.97 | 6.22 |
| K05606\|Bacteroides ovatus | 7.63 | 8.95 | 7.86 | 8.55 | 7.97 | 6.22 |
| K05606\|Bacteroides thetaiotaomicron VPI-5482 | 7.63 | 8.95 | 7.86 | 8.55 | 7.97 | 6.22 |
| K05606\|Bacteroides xylanisolvens | 7.63 | 8.95 | 7.86 | 8.55 | 7.97 | 6.22 |
| K05606\|Bacteroides dorei HS1_L_1_B_010 | 7.59 | 9.78 | 6.65 | 8.21 | 6.83 | 6.55 |
| K05606\|Porphyromonas crevioricanis | 7.05 | 7.54 | 6.46 | 8.76 | 6.40 | 3.89 |
| K05606\|Barnesiella viscericola | 6.41 | 4.43 | 5.68 | 8.88 | 8.19 | 5.71 |
| K05606\|Bacteroides heparinolyticus | 5.14 | 4.94 | 4.10 | 4.92 | 6.49 | 3.64 |
| K05606\|Paludibacter propionicigenes | 1.80 | 2.89 | 2.14 | 2.45 | 2.20 | 2.93 |
| K05606\|Cloacibacillus porcorum | 1.17 | 1.95 | 1.92 | 2.64 | 4.18 | 2.10 |
| K05606\|Proteiniphilum saccharofermentans | 1.32 | 1.28 | 1.20 | 1.06 | 1.22 | 0.89 |
| K05606\|All others | 10.93 | 10.51 | 14.63 | 10.77 | 12.69 | 9.16 |

Table S23: List of metagenomes assembled genomes (MAGs) identified in early-lactation dairy cows.

| Bin id | Bin name | Full lineage names |
| --- | --- | --- |
| Bin 1 | PCMP_s.11S_bin.4.fasta | root (no rank)\|cellular organisms (no rank)\|Bacteria (superkingdom)\|FCB group (clade)\|Bacteroidetes/Chlorobi group (clade)\|Bacteroidetes (phylum)\|Bacteroidia (class)\|Bacteroidales (order)\|Prevotellaceae (family)\|Prevotella (genus) |
| Bin 2 | PCMP_s.12S_bin.18.fasta | root (no rank)\|cellular organisms (no rank)\|Bacteria (superkingdom)\|Terrabacteria group (clade)\|Actinobacteria (phylum)\|Coriobacteriia (class) |
| Bin 3 | PCMP_s.12S_bin.34.fasta | root (no rank)\|cellular organisms (no rank)\|Bacteria (superkingdom)\|Terrabacteria group (clade)\|Firmicutes (phylum) |
| Bin 4 | PCMP_s.12S_concoct.23.fasta | root (no rank)\|cellular organisms (no rank)\|Bacteria (superkingdom)\|Terrabacteria group (clade)\|Cyanobacteria/Melainabacteria group (clade)\|Cyanobacteria (phylum)\|unclassified Cyanobacteria (no rank)\|Cyanobacteria bacterium UBA11991 (species)\|\| |
| Bin 5 | PCMP_s.12S_concoct.60.fasta | root (no rank)\|cellular organisms (no rank)\|Archaea (superkingdom)\|Euryarchaeota (phylum)\|Methanomada group (clade)\|Methanobacteria (class)\|Methanobacteriales (order)\|Methanobacteriaceae (family)\|Methanobrevibacter (genus) |
| Bin 6 | PCMP_s.13S_bin.7.fasta | root (no rank)\|cellular organisms (no rank)\|Bacteria (superkingdom)\|Terrabacteria group (clade)\|Firmicutes (phylum)\|Clostridia (class)\|Clostridiales (order) |
| Bin 7 | PCMP_s.14S_concoct.29.fasta | root (no rank)\|cellular organisms (no rank)\|Bacteria (superkingdom)\|FCB group (clade)\|Bacteroidetes/Chlorobi group (clade)\|Bacteroidetes (phylum)\|Bacteroidia (class)\|Bacteroidales (order) |
| Bin 8 | PCMP_s.15S_bin.14.fasta | root (no rank)\|cellular organisms (no rank)\|Bacteria (superkingdom)\|Terrabacteria group (clade)\|Firmicutes (phylum) |
| Bin 9 | PCMP_s.15S_bin.8_sub.fasta | root (no rank)\|cellular organisms (no rank)\|Bacteria (superkingdom)\|FCB group (clade)\|Bacteroidetes/Chlorobi group (clade)\|Bacteroidetes (phylum)\|Bacteroidia (class)\|Bacteroidales (order)\|Prevotellaceae (family) |
| Bin 10 | PCMP_s.15S_concoct.15.fasta | root (no rank)\|cellular organisms (no rank)\|Bacteria (superkingdom)\|Terrabacteria group (clade)\|Firmicutes (phylum)\|Clostridia (class)\|Clostridiales (order) |
| Bin 11 | PCMP_s.15S_concoct.22.fasta | root (no rank)\|cellular organisms (no rank)\|Bacteria (superkingdom)\|FCB group (clade)\|Bacteroidetes/Chlorobi group (clade)\|Bacteroidetes (phylum)\|Bacteroidia (class)\|Bacteroidales (order)\|Prevotellaceae (family) |
| Bin 12 | PCMP_s.17S_concoct.51.fasta | root (no rank)\|cellular organisms (no rank)\|Bacteria (superkingdom)\|FCB group (clade)\|Bacteroidetes/Chlorobi group (clade)\|Bacteroidetes (phylum)\|Bacteroidia (class)\|Bacteroidales (order) |
| Bin 13 | PCMP_s.18S_bin.1.fasta | root (no rank)\|cellular organisms (no rank)\|Bacteria (superkingdom)\|Terrabacteria group (clade)\|Firmicutes (phylum) |
| Bin 14 | PCMP_s.18S_concoct.17.fasta | root (no rank)\|cellular organisms (no rank)\|Bacteria (superkingdom)\|Terrabacteria group (clade)\|Firmicutes (phylum)\|Clostridia (class)\|Clostridiales (order) |
| Bin 15 | PCMP_s.19S_bin.1.fasta | root (no rank)\|cellular organisms (no rank)\|Bacteria (superkingdom)\|Terrabacteria group (clade)\|Firmicutes (phylum)\|Clostridia (class)\|Clostridiales (order)\|Lachnospiraceae (family)\|Butyrivibrio (genus) |
| Bin 16 | PCMP_s.1S_bin.23_sub.fasta | root (no rank)\|cellular organisms (no rank)\|Bacteria (superkingdom)\|Terrabacteria group (clade)\|Firmicutes (phylum)\|Clostridia (class)\|Clostridiales (order) |
| Bin 17 | PCMP_s.20S_maxbin_out.002.fasta | root (no rank)\|cellular organisms (no rank)\|Archaea (superkingdom)\|Euryarchaeota (phylum)\|Methanomada group (clade)\|Methanobacteria (class)\|Methanobacteriales (order)\|Methanobacteriaceae (family)\|Methanobrevibacter (genus) |
| Bin 18 | PCMP_s.21S_concoct.43.fasta | root (no rank)\|cellular organisms (no rank)\|Archaea (superkingdom)\|Euryarchaeota (phylum)\|Methanomada group (clade)\|Methanobacteria (class)\|Methanobacteriales (order)\|Methanobacteriaceae (family)\|Methanobrevibacter (genus) |
| Bin 19 | PCMP_s.22S_bin.11.fasta | root (no rank)\|cellular organisms (no rank)\|Bacteria (superkingdom)\|FCB group (clade)\|Bacteroidetes/Chlorobi group (clade)\|Bacteroidetes (phylum)\|Bacteroidia (class)\|Bacteroidales (order) |
| Bin 20 | PCMP_s.23S_concoct.20.fasta | root (no rank)\|cellular organisms (no rank)\|Bacteria (superkingdom)\|Terrabacteria group (clade)\|Firmicutes (phylum)\|Clostridia (class)\|Clostridiales (order) |
| Bin 21 | PCMP_s.23S_concoct.21.fasta | root (no rank)\|cellular organisms (no rank)\|Bacteria (superkingdom) |
| Bin 22 | PCMP_s.24S_concoct.29.fasta | root (no rank)\|cellular organisms (no rank)\|Bacteria (superkingdom)\|Terrabacteria group (clade)\|Firmicutes (phylum)\|Clostridia (class)\|Clostridiales (order) |
| Bin 23 | PCMP_s.2S_bin.12.fasta | root (no rank)\|cellular organisms (no rank)\|Bacteria (superkingdom)\|Terrabacteria group (clade)\|Firmicutes (phylum) |
| Bin 24 | PCMP_s.2S_bin.2.fasta | root (no rank)\|cellular organisms (no rank)\|Bacteria (superkingdom)\|Terrabacteria group (clade)\|Firmicutes (phylum) |
| Bin 25 | PCMP_s.2S_maxbin_out.010.fasta | root (no rank)\|cellular organisms (no rank)\|Bacteria (superkingdom)\|Terrabacteria group (clade)\|Firmicutes (phylum)\|Clostridia (class)\|Clostridiales (order) |
| Bin 26 | PCMP_s.4S_bin.3.fasta | root (no rank)\|cellular organisms (no rank)\|Bacteria (superkingdom)\|Terrabacteria group (clade)\|Firmicutes (phylum)\|Clostridia (class)\|Clostridiales (order)\|Lachnospiraceae (family)\|Butyrivibrio (genus) |
| Bin 27 | PCMP_s.5S_bin.12.fasta | root (no rank)\|cellular organisms (no rank)\|Bacteria (superkingdom)\|FCB group (clade)\|Bacteroidetes/Chlorobi group (clade)\|Bacteroidetes (phylum)\|Bacteroidia (class)\|Bacteroidales (order)\|Prevotellaceae (family)\|Prevotella (genus) |
| Bin 28 | PCMP_s.5S_concoct.6_sub.fasta | root (no rank)\|cellular organisms (no rank)\|Archaea (superkingdom)\|Euryarchaeota (phylum)\|Methanomada group (clade)\|Methanobacteria (class)\|Methanobacteriales (order)\|Methanobacteriaceae (family)\|Methanobrevibacter (genus) |
| Bin 29 | PCMP_s.6S_concoct.19.fasta | root (no rank)\|cellular organisms (no rank)\|Archaea (superkingdom)\|Euryarchaeota (phylum)\|Methanomada group (clade)\|Methanobacteria (class)\|Methanobacteriales (order)\|Methanobacteriaceae (family)\|Methanobrevibacter (genus) |
| Bin 30 | PCMP_s.6S_concoct.51.fasta | root (no rank)\|cellular organisms (no rank)\|Bacteria (superkingdom)\|Terrabacteria group (clade)\|Firmicutes (phylum)\|Clostridia (class)\|Clostridiales (order) |
| Bin 31 | PCMP_s.7S_concoct.21.fasta | root (no rank)\|cellular organisms (no rank)\|Bacteria (superkingdom)\|Terrabacteria group (clade)\|Firmicutes (phylum)\|Negativicutes (class)\|Acidaminococcales (order)\|Acidaminococcaceae (family)\|Succiniclasticum (genus)\|Succiniclasticum ruminis* (species) |
| Bin 32 | PCMP_s.8S_bin.17.fasta | root (no rank)\|cellular organisms (no rank)\|Bacteria (superkingdom)\|FCB group (clade)\|Bacteroidetes/Chlorobi group (clade)\|Bacteroidetes (phylum)\|Bacteroidia (class)\|Bacteroidales (order)\|Prevotellaceae (family) |
| Bin 33 | PCMP_s.8S_concoct.39.fasta | root (no rank)\|cellular organisms (no rank)\|Bacteria (superkingdom) |
| Bin 34 | PCMP_s.9S_concoct.44.fasta | root (no rank)\|cellular organisms (no rank)\|Bacteria (superkingdom)\|Terrabacteria group (clade)\|Firmicutes (phylum)\|Negativicutes (class)\|Acidaminococcales (order)\|Acidaminococcaceae (family)\|Succiniclasticum (genus)\|Succiniclasticum ruminis* (species) |
| Bin 35 | cnt_w12_20_bin_44.fasta | root (no rank)\|cellular organisms (no rank)\|Bacteria (superkingdom)\|Terrabacteria group (clade)\|Firmicutes (phylum)\|Clostridia (class)\|Clostridiales (order)\|Lachnospiraceae (family)\|unclassified Lachnospiraceae (no rank)\|Lachnospiraceae bacterium NE2001 (species) |
| Bin 36 | cnt_w12_21_concoct_24.fasta | root (no rank)\|cellular organisms (no rank)\|Archaea (superkingdom)\|Euryarchaeota (phylum)\|Methanomada group (clade)\|Methanobacteria (class)\|Methanobacteriales (order)\|Methanobacteriaceae (family)\|Methanobrevibacter (genus) |
| Bin 37 | cnt_w12_2_concoct_156_sub.fasta | root (no rank)\|cellular organisms (no rank)\|Bacteria (superkingdom)\|Terrabacteria group (clade)\|Firmicutes (phylum)\|Clostridia (class)\|Clostridiales (order) |
| Bin 38 | cnt_w12_3_bin_27.fasta | root (no rank)\|cellular organisms (no rank)\|Bacteria (superkingdom)\|Terrabacteria group (clade) |
| Bin 39 | cnt_w12_5_bin_14.fasta | root (no rank)\|cellular organisms (no rank)\|Bacteria (superkingdom)\|Terrabacteria group (clade)\|Firmicutes (phylum) |
| Bin 40 | cnt_w12_6_concoct_120.fasta | root (no rank)\|cellular organisms (no rank)\|Bacteria (superkingdom)\|Terrabacteria group (clade)\|Firmicutes (phylum) |
| Bin 41 | cnt_w4_11_bin_88.fasta | root (no rank)\|cellular organisms (no rank)\|Bacteria (superkingdom)\|Terrabacteria group (clade)\|Firmicutes (phylum)\|Clostridia (class)\|Clostridiales (order) |
| Bin 42 | cnt_w4_4_concoct_171.fasta | root (no rank)\|cellular organisms (no rank)\|Bacteria (superkingdom)\|Terrabacteria group (clade)\|Firmicutes (phylum) |
| Bin 43 | cnt_w4_9_bin_86.fasta | root (no rank)\|cellular organisms (no rank)\|Bacteria (superkingdom)\|Terrabacteria group (clade)\|Actinobacteria (phylum)\|Actinobacteria (class)\|Bifidobacteriales (order)\|Bifidobacteriaceae (family)\|Bifidobacterium (genus) |
| Bin 44 | cnt_w8_10_concoct_93.fasta | root (no rank)\|cellular organisms (no rank)\|Bacteria (superkingdom)\|Terrabacteria group (clade)\|Firmicutes (phylum)\|Clostridia (class)\|Clostridiales (order) |
| Bin 45 | cnt_w8_17_concoct_87.fasta | root (no rank)\|cellular organisms (no rank)\|Archaea (superkingdom)\|Euryarchaeota (phylum)\|Methanomada group (clade)\|Methanobacteria (class)\|Methanobacteriales (order)\|Methanobacteriaceae (family)\|Methanosphaera (genus) |
| Bin 46 | cnt_w8_19_bin_86.fasta | root (no rank)\|cellular organisms (no rank)\|Bacteria (superkingdom)\|Terrabacteria group (clade)\|Firmicutes (phylum)\|Clostridia (class)\|Clostridiales (order)\|Lachnospiraceae (family)\|unclassified Lachnospiraceae (no rank)\|Lachnospiraceae bacterium (species) |
| Bin 47 | cnt_w8_1_bin_37.fasta | root (no rank)\|cellular organisms (no rank)\|Bacteria (superkingdom)\|Terrabacteria group (clade)\|Firmicutes (phylum) |
| Bin 48 | cnt_w8_23_concoct_137.fasta | root (no rank)\|cellular organisms (no rank)\|Bacteria (superkingdom)\|Terrabacteria group (clade)\|Firmicutes (phylum) |
| Bin 49 | cnt_w8_6_bin_11_sub.fasta | root (no rank)\|cellular organisms (no rank)\|Bacteria (superkingdom)\|Terrabacteria group (clade)\|Firmicutes (phylum)\|Clostridia (class)\|Clostridiales (order)\|Lachnospiraceae (family)\|Pseudobutyrivibrio (genus) |
| Bin 50 | cnt_w8_7_bin_50_sub.fasta | root (no rank)\|cellular organisms (no rank)\|Bacteria (superkingdom)\|Terrabacteria group (clade)\|Firmicutes (phylum)\|Clostridia (class)\|Clostridiales (order)\|Clostridiaceae (family)\|Sarcina (genus)\|unclassified Sarcina* (no rank) |
| Bin 51 | trt_w12_2_bin_63.fasta | root (no rank)\|cellular organisms (no rank)\|Bacteria (superkingdom)\|Terrabacteria group (clade)\|Firmicutes (phylum)\|Clostridia (class)\|Clostridiales (order) |
| Bin 52 | trt_w12_4_concoct_98.fasta | root (no rank)\|cellular organisms (no rank)\|Bacteria (superkingdom)\|Terrabacteria group (clade)\|Firmicutes (phylum) |
| Bin 53 | trt_w12_7_concoct_22.fasta | root (no rank)\|cellular organisms (no rank)\|Bacteria (superkingdom)\|FCB group (clade)\|Bacteroidetes/Chlorobi group (clade)\|Bacteroidetes (phylum)\|Bacteroidia (class)\|Bacteroidales (order) |
| Bin 54 | trt_w4_5_bin_59.fasta | root (no rank)\|cellular organisms (no rank)\|Bacteria (superkingdom)\|Terrabacteria group (clade)\|Firmicutes (phylum)\|Clostridia (class)\|Clostridiales (order) |
| Bin 55 | trt_w4_8_bin_9_sub.fasta | root (no rank)\|cellular organisms (no rank)\|Bacteria (superkingdom)\|Terrabacteria group (clade)\|Firmicutes (phylum) |
| Bin 56 | trt_w4_9_concoct_150.fasta | root (no rank)\|cellular organisms (no rank)\|Bacteria (superkingdom) |
| Bin 57 | trt_w8_10_bin_17_sub.fasta | root (no rank)\|cellular organisms (no rank)\|Bacteria (superkingdom)\|Terrabacteria group (clade)\|Firmicutes (phylum) |
| Bin 58 | trt_w8_14_concoct_100.fasta | root (no rank)\|cellular organisms (no rank)\|Bacteria (superkingdom)\|Terrabacteria group (clade)\|Firmicutes (phylum) |
| Bin 59 | trt_w8_18_concoct_87.fasta | root (no rank)\|cellular organisms (no rank)\|Bacteria (superkingdom)\|Terrabacteria group (clade)\|Firmicutes (phylum)\|Clostridia (class)\|Clostridiales (order) |
| Bin 60 | trt_w8_21_bin_20.fasta | root (no rank)\|cellular organisms (no rank)\|Bacteria (superkingdom)\|Terrabacteria group (clade)\|Firmicutes (phylum)\|Clostridia (class)\|Clostridiales (order)\|Clostridiaceae (family)\|Sarcina (genus)\|unclassified Sarcina* (no rank) |
| Bin 61 | trt_w8_7_concoct_37.fasta | root (no rank)\|cellular organisms (no rank)\|Bacteria (superkingdom)\|Terrabacteria group (clade)\|Firmicutes (phylum)\|Clostridia (class)\|Clostridiales (order) |

**Table S24:** Transcripts (cpm; copies per million) coding for EC: 1.8.98.1 in cows supplemented with 3-nitrooxypropanol (3-NOP) compared to control cows at weeks 4, 8, and 12. ND = not detected.

|  | **Week 4** | | **Week 8** | | **Week 12** | | |
| --- | --- | --- | --- | --- | --- | --- | --- |
|  | **Control** | **3-NOP** | **Control** | **3-NOP** | **Control** | **3-NOP** |  |
| **Step 8b \| 1.8.98.1 Co-B,**  **Co-M:methanophenazine oxidoreductase** |  |  |  |  |  |  |  |
| **K08264 (heterodisulfide reductase subunit D) - HdrD** | **6.90** | **5.18** | **5.43** | **5.56** | **5.15** | **7.92** |  |
| K08264 \| *Methanogenic archaeon ISO4-H5* | 6.03 | 4.59 | 4.78 | 5.18 | 4.58 | 7.22 |  |
| K08264 \| *Thermoplasmatales archaeon BRNA1* | 0.57 | 0.31 | 0.37 | 0.21 | 0.33 | 0.38 |  |
| K08264 \| *Candidatus Methanomethylophilus alvus Mx1201* | 0.25 | 0.23 | 0.24 | 0.16 | 0.24 | 0.22 |  |
| K08264 \| *Methanosarcina thermophila CHTI-55* | 0.01 | 0.04 | 0.00 | 0.00 | 0.00 | 0.01 |  |
| K08264 \| *Candidatus Methanoplasma termitum MpT1* | 0.00 | 0.00 | 0.03 | 0.00 | 0.00 | 0.00 |  |
| K08265 (heterodisulfide reductase subunit E) - HdrE | ND | ND | ND | ND | ND | ND |  |

**Table S25:** Transcripts (cpm; copies per million) coding for EC: 1.8.98.5 in cows supplemented with 3-nitrooxypropanol (3-NOP) compared to control cows at weeks 4, 8, and 12. ND = not detected.

|  | **Week 4** | | **Week 8** | | **Week 12** | |
| --- | --- | --- | --- | --- | --- | --- |
| **Step 8d \| 1.8.98.5**  **(Co-B, Co-M, ferredoxin:H_2_ oxidoreductase)** | Control | 3-NOP | Control | 3-NOP | Control | 3-NOP |
| **K14126 (F420-non-reducing hydrogenase large subunit)** | **1228.11** | **1031.67** | **1370.01** | **887.93** | **995.02** | **847.54** |
| K14126 \| *Methanobrevibacter ruminantium M1* | 321.57 | 242.29 | 264.01 | 160.30 | 172.06 | 131.13 |
| K14126 \| *Methanobrevibacter olleyae YLM1* | 211.47 | 205.15 | 237.55 | 162.55 | 160.35 | 162.09 |
| K14126 \| *Methanobrevibacter sp. YE315* | 163.31 | 93.81 | 172.48 | 89.72 | 133.19 | 87.93 |
| K14126 \| *Methanobrevibacter millerae SM9* | 119.72 | 70.50 | 122.75 | 76.24 | 101.82 | 72.46 |
| K14126 \| *Methanobrevibacter smithii ATCC 35061* | 52.09 | 63.40 | 84.62 | 60.87 | 60.67 | 65.56 |
| K14126 \| *Methanosphaera stadtmanae DSM 3091* | 51.58 | 53.01 | 78.06 | 52.75 | 61.29 | 42.85 |
| K14126 \| *Methanosphaera sp. BMS* | 43.42 | 45.20 | 66.30 | 46.30 | 51.87 | 35.15 |
| K14126 \| *Methanobrevibacter sp. AbM4* | 28.75 | 28.10 | 40.95 | 26.67 | 29.46 | 27.07 |
| K14126 \| *Methanobacterium lacus (Methanobacterium sp. AL-21)* | 35.92 | 29.29 | 35.12 | 22.42 | 25.40 | 20.18 |
| K14126 \| *Methanobacterium congolense isolate Buetzberg* | 28.38 | 22.19 | 26.67 | 17.25 | 18.80 | 15.19 |
| K14126 \| *Methanobacterium paludis (Methanobacterium sp. SWAN-1)* | 28.07 | 20.45 | 26.38 | 15.61 | 18.70 | 14.05 |
| **K14127 (F420-non-reducing hydrogenase iron-sulfur subunit)** | **1409.77** | **1509.81** | **1970.35** | **1411.82** | **1490.49** | **1435.43** |
| K14127 \| *Methanobrevibacter olleyae YLM1* | 247.87 | 293.08 | 339.78 | 256.50 | 236.90 | 267.54 |
| K14127 \| *Methanobrevibacter ruminantium M1* | 245.35 | 290.85 | 339.03 | 255.42 | 236.37 | 267.14 |
| K14127 \| *Methanobrevibacter sp. YE315* | 168.71 | 166.43 | 228.13 | 156.16 | 180.69 | 168.12 |
| K14127 \| *Methanobrevibacter smithii ATCC 35061* | 159.93 | 146.26 | 205.47 | 130.10 | 156.67 | 143.38 |
| K14127 \| *Methanobrevibacter millerae SM9* | 111.60 | 130.56 | 163.98 | 130.88 | 127.02 | 142.35 |
| K14127 \| *Methanosphaera sp. BMS* | 104.58 | 113.18 | 189.51 | 134.89 | 150.53 | 99.78 |
| K14127 \| *Methanosphaera stadtmanae DSM 3091* | 72.44 | 77.29 | 126.67 | 90.45 | 100.87 | 61.00 |
| K14127 \| *Methanobrevibacter sp. AbM4* | 52.71 | 47.35 | 61.97 | 39.01 | 48.44 | 40.69 |
| **K14128 (F420-non-reducing hydrogenase small subunit)** | **1269.56** | **1097.96** | **1431.32** | **937.00** | **1057.45** | **878.48** |
| K14128 \| *Methanobrevibacter ruminantium M1* | 348.46 | 251.28 | 278.04 | 173.74 | 183.42 | 136.16 |
| K14128 \| *Methanobrevibacter olleyae YLM1* | 244.41 | 213.83 | 246.33 | 159.25 | 166.78 | 146.36 |
| K14128 \| *Methanobrevibacter smithii ATCC 35061* | 154.67 | 182.54 | 236.05 | 172.81 | 175.12 | 187.89 |
| K14128 \| *Methanobrevibacter millerae SM9* | 150.41 | 108.75 | 163.46 | 105.71 | 135.67 | 107.40 |
| K14128 \|*Methanobrevibacter sp. YE315* | 137.43 | 78.33 | 139.37 | 73.30 | 115.19 | 76.52 |
| K14128 \| *Methanosphaera sp. BMS* | 77.96 | 79.78 | 117.22 | 86.45 | 95.14 | 57.15 |
| K14128 \| *Methanosphaera stadtmanae DSM 3091* | 57.97 | 60.40 | 86.83 | 53.09 | 69.45 | 40.64 |
| K14128 \| *Methanobrevibacter sp. AbM4* | 18.92 | 27.84 | 38.11 | 25.04 | 26.85 | 29.41 |

**Table S26:** Transcripts (cpm; copies per million) coding for EC: 1.8.98.6 in cows supplemented with 3-nitrooxypropanol (3-NOP) compared to control cows at weeks 4, 8, and 12. ND = not detected.

|  | **Week 4** | | **Week 8** | | **Week 12** | | |
| --- | --- | --- | --- | --- | --- | --- | --- |
| **Step 8e \| 1.8.98.6** | **Control** | **3-NOP** | **Control** | **3-NOP** | **Control** | **3-NOP** |  |
| **K00125 (formate dehydrogenase [coenzyme F420] alpha subunit)** | **994.36** | **773.73** | **1086.36** | **727.74** | **766.82** | **551.83** |  |
| K00125 \| *Methanobrevibacter ruminantium M1* | 328.34 | 299.72 | 363.06 | 260.34 | 234.19 | 192.90 |  |
| K00125 \| *Methanobrevibacter sp. YE315* | 285.05 | 106.49 | 256.34 | 101.89 | 196.43 | 75.18 |  |
| K00125 \| *Methanobrevibacter olleyae YLM1* | 209.10 | 175.68 | 213.27 | 146.42 | 140.25 | 103.22 |  |
| K00125 \| *Methanobrevibacter sp. AbM4* | 70.22 | 111.69 | 145.00 | 117.23 | 102.65 | 98.72 |  |
| K00125 \| *Methanobrevibacter millerae SM9* | 55.34 | 51.47 | 58.32 | 73.23 | 56.38 | 60.24 |  |
| K00125 \| *Methanobrevibacter smithii ATCC 35061* | 43.09 | 26.31 | 44.52 | 26.64 | 33.39 | 19.52 |  |
| **K22516 (formate dehydrogenase [coenzyme F420] beta subunit)** | **1044.91** | **1162.98** | **1480.52** | **1173.82** | **1041.17** | **938.77** |  |
| K22516 \| *Methanobrevibacter ruminantium M1* | 346.29 | 423.40 | 491.05 | 403.88 | 327.24 | 314.48 |  |
| K22516 \| *Methanobrevibacter olleyae YLM1* | 277.76 | 399.92 | 468.56 | 408.71 | 314.71 | 324.88 |  |
| K22516 \| *Methanobrevibacter millerae SM9* | 262.51 | 159.20 | 268.55 | 165.60 | 210.95 | 130.85 |  |
| K22516 \| *Methanobrevibacter smithii ATCC 35061* | 134.28 | 148.58 | 205.65 | 158.51 | 153.29 | 135.81 |  |
| K22516 \| *Methanobrevibacter sp. AbM4* | 11.96 | 22.68 | 31.86 | 26.63 | 22.62 | 23.76 |  |
